# Rapid MinION profiling of preterm microbiota and antimicrobial-resistant pathogens

**DOI:** 10.1038/s41564-019-0626-z

**Published:** 2019-12-16

**Authors:** Richard M. Leggett, Cristina Alcon-Giner, Darren Heavens, Shabhonam Caim, Thomas C. Brook, Magdalena Kujawska, Samuel Martin, Ned Peel, Holly Acford-Palmer, Lesley Hoyles, Paul Clarke, Lindsay J. Hall, Matthew D. Clark

**Affiliations:** 1 Earlham Institute, Norwich Research Park, Norwich, UK; 2 Quadram Institute Bioscience, Norwich Research Park, Norwich, UK; 3 University of Westminster, London, UK; 4 Nottingham Trent University, Nottingham, UK; 5 Norfolk and Norwich University Hospital, Norwich, UK; 6 Norwich Medical School, University of East Anglia, Norwich, UK; 7 Natural History Museum, London, UK

**Keywords:** Microbiome, Antimicrobial resistance, DNA sequencing, Translational research, Clinical microbiology

## Abstract

The MinION sequencing platform offers near real-time analysis of DNA sequence; this makes the tool attractive for deployment in fieldwork or clinical settings. We used the MinION platform coupled to the NanoOK RT software package to perform shotgun metagenomic sequencing and profile mock communities and faecal samples from healthy and ill preterm infants. Using Nanopore data, we reliably classified a 20-species mock community and captured the diversity of the immature gut microbiota over time and in response to interventions such as probiotic supplementation, antibiotic treatment or episodes of suspected sepsis. We also performed rapid real-time runs to assess gut-associated microbial communities in critically ill and healthy infants, facilitated by NanoOK RT software package, which analysed sequences as they were generated. Our pipeline reliably identified pathogenic bacteria (that is, *Klebsiella pneumoniae* and *Enterobacter cloacae*) and their corresponding antimicrobial resistance gene profiles within as little as 1 h of sequencing. Results were confirmed using pathogen isolation, whole-genome sequencing and antibiotic susceptibility testing, as well as mock communities and clinical samples with known antimicrobial resistance genes. Our results demonstrate that MinION (including cost-effective Flongle flow cells) with NanoOK RT can process metagenomic samples to a rich dataset in < 5 h, which creates a platform for future studies aimed at developing these tools and approaches in clinical settings with a focus on providing tailored patient antimicrobial treatment options.

## Abstract (web summary)

Shotgun metagenomic sequencing (using the MinION platform) of mock microbial communities and faecal samples from healthy and ill preterm infants can be used to identify pathogens and their antimicrobial resistance gene profiles in real time, indicating the potential for translation into clinical settings.

## Main

Next-generation sequencing (NGS) has revolutionized the profiling of environmental and clinical microbial communities. The culture-independent, sensitive, data-rich nature of metagenomic sequencing, combined with powerful bioinformatics tools, have allowed researchers to differentiate patient groups from healthy individuals based on their microbial profiles^1–6^, including those with increased risk of pathogen overgrowth^7^. Metagenomics also allows the identification of functional traits, for example, antibiotic resistance genes, which are important in light of the antimicrobial resistance (AMR) threat^8–10^. Optimization of metagenomic methodologies and bioinformatics tools could allow the identification of at-risk individuals, profiling of infectious agents and tailoring of treatments^11^.

In contrast to many NGS platforms, which require large capital investments and numerous samples to be multiplexed, newer sequencing platforms such as the MinION by Oxford Nanopore Technologies (ONT) represent inexpensive portable sequencing devices capable of producing long reads^12^. The real-time nature of data generation could provide users with a rapid screening platform; however, this real-time functionality and a different error profile require development of methods and bioinformatics pipelines, particularly for the clinical arena.

Despite technical challenges in metagenomic profiling and diagnostics^13^, MinIONs have been successfully used in medical research on low-complexity samples including: outbreak surveillance^14^; characterization of bacterial isolates^15^; and low microbial biomass samples^16,17^. Diagnostics in metagenomic samples is still challenging due to lower MinION sequence yields and accuracy, but essential since many clinical samples are complex. To date no studies have explored MinION technology in clinical gut metagenomic samples. For such applications, it is important to confidently identify (1) species-level profiles, (2) species abundance within the microbiota and (3) AMR gene repertoires. The development of a software tool, NanoOK RT, allowed us to perform real-time analysis and benchmark MinION-based metagenomics using mock communities and clinical samples from healthy and ill preterm infants. These studies allowed us to determine longitudinal microbiota profiles, gut-associated pathogens linked with sepsis or necrotizing enterocolitis (NEC) and their AMR profiles.

## Results

### Accurate classification of a microbial mock community using MinION sequencing

**Fig. 1:**
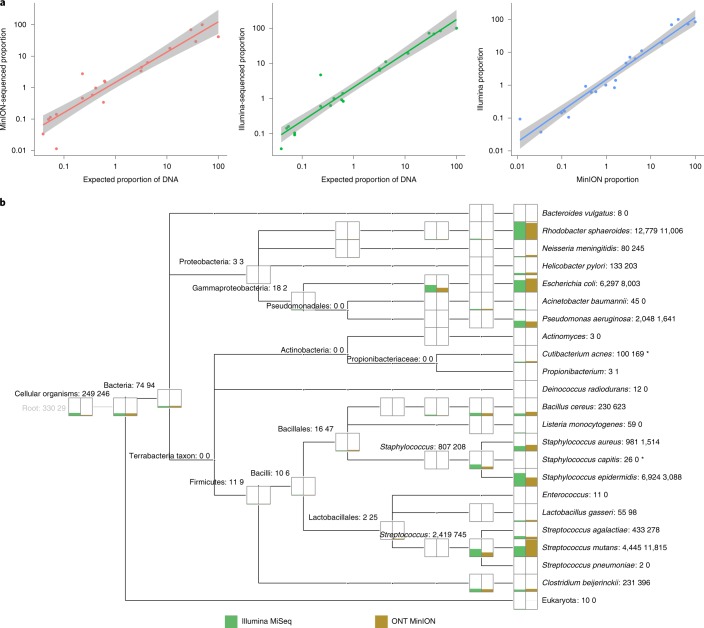
Sequencing of microbial mock community (HM-277D) with Illumina and MinION sequencing. **a**, Correlation plot comparing the expected proportions of DNA from each of the 20 species in the mock community with the proportions of sequence obtained by MinION (left, log-transformed Pearson’s *r* = 0.94) and Illumina platforms (log-transformed Pearson’s *r* = 0.97). The right-hand plot shows the correlation between MinION and Illumina (log-transformed Pearson’s *r* = 0.98). The grey region either side of the fit line represents the 95% CIs. **b**, MEGAN^37^ taxonomy tree representing taxa assigned from the mock community as sequenced by Illumina MiSeq (green) and ONT MinION (brown). The height of the bars indicates the relative number of reads assigned, with normalized counts given next to the taxa name. Asterisks represent species assigned by MEGAN but not specified as members of the mock community. Numbers assigned to taxa correspond to Illumina MiSeq (left) and ONT MinION (right) normalized counts, respectively.

We benchmarked MinION technology by profiling a bacterial mock community using R7.3 flow cells. Reads were analysed with NanoOK^18^ and produced alignments to the 20 microbial reference sequences with 82–89% identity^19^. Coverage ranged from almost 0 × (8 reads) of *Actinomyces odontolyticus* to 13 × (7,695 reads) of *Streptococcus mutans*, which is consistent with expected mock concentrations (Supplementary Table 1). Benchmarking to Illumina sequencing demonstrated high correlation with expected proportions (Fig. 1a, log-transformed Pearson’s *r* = 0.94 for MinION and 0.97 for Illumina), and with each other (log-transformed Pearson’s *r* = 0.98). Broadly similar abundance levels across both platforms were observed, with some differences in assignment to species versus genus/family (Fig. 1b). This is probable since the longer length Nanopore reads should provide better specificity; however, in some cases lower Nanopore per base accuracy may reduce the ability to discriminate between closely related species.

### Monitoring microbial disturbances in the preterm gut microbiota using MinION

**Fig. 2:**
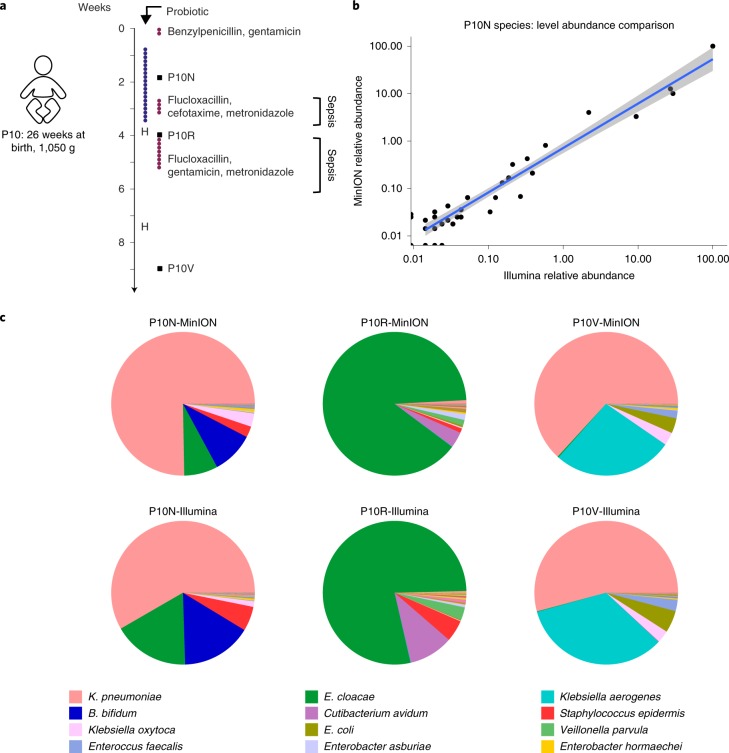
Longitudinal study on preterm infant P10 using MinION and Illumina sequencing. **a**, Timeline diagram of preterm P10 indicating the times of faecal sample collection (P10N, P10R and P10V), duration of antibiotic and probiotic treatment and relevant clinical observations. The diagram is divided into weeks and the dots represent days within the timescale. The blue dots represent the days of probiotic treatment; the red dots represent the days of antibiotic treatment; the black squares represent the time points for sample collection; and the letter H represents the transfer of the preterm infant to another hospital. **b**, Correlation plot of normalized species abundance in taxonomic assignments of Illumina (*x* axis) and MinION (*y* axis) data for sample P10N, with log-transformed Pearson’s *r* = 0.95 (taxa *n* = 92). The values for P10R and P10V were 0.90 and 0.94, respectively. The grey region either side of the fit line represents the 95% CIs. **c**, Taxonomic profiles at time points P10N, P10R and P10V, as assigned by MEGAN. The top row corresponds to the results obtained using MinION sequencing; the bottom row displays the results obtained using Illumina HiSeq. The legend comprises the 12 most abundant taxa obtained. Further information on all taxa and corresponding read counts can be found in Supplementary Table 3.

**Extended Data Fig. 1:**
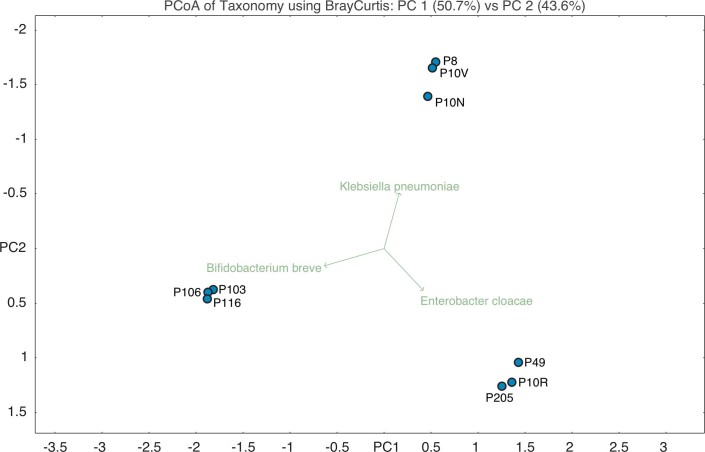
Principal Coordinate Analysis (PCoA) based on taxonomic assignments of MinION preterm faecal samples. PCoA was performed in MEGAN using the species-level taxonomic assignments of the preterm infant samples (n=9 biologically independent samples). Green arrows indicate the biplot loading vectors determined by MEGAN (the taxa with the largest influence in the plot). Samples were drawn from two groups: (i) healthy preterms (P106, P103 and P116) and (ii) preterms diagnosed with NEC or sepsis (P8, P49, P205 and P10). The PCoA plot indicates that distribution of samples from healthy preterms was distinct from samples belonging to infants diagnosed with suspected sepsis or NEC.

We next tested if MinION technology could be used for real metagenomic samples, profiling eight preterm infants (three healthy and five diagnosed with suspected sepsis or NEC; Supplementary Figs. 1 and 2 and Fig. 2a). These infants are born with underdeveloped gut physiology and immunity, and have an altered gut microbiota; this increases the risk of life-threatening infections^20,21^. Principal coordinates analysis (PCoA) of faecal samples indicated three distinct clusters, driven by the presence of either beneficial *Bifidobacterium breve* or potentially pathogenic microbiota members *Enterobacter* *cloacae*^22^ or *Klebsiella* *pneumoniae* (Extended Data Fig. 1).

**Extended Data Fig. 2:**
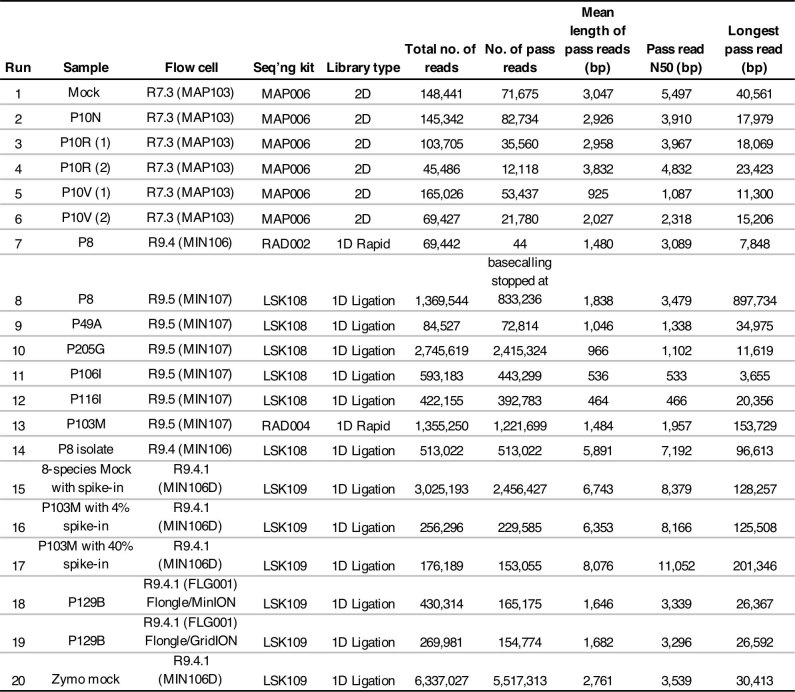
Nanopore flow cell versions and yields. Yield and length metrics for Nanopore runs. Flow cell for sample P8 (2D) was used for two experiments: (a) Initial Metrichor basecalled run which was abandoned due to network lag and (b) Local basecalled run which was used for subsequent analysis. Runs 1-8 were performed using an earlier version of MinKNOW (ONT’s control software) which classified reads into “pass” and “fail” according to quality. Runs 9-14 used a later version of MinKNOW which removes the quality distinction, however some reads can still fail basecalling. The low yield for run 10 (P49A) is due to the flow cell having a low number of active pores at QC.

We carried out longitudinal profiling of a preterm infant patient (P10) at days 13, 28 and 64 after birth (Fig. 2a). Comparing MinION (R7.3) to Illumina shotgun metagenomics sequencing confirmed that MinION sequencing depth was sufficient to capture the complete species diversity of the samples (Extended Data Fig. 2 and Supplementary Fig. 3). Taxonomic assignments using MinION versus Illumina shotgun data were comparable (species-level, log-transformed Pearson’s *r* = 0.95, *r*  = 0.90 and *r*  = 0.94 for P10N, P10R and P10V respectively; Fig. 2b,c), for example, *Klebsiella*, *Enterobacter*, *Enterococcus*, *Veillonella*, *Staphylococcus* and *Bifidobacterium*, which correlated to probiotic supplementation or suspected sepsis periods. These data highlight the potential for MinION shotgun metagenomics to confirm the impact of interventions (for example, probiotic supplementation) and profile potential pathogenic microbes.

**Extended Data Fig. 3:**
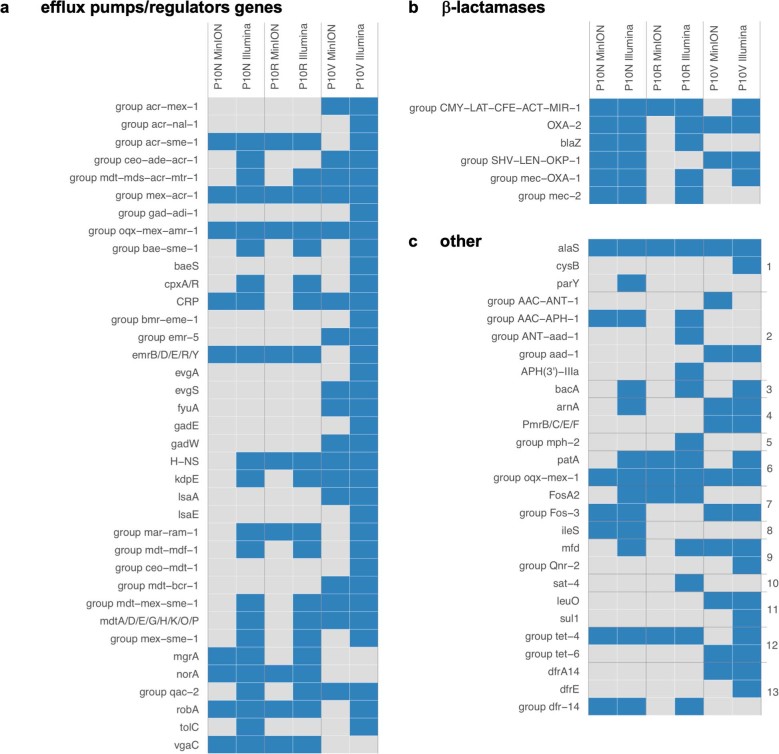
Presence/absence heat maps of AMR hits found in P10 using MinION and Illumina. Three samples of preterm P10 were analysed and sequenced using MinION (P10N-N, P10R-N and P10V-N), and Illumina technology (P10N-I, P10R-I and P10V-I). (**a**) Heat map representing efflux pumps inhibitors or regulators genes found in samples P10N, P10R and P10V. (**b**) Heat map highlighting b-lactamases. (**c**) Heat map showing (1) aminocoumarin resistance genes (2) aminoglycosides resistance (3) bacitracin resistance (4) colistin resistance (5) erythromycin resistance (6) fluoroquinolone resistance (7) fosfomycin resistance (8) mupirocin resistance (9) quinolone resistance (10) streptothricin resistance (11) sulphonamide resistance (12) tetracycline resistance (13) trimethoprim resistance. AMR genes were grouped according to sequence similarity. Blue indicates presence, grey indicates absence. Further information on genes detected can be found in Supplementary Table 2.

Antibiotics can lead to disruption of the gut microbiota and create a selection pressure that may change the profile of AMR genes (the resistome)^23^. We determined AMR profiles by comparing MinION to Illumina results. To avoid overcalling numerous subtypes of resistance genes (due to the higher error rates of Nanopore sequencing), we grouped together those detected genes that shared sequence similarity (see Methods for details). Classifying AMR genes by mode of action indicated comparable detection efficiency of MinION and Illumina. However, since Illumina datasets were capped at 1 million reads, whereas MinION datasets ranged from 48,000 to 83,000, three low-abundance genes/groups with unique resistance mechanisms (*bacA*, *sat4* and group *mph2*) were only detected by the deeper Illumina sequencing (Extended Data Fig. 3). Overall, four AMR classes—efflux pumps, β-lactamases, aminoglycosides and fluoroquinolones—were particularly prevalent (Supplementary Fig. 4), with MinION technology able to detect species-specific AMR genes, for example, *ileS* encoding mupirocin^24^ resistance in *Bifidobacterium* or *fosA2* (ref. ^25^) encoding fosfomycin resistance in *E. cloacae* (Supplementary Table 2).

**Extended Data Fig. 4:**
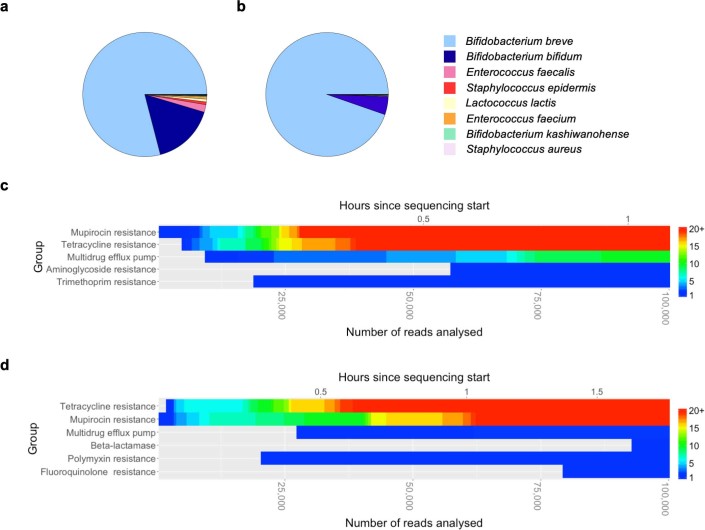
Rapid diagnostic of healthy preterms P106 and P116 receiving probiotic supplementation. (**a**), (**b**) Taxonomic profiles obtained using MinION technology for preterms P106 and P116, respectively. Figure legend comprises the 8 most abundant taxa represented. Further information on number of reads classified can be found in Supplementary Table 3. (**c**), (**d**) Heat maps displaying number of CARD database hits detected among the most common groups of antibiotic resistance genes found in preterms P106 and P116. Top and lower panel indicate the hours since sequencing started and the number of reads analyzed, respectively within this timeframe.

Further sequencing and analysis (using newer R9.4 flow cells) also allowed accurate taxonomic profiling; the gut microbiota of healthy infants P106 and P116 were dominated with *B. breve* and *Bifidobacterium* *bifidum* (Extended Data Fig. 4a,b), with a correspondingly limited resistome, consistent with beneficial taxonomic profiles and short antibiotic treatments (Extended Data Fig. 4c,d). These data indicate that MinION technology can profile preterm gut metagenomic samples, including determination of known species (that is, *B. bifidum*) and AMR profiles.

### Bioinformatics tools use MinION-specific features to rapidly characterize gut-associated bacteria and antibiotic resistance profiles

MinIONs provide near real-time sequencing and longer reads than Illumina sequencing, but the available software must take advantage of these useful features. To improve speed and incorporate bespoke analyses, we added real-time functionality to the NanoOK software (v.0.95)^18^ thereby creating NanoOK RT, which aligns reads to bacterial and AMR databases as they are generated. A second tool, NanoOK Reporter, provides a graphical user interface to view results and performs walkout analysis from AMR genes into the flanking DNA of host bacteria (see Methods).

**Fig. 3:**
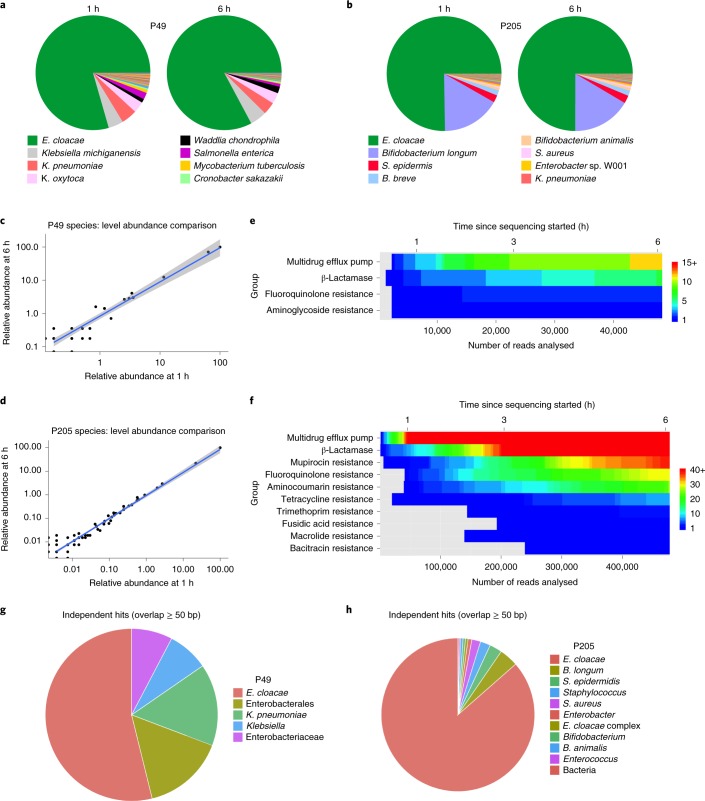
Rapid diagnostic using MinION technology for preterm infants clinically diagnosed with suspected NEC (P49 and P205). **a**,**b**, Taxonomic profiles comparing the results obtained at 1 h and 6 h for P49 (**a**) and P205 (**b**) after sequencing started. The pie chart legends comprise the eight most abundant taxa. Detailed counts can be found in Supplementary Table 3. **c**,**d**, Correlation plots representing normalized assigned reads at 1 h and 6 h for P49 (taxa *n* = 35, log-transformed Pearson’s *r* = 0.97) (**c**) and P205 (taxa *n* = 120, log-transformed Pearson’s *r* = 0.98) (**d**). The grey region either side of the fit line represents the 95% CIs. **e**,**f**, Heat maps displaying the number of CARD hits detected among the most common groups of antibiotic resistance genes found in preterm P49 (**e**) and P205 (**f**). Further information on all the AMR genes classified can be found in Supplementary Table 4. **g**,**h**, Walkout results for preterm infants P49 (**g**) and P205 (**h**) at 6 h, as reported by the NanoOK RT’s walkout option. Results shown are for independent bacterial hits (defined as ≥ 50 bp away from the AMR sequence), at 6 h of sequencing.

To test these tools, we profiled samples from preterm infants who were clinically diagnosed with NEC^26^ (Supplementary Fig. 2a,b). Samples from infants P49 and P205 both contained high proportions of *E. cloacae* (Fig. 3a,b), with the correlation plots of normalized reads assigned at 1 and 6 h being almost identical (log-transformed Pearson’s *r* = 0.97 for P49 and *r* = 0.98 for P205; Fig. 3c,d). Resistome analysis highlighted a substantial number of AMR genes and classes (that is, efflux pump and β-lactamases), which were detected within minutes of the start of sequencing (Fig. 3e,f). Although these infants had gut microbiota dominated by *E. cloacae*, they also harboured other potentially pathogenic bacteria, highlighting the importance of determining which bacteria are harbouring AMR genes if these approaches are to be developed for more clinically based analysis.

Since MinION reads are typically longer than Illumina reads, we reasoned that we could extract additional information by examining the flanking sequences either side of each AMR hit that were independent (defined as ≥ 50 bp). Using this walkout approach in the NanoOK RT tool, we determined that for infant P205 the vast majority of AMR genes mapped back to *E. cloacae* (87%; Fig. 3h). Contrastingly, although infant P49 had similar levels of *E. cloacae*, only 54% of AMR hits were associated with *E. cloacae* (and a further 15% to its order Enterobacterales), with (low-abundance) *Klebsiella* containing a range of AMR genes, for example, *OXA-2* (β-lactamases) and *patA* (efflux pump), constituting 23% of total AMR genes present (Fig. 3g and Supplementary Table 4). These data highlight that MinION sequencing coupled with the NanoOK Reporter analysis software can potentially map AMR genes to specific bacteria.

**Fig. 4:**
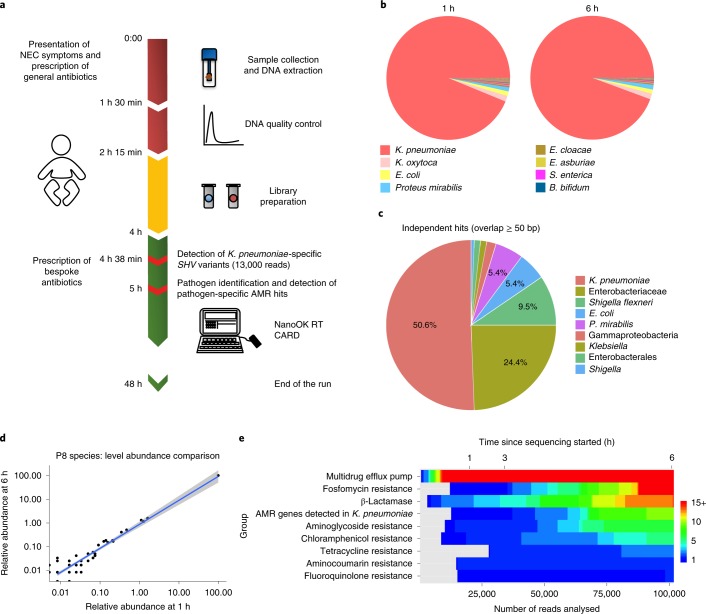
Rapid diagnostic of preterm P8 clinically diagnosed with suspected NEC. **a**, Time frame diagram showing: sample collection, DNA extraction and quality control (red, 2 h 15 min); library preparation incorporating bead clean-up and DNA repair (yellow, 1 h 45 min); data analysis using local base calling and NanoOK RT (green). Pathogen detection (*K. pneumoniae*) and *K. pneumoniae*-specific AMR genes were first detected at 4 h and 38 min (13,000 reads analysed). The left side of the panel indicates the clinical symptoms and general guidelines for antibiotic prescription. **b**, Taxonomic profiles obtained using MinION at 1 and 6 h since sequencing started. The pie chart legend comprises the eight most abundant taxa classified. Further taxa can be found in Supplementary Table 3. **c**, Walkout study of P8 reported by the NanoOK RT software showing taxa containing AMR genes. The results shown are for independent bacterial hits (defined as ≥ 50 bp away from the AMR sequence) at 6 h of sequencing. **d**, Correlation plot of species-level normalized assigned read counts at 1 and 6 h, with log-transformed Pearson’s *r* = 0.97 (taxa *n* = 133). The grey region either side of the fit line represents the 95% CIs. **e**, Heat map displaying the number of CARD hits detected among the most common groups of antibiotic resistance genes found in preterm P8. Further information on all the AMR genes obtained can be found in Supplementary Table 4.

Next we performed a real-time run to evaluate how rapidly MinION plus NanoOK RT could detect potential pathogens and their corresponding AMR profiles in preterm infant P8 (diagnosed with suspected NEC and treated with multiple antibiotics). Current rapid clinical microbiology tests, including determination of antibiotic susceptibility, take between 36 and 48 h. Our real-time run (from sample preparation to analysis) identified pathogens and resistances in approximately 5 h (Supplementary Fig. 2c and Fig. 4a).

**Extended Data Fig. 5:**
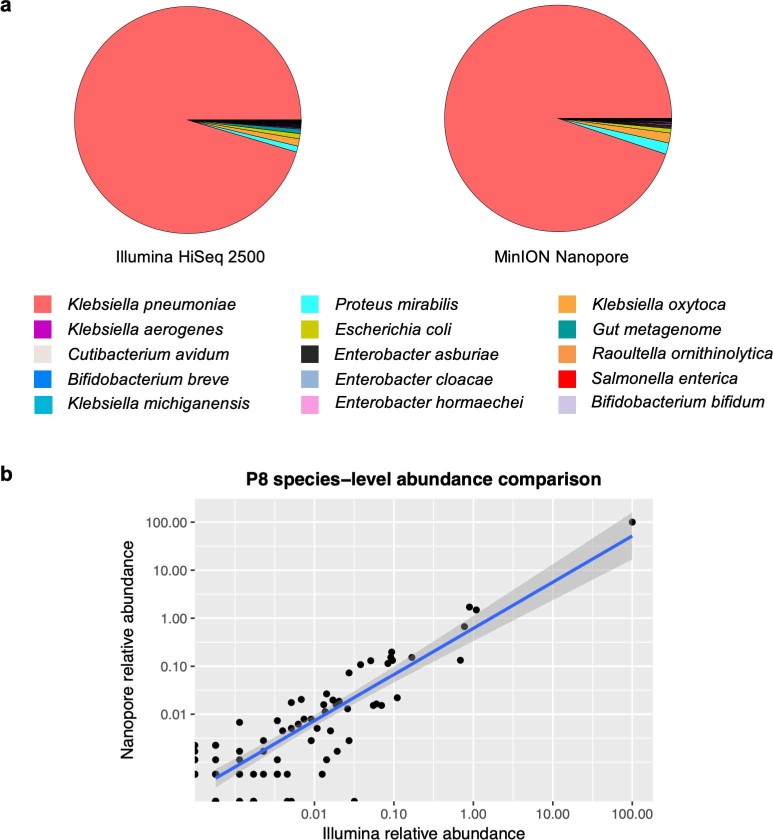
Comparison of taxonomic profiles using Illumina and MinION technology for preterm P8. (**a**) Taxonomic profiles obtained using Illumina HiSeq 2500 and MinION technology for preterm P8. Figure legend comprises the 15 most abundant taxa classified. (**b**) Species-level correlation plot between normalised read count using Illumina (x-axis) and MinION (y-axis) sequencing. Taxa n=121, with log transformed Pearson’s *r* = 0.91. The grey region either side of fit line represents 95% confidence intervals.

Reads were analysed using NanoOK RT, with the first 500 reads indicating a dominance (332 reads) of *K. pneumoniae* (a potential causative organism that has been associated with NEC)^27^. By 1 h after sequencing started (5 h total), the pipeline had analysed 20,000 reads with *K. pneumoniae* accounting for approximately 70% of reads. Further analysis at 6 h showed no significant differences (Fig. 4b,d, log-transformed Pearson’s *r* = 0.97) and was validated by Illumina sequencing (Extended Data Fig. 5a,b)

Our real-time run also indicated that we could rapidly (1 h after sequencing started) map AMR genes/groups (Fig. 4e) including fosfomycin, aminoglycoside and fluoroquinolone resistance, β-lactamases and efflux pumps. We detected *K. pneumoniae*-specific SHV variants^28^ as early as 38 min (at 13,000 reads, 4 h 38 min total time), whereas lower-abundance AMR genes in the sample, for example, those conferring tetracycline resistance, were not detected until 2 h post-sequencing (6 h total).

NanoOK Reporter AMR walkout analysis indicated that the majority of AMR genes within the sample were assigned to *K. pneumoniae* (approximately 51%) or by the lowest common ancestor algorithm (Methods) to within its Enterobacteriaceae family (approximately 24%) (Fig. 4c), including efflux pumps *oqxB* (group *oqx*-*mex*-*amr1*), *mdtC* (conferring multidrug resistance, group *mdt*-*mex*-*sme1*), *patA* (resistance to fluoroquinolone) and *FosA5* (resistance to fosfomycin) (Supplementary Table 4).

### Whole-genome sequencing (WGS) analysis and phenotypic assays indicate the robustness of NanoOK RT walkout analysis

**Extended Data Fig. 6:**
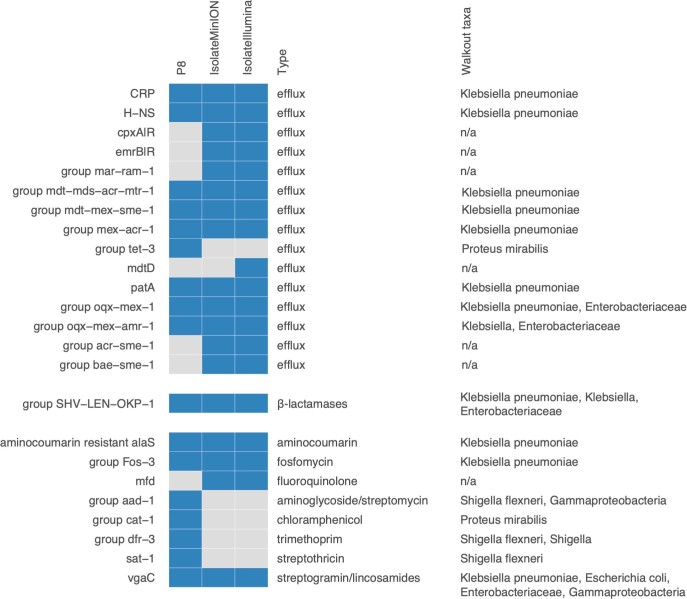
AMR genes associated to *Klebsiella pneumoniae* from a metagenomic sample P8 compared to those found in P8 *Klebsiella pneumoniae* isolate. Heat maps displaying AMR genes associated to *K. pneumoniae* from faecal sample P8 and AMR genes detected from a *K. pneumoniae* isolate from the same sample. Sequencing of the metagenomic sample was performed for only 6h using MinION and NanoOK RT tool (highlighted as ‘P8’’). Sequencing and assembly of the *K. pneumoniae* isolate was performed using both MinION (‘Isolate MinION’) and Illumina HiSeq (‘Isolate Illumina’’). AMR genes were grouped according to sequence similarity. Blue indicates presence, grey indicates absence. The ”Walkout taxa” column shows the taxa that NanoOK RT’s walkout function classified the nanopore AMR hits to. An “n/a” indicates the gene is not found in the metagenomic nanopore sample. A single read can only be classified to a single taxa, but multiple taxa are shown if different reads classify to different taxa. Further details of specific gene names can be found in Supplementary Table 8.

To validate the genotypes obtained from our real-time MinION run, 8 *K. pneumoniae* isolates from P8 were obtained (the 16S ribosomal RNA gene sequence alignment indicated similarity levels ranging from 99.8 to 100%; Supplementary Table 5), with whole-genome shotgun sequencing and assembly on one *K. pneumoniae* isolate performed using Illumina and Nanopore technologies. The longer Nanopore reads produced a single contig of 5.47 Mb and two further contigs of 0.37 Mb, while Illumina produced 69 contigs totalling 5.73 Mb. Many of the AMR genes/groups detected in the walkout analysis from the metagenomic sample P8 correlated with both the Illumina and MinION isolate data (Extended Data Fig. 6). A significant proportion (approximately 60%) of the resistance genes/groups in the metagenomics walkout and the WGS isolate correlated with the efflux pumps (for example, groups *mdt*-*mds*-*acr*-*mtr*, *mdt*-*mex*-*sme*, *mex*-*acr* and *oqx*-*mex*-*amr*), while other hits correlated to known *K. pneumoniae* AMR genes/groups including β-lactamases (for example, the *SHV*-*LEN*-*OKP* group) or fosfomycin resistance (group *Fos3*).

To confirm these genomic AMR profiles, we carried out antibiotic phenotyping on three preterm bacterial isolates: two pathogenic (P8 *K. pneumoniae* and P49 *E. cloacae*); and one beneficial (P103 *B. bifidum*). The P8 *K. pneumoniae* isolate was tested against the seven most commonly used antibiotics in neonatal intensive care units (Supplementary Table 10), with the isolate found to have higher minimum inhibitory concentration (MIC) breakpoint values than those put forward by the European Committee on Antimicrobial Susceptibility Testing^29^for previously prescribed antibiotics, that is, benzylpenicillin, amoxicillin and gentamicin. In contrast, the only MIC breakpoint value lower than the European Committee on Antimicrobial Susceptibility Testing was for cefotaxime, an antibiotic not prescribed. These data correlate with the AMR data generated by the NanoOK Reporter and walkout analysis (Extended Data Fig. 6). Phenotypic testing for P49 *E. cloacae* indicated resistance to gentamicin and benzylpenicillin (Supplementary Table 10), correlating with prescribed antibiotics (Supplementary Fig. 2a), and the AMR genes detected by our ‘walkout’ analysis: the *ACT* (resistance to benzylpenicillin) and *acrB* genes (resistance to gentamicin) (Supplementary Table 4). P103 *B. bifidum* showed resistance towards mupirocin (Supplementary Table 10), in agreement with the detection of the *ileS* gene (Supplementary Table 4) from the walkout analysis.

### Further enhancements to the Nanopore sequencing technology

**Extended Data Fig. 7:**
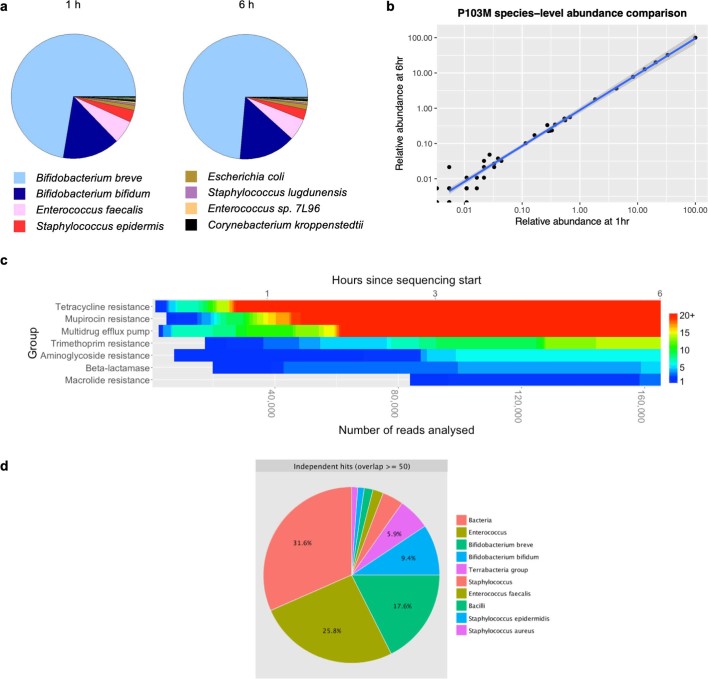
Rapid diagnostic of healthy preterm P103 using SQK-RAD004. **(a)** Taxonomic profiles detected for preterm P103. Figure legend comprises the 8 most abundant taxa obtained. Further information on specific taxa read counts can be found in Supplementary Table 3. (b) Correlation plot of normalised assigned reads from P103 at 1h of sequencing (x-axis) and at 6h of sequencing (y-axis). Taxa n=131, with log transformed Pearson’s *r* = 0.99. The grey region either side of fit line represents 95% confidence intervals. (c) Heat map displaying number of CARD database hits detected among the most common groups of antibiotic resistance genes found in preterm P103. Top and lower panel indicate the hours since sequencing started and the number of reads analyzed within this timeframe. Further information on the specific AMR genes classified can be found in Supplementary Table 4. **(d)** Walkout results reported by NanoOK RT software. Results shown are from independent hits (bacterial host hit ⋝ 50 bp from the AMR hit) at 6 hours of sequencing.

ONT now produce a rapid library kit that requires as little as 10 min preparation time. Profiling the gut microbiota of healthy infant P103 produced 1.2 million reads (read N50 of 1,957 bp), with a sample-to-analysis time around 60 min faster than our one-dimensional (1D) real-time run on infant P8. We confirmed dominance of commensal *Bifidobacterium* species, including *B. bifidum* (also probiotic species; Extended Data Fig. 7a,b), with NanoOK RT AMR profiling indicating a high proportion of mupirocin and tetracycline resistance (Extended Data Fig. 7c).

We performed a reference-guided assembly of the *B. bifidum* genome, which resulted in 3 contigs with an average identity of 98.86% (Supplementary Fig. 5a,b). A de novo assembly generated 24 contigs mapping to 1.7 Mb of the 2.2 Mb reference with an average identity of 98.64%, demonstrating the potential to resolve whole microbial genomes from metagenomic samples, although the error rate is currently high making SNP analysis, and therefore strain level profiling, challenging.

**Extended Data Fig. 8:**
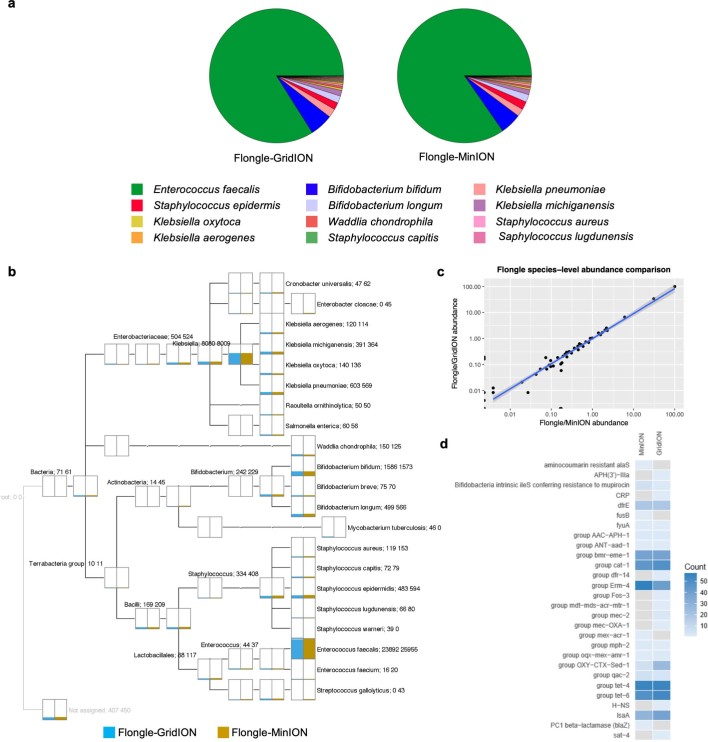
Use of flongle flowcells to profile preterm infant P129 suffering from NEC. (**a**) Taxonomic profiles obtained using flongle flowcell on Gridion and MinION for preterm P129. Figure legend comprises the 12 most abundant taxa. (**b**) MEGAN taxonomy tree representing species assigned for GridION (blue) and MinION (brown) flongle flowcells. The height of the bars corresponds to the number of reads assigned, with normalised counts given next to the species name. (**c**) Species-level correlation plot between normalised read counts from GridION and MinION. Taxa n=60, with log transformed Pearson’s *r* = 0.92. The grey region either side of fit line represents 95% confidence intervals. (**d**) Heat map indicating AMR genes detected for preterm P129 using NanoOK RT tool and Flongle flowcells run on a GridION and MinION.

The Flongle flow cell adaptor (ONT) is another recent enhancement that facilitates the use of cheaper (approximately US$90) flow cells. Using Flongle flow cells on the MinION and GridION, we evaluated P129 (Supplementary Fig. 2d) and confirmed a dominance of potentially pathogenic *Enterococcus faecalis* (Extended Data Fig. 8a,b), as well as a diverse resistome conferring resistance to this infant’s antibiotic treatment, that is, group *AAC*-*APH* genes (gentamicin resistance) and the *PC1* gene (benzylpenicillin resistance). Taxonomic and AMR profiles obtained for the MinION or GridION Flongle datasets were comparable (log-transformed Pearson’s *r* = 0.92 at the species level; Extended Data Fig. 8c,d).

### Benchmarking and validation of MinION and NanoOK RT using mock resistome samples

**Fig. 5:**
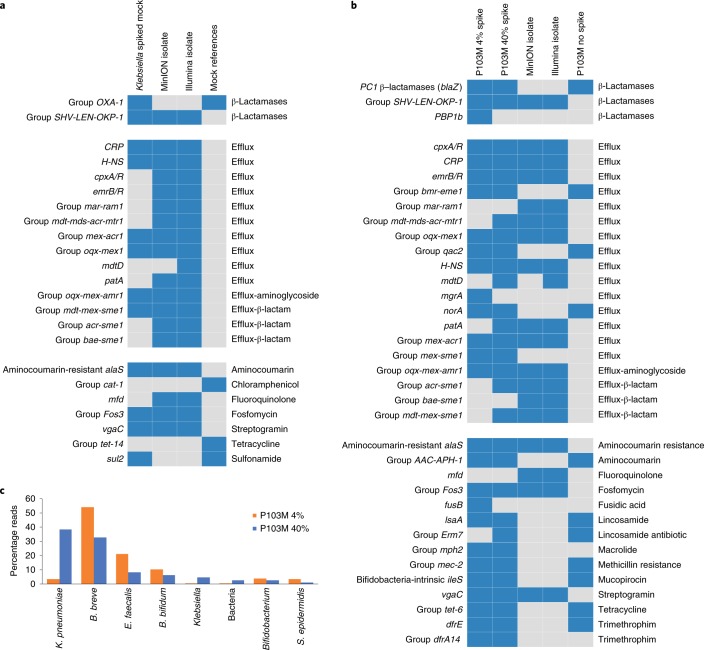
Benchmarking and validation of MinION and NanoOK RT using mock resistome samples. **a**, A known mock community comprising eight bacteria and an isolate of *K. pneumoniae* from P8 was sequenced using MinION and analysed with NanoOK RT. Findings were compared to the results obtained from the P8 *K. pneumoniae* isolate sequenced and assembled by Illumina and MinION. The AMR genes were grouped according to sequence similarity. Blue indicates presence, grey indicates absence. Details of specific genes detected can be found in Supplementary Table 9. **b**, MinION AMR profiles obtained from a faecal sample from a healthy preterm infant (P103M no spike) were compared to the *K. pneumoniae* spiked forms. Two different concentrations of P8 *K. pneumoniae* isolate were inoculated on sample P103M; 4% (P103M 4% spike) and 40% (P103M 40% spike). Findings were compared to the results obtained from the P8 *K. pneumoniae* isolate sequenced and assembled from Illumina and MinION. AMR genes were grouped according to sequence similarity. **c**, Percentages of reads assigned to P103M spiked with 40% P8 *K. pneumoniae* isolate (P103M 40% spike) and 4% P8 *K. pneumoniae* isolate (P103M 4% spike); only taxa representing ≥ 1% are shown.

The data presented so far indicates that we can detect AMR genes using MinION sequencing and bioinformatic tools. However, confirming the robustness and validity of these approaches is important for next-stage clinical studies. Thus, we analysed a mock AMR barcoded seven-strain community, spiking this with the P8 *K. pneumoniae* isolate. Analysis indicated that a significant proportion of AMR genes detected in the spiked mock community corresponded to the WGS isolate data, including group *SHV*-*LEN*-*OKP* (resistant to β-lactam antibiotics) and group *mdt*-*mex*-*sme* (efflux pumps) (Fig. 5a). Some genes in the isolate assemblies were not present in the mock community, probably a consequence of low sequence coverage. By barcoding the mock constituent species, we validated NanoOK RT’s walkout decisions; 97 genes out of 107 were correctly assigned (Supplementary Table 9). Of those incorrectly assigned, five were assigned within the same genus and three appear to be due to barcodes that were wrongly identified by the ONT software (typically due to sequence error), thus independently of the walkout strategy. For the *K. pneumoniae* spike, 34 out of 35 genes were correctly assigned to species or higher taxa, the remaining gene suffering a misassigned barcode. We also spiked a metagenomic DNA sample (healthy preterm infant P103) with two different P8 *K. pneumoniae* sequenced isolate DNA concentrations (4 and 40%) to test sensitivity and specificity (Fig. 5b). The majority (22 out of 31) of AMR genes were detected at both concentrations, although the *mdt*-*mds*-*acr*-*mtr* group, *mdtD*, *patA*, *acr*-*sme* group, *mdt*-*mex*-*sme* group and *ERM-7* group were only detected in the P103M 40% spike mock. Notably, reads assigned to P8 *K. pneumoniae* in P103M 40% were approximately 10× higher than the lower spiked mock, P103M 4% (Fig. 5c).

## Discussion

With worldwide concerns about increasing AMR rates, there is a pressing need for optimized and rapid metagenomic sequencing platforms and bioinformatic tools that could be used to gather clinically relevant data. In this study, we used a combination of improved Nanopore sequencing chemistries and our own open source analysis packages to successfully profile mock and clinical metagenomes. MinION sequencing data were comparable in discriminatory power to Illumina sequencing data, allowing profiling and abundance of microbial species, community resistome profiling and species-specific antibiotic resistance profiles, which were benchmarked using mock communities and phenotypic testing.

Initial mock community profiling confirmed the MinION was a suitable tool (comparable to Illumina) for metagenome profiling^30^, which we extended to preterm gut microbiota profiling, thereby identifying a supplemented probiotic species (that is, *B. bifidum*; Fig. 2c) and *E. cloacae*, a known sepsis pathogen^31^. Furthermore, MinION and Illumina data indicated highly comparable AMR resistome profiles—low numbers of AMR groups within healthy *Bifidobacterium*-dominated preterm infants—whereas a larger AMR gene repertoire was present in the gut microbiota of infants dominated by *Klebsiella* and *Enterobacter*.

With the worldwide AMR threat, metagenomic profiling for resistance genes in a timely and accurate manner could be used in critical care settings. Notably, MinION- and Illumina-generated reads mapped to genes with similar antibiotic resistance mechanisms (Extended Data Fig. 3), including β-lactamase and aminoglycoside genes (conferring resistance to benzylpenicillin and gentamicin, respectively), and only 3 unique resistance mechanisms (*bacA*, *sat4* and the *mph-2* group) of all 70 AMR genes/groups were exclusively detected by Illumina sequencing. This result may be due to the lower MinION read count and might be mitigated by ongoing improvements in MinION technology. Because grouping of genes is based on sequence identity, this approach may not allow differentiation between grouped genes that in fact have different resistance mechanisms despite their sequence similarity. These caveats are important within a clinical context and further studies are required to understand these subtle differences in light of the potential limitations of Nanopore sequencing sensitivity.

Our NanoOK RT software allowed in-depth analysis of species abundance and antibiotic resistance genes in ill infants (P49 and P205). These preterm infants had high levels of *E. cloacae* and a significant resistome (AMR genes including *ACT-27* mapping directly to *E. cloacae*; Supplementary Table 4), which may correlate with the clinical diagnosis of suspected NEC. Our software indicated specific taxa harbouring AMR genes, for example, gene *ACT-27* mapping to *E. cloacae* (Supplementary Table 4). Notably, performing a walkout, rather than de novo metagenomic assembly, requires less computing time and therefore represents a faster method of characterizing potential multidrug-resistant pathogens. However, we also used MinION metagenomic data to assemble *B. bifidum* (P103) using a reference-guided approach and a more challenging de novo assembly, highlighting how more in-depth genomic follow-up studies can be performed from these data.

Next we sought to understand how rapidly we could determine microbial identification and corresponding AMR profiles by mimicking a more clinically relevant diagnostic approach by performing a real-time run using samples from an extremely ill preterm infant (P8) who had received multiple antibiotic courses since birth (46 d antibiotic treatment out of 63 d of life at sample collection). MinION sequencing generated high yields and revealed a *K. pneumoniae*-dominated profile after just 1 h of sequencing, which may link with the clinical NEC diagnosis since intestinal overgrowth of this pathogen can induce pathological inflammatory cascades^32^. Profiling of additional and more complex samples from infants diagnosed with NEC (that is, P49 and P205) indicated distinct and differential microbiota profiles (when compared to P8) also 1 h after the start of sequencing (Fig. 3a,b). Real-time analysis of MinION data using NanoOK RT highlighted the presence of a significant resistome just 10 min after the start of sequencing, including β-lactamases, aminoglycoside resistance genes and multidrug efflux pumps, with greater sequencing depth correlating with higher numbers of AMR genes (Fig. 4e).

*Klebsiella* is of particular AMR concern due to the increasing emergence of multidrug-resistant isolates that cause severe infection and represent a real threat to patient outcomes^33^. Benchmarking with WGS (Illumina and MinION) indicated broad agreement with AMR profiles from the MinION metagenomic run, although we noted a slightly expanded AMR profile at 6 h with the walkout analysis (Extended Data Fig. 5). These differences may correlate with intra-infant strain level variation; thus, single-isolate WGS analysis would not capture the wider AMR repertoire. However, further work is required to determine the utility of strain level analysis, including the development of a standardized framework determining the parameters for single-nucleotide polymorphism analysis, to compensate for the lower read accuracy observed in MinION data, and requiring substantial additional experimental validation. When subjecting strains to MIC testing (the current gold standard for profiling AMR), we observed phenotypic resistance to all main groups of antibiotics that had been prescribed to infant P8, with strong association between AMR gene detection and MIC testing, for example, *SHV* and β-lactam antibiotics, and *oqxB* genes and gentamicin, thereby suggesting that MinION could be useful for rapid AMR profiling.

MIC phenotypic testing on preterm-associated *E. cloacae* and *B. bifidum* isolates agreed with our walkout analysis, with mock community experiments also providing the expected AMR profiles. However, if a potential pathogen is present at low levels within the total microbiome, ability to detect its AMR genes may be reduced. (This is potentially solvable using greater sequencing depth.) From a clinical standpoint, infection is typically associated with pathogen overgrowth; thus, these mock experiments provide strong indications that the MinION and NanoOK RT combination may provide robust antibiotic resistance data. Further (multicentre cohort) clinical studies are required to establish the accuracy of Nanopore/NanoOK methods before they could be considered as clinical diagnostic tools.

Rapid profiling and portability is crucial within clinical and fieldwork settings; however, current standard (large footprint) NGS platforms (Illumina and PacBio) often take > 10–40 h to run (excluding analysis). We obtained MinION bioinformatics results within 1 h of sequencing (5 h total time), with the recent rapid kit being even quicker and the Flongle representing a more cost-effective approach. However, the accuracy of Nanopore reads still lags behind short-read platforms, which necessitate the use of both lower BLAST thresholds and AMR gene groupings. As Nanopore read accuracy continues to reach that of short reads, this will no longer be necessary. The longer length of Nanopore reads results in longer (more significant) alignments, but further optimization and validation, including using standard clinical microbiology testing, is required for refinement and the development of clinical management of patients.

## Conclusion

MinION technology in conjunction with NanoOK RT analysis represents a platform for rapid profiling of gut-associated bacterial species including potential pathogens and corresponding AMR profiles. The accuracy of this approach was confirmed by comparison to Illumina metagenomic sequencing, characterization of patient-derived bacterial isolates, including WGS and phenotypic (that is, MIC) testing, and using mock communities with known AMR profiles. Together these analyses and approaches may prove useful in healthcare settings, particularly with regard to resistome analysis and antibiotic stewardship interventions in the future.

## Methods

### Mock community benchmarking

#### DNA

We used genomic DNA from a microbial mock community used in the Human Microbiome Project (HM-277D; BEI Resources). This mock community contains a mixture of 20 bacterial strains containing staggered RNA operon counts. Details of the strains present in the community are indicated in Supplementary Table 1.

#### Illumina sequencing of mock community

Illumina-compatible, amplification-free, paired-end libraries were constructed with inserts spanning from 600 to > 1,000 base pair (bp). A total of 600 ng of DNA was sheared in a 60 µl volume on a Covaris S2 for 1 cycle of 40 s with a duty cycle of 5%, cycles per burst of 200 and an intensity of 3. Fragmented DNA was then end-repaired using the NEBNext End Repair Module (New England Biolabs), size-selected with a 0.58× Hi Prep bead clean-up (GC Biotech) and followed by A-tailing using the NEBNext dA-Tailing Module (New England Biolabs) and ligation of adaptors using the Blunt/TA Ligase Master Mix (New England Biolabs). Three 1× bead clean-ups were then undertaken to remove all traces of adaptor dimers. Library quality control was performed by running an Agilent Bioanalyzer High Sensitivity Chip and quantified using the KAPA Library Quantification Kit for Illumina Platforms (KAPA Biosystems). Based on the quantitative PCR quantification, libraries were loaded at 9 pM on an Illumina MiSeq System and sequenced with 300 bp paired reads.

#### MinION sequencing of mock community

MinION two-dimensional (2D) libraries were constructed targeting inserts >8 kilo-base pair (kbp). A total of 1 µg of DNA was fragmented in a 46 µl volume in a g-TUBE (Covaris) at 6,000 r.p.m. in an Eppendorf 5417 Centrifuge. Sheared DNA was then subjected to a repair step using the NEBNext FFPE DNA Repair Mix (New England Biolabs) and purified with a 1× Hi Prep bead clean-up (GC Biotech). A DNA control was added to the repaired DNA and then end-repaired and A-tailed using the NEBNext Ultra II End Repair/dA-Tailing Module (New England Biolabs), and purified with a 1× Hi Prep bead clean-up; then the AMX and HPA MinION adaptors were ligated using the Blunt/TA Ligase Master Mix. An HP tether was then added and incubated for 10 min at room temperature followed by a further 10 min room temperature incubation with an equal volume of pre-washed MyOne Streptavidin C1 beads (Thermo Fisher Scientific). The library-bound beads were washed twice with bead binding buffer (ONT) before the final library was eluted via a 10 min incubation at 37 °C in the presence of the MinION elution buffer. The final library was then mixed with running buffer, fuel mix and nuclease-free water and loaded onto an R7.3 flow cell according to the manufacturer’s instructions; sequencing data were collected for 48 h.

### Mock community data analysis

MinION reads were basecalled using the Metrichor service (https://metrichor.com/) and downloaded as FAST5 files. NanoOK v.0.54 (ref. ^18^) was used to extract the FASTA files, align (via the LAST aligner (v.572)^34^) against a reference database of the 20 genomes and generate an analysis report (Supplementary Note 1). Quality control of the Illumina data was carried out with FastQC (https://www.bioinformatics.babraham.ac.uk/projects/fastqc/) to ensure read quality was within the expected bounds. This demonstrated a mean quality control of 30 up to base 250. We subsampled a random set of 1,000,000 reads (subsample.pl script; https://github.com/richardmleggett/scripts) to represent the yield of a MiSeq nano flow cell and ran Trimmomatic (v.0.30)^35^ to remove remaining adaptor content and apply a sliding window quality filter (size 4, mean quality ≥15). Illumina and MinION reads were then BLASTed (BLASTn^36^ v.2.2.29, maximum e-value 10 × 10^−3^) against the National Center for Biotechnology Information (NCBI) nucleotide database and the results were imported into MEGAN6 (ref. ^37^) for taxonomic analysis. In a separate analysis, the reads were mapped against references using minimap2 v2.17-r943 (ref. ^38^) and alignments processed using the bamstats.py script (https://github.com/guigolab/bamstats)^39^. Another script (parse_bamstats.pl; https://github.com/richardmleggett/bambi) then totalled the read mapping to each reference and these counts were imported into Microsoft Excel where they were normalized to relative abundances. Counts were log-transformed and the log-transformed Pearson’s coefficient was computed in Microsoft Excel 2016 using the PEARSON function. Plots were produced in R (v.3.3.2) using the Excel data (plot_correlation.R; https://github.com/richardmleggett/bambi). The grey region either side of fit line represents the 95% confidence intervals (CIs).

### Clinical samples

#### Ethical approval and preterm sample collection

The Ethics Committee of the Faculty of Medical and Health Sciences at the University of East Anglia approved participant recruitment for this study. The protocol for faeces collection was laid out by the Norwich Research Park Biorepository, was in accordance with the terms of the Human Tissue Act 2004 and approved with licence no. 11208 by the Human Tissue Authority. Infants admitted to the Neonatal Intensive Care Unit of the Norfolk and Norwich University Hospital NHS Foundation Trust were recruited by doctors or nurses, with informed and written consent obtained from parents. Oral probiotic supplementation provided to the infants in this study contained *B. bifidum* and *Lactobacillus acidophilus* (Infloran; Desma Healthcare) strains with a daily dose of 1 × 10^9^ of each species. Collection of faecal samples was carried out by researchers and samples were stored at −80 °C before DNA extraction.

#### DNA extraction from faeces samples (preterm infants)

Bacterial DNA was extracted using the FastDNA Spin Kit for Soil (MP Biomedicals) according to the manufacturer’s instructions but extending the bead-beating step to 1 min and eluting the DNA with 55 °C DNA Elution Solution. The starting faecal material used to extract DNA was between 100 and 150 mg. DNA purity and concentration were assessed using a NanoDrop 2000c Spectrophotometer (Thermo Fisher Scientific) and Qubit 2.0 fluorometer (Thermo Fisher Scientific). Samples with DNA concentrations higher than 25 ng µl^−1^ were considered acceptable.

#### MinION shotgun library preparation

MinION 2D libraries were constructed as outlined for the mock community (see earlier) except that, for the R9.4 flow cells, the final library was mixed with running buffer containing fuel mix, library loading beads and nuclease-free water and loaded onto the flow cell according to the manufacturer’s instructions. MinION 1D ligation libraries were constructed using 1 μg unfragmented DNA. This was subjected to a repair step using the NEBNext FFPE DNA Repair Mix (New England Biolabs) and purified with a 1× Hi Prep bead clean-up (GC Biotech). A DNA control was added to the repaired DNA and then end-repaired and A-tailed using the NEBNext Ultra II End Repair/dA-Tailing Module (New England Biolabs), and purified with a 1× Hi Prep bead clean-up. The ONT Adapter Mix MinION adaptors were then ligated using the Blunt/TA Ligase Master Mix (New England Biolabs). Library molecules were then purified with a 0.4× Hi Prep clean-up and washed twice with ABB buffer before the final library was eluted via a 10 min incubation at 37 °C in the presence of the MinION elution buffer. The final library was then mixed with running buffer, fuel mix and nuclease-free water and loaded onto a flow cell according to the manufacturer’s instructions. MinION 1D rapid libraries were prepared by incubating 200 ng of DNA with 2.5 µl FRM buffer for 1 min at 30 °C then 1 min at 75 °C, followed by adding 1 µl Rapid Adapters and incubating at room temperature for 5 min. The final library was then mixed with running buffer, fuel mix and nuclease-free water and loaded onto the flow cell according to the manufacturer’s instructions. Further details on the genomic sequencing kits and samples used in this study can be found in Extended Data Fig. 2.

#### Illumina HiSeq 2500 shotgun library preparation

Libraries for the samples (P10N, P10R and P10V) were prepared using the TruSeq Nano DNA Library Prep Kit (Illumina) according to the manufacturer’s instructions and sequenced with the HiSeq Illumina 2500 System with 150 bp paired-end reads. The library for P8 was prepared as for the amplification-free library for the mock community (see earlier) and run at 9 pM on an Illumina MiSeq System with a 2 × 250 bp read metric.

#### Time series study for infant P10

The Illumina and MinION sequencing data for samples P10N, P10V and P10R from infant P10 were studied. For the Illumina samples, we removed PCR duplicates with the remove_pcr_duplicates.pl script (https://github.com/richardmleggett/scripts), ran Trimmomatic^35^ to remove the adaptors and applied a sliding window quality filter (size 4, mean quality ≥15) and then randomly subsampled 1 million reads (subsample.pl script; https://github.com/richardmleggett/scripts). These reads were used as the input to a BLASTn search (maximum e-value 10 × 10^−3^) of the NCBI’s nucleotide database. For the Nanopore sequencing, we took only the reads classified as pass reads (defined as 2D reads with a mean *Q* > 9) and performed no further preprocessing before running BLASTn^36^. Using MEGAN6, we removed reads matching *Homo sapiens* (accounting for <0.1% per sample) and performed taxonomic analysis. Rarefaction plots (Supplementary Fig. 3) were also plotted in MEGAN6.

#### PCoA analysis of clinical samples

All pass reads were BLASTn-searched against the NCBI nucleotide database (maximum e-value 10 × 10^−3^) and results imported into MEGAN6. Samples were compared on normalized read counts using MEGAN’s Compare option. Taxa were selected at the species level and the MEGAN’s Cluster analysis function was used to produce a PCoA plot using a Bray–Curtis distance measurement.

#### Real-time diagnostic study using MinION and NanoOK RT

One sample from infant P8 was sequenced with Nanopore libraries prepared using the SQK-LSK108 Ligation Sequencing Kit 1D (Oxford Nanopore Technologies) and SQK-RAD002 Rapid Sequencing Kit 1D (Oxford Nanopore Technologies). We could not obtain good-quality data from the infant stool samples with the SQK-RAD002 kit, but were subsequently successful when a newer version of the kit (SQK-RAD004) was released. We evaluated this version using a sample from healthy infant P103.

The SQK-LSK108 Ligation Sequencing Kit 1D was used for samples P49A, P250G, P106I and P116I. The SQK-RAD004 Rapid Sequencing Kit 1D was used for sample P103M. The SQK-LSK109 Ligation Sequencing Kit 1D was used for sample P129B, having superseded SQK-LSK108 by the time of the Flongle experiment. Libraries were sequenced on a mixture of R9.4, R9.5 and R9.4.1 flow cells, as shown in Extended Data Fig. 2.

For sample P129B, 1 Flongle flow cell was sequenced on a MinION Mk1B and 1 on a GridION X5. The MinKNOW software was used to collect signal data. In the first hour, the flow cells generated 41,700 (GridION) and 60,400 (MinION) reads, but a higher proportion of reads was classified as ‘fail’ than with the latest full-size flow cell (Extended Data Fig. 2). Real-time analysis was carried out for the GridION run only; 1 h after sequencing started, NanoOK RT had processed 25,000 ‘pass’ reads.

ONT’s subsidiary Metrichor provide a cloud-based classification tool called ‘What’s In My Pot?’. We initially tried using this for an earlier 2D sequencing run of P8 (data not shown), but found that analysis lagged behind sequencing. The lack of user control over the database and classification tool was also restrictive for our purposes. This necessitated switching to local basecalling through MinKNOW and developing our own analysis pipeline.

To enable the real-time analysis of MinION data, functionality was added to NanoOK^18^. The software, NanoOK RT, monitors a specified directory for basecalled sequence files as they are created by MinKNOW. For efficiency, files are grouped into batches of 500 and each batch was BLASTn-searched against the NCBI nucleotide database (downloaded in April 2017) and the Comprehensive Antibiotic Resistance Database (CARD) (v.1.1.1, downloaded in October 2016) of antibiotic resistance genes^40^. NanoOK RT also writes out command files for MEGAN, which allows more detailed analysis of community composition, either as the run proceeds or on completion. NanoOK RT is available as an extension to NanoOK, selectable as a runtime option, from https://github.com/richardmleggett/NanoOK.

Another bioinformatics tool, NanoOK Reporter, was developed for this project and provides a graphical user interface to monitor the run and view summaries of community composition and any antibiotic resistance genes identified. NanoOK Reporter uses a lowest common ancestor algorithm to assign reads to the lowest possible taxonomy level consistent with all good BLAST matches. Adopting the approach taken by MEGAN, we considered any hits with a bit score of at least 90% of the highest scoring hit. Results are displayed on a taxonomy tree, donut plot or as a summary table showing the most abundant matches. The tool allows the user to browse through data in real time as batches are processed or after all of the results are in, using their timestamps to indicate when a result is first obtained. Summary data can also be exported as plain text files; these were subsequently used for later analysis. The lowest common ancestor algorithm is only appropriate for species assignment, since for AMR hits it is perfectly possible that multiple hits can occur along the length of a single long read. In NanoOK RT, these are accepted if they meet the quality criteria (configurable; we used an e-value <0.001, length >200 bp, identity >80%; see Choice of AMR match criteria section) and do not overlap other AMR hits by more than 10% of their length to avoid multiple hits to the same stretch of sequence. Walkout analysis can be initiated by clicking on an icon and produces a pie chart showing the taxa containing antibiotic resistance genes, as well as generating a text file giving per-read analysis. The walkout analysis proceeds by examining each read that has a good-quality hit to an AMR gene to see if it also has an independent hit to the nucleotide (or bacterial alias) database. In our experiments, we defined independence as a match that stretched at least 50 bases away from the AMR gene in either direction. As with taxonomic assignment, NanoOK Reporter implements a lowest common ancestor algorithm to assign species to the flanking sequence. For walkout analysis, we used the first 6 h of analysed data, which equated to the following number of pass reads: 101,500 (P8); 48,000 (P49A); 165,000 (P103M); and 478,000 (P205G). NanoOK Reporter is available from https://github.com/richardmleggett/NanoOKReporter. The documentation for NanoOK Reporter, as well as a tutorial using the data from this publication, are available at https://nanook.readthedocs.io/en/latest/reporter.html.

#### Choice of AMR match criteria

In accordance with previously published work classifying AMR genes from Nanopore sequence data^16,41–43^, we adopted a minimum BLAST sequence identity requirement of 80%, which takes into account the higher error rate of Nanopore sequencing compared to short-read technologies. We further validated this choice by sequencing a microbial mock community with a known AMR profile and investigating the effect of varying the minimum identity on the precision of AMR gene identification.

MinION libraries were constructed using 500 ng of the ZymoBIOMICS Microbial Community DNA Standard (Cambridge Bioscience) without fragmentation and according to the ONT SQK-LSK109 kit instructions. The final library was loaded onto a FLO-MIN106D Flow Cell (R9.4.1) according to the manufacturer’s instructions; sequencing data were collected for 48 h.

The true set of AMR genes for the mock community was determined by BLASTing the reference genomes against CARD. Because the genomes are finished references with high contiguity and accuracy, we set the BLAST criteria to a maximum e-value of 0.001, minimum length of 200 bp and minimum identity of 95%. The first 100,000 sequenced Nanopore reads were then BLASTed against the same CARD with maximum e-value of 0.001 and minimum length of 200 bp. Supplementary Fig. 6 shows the effect of changing the sequence identity on the true positive, false positive and false negative rates. At 80%, we recorded the highest true positive (117) and lowest false negative (6) rates, while the false positive rate remained low (7). The lowest false negative rate (0) occurs at 100% identity, but this coincides with the lowest true positive (0) and highest false negative (123) rates.

#### AMR gene grouping

Because of the higher error rate of Nanopore sequencing compared to the Illumina platforms, we were not confident that genes with low coverage and high sequence similarity could be differentiated unambiguously. To address this, we wrote a Python script, AMR_gene_grouper.py, which groups similar genes in the CARD according to sequence similarity. This script can be found at https://github.com/SR-Martin/CARD_Tools and can be rerun for future versions of CARD. Input to the script is a BLAST alignment of all genes against all genes. Match parameters are configurable, but in this study genes were grouped if they had at least 70% sequence identity. Group names are generated automatically based on the constituent genes. A full list of the gene groupings used can be found in Supplementary Table 6.

#### Generation of AMR gene presence/absence comparisons

We created a bespoke Java program (amranalyser; https://github.com/richardmleggett/amranalyser) to parse the CARD BLAST results for each sample and output tab-separated files of genes/groups with presence/absence indicators. This program accepts hits if they fall below the maximum e-value (0.001), are greater than a minimum length (200 bp) and meet a minimum sequence identity (80%). Additionally, hits must overlap by no more than 10% of their length with previously accepted hits to facilitate parsing of long reads or contigs that contain multiple AMR loci. A separate R script, plot_amr_heatmaps.R, reads the tab-separated files and generates plots. The number of reads contributing to the plots varied slightly according to the experimental yield: for sample P10, we used all pass reads (83,000 reads for P10N, 48,000 for P10R and 53,000 for P10V); for the barcoded mock community, we used all 90,000 subsampled pass reads; for the P103 spike, P8 and Flongle flow cells, we used the first 100,000 pass reads. The genes/groups for the isolate assemblies are provided in Supplementary Table 8.

#### Generation of AMR gene heat maps

We opened the CARD results using NanoOK Reporter and used the option to save summary data as a plain text file. This saves a text file at each time point (in this study, batches of 500 timestamped reads) summarizing the counts of resistance genes identified up to that point (files available at https://github.com/richardmleggett/bambi in the folder ‘nanook_reporter_files’). CARD hits were only considered for this analysis if they possessed an e-value <0.001, a sequence identity ≥80% and a length ≥200 bp. We took the latest time point file that the heat map was to show (for example, 6 h) and extracted a list of the antibiotic resistance ontology numbers from the ID column. Each unique antibiotic resistance ontology number was manually assigned to its corresponding antibiotic resistance group according to the classification given by CARD. We wrote a script (gather_heatmap_data.pl; available at https://github.com/richardmleggett/bambi) to take the summary files, together with this mapping, and generate a final file summarizing hits per group at each time point. An R script (plot_card_heatmap.R; https://github.com/richardmleggett/bambi) takes this file and produces the heat map.

#### Statistical analysis

Read counts at different stages of the bioinformatics analysis are provided in Extended Data Fig. 2. For comparative analysis, MEGAN6 was set to subsample reads down to the read count of the sample with the lowest number of reads. For Pearson’s correlation comparisons of taxonomic profiles (for example, at 1 and 6 h), the two samples to be compared were loaded into MEGAN6; its comparison function was used to display both on the same tree. MEGAN6 was set to display genus (Fig. 1) or species level (remaining figures); all nodes were selected and the assigned read counts were exported to a single CSV file. The CSV file was imported into Microsoft Excel, relative abundances were calculated and log-transformed, and the PEARSON function was used to calculate Pearson’s *r* from the log-transformed data. The Microsoft Excel data were exported to a tab-separated file and plots were produced using R (plot_correlation.R; https://github.com/richardmleggett/bambi).

#### Isolation and biochemical characterization of P8 *K. pneumoniae* strains

An aliquot (100 mg) of faecal sample was homogenized in 1 ml TBT buffer (100 mM Tris/HCl, pH 8.0, 100 mM NaCl, 10 mM MgCl_2_) by pipetting and plate-mixing at 1,500 r.p.m. for 1 h. Homogenates were serially diluted to 10^−^^4^ in TBT buffer. Aliquots of 50 µl were spread on MacConkey agar plates (Oxoid) in triplicate and incubated aerobically at 37 °C overnight.

Colonies were selectively screened for lactose-positive (that is, pink) colonies. One colony of each morphology type was restreaked on MacConkey agar three times to purify. Biochemical characterization was performed using API 20E tests (Biomerieux) according to the manufacturer’s instructions.

#### 16S rRNA phylogenetic analysis of P8 *K. pneumoniae* isolates

Sequences of the 16S rRNA gene from nine *K. pneumoniae* isolates were prepared to perform the phylogenetic analysis. We extracted DNA using the FastDNA Spin Kit for Soil according to the manufacturer’s instructions and then amplified the 16S rRNA gene with the Verit 96-Well Thermal Cycler (Applied Biosystems), master mix from Kapa2G Robust PCR reagents (KAPA Biosystems) and the following primers: fD1 (forward, 5′-AGA GTT TGA TCC TGG CTC AG-3′); fD2 (forward, 5′-AGA GTT TGA TCA TGG CTC AG-3′); and rP1 (reverse, 5′-ACG GTT ACC TTG TTA CGA CTT- 3′) (ref. ^44^). PCR amplification conditions were: 1 cycle at 94 °C for 5 min, followed by 35 cycles at 94 °C for 1 min, 43 °C for 1 min and 72 °C for 2 min followed by a final strand extension at 72 °C for 7 min. Amplicons were sequenced using an automated Sanger sequencing service (Eurofins Genomics).

Partial 16S rRNA sequences (approximately 900 positions) of 9 isolates of *K. pneumoniae*, obtained using the automated Sanger sequencing service, were compared for similarity. Multiple sequence alignments were performed with the SILVA Incremental Aligner (v.1.2.11)^45^ and manually curated for quality. Nucleotides were coloured using BoxShade v.3.21 (http://www.ch.embnet.org/software/BOX_form.html). The similarity/identity matrix between sequences was calculated using MatGAT v.2.01 (Matrix Global Alignment Tool) using the BLOSUM 50 alignment matrix^46^.

#### Determination of MIC for P8 *K. pneumoniae*, P49 *E. cloacae* and P103 *B. bifidum*

Calculation of the antibiotic MIC was performed using the broth microdilution method^47^. Serial twofold dilution antibiotics (benzylpenicillin, gentamicin, vancomycin, metronidazole, meropenem, cefotaxime and mupirocin) were added to sterile nutrient broth. The bacterial inoculum of the isolate was prepared using 10 μl from a fresh overnight culture and tests were done in triplicate. Microplates were incubated for 24 h at 37 °C under aerobic conditions. Cell density was monitored using a plate reader (BMG Labtech) at 595 nm. MICs were determined as the lowest concentration of antibiotic inhibiting any bacterial growth.

#### DNA extraction from P8 *K. pneumoniae* isolate for WGS analysis

An overnight (10 ml) culture of the isolate was centrifuged at 4,000 r.p.m. for 10 min, resuspended in 30 ml of PBS (Sigma-Aldrich) and centrifuged again. The pellet was then resuspended in 2 ml of 25% sucrose (Thermo Fisher Scientific) in Tris-EDTA buffer (10 mM Tris (Thermo Fisher Scientific) and 1 mM EDTA at pH 8.0 (VWR Chemicals)); 50 µl of Lysozyme (Roche Molecular Systems) at 100 mg ml^−1^ in 0.25 M Tris, pH 8.0, was added. The mixture was incubated at 37 °C for 1 h; 100 µl of Proteinase K at 20 mg ml^−1^ (Roche Molecular Systems), 30 µl of RNase A at 10 mg ml^−1^ (Roche Molecular Systems), 400 µl of 0.5 M EDTA, pH 8.0, and 250 µl of freshly prepared 10% Sarkosyl NL30 (Sigma-Aldrich) were added. The mixture was then incubated on ice for 2 h and subsequently transferred to a water bath at 50 °C overnight. Next, E Buffer (10 mM Tris, pH 8.0) was added to the sample to a final volume of 5 ml, mixed with 5 ml phenol:chloroform:isoamyl alcohol (25:24:1) (Sigma-Aldrich) in a MaXtract High Density Ttube (QIAGEN) and centrifuged for 15 min at 4,000 r.p.m. The aqueous phase was transferred into a new MaXtract High Density Tube, made up with E Buffer to the volume of 5 ml if necessary, mixed with 5 ml of phenol:chloroform:isoamyl alcohol and centrifuged for 10 min at 4,000 r.p.m. This procedure was repeated with a 5 min centrifugation time. Next, the aqueous phase was transferred into a MaXtract High Density Tube made up to 5 ml with E Buffer as necessary, mixed with 5 ml of chloroform:isoamyl alcohol (24:1) (Sigma-Aldrich) and centrifuged for 5 min at 4,000 r.p.m. The chloroform:isoamyl alcohol step was repeated once more, after which the final aqueous phase was transferred into a sterile Corning 50 ml centrifuge tube and 2.5 volumes of ethanol (VWR Chemicals) were added. The sample was incubated for 15 min at −20 °C, then centrifuged for 10 min at 4,000 r.p.m. and 4 °C. Finally, the DNA pellet was washed with 10 ml of 70% ethanol and centrifuged at 4,000 r.p.m. for 10 min twice, dried overnight and resuspended in 300 µl of E buffer.

#### WGS library preparation and sequencing of P8 *K. pneumoniae* isolate

DNA samples containing 500 ng genomic DNA were analysed. DNA was sheared into fragments of 400–600 bp using a Covaris plate with glass wells and Adaptive Focused Acoustics fibres. Solid-phase reversible immobilization clean-up was used to remove smaller-sized fragments and concentrate the sheared DNA samples. Whole-genome library construction performed by a liquid handling robot comprised end repair, A-tailing and adaptor ligation reactions. Adaptor-ligated samples were subsequently amplified using the following PCR conditions: 5 min at 95 °C; 10 cycles of 30 s at 98 °C, 30 s at 65 °C and 1 min at 72 °C; and 10 min at 72 °C. LabChip GX (Perkin Elmer) was then used to size and assess the quality of the libraries and determine the pooling volumes for each library using Biomek NX^P^ (Span-8; Beckman Coulter Life Sciences). Libraries were prepared using the Sure Select Custom Library Prep kit (Agilent Technologies). Final pools were loaded on the HiSeq 2500 sequencers. For MinION sequencing, a total of 1.5 µg of genomic DNA in a 46 µl volume was fragmented with a g-TUBE at 6,000 r.p.m. in an Eppendorf 5417 centrifuge. A Nanopore library was prepared using the SQK-LSK108 Ligation Sequencing Kit according to the manufacturer’s protocol with the optional FFPE DNA repair step. The library was mixed with running buffer and loading beads, loaded onto an R9.4 flow cell and sequenced for 48 h.

#### Assembly of WGS isolate (P8 *K. pneumoniae*)

Presence or absence of AMR genes was performed on one *K. pneumoniae* isolate from sample P8, benchmarking two different sequencing platforms: MinION and Illumina HiSeq 2500. Sequencing data from the MinION run was assembled using Canu v.1.5 (ref. ^48^) corrected with Racon v.1.3.1 (ref. ^49^) and polished with nanopolish v.0.9.0 (ref. ^50^). Sequencing data from the Illumina HiSeq 2500 run was assembled using Velvet (v.1.1)^51^. Gene presence/absence diagrams were generated as described earlier.

#### *B. bifidum* assembly

For the reference-guided assembly, the P103M MinION pass reads were aligned against an Illumina assembly of the same strain using minimap2. All mapping reads with an alignment quality of 50 or greater were used as the input to the assembly. Reads were processed with Porechop (v.0.2.1) to remove adaptors, before assembly with Canu and polishing with nanopolish. The output contigs from this step were used as the input to minimus2 (ref. ^52^), resulting in three final contigs. Accuracy of assembly was assessed using dnadiff, which is part of MUMmer (v.3.23)^53^, and with the BLAST Ring Image Generator^54^. For the de novo assembly, all metagenomic shotgun MiniKNOW pass reads were processed with Porechop and assembled with Flye v.2.4 (ref. ^55^). Contigs were mapped against the *B. bifidum* PRL2010 reference sequence from the NCBI to identify contigs; the sequence identity of these contigs was assessed using dnadiff.

### Preparation of in vitro mock resistome and clinical mock

#### DNA

We extracted DNA from 2 ml overnight cultures of 7 National Collection of Type Cultures bacterial samples and P8 *K. pneumoniae* isolate. The DNA extraction protocol followed the manufacturer’s instructions (MagAttract HMW DNA Kit; QIAGEN). Details of the strains present in this community are shown in Supplementary Table 7.

#### MinION sequencing of mock resistome

Nanopore 1D native barcoded libraries were constructed targeting inserts >8 kbp using the ONT SQK-LSK109 and EXP-NBD104 kits based on the DNA concentration of the native barcode adaptor-ligated molecules. The mock resistome consisted of 12.5% of each of the 8 strains (for example, an even mock).

A total of 1 µg of each DNA was fragmented in a 46 µl volume in a g-TUBE at 6,000 r.p.m. in an Eppendorf 5417 centrifuge. Sheared DNA was then subjected to a combined repair and A-tailing step using the FFPE DNA Repair Mix and NEBNext Ultra II End Repair/dA-Tailing Module and purified with a 1× KAPA bead (Roche Sequencing) clean-up. Repaired and A-tailed DNA had native barcode adaptors ligated using the Blunt/TA Ligase Master Mix followed by a further purification step with a 1× KAPA bead clean-up. To create an even abundance mock, 87.5 ng of each native barcode adaptor-ligated molecules were pooled. AMXII (ONT) adaptors were ligated to the two pooled mock samples using the Quick T4 DNA ligase (New England Biolabs). Libraries were purified using 0.4× KAPA beads, washed twice with ONT’s long fragment buffer and then eluted in MinION elution buffer by incubating for 10 min at room temperature. The final library was mixed with the sequencing buffer and loading beads, and then loaded onto a FLO-MIN106D Flow Cell (R9.4.1) flow cell according to the manufacturer’s instructions; sequencing data were collected for 48 h.

#### Analysis of barcoded mock data

Barcoded reads from the mock data were basecalled with ONT’s Guppy v.2.3.1. From the pass reads, we took all reads ≤3,000 bp in length (to reflect the reduced read lengths probably found in real samples) and randomly sampled approximately 11,000 reads from each of the barcodes to make a single FASTA file, which was used as the input for the NanoOK RT analysis. Walkout analysis was performed by clicking on the ‘Walk’ icon in NanoOK Reporter; the resultant walkout_results.txt file was processed with a custom Perl script (parse_walkout_barcodes.pl; https://github.com/richardmleggett/bambi), which looks up each read ID in the walkout to discover which barcode is associated with it (Supplementary Table 9). Annotated assemblies of the mock constituents are available from the Public Health England reference collections at the Wellcome Sanger Institute (https://www.sanger.ac.uk/resources/downloads/bacteria/nctc/). We BLASTed these and the P8 *K. pneumoniae* isolate assembly against the CARD (as used previously), filtering for a maximum e-value of 0.001, minimum identity of 80% and minimum length of 200 bp, to determine the expected AMR profile. The amranalyser Java program described earlier was then used to create the presence/absence maps, comparing the profiles of the metagenomic sample with the genome assemblies.

#### MinION sequencing of clinical mock data

We spiked a sample from healthy infant P103 with the DNA from the P8 *K. pneumoniae* isolate. DNA from P103 was run on a TapeStation 2100 (Agilent Technologies) to determine average molecule length. DNA from the P8 *K. pneumoniae* isolate was then fragmented to a similar length using a g-TUBE. The DNA from P8 *K. pneumoniae* was spiked into the P103 sample targeting 10 and 50% of total DNA, based on concentration, and MinION 1D libraries constructed using the ONT SQK-LSK109 Kit. Libraries were constructed as outlined in the manufacturer’s protocol, loaded onto a FLO-MIN106D Flow Cell (R9.4.1) flow cells and sequence data were collected for 48 h. Reads were basecalled with ONT’s Guppy and analysed after sequencing with NanoOK RT.

#### Analysis of Flongle flow cells

For the GridION run, reads were basecalled live, then passed to NanoOK RT for analysis, as per the real-time diagnostic study (see earlier). For the MinION run, reads were basecalled post-sequencing with Guppy, then passed to NanoOK RT for analysis.

### Reporting Summary

Further information on research design is available in the Nature Research Reporting Summary linked to this article.

## Supplementary information

Supplementary Information Supplementary Figs. 1–6 and Supplementary Table 5.

Reporting Summary

Supplementary Tables Supplementary Tables 1–4 and 6–10.

Supplementary Data NanoOK report for mock community (run N79596 MOCK SQKMAP006 24082015).

## Data Availability

Sequence data (Illumina and MinION) that support the findings of this study have been deposited with the European Nucleotide Archive (http://www.ebi.ac.uk/ena) under accession no. PRJEB22207.

## Code availability

NanoOK RT is available as an extension to NanoOK, selectable as a runtime option, from https://github.com/richardmleggett/NanoOK. NanoOKReporter is available from https://github.com/richardmleggett/NanoOKReporter.

## References

[1] Blander JM, Longman RS, Iliev ID, Sonnenberg GF, Artis D (2017). Regulation of inflammation by microbiota interactions with the host. Nat. Immunol..

[2] Lewis BB, Pamer EG (2017). Microbiota-based therapies for *Clostridium difficile* and antibiotic-resistant enteric infections. Annu. Rev. Microbiol..

[3] Pedersen HK (2016). Human gut microbes impact host serum metabolome and insulin sensitivity. Nature.

[4] Lane ER, Zisman TL, Suskind DL (2017). The microbiota in inflammatory bowel disease: current and therapeutic insights. J. Inflamm. Res..

[5] Lippert K (2017). Gut microbiota dysbiosis associated with glucose metabolism disorders and the metabolic syndrome in older adults. Benef. Microbes.

[6] Wekerle H (2017). Brain autoimmunity and intestinal microbiota: 100 trillion game changers. Trends Immunol..

[7] Khoruts A, Sadowsky MJ (2016). Understanding the mechanisms of faecal microbiota transplantation. Nat. Rev. Gastroenterol. Hepatol..

[8] Mayor S (2018). First WHO antimicrobial surveillance data reveal high levels of resistance globally.. BMJ.

[9] Hoffman SJ (2015). An international legal framework to address antimicrobial resistance.. Bull. World Health Organ..

[10] *Tackling Drug-Resistant Infections Globally: Final Report and Recommendations. The Review on Antimicrobial Resistance* (Wellcome Trust and HM Government, 2016).

[11] Boolchandani M, D’Souza AW, Dantas G (2019). Sequencing-based methods and resources to study antimicrobial resistance.. Nat. Rev. Genet..

[12] Leggett RM, Clark MD (2017). A world of opportunities with nanopore sequencing. J. Exp. Bot..

[13] Greninger AL (2018). The challenge of diagnostic metagenomics. Expert Rev. Mol. Diagn..

[14] Quick J (2016). Real-time, portable genome sequencing for Ebola surveillance. Nature.

[15] Ashton PM (2015). MinION nanopore sequencing identifies the position and structure of a bacterial antibiotic resistance island. Nat. Biotechnol..

[16] Schmidt K (2017). Identification of bacterial pathogens and antimicrobial resistance directly from clinical urines by nanopore-based metagenomic sequencing. J. Antimicrob. Chemother..

[17] Greninger AL (2015). Rapid metagenomic identification of viral pathogens in clinical samples by real-time nanopore sequencing analysis. Genome Med..

[18] Leggett RM, Heavens D, Caccamo M, Clark MD, Davey RP (2016). NanoOK: multi-reference alignment analysis of nanopore sequencing data, quality and error profiles. Bioinformatics.

[19] Ip CLC (2015). MinION Analysis and Reference Consortium: phase 1 data release and analysis. F1000Res..

[20] Melville JM, Moss TJ (2013). The immune consequences of preterm birth. Front. Neurosci..

[21] Alcon-Giner C (2017). Optimisation of 16S rRNA gut microbiota profiling of extremely low birth weight infants. BMC Genomics.

[22] Chen HN, Lee ML, Yu WK, Lin YW, Tsao LY (2009). Late-onset *Enterobacter cloacae* sepsis in very-low-birth-weight neonates: experience in a medical center. Pediatr. Neonatol..

[23] Hu Y, Gao GF, Zhu B (2017). The antibiotic resistome: gene flow in environments, animals and human beings. Front. Med..

[24] Serafini F (2011). Insights into physiological and genetic mupirocin susceptibility in bifidobacteria. Appl. Environ. Microbiol..

[25] Xu H, Miao V, Kwong W, Xia R, Davies J (2011). Identification of a novel fosfomycin resistance gene (*fosA2*) in *Enterobacter cloacae* from the Salmon River, Canada. Lett. Appl. Microbiol..

[26] Brooks B (2018). The developing premature infant gut microbiome is a major factor shaping the microbiome of neonatal intensive care unit rooms. Microbiome.

[27] Sim K (2015). Dysbiosis anticipating necrotizing enterocolitis in very premature infants. Clin. Infect. Dis..

[28] Liakopoulos A, Mevius D, Ceccarelli D (2016). A review of SHV extended-spectrum β-lactamases: neglected yet ubiquitous. Front. Microbiol..

[29] *Antimicrobial wild type distributions of microorganisms* Version 5.26 (European Committee on Antimicrobial Susceptibility Testing, 2018); https://mic.eucast.org/Eucast2/

[30] Brown BL, Watson M, Minot SS, Rivera MC, Franklin RB (2017). MinION™ nanopore sequencing of environmental metagenomes: a synthetic approach. Gigascience.

[31] Anand RJ, Leaphart CL, Mollen KP, Hackam DJ (2007). The role of the intestinal barrier in the pathogenesis of necrotizing enterocolitis. Shock.

[32] Hodzic Z, Bolock AM, Good M (2017). The role of mucosal immunity in the pathogenesis of necrotizing enterocolitis. Front. Pediatr..

[33] Wyres KL, Holt KE (2016). *Klebsiella pneumoniae* population genomics and antimicrobial-resistant clones. Trends Microbiol..

[34] Kielbasa SM, Wan R, Sato K, Horton P, Frith MC (2011). Adaptive seeds tame genomic sequence comparison. Genome Res..

[35] Bolger AM, Lohse M, Usadel B (2014). Trimmomatic: a flexible trimmer for Illumina sequence data. Bioinformatics.

[36] Altschul SF, Gish W, Miller W, Myers EW, Lipman DJ (1990). Basic local alignment search tool. J. Mol. Biol..

[37] Huson DH (2016). MEGAN Community Edition: interactive exploration and analysis of large-scale microbiome sequencing data. PLoS Comput. Biol..

[38] Li H (2018). Minimap2: pairwise alignment for nucleotide sequences. Bioinformatics.

[39] Nicholls S, Quick JC, Tang S, Loman NJ (2019). Ultra-deep, long-read nanopore sequencing of mock microbial community standards. Gigascience.

[40] McArthur AG (2013). The comprehensive antibiotic resistance database. Antimicrob. Agents Chemother..

[41] van der Helm E (2017). Rapid resistome mapping using nanopore sequencing.. Nucleic Acids Res..

[42] Xia Y (2017). MinION nanopore sequencing enables correlation between resistome phenotype and genotype of coliform bacteria in municipal sewage.. Front. Microbiol..

[43] Arango-Argoty GA (2019). NanoARG: a web service for detecting and contextualizing antimicrobial resistance genes from nanopore-derived metagenomes.. Microbiome.

[44] Weisburg WG, Barns SM, Pelletier DA, Lane DJ (1991). 16S ribosomal DNA amplification for phylogenetic study. J. Bacteriol..

[45] Pruesse E, Peplies J, Glöckner FO (2012). SINA: accurate high-throughput multiple sequence alignment of ribosomal RNA genes. Bioinformatics.

[46] Campanella JJ, Bitincka L, Smalley J (2003). MatGAT: an application that generates similarity/identity matrices using protein or DNA sequences. BMC Bioinformatics.

[47] Jorgensen JH, Ferraro MJ (2009). Antimicrobial susceptibility testing: a review of general principles and contemporary practices. Clin. Infect. Dis..

[48] Koren S (2017). Canu: scalable and accurate long-read assembly via adaptive *k*-mer weighting and repeat separation. Genome Res..

[49] Vaser R, Sović I, Nagarajan N, Šikić M (2017). Fast and accurate de novo genome assembly from long uncorrected reads. Genome Res..

[50] Loman NJ, Quick J, Simpson JT (2015). A complete bacterial genome assembled de novo using only nanopore sequencing data. Nat. Methods.

[51] Zerbino D, Birney E (2008). Velvet: algorithms for de novo short read assembly using de Bruijn graphs. Genome Res..

[52] Sommer DD, Delcher AL, Salzberg SL, Pop M (2007). Minimus: a fast, lightweight genome assembler. BMC Bioinformatics.

[53] Kurtz S (2004). Versatile and open software for comparing large genomes. Genome Biol..

[54] Alikhan NF, Petty NK, Ben Zakour NL, Beatson SA (2011). BLAST Ring Image Generator (BRIG): simple prokaryote genome comparisons. BMC Genomics.

[55] Kolmogorov M, Yuan J, Lin Y, Pevzner PA (2019). Assembly of long, error-prone reads using repeat graphs. Nat. Biotechnol..

