# Supplementary material for: Rapid MinION profiling of preterm microbiota and antimicrobial-resistant pathogens
Source: Nat Microbiol. 2019 Dec 16;5(3):430–42. doi: 10.1038/s41564-019-0626-z (PMC7044117; doi:10.1038/s41564-019-0626-z)
Supplement: Supplementary file 1 — Supplementary Figs. 1–6 and Supplementary Table 5. [file 41564_2019_626_MOESM1_ESM.pdf]

nature microbiology

SUPPLEMENTARY INFORMATION

https://doi.org/10.1038/s41564-019-0626-z

In the format provided by the authors and unedited.

Rapid MinION profiling of preterm microbiota and antimicrobial-resistant pathogens

Richard M. Leggett[1,8*], Cristina Alcon-Giner[2,8], Darren Heavens[1], Shabhonam Caim[2], Thomas C. Brook[3], Magdalena Kujawska[2], Samuel Martin[1], Ned Peel[1], Holly Acford-Palmer[2], Lesley Hoyles[4], Paul Clarke[5,6], Lindsay J. Hall[2*] and Matthew D. Clark[1,7*]

[1]Earlham Institute, Norwich Research Park, Norwich, UK. [2]Quadram Institute Bioscience, Norwich Research Park, Norwich, UK. [3]University of Westminster, London, UK. [4]Nottingham Trent University, Nottingham, UK. [5]Norfolk and Norwich University Hospital, Norwich, UK. [6]Norwich Medical School, University of East Anglia, Norwich, UK. [7]Natural History Museum, London, UK. [8]These authors contributed equally to this work: R. M. Leggett, C. Alcon-Giner.
*e-mail: Richard.Leggett@earlham.ac.uk; lindsay.hall@quadram.ac.uk; matt.clark@nhm.ac.uk

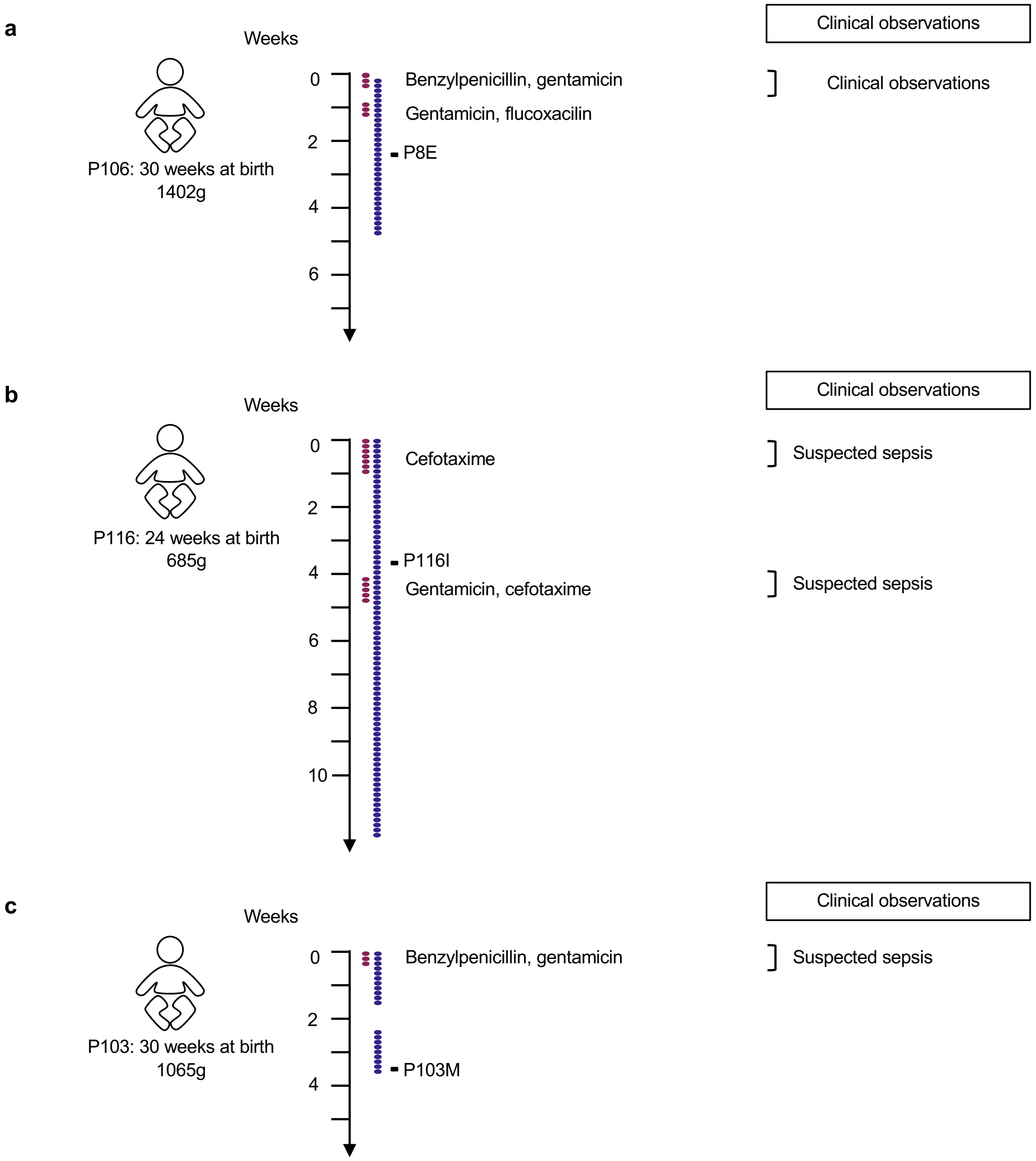

Supplementary Figure 1: Timeline diagrams for healthy preterms (P106, P116, P103).
Timeline diagrams indicating time points of faecal sample collection, duration of antibiotic, probiotic treatment, and relevant clinical observations. The timeline diagrams are divided in weeks and dots represent days within the scale. Blue dots indicate days of probiotic treatment, red dots antibiotic treatment, and black squares time points for sample collection. (**a**) timeline diagram for preterm P106, (**b**) timeline diagram for preterm P116 and (**c**) timeline diagram for preterm P103.

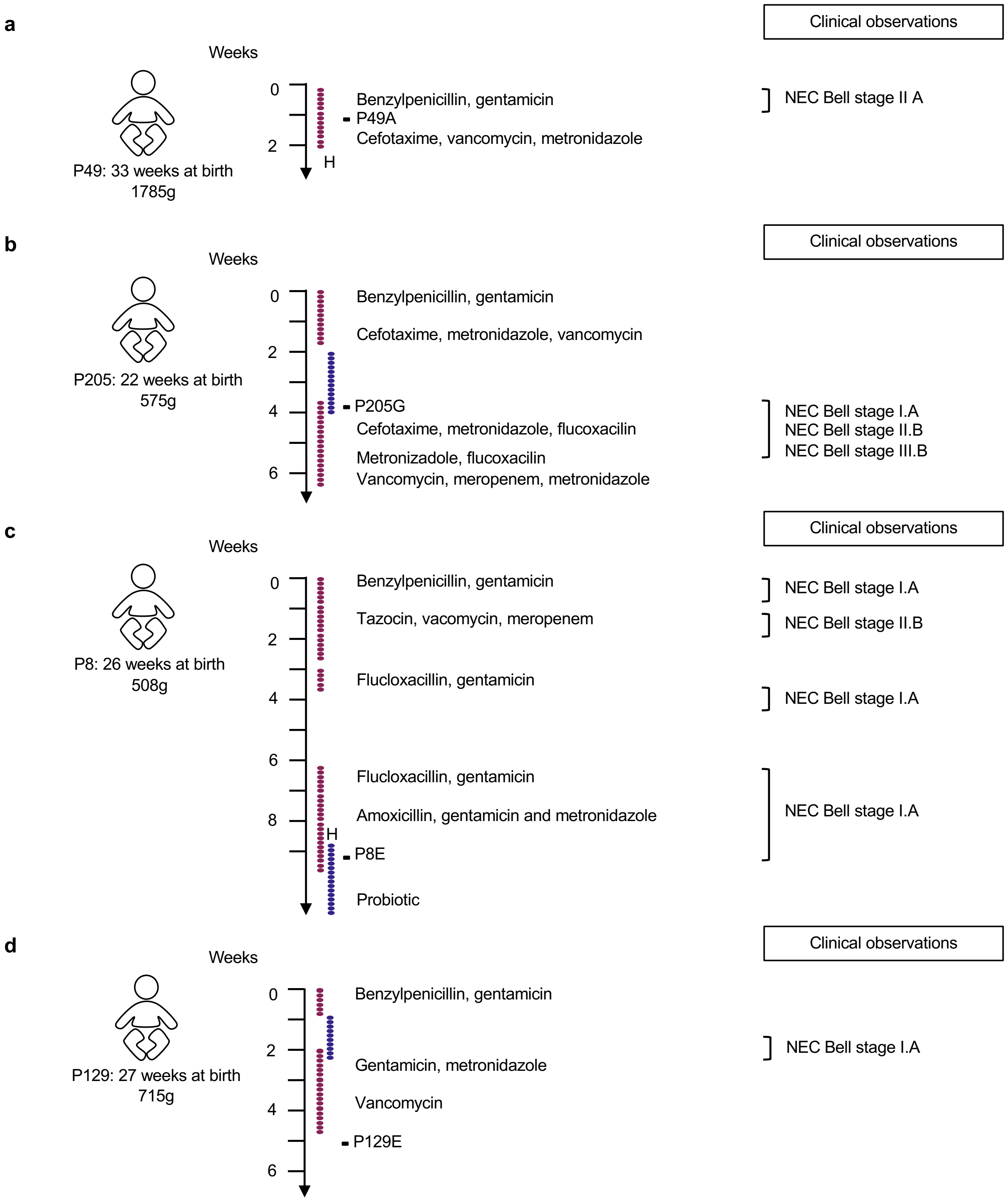

Supplementary Figure 2: Timeline diagrams for preterms diagnosed with NEC (P49, P205, P8 and P129). Timeline diagrams indicating time points of faecal sample collection, duration of antibiotic, probiotic treatment, and relevant clinical observations. The timeline diagrams are divided in weeks and dots represent days within the scale. Blue dots indicate days of probiotic treatment, red dots antibiotic treatment, black squares time points for sample collection, letter H transfer of the preterm to another hospital. Clinical observations highlight Bell stages of necrotising enterocolitis (Gregory, DeForge et al. 2011) commonly used by clinicians to assign the severity of this disease. (**a**) timeline diagram for preterm P49, (**b**) timeline diagram for preterm P205 and (**c**) timeline diagram for preterm P8, (**d**) timeline diagram for preterm P129.

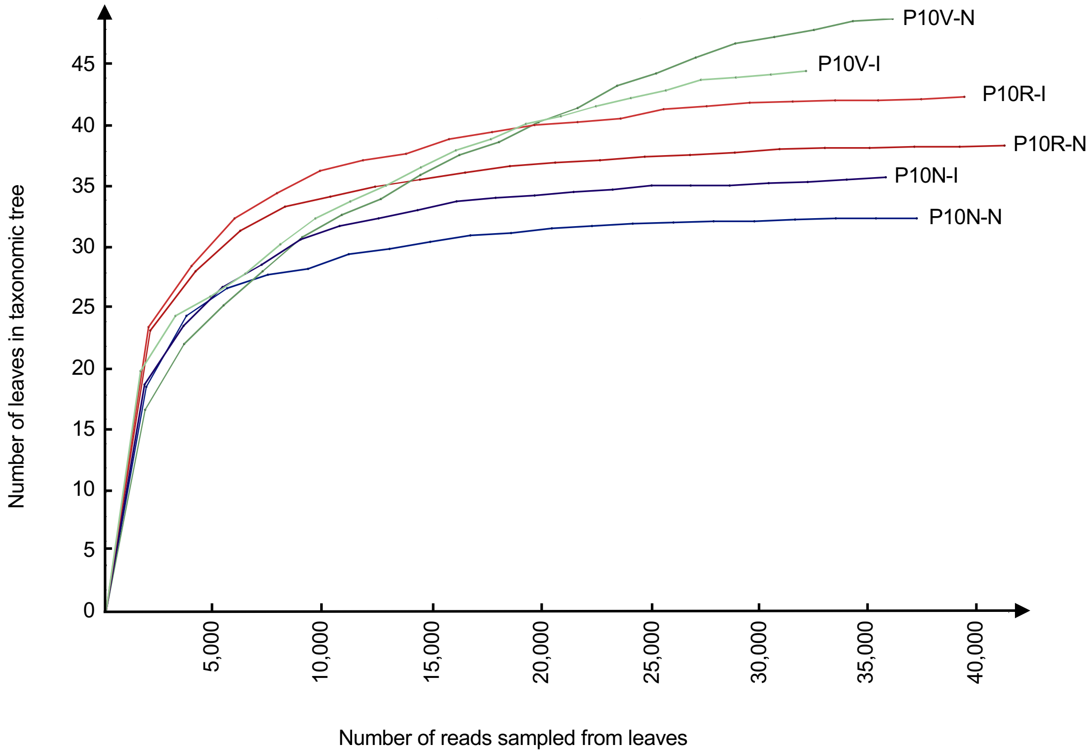

Supplementary Figure 3: Rarefaction curves comparing MinION and Illumina for preterm P10.
Rarefaction curves representing number of species (leaves) detected in the taxonomic tree vs number of reads sampled. Three samples from preterm P10 were analysed: samples (P10N-N, P10R-N and P10V-N) were sequenced with MinION technology, while samples (P10N-I, P10R-I and P10V-I) were sequenced with Illumina technology.

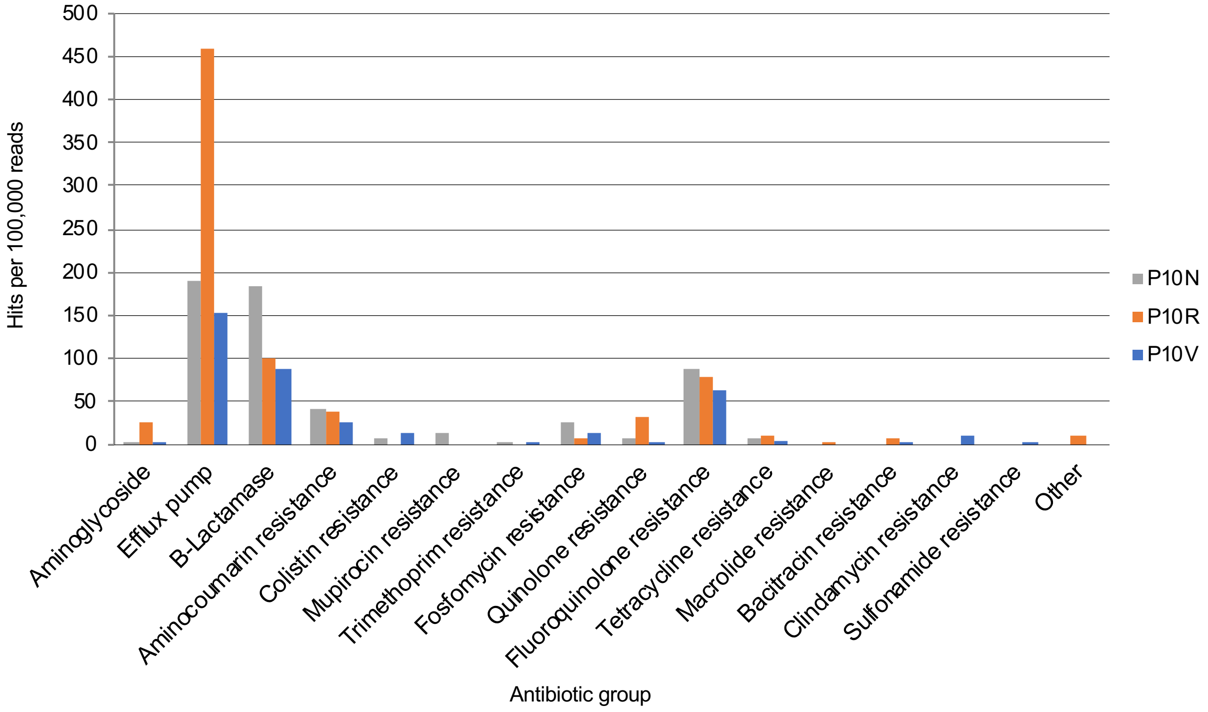

Supplementary Figure 4: Comparison of AMR hits found in preterm P10.
Height of bars indicates number of CARD database hits per 100,000 reads to each of 16 antibiotic resistance groups for sample P10N (grey, 13 days after birth), P10R (orange, 28 days after birth), P10V (blue, 64 days after birth).

a

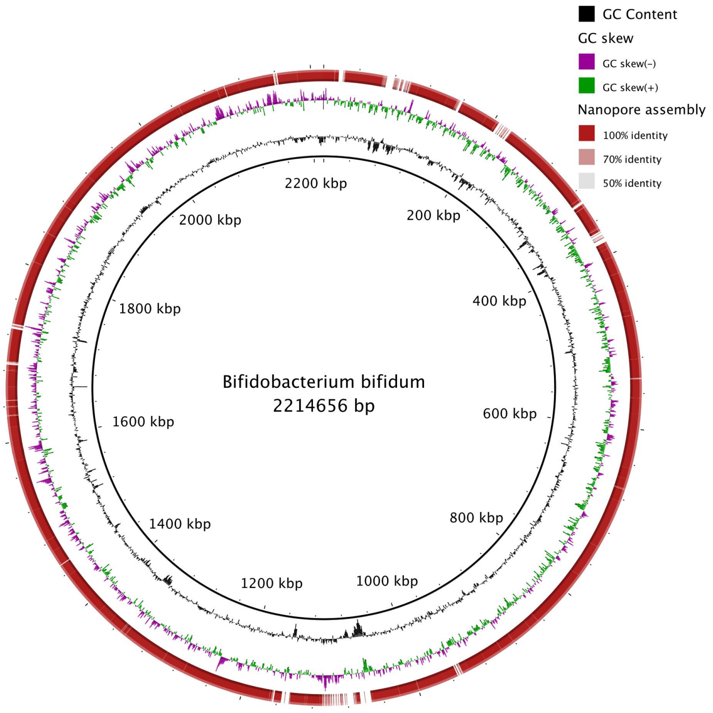

b

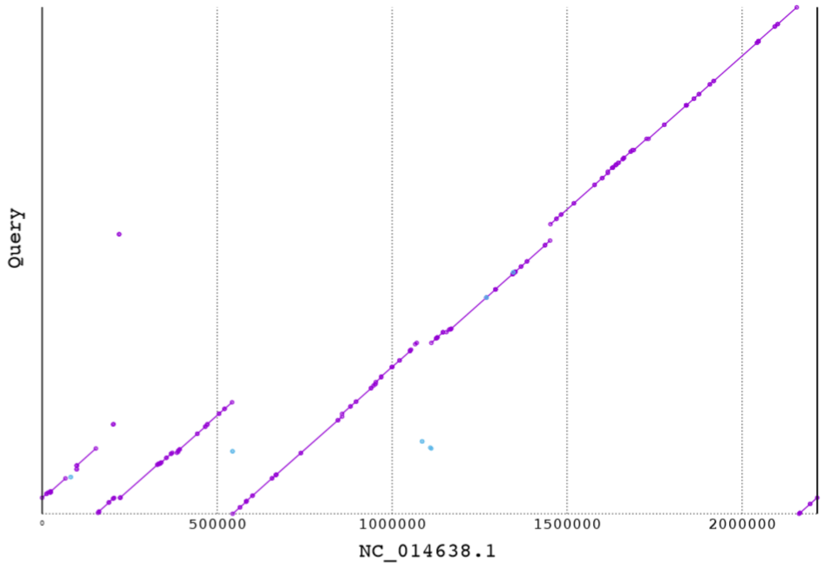

Supplementary Figure 5: Comparison between MinION assembly of *Bifidobacterium bifidum* and the reference strain *Bifidobacterium bifidum* PRL2010.

(**a**) BRIG plot showing BLAST alignment identity between *Bifidobacterium bifidum* assembled out of sample P103M and the reference strain NC_014638.1. Also shown is reference GC content and skew. (**b**) MUMmer dot plots illustrating alignment between the assembly and reference genome. Purple dots/lines indicate forward alingnments; blue dots/lines indicate reverse alignments.

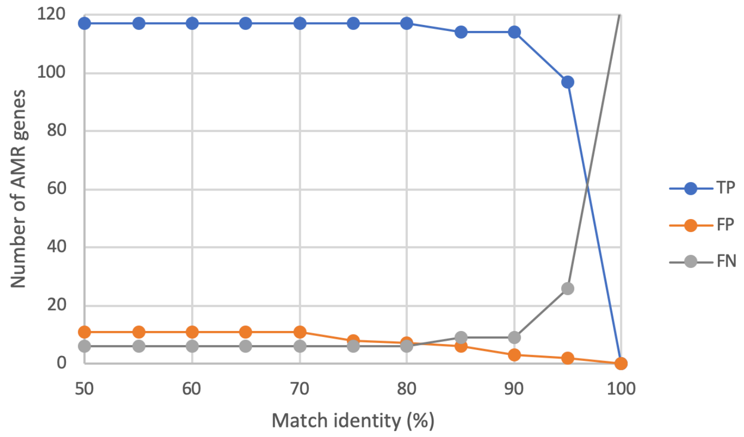

Supplementary Figure 6: Effect of AMR BLAST sequence identity when applied to nanopore sequencing of the Zymo Mock Community Standard.
The graph shows how true positive (TP), false positive (FP) and false negative (FN) varies with choice of BLAST match identity. At 80%, there is the highest TP (117) and lowest FN (6), while FP remains low (7). In all cases, maximum e-value of 0.001 and minimum match length of 200bp was required.

Supplementary Table 5: *Klebsiella pneumoniae* 16S rRNA gene alignment analysis

a) Alignment of partial 16S rRNA gene sequences (892 positions) of 9 isolates of *Klebsiella pneumoniae*. The alignment demonstrates nucleotide differences between the isolates.

```
K_L1     1  ATGCAGTCGAGCGGTAGCACAGAGAGCTTGCTCTCGGGTGACGAGCGGCGGACGGGTGAGTAATGTCTGG
K_L2     1  ATGCAGTCGAGCGGTAGCACAGAGAGCTTGCTCTCGGGTGACGAGCGGCGGACGGGTGAGTAATGTCTGG
K_L3     1  ATGCAGTCGAGCGGTAGCACAGAGAGCTTGCTCTCGGGTGACGAGCGGCGGACGGGTGAGTAATGTCTGG
K_M1     1  ATGCAGTCGAGCGGTAGCACAGAGAGCTTGCTCTCGGGTGACGAGCGGCGGACGGGTGAGTAATGTCTGG
K_M2     1  ATGCAGTCGAGCGGTAGCACAGAGAGCTTGCTCTCGGGTGACGAGCGGCGGACGGGTGAGTAATGTCTGG
K_M3     1  ATGCAGTCGAGCGGTAGCACAGAGAGCTTGCTCTCGGGTGACGAGCGGCGGACGGGTGAGTAATGTCTGG
K_S1     1  ATGCAGTCGAGCGGTAGCACAGAGAGCTTGCTCTCGGGTGACGAGCGGCGGACGGGTGAGTAATGTCTGG
K_S2     1  ATGCAGTCGAGCGGTAGCACAGAGAGCTTGCTCTCGGGTGACGAGCGGCGGACGGGTGAGTAATGTCTGG
K_S3     1  ATGCAGTCGAGCGGTAGCACAGAGAGCTTGCTCTCGGGTGACGAGCGGCGGACGGGTGAGTAATGTCTGG

K_L1    71  GAAACTGCCTGATGGAGGGGGATAACTACTGGAAACGGTAGCTAATACCGCATAATGTCGCAAGACCAAA
K_L2    71  GAAACTGCCTGATGGAGGGGGATAACTACTGGAAACGGTAGCTAATACCGCATAATGTCGCAAGACCAAA
K_L3    71  GAAACTGCCTGATGGAGGGGGATAACTACTGGAAACGGTAGCTAATACCGCATAATGTCGCAAGACCAAA
K_M1    71  GAAACTGCCTGATGGAGGGGGATAACTACTGGAAACGGTAGCTAATACCGCATAATGTCGCAAGACCAAA
K_M2    71  GAAACTGCCTGATGGAGGGGGATAACTACTGGAAACGGTAGCTAATACCGCATAATGTCGCAAGACCAAA
K_M3    71  GAAACTGCCTGATGGAGGGGGATAACTACTGGAAACGGTAGCTAATACCGCATAATGTCGCAAGACCAAA
K_S1    71  GAAACTGCCTGATGGAGGGGGATAACTACTGGAAACGGTAGCTAATACCGCATAATGTCGCAAGACCAAA
K_S2    71  GAAACTGCCTGATGGAGGGGGATAACTACTGGAAACGGTAGCTAATACCGCATAATGTCGCAAGACCAAA
K_S3    71  GAAACTGCCTGATGGAGGGGGATAACTACTGGAAACGGTAGCTAATACCGCATAATGTCGCAAGACCAAA

K_L1   141  GTGGGGGACCTTCGGGCCTCATGCCATCAGATGTGCCCAGATGGGATTAGCTAGTAGGTGGGGTAACGGC
K_L2   141  GTGGGGGACCTTCGGGCCTCATGCCATCAGATGTGCCCAGATGGGATTAGCTAGTAGGTGGGGTAACGGC
K_L3   141  GTGGGGGACCTTCGGGCCTCATGCCATCAGATGTGCCCAGATGGGATTAGCTAGTAGGTGGGGTAACGGC
K_M1   141  GTGGGGGACCTTCGGGCCTCATGCCATCAGATGTGCCCAGATGGGATTAGCTAGTAGGTGGGGTAACGGC
K_M2   141  GTGGGGGACCTTCGGGCCTCATGCCATCAGATGTGCCCAGATGGGATTAGCTAGTAGGTGGGGTAACGGC
K_M3   141  GTGGGGGACCTTCGGGCCTCATGCCATCAGATGTGCCCAGATGGGATTAGCTAGTAGGTGGGGTAACGGC
K_S1   141  GTGGGGGACCTTCGGGCCTCATGCCATCAGATGTGCCCAGATGGGATTAGCTAGTAGGTGGGGTAACGGC
K_S2   141  GTGGGGGACCTTCGGGCCTCATGCCATCAGATGTGCCCAGATGGGATTAGCTAGTAGGTGGGGTAACGGC
K_S3   141  GTGGGGGACCTTCGGGCCTCATGCCATCAGATGTGCCCAGATGGGATTAGCTAGTAGGTGGGGTAACGGC

K_L1   211  TCACCTAGGCGACGATCCCTAGCTGGTCTGAGAGGATGACCAGCCACACTGGAACTGAGACACGGTCCAG
K_L2   211  TCACCTAGGCGACGATCCCTAGCTGGTCTGAGAGGATGACCAGCCACACTGGAACTGAGACACGGTCCAG
K_L3   211  TCACCTAGGCGACGATCCCTAGCTGGTCTGAGAGGATGACCAGCCACACTGGAACTGAGACACGGTCCAG
K_M1   211  TCACCTAGGCGACGATCCCTAGCTGGTCTGAGAGGATGACCAGCCACACTGGAACTGAGACACGGTCCAG
K_M2   211  TCACCTAGGCGACGATCCCTAGCTGGTCTGAGAGGATGACCAGCCACACTGGAACTGAGACACGGTCCAG
K_M3   211  TCACCTAGGCGACGATCCCTAGCTGGTCTGAGAGGATGACCAGCCACACTGGAACTGAGACACGGTCCAG
K_S1   211  TCACCTAGGCGACGATCCCTAGCTGGTCTGAGAGGATGACCAGCCACACTGGAACTGAGACACGGTCCAG
K_S2   211  TCACCTAGGCGACGATCCCTAGCTGGTCTGAGAGGATGACCAGCCACACTGGAACTGAGACACGGTCCAG
K_S3   211  TCACCTAGGCGACGATCCCTAGCTGGTCTGAGAGGATGACCAGCCACACTGGAACTGAGACACGGTCCAG

K_L1   281  ACTCCTACGGGAGGCAGCAGTGGGGAATATTGCACAATGGGCGCAAGCCTGATGCAGCCATGCCGCGTGT
K_L2   281  ACTCCTACGGGAGGCAGCAGTGGGGAATATTGCACAATGGGCGCAAGCCTGATGCAGCCATGCCGCGTGT
K_L3   281  ACTCCTACGGGAGGCAGCAGTGGGGAATATTGCACAATGGGCGCAAGCCTGATGCAGCCATGCCGCGTGT
K_M1   281  ACTCCTACGGGAGGCAGCAGTGGGGAATATTGCACAATGGGCGCAAGCCTGATGCAGCCATGCCGCGTGT
K_M2   281  ACTCCTACGGGAGGCAGCAGTGGGGAATATTGCACAATGGGCGCAAGCCTGATGCAGCCATGCCGCGTGT
K_M3   281  ACTCCTACGGGAGGCAGCAGTGGGGAATATTGCACAATGGGCGCAAGCCTGATGCAGCCATGCCGCGTGT
K_S1   281  ACTCCTACGGGAGGCAGCAGTGGGGAATATTGCACAATGGGCGCAAGCCTGATGCAGCCATGCCGCGTGT
K_S2   281  ACTCCTACGGGAGGCAGCAGTGGGGAATATTGCACAATGGGCGCAAGCCTGATGCAGCCATGCCGCGTGT
K_S3   281  ACTCCTACGGGAGGCAGCAGTGGGGAATATTGCACAATGGGCGCAAGCCTGATGCAGCCATGCCGCGTGT

K_L1   351  GTGAAGAAGGCCTTCGGGTTGTAAAGCACTTTCAGCGGGGAGGAAGGCGATAAGGTTAATAACCTTGTCG
K_L2   351  GTGAAGAAGGCCTTCGGGTTGTAAAGCACTTTCAGCGGGGAGGAAGGCGATAAGGTTAATAACCTTGTCG
K_L3   351  GTGAAGAAGGCCTTCGGGTTGTAAAGCACTTTCAGCGGGGAGGAAGGCGATAAGGTTAATAACCTTGTCG
K_M1   351  GTGAAGAAGGCCTTCGGGTTGTAAAGCACTTTCAGCGGGGAGGAAGGCGATAAGGTTAATAACCTTGTCG
K_M2   351  GTGAAGAAGGCCTTCGGGTTGTAAAGCACTTTCAGCGGGGAGGAAGGCGATAAGGTTAATAACCTTGTCG
K_M3   351  GTGAAGAAGGCCTTCGGGTTGTAAAGCACTTTCAGCGGGGAGGAAGGCGATAAGGTTAATAACCTTGTCG
K_S1   351  GTGAAGAAGGCCTTCGGGTTGTAAAGCACTTTCAGCGGGGAGGAAGGCGTTAAGGTTAATAACCTTGTCG
K_S2   351  GTGAAGAAGGCCTTCGGGTTGTAAAGCACTTTCAGCGGGGAGGAAGGCGATAAGGTTAATAACCTTGTCG
K_S3   351  GTGAAGAAGGCCTTCGGGTTGTAAAGCACTTTCAGCGGGGAGGAAGGCGATAAGGTTAATAACCTTGTCG

K_L1   421  ATTGACGTTACCCGCAGAAGAAGCACCGGCTAACTCCGTGCCAGCAGCCGCGGTAATACGGAGGGTGCAA
K_L2   421  ATTGACGTTACCCGCAGAAGAAGCACCGGCTAACTCCGTGCCAGCAGCCGCGGTAATACGGAGGGTGCAA
K_L3   421  ATTGACGTTACCCGCAGAAGAAGCACCGGCTAACTCCGTGCCAGCAGCCGCGGTAATACGGAGGGTGCAA
```

```
K_M1   421 ATTGACGTTACCCGCAGAAGAAGCACCGGCTAACTCCGTGCCAGCAGCCGCGGTAATACGGAGGGTGCAA
K_M2   421 ATTGACGTTACCCGCAGAAGAAGCACCGGCTAACTCCGTGCCAGCAGCCGCGGTAATACGGAGGGTGCAA
K_M3   421 ATTGACGTTACCCGCAGAAGAAGCACCGGCTAACTCCGTGCCAGCAGCCGCGGTAATACGGAGGGTGCAA
K_S1   421 ATTGACGTTACCCGCAGAAGAAGCACCGGCTAACTCCGTGCCAGCAGCCGCGGTAATACGGAGGGTGCAA
K_S2   421 ATTGACGTTACCCGCAGAAGAAGCACCGGCTAACTCCGTGCCAGCAGCCGCGGTAATACGGAGGGTGCAA
K_S3   421 ATTGACGTTACCCGCAGAAGAAGCACCGGCTAACTCCGTGCCAGCAGCCGCGGTAATACGGAGGGTGCAA

K_L1   491 GCGTTAATCGGAATTACTGGGCGTAAAGCGCACGCAGGCGGTCTGTCAAGTCGGATGTGAAATCCCCGGG
K_L2   491 GCGTTAATCGGAATTACTGGGCGTAAAGCGCACGCAGGCGGTCTGTCAAGTCGGATGTGAAATCCCCGGG
K_L3   491 GCGTTAATCGGAATTACTGGGCGTAAAGCGCACGCAGGCGGTCTGTCAAGTCGGATGTGAAATCCCCGGG
K_M1   491 GCGTTAATCGGAATTACTGGGCGTAAAGCGCACGCAGGCGGTCTGTCAAGTCGGATGTGAAATCCCCGGG
K_M2   491 GCGTTAATCGGAATTACTGGGCGTAAAGCGCACGCAGGCGGTCTGTCAAGTCGGATGTGAAATCCCCGGG
K_M3   491 GCGTTAATCGGAATTACTGGGCGTAAAGCGCACGCAGGCGGTCTGTCAAGTCGGATGTGAAATCCCCGGG
K_S1   491 GCGTTAATCGGAATTACTGGGCGTAAAGCGCACGCAGGCGGTCTGTCAAGTCGGATGTGAAATCCCCGGG
K_S2   491 GCGTTAATCGGAATTACTGGGCGTAAAGCGCACGCAGGCGGTCTGTCAAGTCGGATGTGAAATCCCCGGG
K_S3   491 GCGTTAATCGGAATTACTGGGCGTAAAGCGCACGCAGGCGGTCTGTCAAGTCGGATGTGAAATCCCCGGG

K_L1   561 CTCAACCTGGGAACTGCATTCGAAACTGGCAGGCTAGAGTCTTGTAGAGGGGGGTAGAATTCCAGGTGTA
K_L2   561 CTCAACCTGGGAACTGCATTCGAAACTGGCAGGCTAGAGTCTTGTAGAGGGGGGTAGAATTCCAGGTGTA
K_L3   561 CTCAACCTGGGAACTGCATTCGAAACTGGCAGGCTAGAGTCTTGTAGAGGGGGGTAGAATTCCAGGTGTA
K_M1   561 CTCAACCTGGGAACTGCATTCGAAACTGGCAGGCTAGAGTCTTGTAGAGGGGGGTAGAATTCCAGGTGTA
K_M2   561 CTCAACCTGGGAACTGCATTCGAAACTGGCAGGCTAGAGTCTTGTAGAGGGGGGTAGAATTCCAGGTGTA
K_M3   561 CTCAACCTGGGAACTGCATTCGAAACTGGCAGGCTAGAGTCTTGTAGAGGGGGGTAGAATTCCAGGTGTA
K_S1   561 CTCAACCTGGGAACTGCATTCGAAACTGGCAGGCTAGAGTCTTGTAGAGGGGGGTAGAATTCCAGGTGTA
K_S2   561 CTCAACCTGGGAACTGCATTCGAAACTGGCAGGCTAGAGTCTTGTAGAGGGGGGTAGAATTCCAGGTGTA
K_S3   561 CTCAACCTGGGAACTGCATTCGAAACTGGCAGGCTAGAGTCTTGTAGAGGGGGGTAGAATTCCAGGTGTA

K_L1   631 GCGGTGAAATGCGTAGAGATCTGGAGGAATACCGGTGGCGAAGGCGGCCCCCTGGACAAAGACTGACGCT
K_L2   631 GCGGTGAAATGCGTAGAGATCTGGAGGAATACCGGTGGCGAAGGCGGCCCCCTGGACAAAGACTGACGCT
K_L3   631 GCGGTGAAATGCGTAGAGATCTGGAGGAATACCGGTGGCGAAGGCGGCCCCCTGGACAAAGACTGACGCT
K_M1   631 GCGGTGAAATGCGTAGAGATCTGGAGGAATACCGGTGGCGAAGGCGGCCCCCTGGACAAAGACTGACGCT
K_M2   631 GCGGTGAAATGCGTAGAGATCTGGAGGAATACCGGTGGCGAAGGCGGCCCCCTGGACAAAGACTGACGCT
K_M3   631 GCGGTGAAATGCGTAGAGATCTGGAGGAATACCGGTGGCGAAGGCGGCCCCCTGGACAAAGACTGACGCT
K_S1   631 GCGGTGAAATGCGTAGAGATCTGGAGGAATACCGGTGGCGAAGGCGGCCCCCTGGACAAAGACTGACGCT
K_S2   631 GCGGTGAAATGCGTAGAGATCTGGAGGAATACCGGTGGCGAAGGCGGCCCCCTGGACAAAGACTGACGCT
K_S3   631 GCGGTGAAATGCGTAGAGATCTGGAGGAATACCGGTGGCGAAGGCGGCCCCCTGGACAAAGACTGACGCT

K_L1   701 CAGGTGCGAAAGCGTGGGGAGCAAACAGGATTAGATACCCTGGTAGTCCACGCCGTAAACGATGTCGATT
K_L2   701 CAGGTGCGAAAGCGTGGGGAGCAAACAGGATTAGATACCCTGGTAGTCCACGCCGTAAACGATGTCGATT
K_L3   701 CAGGTGCGAAAGCGTGGGGAGCAAACAGGATTAGATACCCTGGTAGTCCACGCCGTAAACGATGTCGATT
K_M1   701 CAGGTGCGAAAGCGTGGGGAGCAAACAGGATTAGATACCCTGGTAGTCCACGCCGTAAACGATGTCGATT
K_M2   701 CAGGTGCGAAAGCGTGGGGAGCAAACAGGATTAGATACCCTGGTAGTCCACGCCGTAAACGATGTCGATT
K_M3   701 CAGGTGCGAAAGCGTGGGGAGCAAACAGGATTAGATACCCTGGTAGTCCACGCCGTAAACGATGTCGATT
K_S1   701 CAGGTGCGAAAGCGTGGGGAGCAAACAGGATTAGATACCCTGGTAGTCCACGCCGTAAACGATGTCGATT
K_S2   701 CAGGTGCGAAAGCGTGGGGAGCAAACAGGATTAGATACCCTGGTAGTCCACGCCGTAAACGATGTCGATT
K_S3   701 CAGGTGCGAAAGCGTGGGGAGCAAACAGGATTAGATACCCTGGTAGTCCACGCCGTAAACGATGTCGATT

K_L1   771 TGGAGGTTGTGCCCTTGAGGCGTGGCTTCCGGAGCTAACGCGTTAAATCGACCGCCTGGGGAGTACGGCC
K_L2   771 TGGAGGTTGTGCCCTTGAGGCGTGGCTTCCGGAGCTAACGCGTTAAATCGACCGCCTGGGGAGTACGGCC
K_L3   771 TGGAGGTTGTGCCCTTGAGGCGTGGCTTCCGGAGCTAACGCGTTAAATCGACCGCCTGGGGAGTACGGCC
K_M1   771 TGGAGGTTGTGCCCTTGAGGCGTGGCTTCCGGAGCTAACGCGTTAAATCGACCGCCTGGGGAGTACGGCC
K_M2   771 TGGAGGTTGTGCCCTTGAGGCGTGGCTTCCGGAGCTAACGCGTTAAATCGACCGCCTGGGGAGTACGGCC
K_M3   771 TGGAGGTTGTGCCCTTGAGGCGTGGCTTCCGGAGCTAACGCGTTAAATCGACCGCCTGGGGAGTACGGCC
K_S1   771 TGGAGGTTGTGCCCTTGAGGCGTGGCTTCCGGAGCTAACGCGTTAAATCGACCGCCTGGGGAGTACGGCC
K_S2   771 TGGAGGTTGTGCCCTTGAGGCGTGGCTTCCGGAGCTAACGCGTTAAATCGACCGCCTGGGGAGTACGGCC
K_S3   771 TGGAGGTTGTGCCCTTGAGGCGTGGCTTCCGGAGCTAACGCGTTAAATCGACCGCCTGGGGAGTACGGCC

K_L1   841 GCAAGGTTAAAACTCAAATGAATTGACGGGGGCCCGCACAAGCGGTGGAACA
K_L2   841 GCAAGGTTAAAACTCAAATGAATTGACGGGGGCCCGCACAAGCGGTGGAGCA
K_L3   841 GCAAGGTTAAAACTCAAATGAATTGACGGGGGCCCGCACAAGCGGTGGAGCA
K_M1   841 GCAAGGTTAAAACTCAAATGAATTGACGGGGGCCCGCACAAGCGGTGGAGCA
K_M2   841 GCAAGGTTAAAACTCAAATGAATTGACGGGGGCCCGCACAAGCGGTGGAGCA
K_M3   841 GCAAGGTTAAAACTCAAATGAATTGACGGGGGCCCGCACAAGCGGTGGAGCA
K_S1   841 GCAAGGTTAAAACTCAAATGAATTGACGGGGGCCCGCACAAGCGGTGGAGCA
K_S2   841 GCAAGGTTAAAACTCAAATGAATTGACGGGGGCCCGCACAAGCGGTGGAGCA
K_S3   841 GCAAGGTTAAAACTCAAATGAATTGACGGGGGCCCGCACAAGCGGTGGAGCA
```

b) Levels of 16S rRNA gene sequence similarity and identity between the 9 *Klebsiella pneumoniae* isolates. The similarity and identity levels range from 99.8% to 100%. The values on the upper right are levels of 16S rRNA gene identity, and the values on the lower left are levels of 16S rRNA gene similarity.

	1. K_L1	2. K_L2	3. K_L3	4. K_M1	5. K_M2	6. K_M3	7. K_S1	8. K_S2	9. K_S3
1. K_L1		99.9	99.9	99.9	99.9	99.9	99.8	99.9	99.9
2. K_L2	99.9		100	100	100	100	99.9	100	100
3. K_L3	99.9	100		100	100	100	99.9	100	100
4. K_M1	99.9	100	100		100	100	99.9	100	100
5. K_M2	99.9	100	100	100		100	99.9	100	100
6. K_M3	99.9	100	100	100	100		99.9	100	100
7. K_S1	99.8	99.9	99.9	99.9	99.9	99.9		99.9	99.9
8. K_S2	99.9	100	100	100	100	100	99.9		100
9. K_S3	99.9	100	100	100	100	100	99.9	100	
