# Supplementary material for: Rapid MinION profiling of preterm microbiota and antimicrobial-resistant pathogens
Source: Nat Microbiol. 2019 Dec 16;5(3):430–42. doi: 10.1038/s41564-019-0626-z (PMC7044117; doi:10.1038/s41564-019-0626-z)
Supplement: Supplementary file 4 — NanoOK report for mock community (run N79596 MOCK SQKMAP006 24082015). [file 41564_2019_626_MOESM4_ESM.pdf]

NanoOK report for N79596_MOCK_SQKMAP006_24082015

Pass and fail counts

Type	Pass	Fail
Template	71714	0
Complement	71714	0
2D	71714	0

Read lengths

Type	NumReads	TotalBases	Mean	Longest	Shortest	N50	N50Count	N90	N90Count
Template	71714	195284744	2723.10	21916	129	4904	13744	1273	42670
Complement	71714	180833032	2521.59	20665	116	4550	13671	1184	42657
2D	71714	218590744	3048.09	40561	141	5499	13738	1435	42664

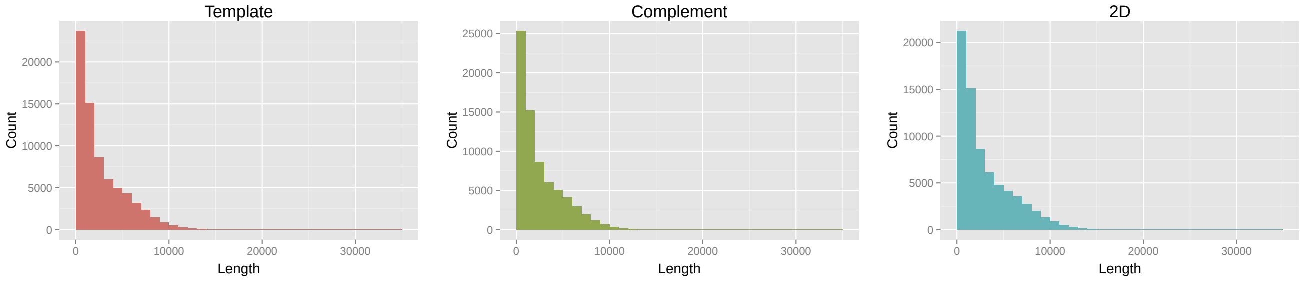

Template alignments

Number of reads	71714	
Number of reads with alignments	25099	(35.00%)
Number of reads without alignments	46615	(65.00%)

ID	Size	Number of Reads	% of Reads	Mean read length	Aligned bases	Mean coverage	Longest Perf Kmer
Acinetobacter baumannii	3976747	34	0.05	3897.35	133246	0.03	46
Actinomyces odontolyticus	2391230	8	0.01	3257.63	7811	0.00	28
Bacillus cereus	5224283	538	0.75	2880.09	1634026	0.31	62
Bacteroides vulgatus	5163189	8	0.01	3807.13	34957	0.01	29
Clostridium beijerinckii	6000632	465	0.65	2638.74	1231829	0.21	49
Control sequence	3560	12	0.02	2906.50	19046	5.35	28
Deinococcus radiodurans 1	2648638	19	0.03	3491.21	55236	0.02	42
Deinococcus radiodurans 2	412348	4	0.01	2885.25	11990	0.03	31
Enterococcus faecalis	2739625	25	0.03	3586.24	46771	0.02	51
Escherichia coli	4641652	8112	11.31	5854.24	52613058	11.33	87
Helicobacter pylori	1667867	144	0.20	4528.35	731969	0.44	40
Lactobacillus gasseri	1894360	52	0.07	3138.79	176704	0.09	34
Listeria monocytogenes	2944528	41	0.06	6242.54	260306	0.09	39
Neisseria meningitidis	2272360	186	0.26	2615.92	512471	0.23	48
Propionibacterium acnes	2560265	142	0.20	5258.77	794011	0.31	46
Pseudomonas aeruginosa	6264404	1094	1.53	6650.60	7725124	1.23	47
Rhodobacter sphaeroides 1	3188524	2762	3.85	3034.55	8514434	2.67	47
Rhodobacter sphaeroides 2	943018	882	1.23	3102.91	2660052	2.82	44
Staphylococcus aureus	2872915	859	1.20	2176.04	1934292	0.67	65
Staphylococcus epidermidis	2499279	1810	2.52	5214.51	9780899	3.91	59
Streptococcus agalactiae	2160267	190	0.26	5181.01	1055562	0.49	74
Streptococcus mutans	2032925	7695	10.73	3207.98	26884543	13.22	66
Streptococcus pneumoniae	2160842	17	0.02	1907.65	19705	0.01	30

Complement alignments

Number of reads	71714	
Number of reads with alignments	26626	(37.13%)
Number of reads without alignments	45088	(62.87%)

ID	Size	Number of Reads	% of Reads	Mean read length	Aligned bases	Mean coverage	Longest Perf Kmer
Acinetobacter baumannii	3976747	39	0.05	3606.31	152613	0.04	43
Actinomyces odontolyticus	2391230	3	0.00	1414.67	2305	0.00	45
Bacillus cereus	5224283	544	0.76	2618.24	1548939	0.30	50
Bacteroides vulgatus	5163189	9	0.01	2974.67	31138	0.01	31
Clostridium beijerinckii	6000632	516	0.72	2298.26	1234291	0.21	63
Control sequence	3560	6	0.01	2795.83	7470	2.10	26
Deinococcus radiodurans 1	2648638	14	0.02	3024.71	38220	0.01	36
Deinococcus radiodurans 2	412348	3	0.00	4065.33	9198	0.02	28
Enterococcus faecalis	2739625	15	0.02	2684.73	37391	0.01	37
Escherichia coli	4641652	7197	10.04	5634.55	45382301	9.78	60
Helicobacter pylori	1667867	127	0.18	4309.06	554267	0.33	36
Lactobacillus gasseri	1894360	71	0.10	2427.69	177302	0.09	43
Listeria monocytogenes	2944528	41	0.06	5697.22	250034	0.08	77
Neisseria meningitidis	2272360	115	0.16	2630.86	335017	0.15	58
Propionibacterium acnes	2560265	129	0.18	4840.42	690125	0.27	44
Pseudomonas aeruginosa	6264404	1268	1.77	6113.79	8387260	1.34	59
Rhodobacter sphaeroides 1	3188524	3893	5.43	2950.76	12048316	3.78	61
Rhodobacter sphaeroides 2	943018	1283	1.79	3033.56	3984801	4.23	53
Staphylococcus aureus	2872915	1099	1.53	1950.85	2216227	0.77	53
Staphylococcus epidermidis	2499279	2108	2.94	4820.47	10925868	4.37	62
Streptococcus agalactiae	2160267	200	0.28	4781.10	1052253	0.49	51
Streptococcus mutans	2032925	7931	11.06	2848.58	24891262	12.24	70
Streptococcus pneumoniae	2160842	15	0.02	2529.87	19671	0.01	28

2D alignments

Number of reads	71714	
Number of reads with alignments	53278	(74.29%)
Number of reads without alignments	18436	(25.71%)

ID	Size	Number of Reads	% of Reads	Mean read length	Aligned bases	Mean coverage	Longest Perf Kmer
Acinetobacter baumannii	3976747	68	0.09	3606.29	225276	0.06	85
Actinomyces odontolyticus	2391230	17	0.02	2267.59	17278	0.01	54
Bacillus cereus	5224283	1308	1.82	2304.81	3013953	0.58	150
Bacteroides vulgatus	5163189	20	0.03	3486.85	71583	0.01	79
Clostridium beijerinckii	6000632	1251	1.74	2205.97	2719065	0.45	132
Control sequence	3560	40	0.06	2963.73	82193	23.09	55
Deinococcus radiodurans 1	2648638	45	0.06	2604.51	88790	0.03	98
Deinococcus radiodurans 2	412348	8	0.01	2100.00	17270	0.04	75
Enterococcus faecalis	2739625	45	0.06	1555.62	66817	0.02	102
Escherichia coli	4641652	9613	13.40	5999.13	58714643	12.65	210
Helicobacter pylori	1667867	245	0.34	3937.67	986209	0.59	87
Lactobacillus gasseri	1894360	160	0.22	2055.27	335450	0.18	111
Listeria monocytogenes	2944528	53	0.07	6341.79	342404	0.12	112
Neisseria meningitidis	2272360	344	0.48	1949.83	673336	0.30	162
Propionibacterium acnes	2560265	190	0.26	4998.12	959162	0.37	130
Pseudomonas aeruginosa	6264404	1905	2.66	6709.39	12644049	2.02	212
Rhodobacter sphaeroides 1	3188524	10313	14.38	2745.14	27347438	8.58	151
Rhodobacter sphaeroides 2	943018	3289	4.59	2806.28	8647423	9.17	135
Staphylococcus aureus	2872915	2852	3.98	1895.46	5253804	1.83	152
Staphylococcus epidermidis	2499279	3802	5.30	5192.07	19819884	7.93	190
Streptococcus agalactiae	2160267	340	0.47	4660.89	1653289	0.77	171
Streptococcus mutans	2032925	17343	24.18	2481.64	44109255	21.70	223
Streptococcus pneumoniae	2160842	27	0.04	1065.67	27540	0.01	73

Acinetobacter baumannii error analysis

	Template	Complement	2D
Overall base identity (excluding indels)	64.64%	69.04%	70.09%
Aligned base identity (excluding indels)	76.75%	78.22%	86.64%
Identical bases per 100 aligned bases (including indels)	64.29%	63.63%	76.30%
Inserted bases per 100 aligned bases (including indels)	4.29%	2.43%	6.87%
Deleted bases per 100 aligned bases (including indels)	11.95%	16.22%	5.07%
Substitutions per 100 aligned bases (including indels)	19.48%	17.72%	11.76%
Mean insertion size	1.52	1.36	1.60
Mean deletion size	1.66	1.83	1.41

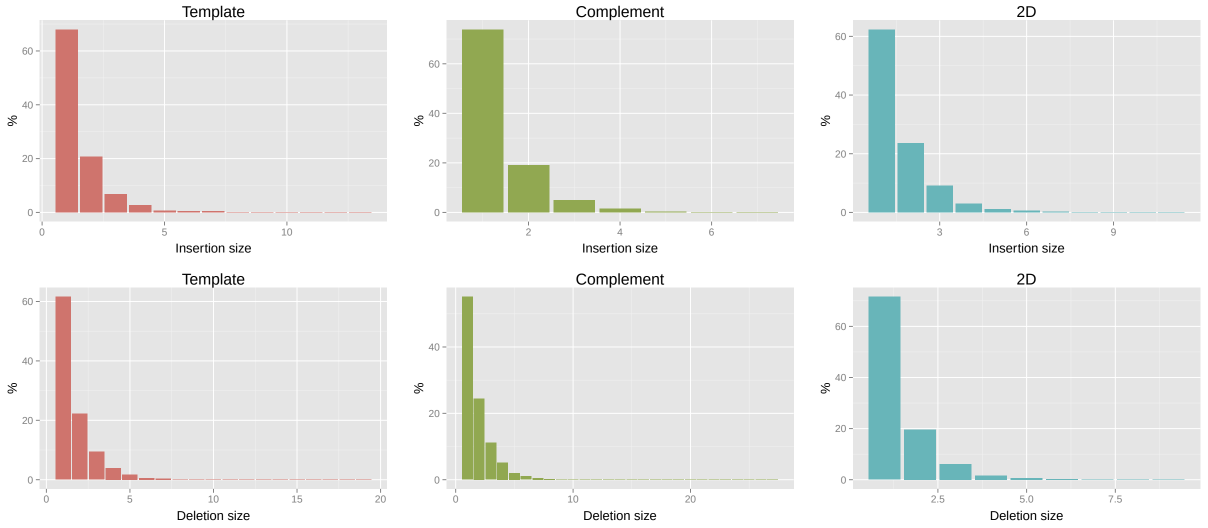

Acinetobacter baumannii read identity

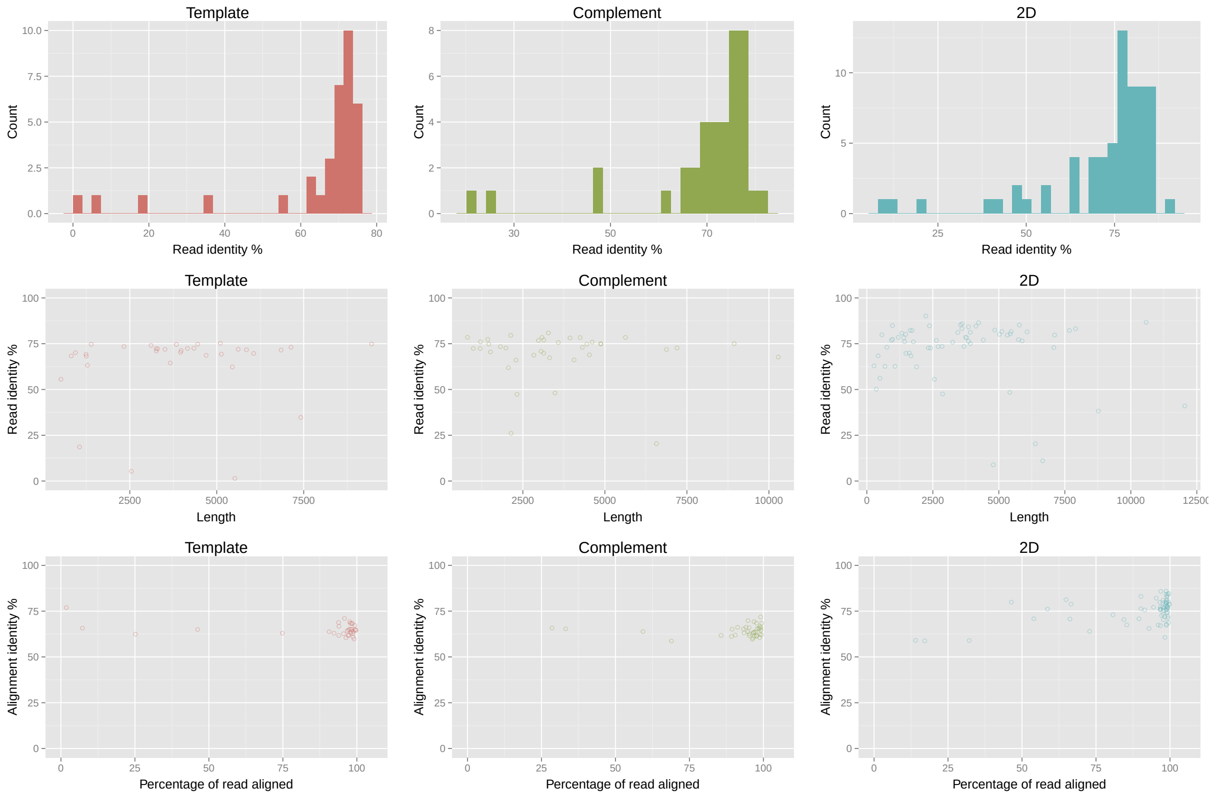

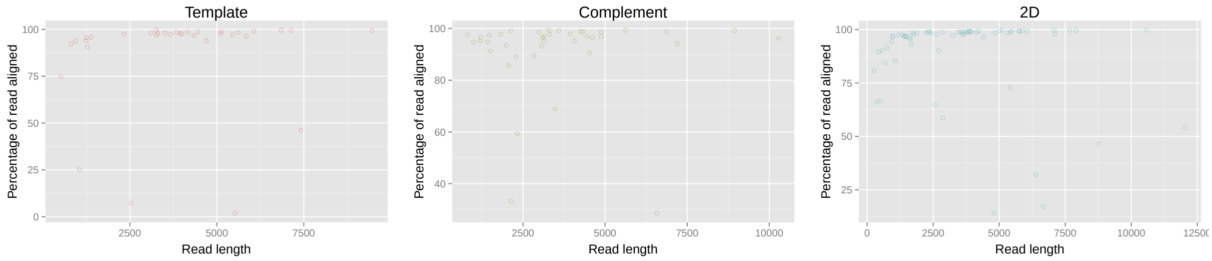

Acinetobacter baumannii perfect kmers

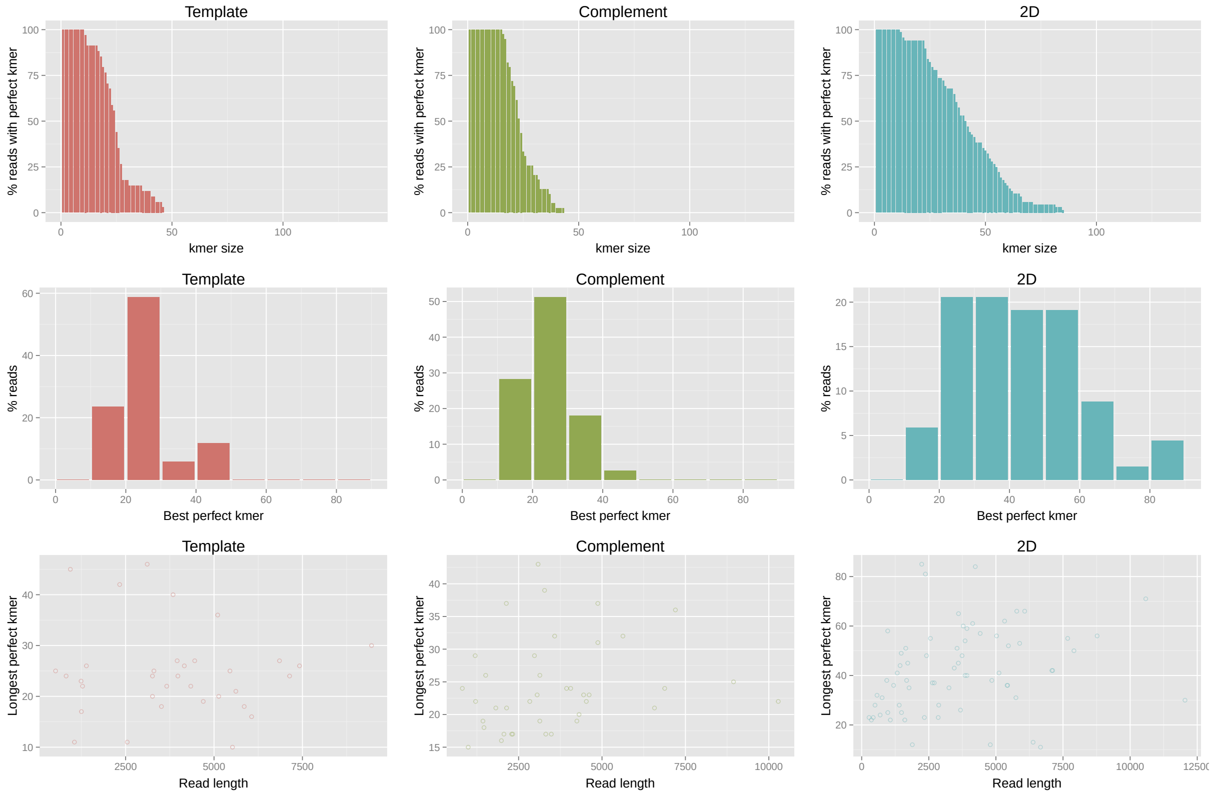

Acinetobacter baumannii coverage

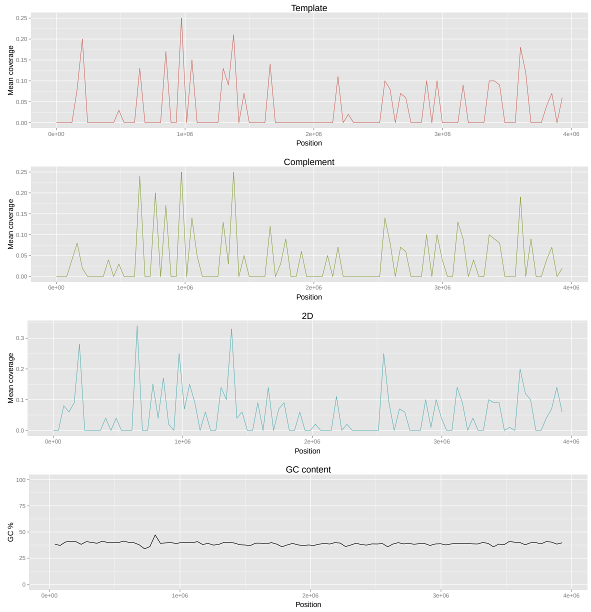

Acinetobacter baumannii 5-mer analysis

Under-represented 5-mers

	Template				Complement				2D			
Rank	kmer	Ref %	Read %	Diff %	kmer	Ref %	Read %	Diff %	kmer	Ref %	Read %	Diff %
1	AAAAA	0.493	0.085	-0.408	AAAAA	0.493	0.075	-0.418	TTTTT	0.487	0.103	-0.384
2	TTTTT	0.487	0.107	-0.381	TTTTT	0.487	0.090	-0.397	AAAAA	0.493	0.136	-0.357
3	TAAAA	0.415	0.066	-0.349	TAAAA	0.415	0.111	-0.304	TAAAA	0.415	0.188	-0.227
4	AAAAT	0.416	0.100	-0.316	TTAAA	0.418	0.135	-0.283	TTTTA	0.416	0.206	-0.210
5	TTAAA	0.418	0.119	-0.298	AAAAT	0.416	0.136	-0.280	AAAAT	0.416	0.216	-0.201
6	TTTAA	0.423	0.145	-0.278	TTTTA	0.416	0.148	-0.268	ATTTT	0.414	0.216	-0.198
7	TTTTA	0.416	0.172	-0.244	ATTTT	0.414	0.155	-0.259	TTTAA	0.423	0.229	-0.194
8	ATAAA	0.322	0.079	-0.243	TTTAA	0.423	0.184	-0.240	AATTT	0.359	0.195	-0.163
9	CAAAA	0.300	0.066	-0.234	AAATT	0.355	0.122	-0.233	TTAAA	0.418	0.259	-0.159
10	AAATT	0.355	0.144	-0.210	ATAAA	0.322	0.098	-0.224	AAATT	0.355	0.217	-0.137

Over-represented 5-mers

	Template				Complement				2D			
Rank	kmer	Ref %	Read %	Diff %	kmer	Ref %	Read %	Diff %	kmer	Ref %	Read %	Diff %
1	CGGGC	0.026	0.178	0.151	CGGCT	0.055	0.185	0.130	CGGGC	0.026	0.079	0.053
2	ATCGT	0.079	0.195	0.115	CGGCG	0.034	0.157	0.123	GCCGG	0.023	0.075	0.052
3	TCGTC	0.046	0.153	0.107	GCGGC	0.046	0.167	0.122	CGCAG	0.047	0.096	0.049
4	TCGTG	0.062	0.168	0.106	GCGTA	0.063	0.173	0.110	CCGGG	0.017	0.064	0.047
5	CGTCG	0.026	0.129	0.103	CCGGC	0.025	0.132	0.107	GCCCG	0.026	0.072	0.047
6	CCGGG	0.017	0.110	0.094	CGTAG	0.036	0.140	0.104	GGGCT	0.046	0.091	0.046
7	CTTCG	0.055	0.147	0.092	TGCGT	0.059	0.161	0.102	GCCGC	0.045	0.090	0.045
8	TCGTA	0.062	0.153	0.090	TCGTA	0.062	0.164	0.102	TACGA	0.063	0.108	0.045
9	GAGGA	0.033	0.122	0.089	GCGTG	0.048	0.145	0.096	CCTGG	0.018	0.062	0.044
10	CGGCT	0.055	0.144	0.089	TCGGC	0.056	0.152	0.096	CCCGG	0.017	0.061	0.044

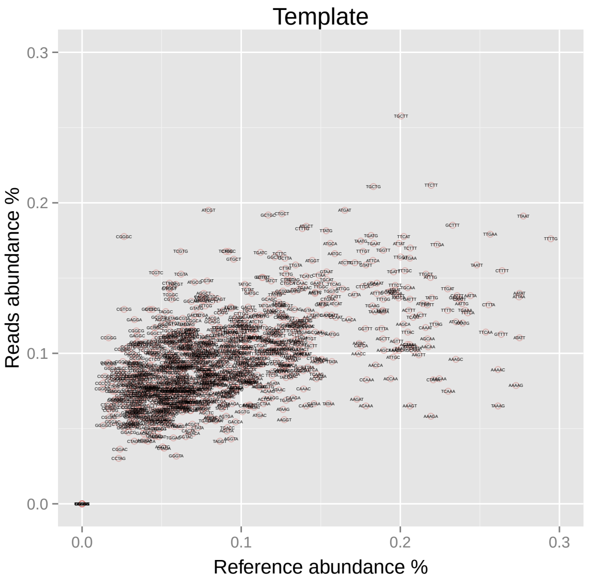

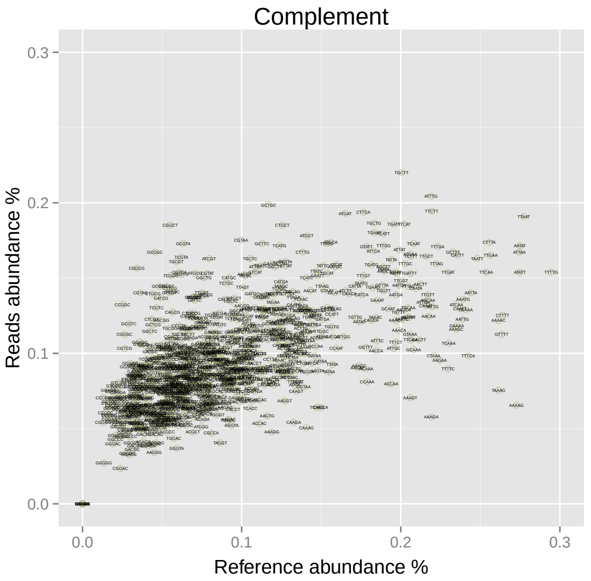

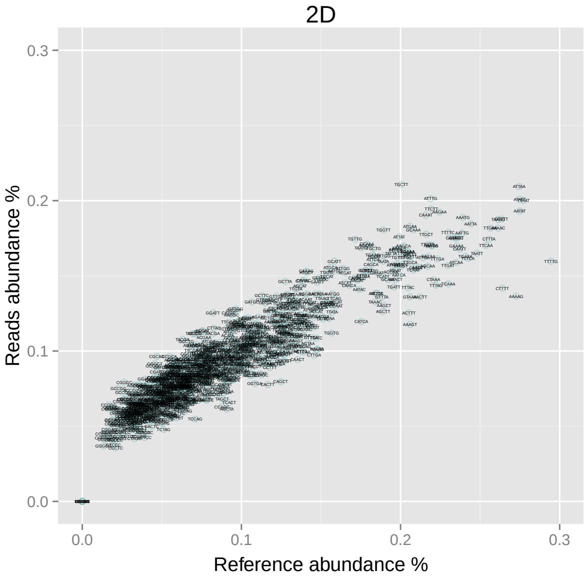

Acinetobacter baumannii GC content

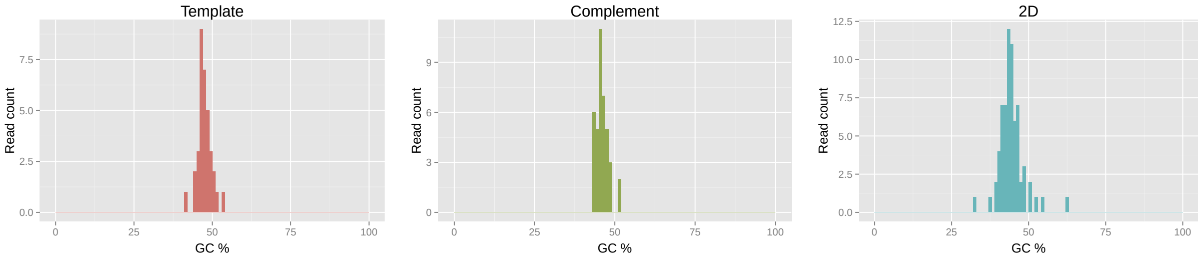

Actinomyces odontolyticus error analysis

	Template	Complement	2D
Overall base identity (excluding indels)	18.34%	35.67%	31.37%
Aligned base identity (excluding indels)	74.50%	78.36%	81.51%
Identical bases per 100 aligned bases (including indels)	61.18%	65.68%	69.99%
Inserted bases per 100 aligned bases (including indels)	2.83%	2.43%	7.27%
Deleted bases per 100 aligned bases (including indels)	15.04%	13.75%	6.86%
Substitutions per 100 aligned bases (including indels)	20.94%	18.13%	15.88%
Mean insertion size	1.28	1.33	1.57
Mean deletion size	1.82	1.88	1.50

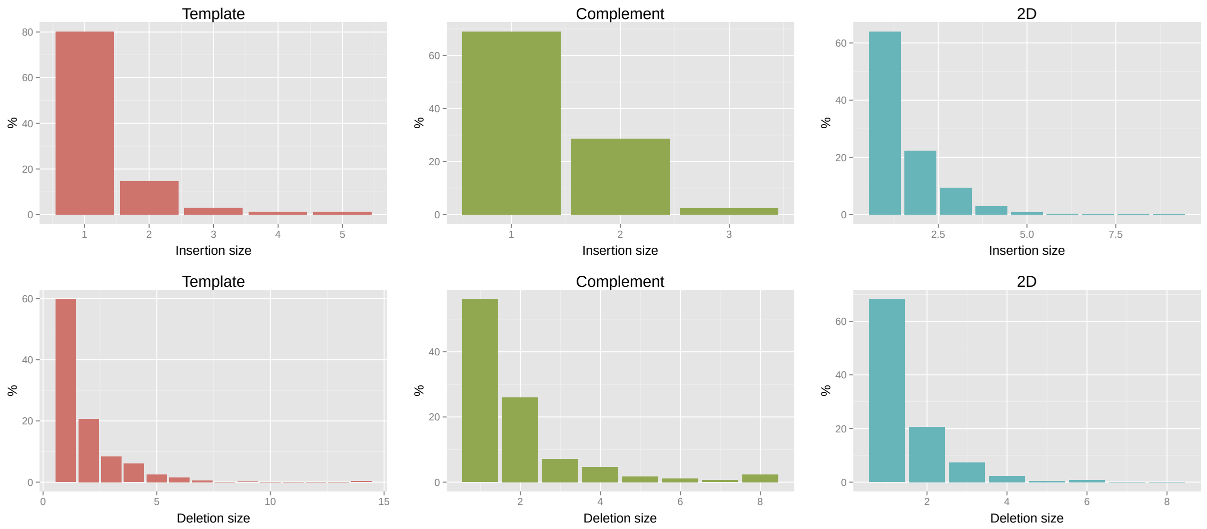

Actinomyces odontolyticus read identity

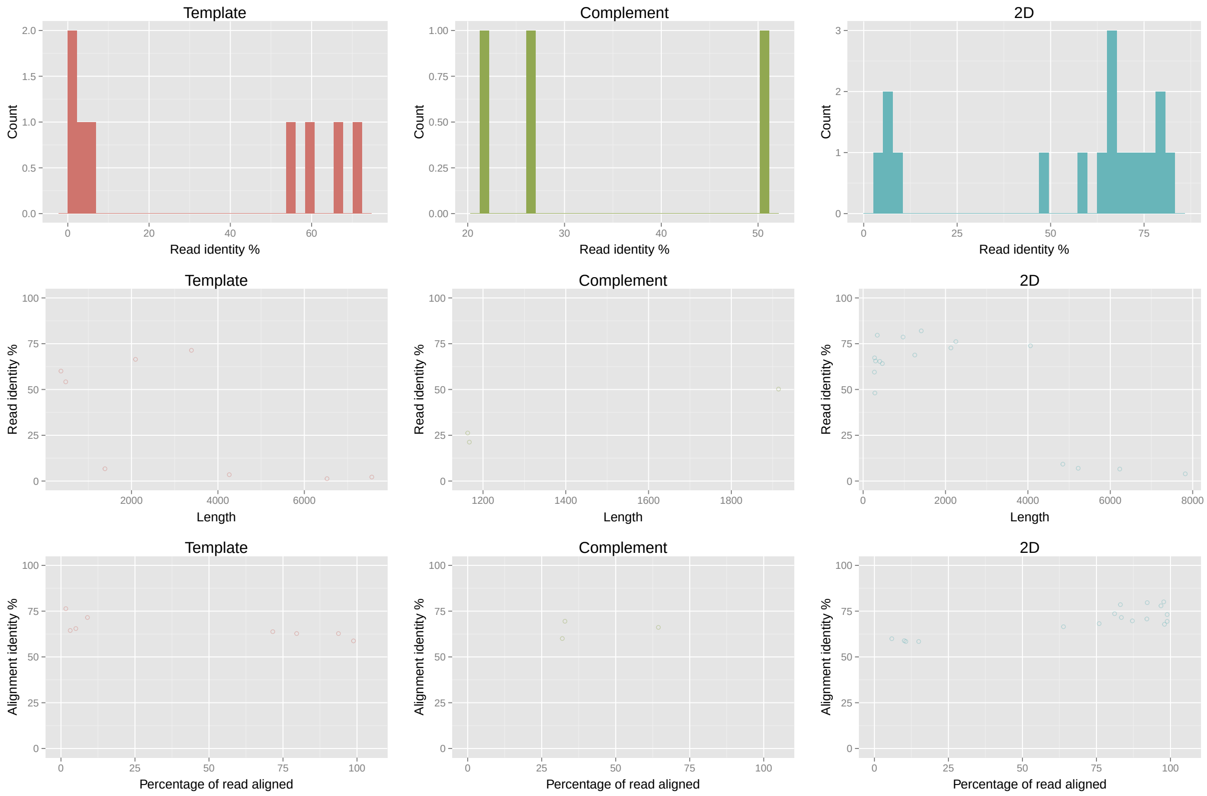

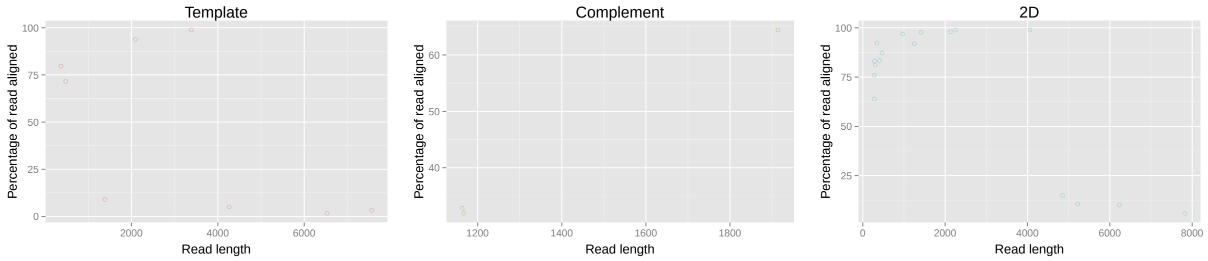

Actinomyces odontolyticus perfect kmers

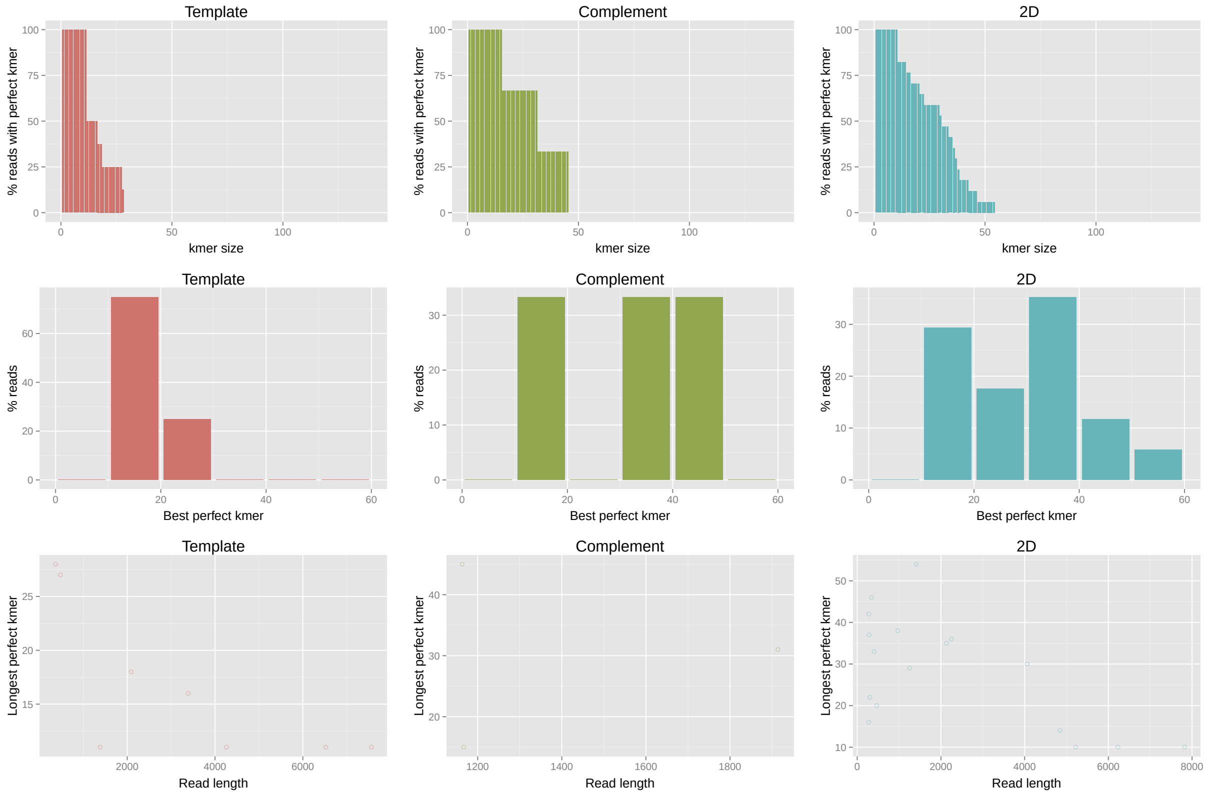

Actinomyces odontolyticus coverage

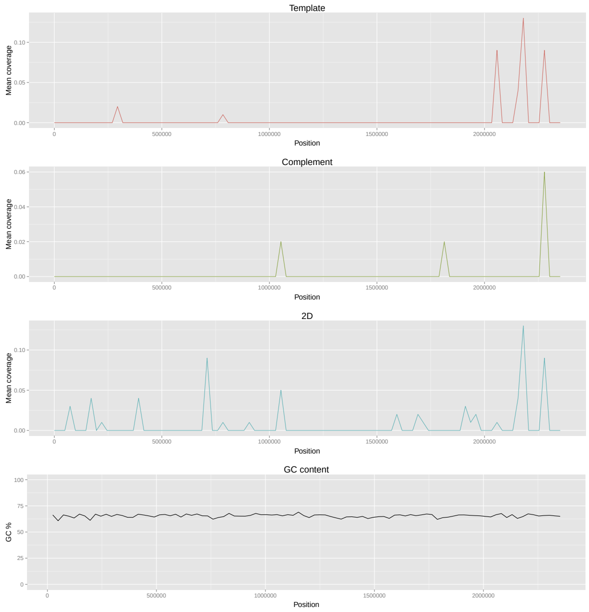

Actinomyces odontolyticus 5-mer analysis

Under-represented 5-mers

	Template				Complement				2D			
Rank	kmer	Ref %	Read %	Diff %	kmer	Ref %	Read %	Diff %	kmer	Ref %	Read %	Diff %
1	AGGTA	0.000	0.019	0.019	GGGAG	0.000	0.024	0.024	AAAAA	0.000	0.005	0.005
2	GTGTG	0.000	0.023	0.023	GGGAT	0.000	0.024	0.024	AAAAG	0.000	0.018	0.018
3	AAGGG	0.000	0.023	0.023	TACTT	0.000	0.024	0.024	AAATC	0.000	0.021	0.021
4	GGGTA	0.000	0.023	0.023	TCGAT	0.000	0.024	0.024	TAAAA	0.000	0.021	0.021
5	GGTGA	0.000	0.027	0.027	ACGGT	0.000	0.024	0.024	ATTTT	0.000	0.021	0.021
6	GAAGA	0.000	0.027	0.027	TTTTT	0.000	0.024	0.024	CAAAA	0.000	0.023	0.023
7	GGTAT	0.000	0.027	0.027	TTTTA	0.000	0.024	0.024	AATCG	0.000	0.023	0.023
8	GGACA	0.000	0.027	0.027	CAAAG	0.000	0.024	0.024	AATAA	0.000	0.023	0.023
9	GGACG	0.000	0.027	0.027	TTTTG	0.000	0.024	0.024	GTTAA	0.000	0.023	0.023
10	TAGGT	0.000	0.027	0.027	ATACA	0.000	0.024	0.024	AATTT	0.000	0.023	0.023

Over-represented 5-mers

	Template				Complement				2D			
Rank	kmer	Ref %	Read %	Diff %	kmer	Ref %	Read %	Diff %	kmer	Ref %	Read %	Diff %
1	CGACG	0.000	0.749	0.749	TCGGC	0.000	0.378	0.378	CGCCG	0.000	0.325	0.325
2	ACGAC	0.000	0.738	0.738	CGTCG	0.000	0.378	0.378	CGCGC	0.000	0.320	0.320
3	GACGA	0.000	0.722	0.722	CTCGG	0.000	0.355	0.355	GCGCG	0.000	0.312	0.312
4	CGGGC	0.000	0.281	0.281	GCTCG	0.000	0.331	0.331	CGGGC	0.000	0.307	0.307
5	CTGCT	0.000	0.269	0.269	CTGAT	0.000	0.331	0.331	GCCGC	0.000	0.304	0.304
6	CGGCT	0.000	0.265	0.265	CTGCG	0.000	0.331	0.331	GGCCG	0.000	0.302	0.302
7	CTTCT	0.000	0.257	0.257	GCTGA	0.000	0.307	0.307	CGGCG	0.000	0.302	0.302
8	CGGCG	0.000	0.257	0.257	CGGCG	0.000	0.307	0.307	GCGCC	0.000	0.289	0.289
9	GCGGC	0.000	0.254	0.254	GCGCA	0.000	0.284	0.284	GCCGG	0.000	0.286	0.286
10	TTCTT	0.000	0.242	0.242	CAGCC	0.000	0.284	0.284	GGCGG	0.000	0.278	0.278

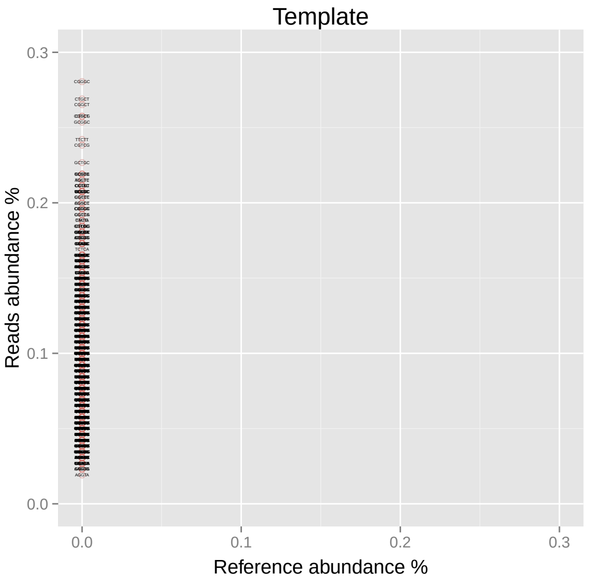

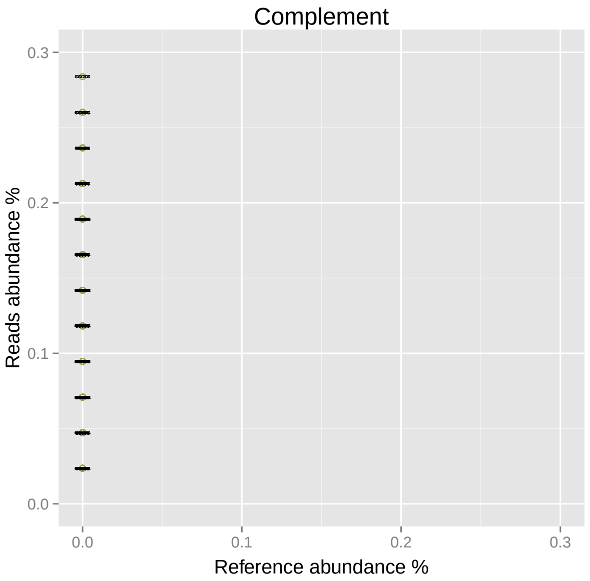

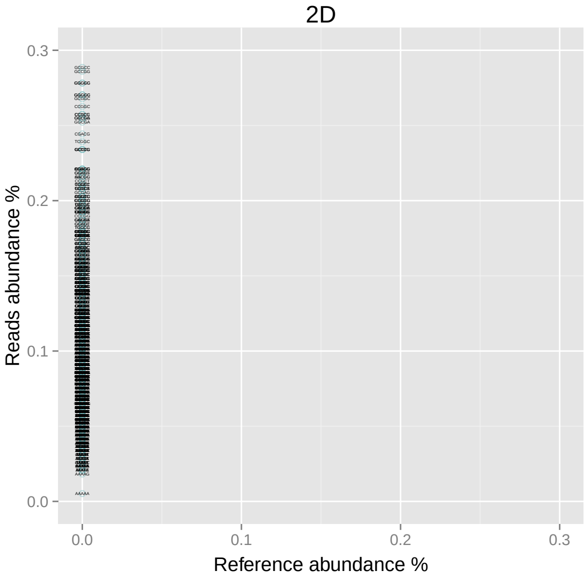

Actinomyces odontolyticus GC content

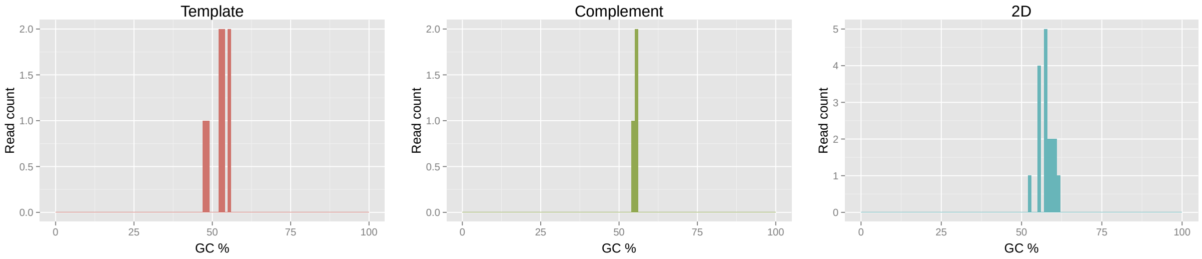

Bacillus cereus error analysis

	Template	Complement	2D
Overall base identity (excluding indels)	67.66%	70.37%	75.27%
Aligned base identity (excluding indels)	77.22%	79.75%	85.64%
Identical bases per 100 aligned bases (including indels)	64.16%	64.71%	75.28%
Inserted bases per 100 aligned bases (including indels)	3.25%	2.18%	6.19%
Deleted bases per 100 aligned bases (including indels)	13.66%	16.68%	5.91%
Substitutions per 100 aligned bases (including indels)	18.93%	16.43%	12.62%
Mean insertion size	1.44	1.34	1.54
Mean deletion size	1.73	1.86	1.44

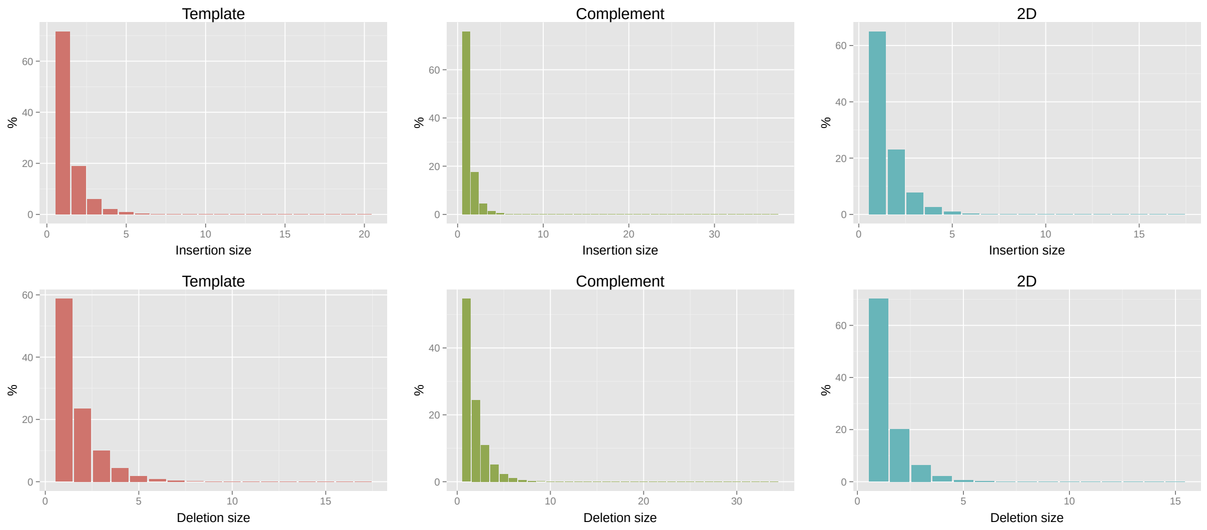

Bacillus cereus read identity

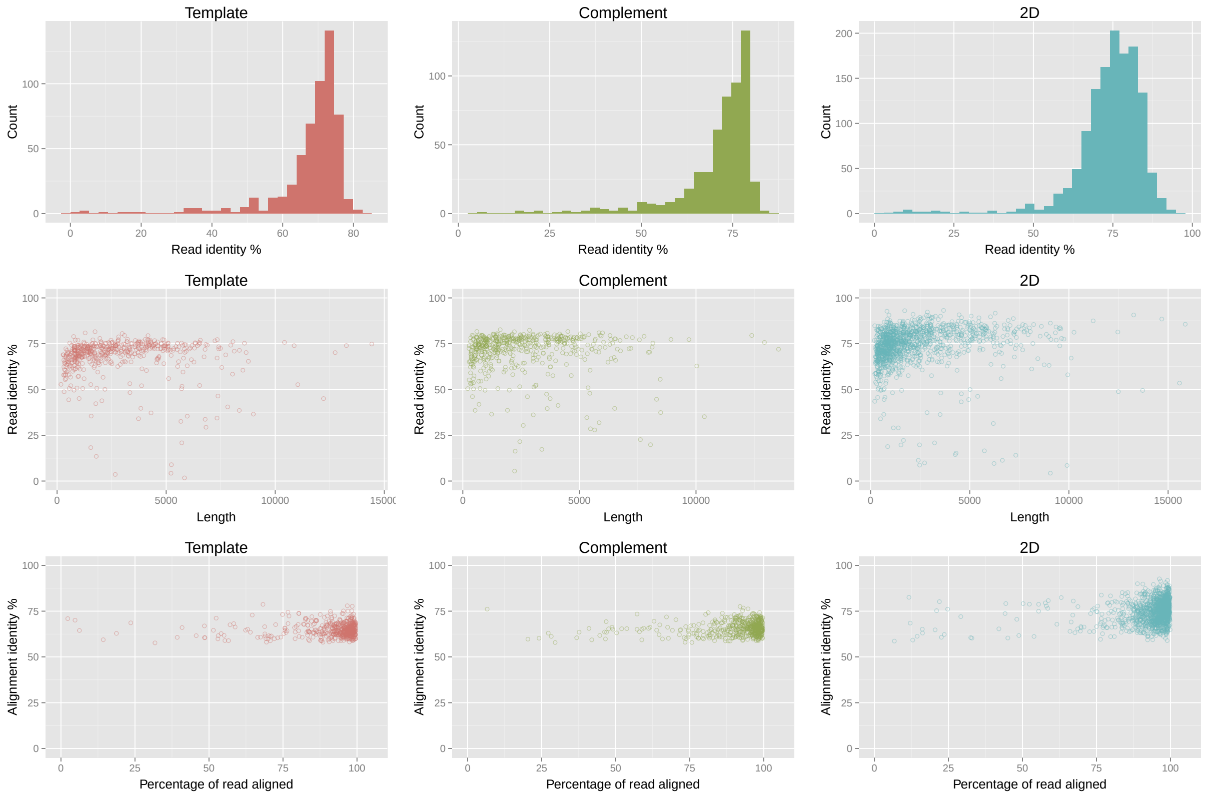

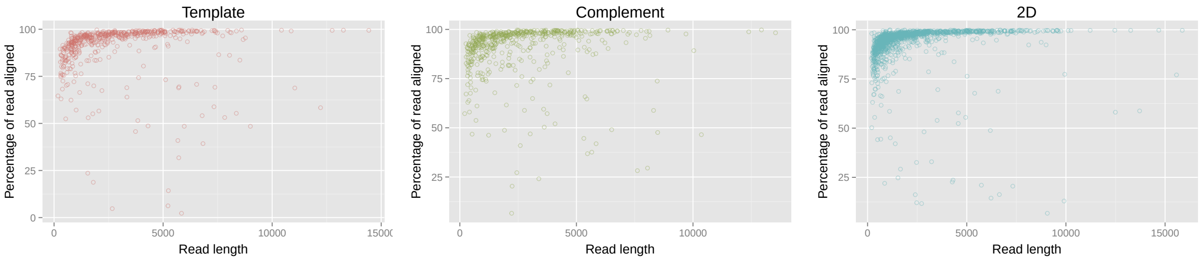

Bacillus cereus perfect kmers

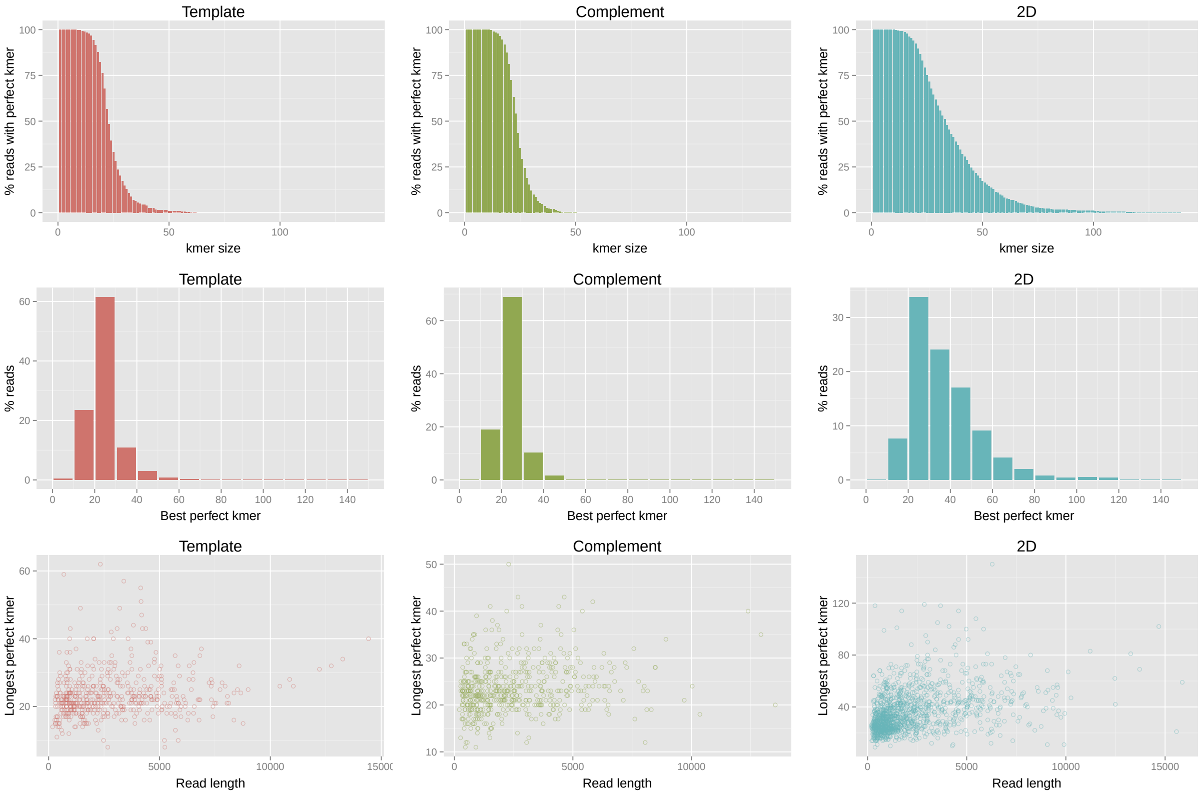

Bacillus cereus coverage

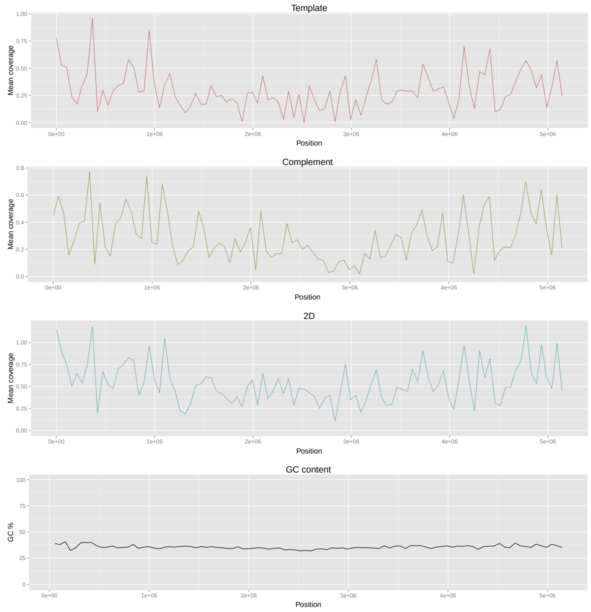

Bacillus cereus 5-mer analysis

Under-represented 5-mers

	Template				Complement				2D			
Rank	kmer	Ref %	Read %	Diff %	kmer	Ref %	Read %	Diff %	kmer	Ref %	Read %	Diff %
1	AAAAA	0.573	0.104	-0.470	TTTTT	0.578	0.098	-0.480	TTTTT	0.578	0.117	-0.461
2	TTTTT	0.578	0.120	-0.458	AAAAA	0.573	0.104	-0.470	AAAAA	0.573	0.140	-0.433
3	TAAAA	0.431	0.081	-0.351	TAAAA	0.431	0.124	-0.308	TAAAA	0.431	0.182	-0.250
4	AAAAT	0.454	0.146	-0.308	AAAAT	0.454	0.150	-0.304	TTTTA	0.426	0.180	-0.246
5	ATAAA	0.393	0.094	-0.300	ATTTT	0.452	0.162	-0.291	AAAAT	0.454	0.225	-0.229
6	AAAAG	0.354	0.097	-0.256	ATAAA	0.393	0.116	-0.278	ATTTT	0.452	0.224	-0.229
7	ATTTT	0.452	0.208	-0.245	TTTTC	0.362	0.101	-0.262	AAAAG	0.354	0.157	-0.197
8	TTTTA	0.426	0.185	-0.241	AAAAG	0.354	0.093	-0.261	CTTTT	0.352	0.168	-0.184
9	AAATA	0.402	0.168	-0.234	TTTTA	0.426	0.167	-0.259	TTTAT	0.393	0.215	-0.178
10	TTAAA	0.352	0.121	-0.231	AAATA	0.402	0.168	-0.234	TATTT	0.409	0.235	-0.174

Over-represented 5-mers

	Template				Complement				2D			
Rank	kmer	Ref %	Read %	Diff %	kmer	Ref %	Read %	Diff %	kmer	Ref %	Read %	Diff %
1	CGGGC	0.013	0.165	0.151	TCGGC	0.032	0.151	0.119	CGGGC	0.013	0.069	0.055
2	TCGTC	0.053	0.175	0.122	CGGCT	0.032	0.146	0.114	TCGAC	0.025	0.078	0.054
3	CGTCG	0.023	0.130	0.107	TCGTG	0.048	0.159	0.111	CGCAG	0.031	0.083	0.051
4	AGGCT	0.040	0.143	0.102	GTCGT	0.050	0.160	0.110	CATGC	0.056	0.106	0.050
5	GAGGA	0.072	0.169	0.097	CTTCG	0.070	0.175	0.105	CGACC	0.019	0.068	0.050
6	TAGGC	0.027	0.124	0.097	TCGTC	0.053	0.157	0.104	CGTAG	0.033	0.083	0.050
7	GTCGT	0.050	0.146	0.097	CGTAG	0.033	0.132	0.098	GGTTC	0.051	0.098	0.047
8	TCGTG	0.048	0.140	0.092	GCGTG	0.030	0.128	0.098	GCCTG	0.026	0.072	0.046
9	CGGCT	0.032	0.120	0.089	CGTCG	0.023	0.120	0.097	GCAGG	0.046	0.092	0.045
10	GGCTG	0.034	0.123	0.089	TCGTA	0.111	0.208	0.097	GGTCG	0.019	0.064	0.045

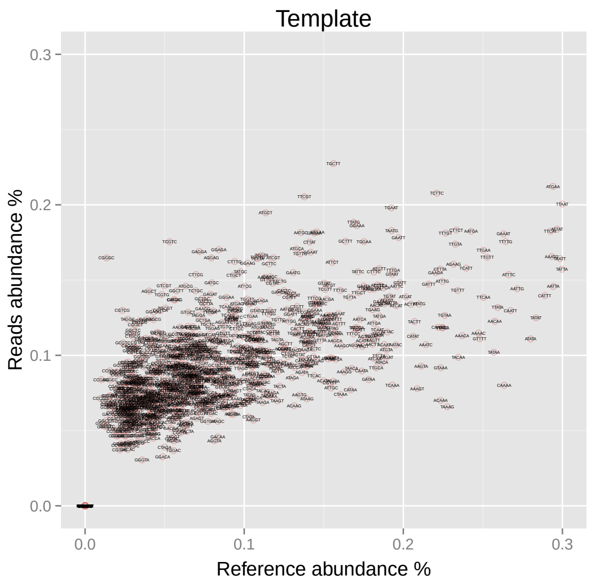

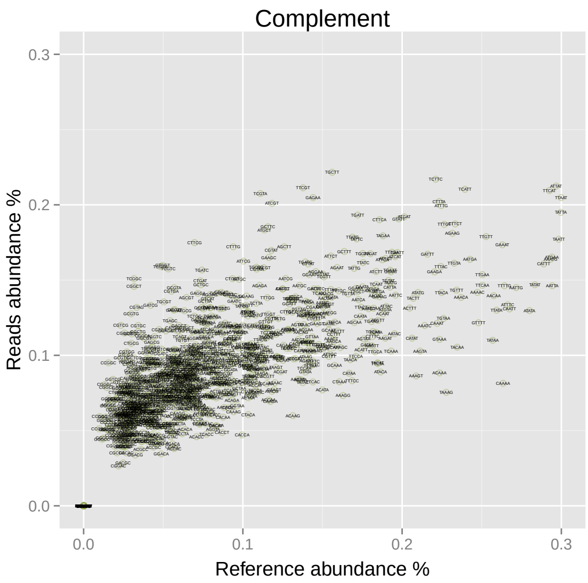

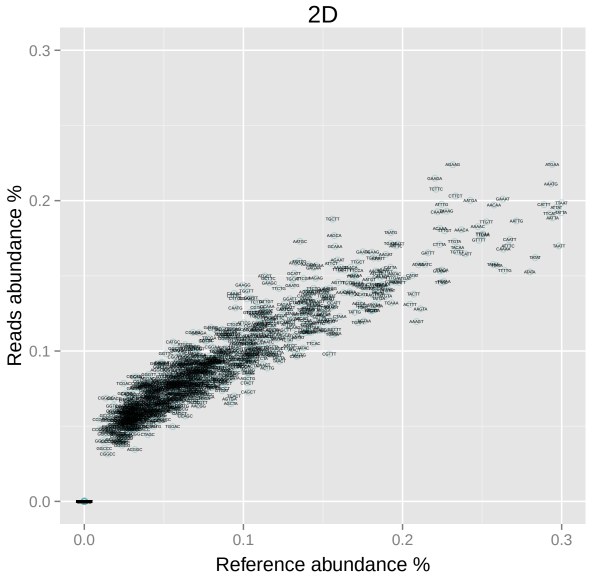

Bacillus cereus GC content

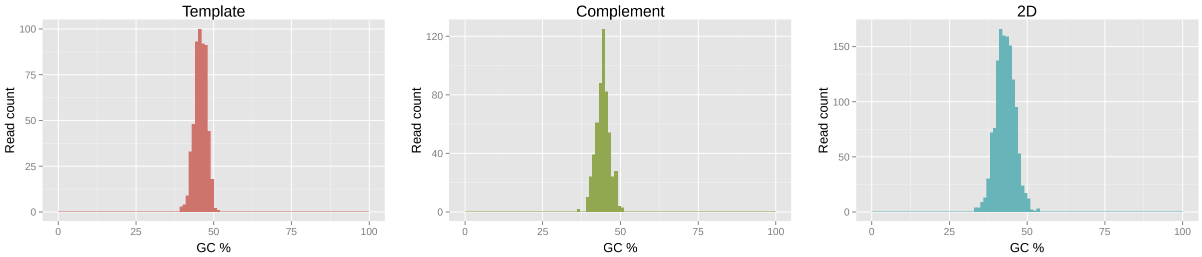

Bacteroides vulgatus error analysis

	Template	Complement	2D
Overall base identity (excluding indels)	74.68%	76.08%	77.23%
Aligned base identity (excluding indels)	78.05%	80.08%	85.73%
Identical bases per 100 aligned bases (including indels)	65.07%	65.41%	75.24%
Inserted bases per 100 aligned bases (including indels)	2.61%	2.34%	6.36%
Deleted bases per 100 aligned bases (including indels)	14.02%	15.98%	5.87%
Substitutions per 100 aligned bases (including indels)	18.30%	16.27%	12.53%
Mean insertion size	1.36	1.33	1.60
Mean deletion size	1.70	1.80	1.43

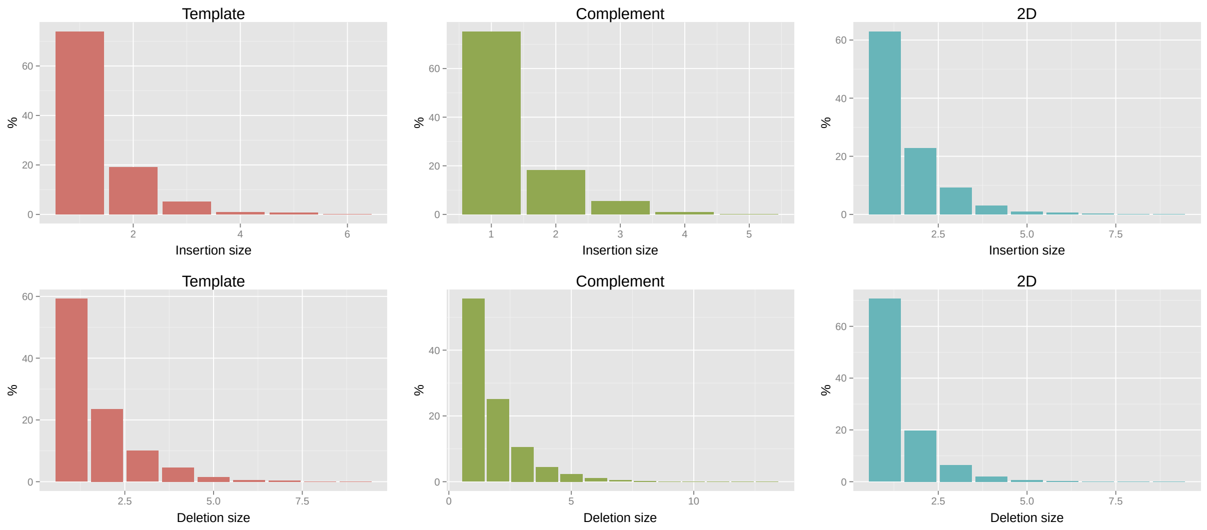

Bacteroides vulgatus read identity

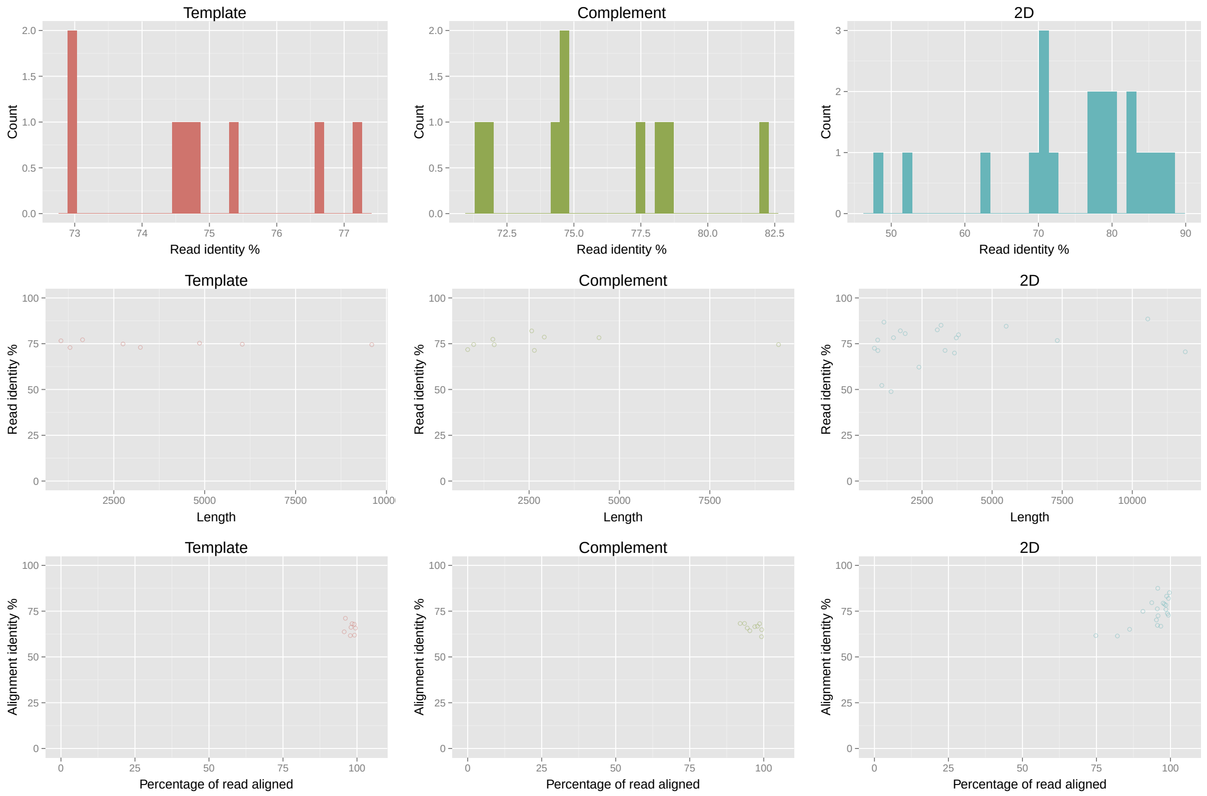

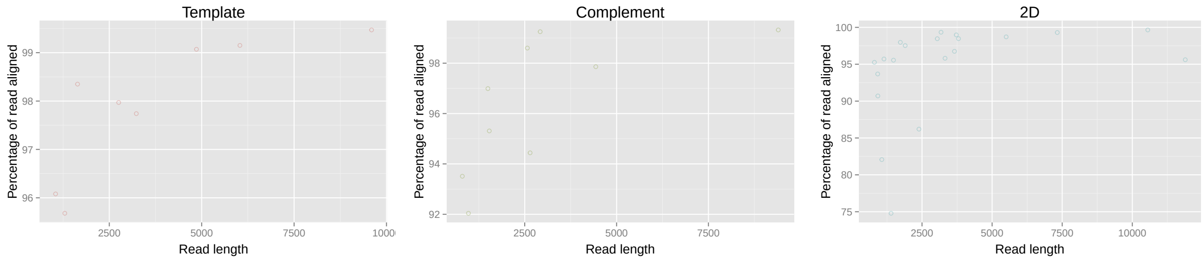

Bacteroides vulgatus perfect kmers

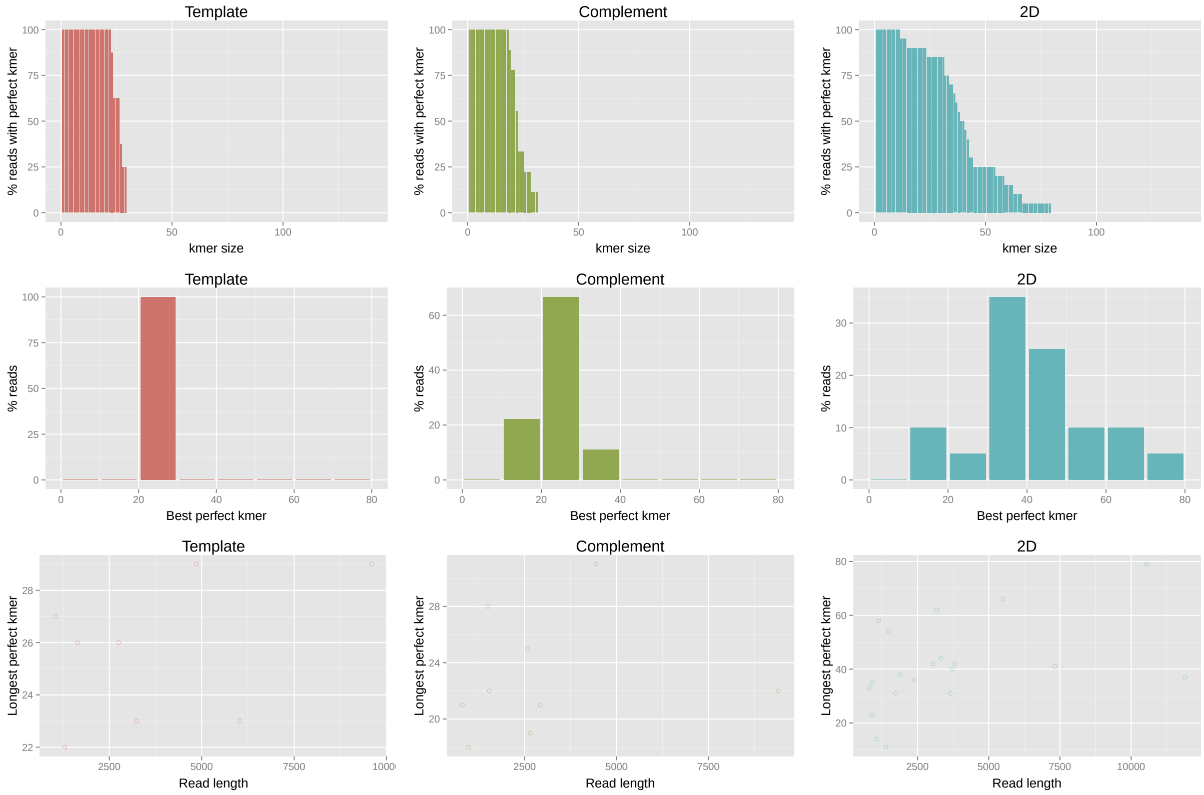

Bacteroides vulgatus coverage

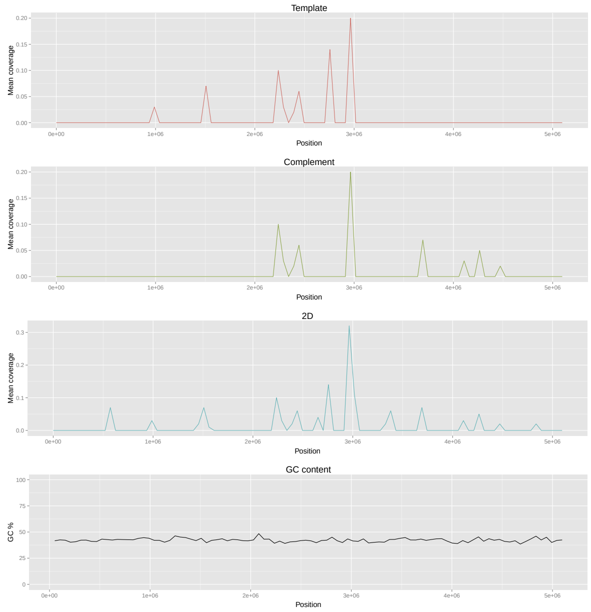

Bacteroides vulgatus 5-mer analysis

Under-represented 5-mers

	Template				Complement				2D			
Rank	kmer	Ref %	Read %	Diff %	kmer	Ref %	Read %	Diff %	kmer	Ref %	Read %	Diff %
1	AAAAA	0.352	0.046	-0.306	TTTTT	0.354	0.060	-0.294	TTTTT	0.354	0.078	-0.276
2	TTTTT	0.354	0.079	-0.275	AAAAA	0.352	0.127	-0.225	AAAAA	0.352	0.113	-0.239
3	AAGAA	0.284	0.092	-0.192	ATAAA	0.285	0.082	-0.203	AAAAG	0.253	0.101	-0.152
4	AAAGA	0.274	0.082	-0.191	TTTTC	0.281	0.086	-0.195	TTTAT	0.283	0.136	-0.146
5	AAAAT	0.278	0.092	-0.186	AAAGA	0.274	0.090	-0.184	CTTTT	0.258	0.125	-0.133
6	TTTTC	0.281	0.102	-0.179	TTTCA	0.261	0.094	-0.167	TTTTA	0.229	0.106	-0.123
7	AAAAG	0.253	0.076	-0.177	ATTTT	0.278	0.112	-0.166	GAAAA	0.274	0.161	-0.114
8	ATAAA	0.285	0.118	-0.167	AAAAT	0.278	0.116	-0.162	TGAAA	0.261	0.149	-0.112
9	ATTTT	0.278	0.115	-0.163	AAAAG	0.253	0.094	-0.159	TAAAA	0.231	0.121	-0.110
10	GAAAA	0.274	0.115	-0.159	TTTCT	0.246	0.094	-0.153	AAAGA	0.274	0.165	-0.108

Over-represented 5-mers

	Template				Complement				2D			
Rank	kmer	Ref %	Read %	Diff %	kmer	Ref %	Read %	Diff %	kmer	Ref %	Read %	Diff %
1	TTCGT	0.088	0.204	0.116	TGATC	0.063	0.195	0.132	TCGAC	0.026	0.096	0.070
2	CGGGC	0.055	0.171	0.116	GCGTA	0.059	0.187	0.128	GTTAG	0.028	0.085	0.057
3	TCGTG	0.047	0.155	0.108	TCGTG	0.047	0.146	0.099	TAACA	0.083	0.136	0.053
4	ATCGT	0.076	0.184	0.108	ATCGA	0.048	0.146	0.098	TAGAT	0.069	0.122	0.053
5	CTTAG	0.026	0.122	0.096	AACGT	0.072	0.168	0.096	AGTCG	0.039	0.090	0.052
6	CATGC	0.081	0.174	0.094	CTGCT	0.110	0.202	0.093	CTTAG	0.026	0.076	0.050
7	CGTGC	0.054	0.145	0.090	GCGTG	0.047	0.138	0.091	GCATG	0.077	0.126	0.049
8	CTTCG	0.080	0.168	0.088	CGCGT	0.026	0.116	0.090	TTCGA	0.061	0.109	0.048
9	CGTCG	0.024	0.112	0.088	CGTAA	0.078	0.168	0.090	CACGA	0.049	0.098	0.048
10	CGACT	0.038	0.125	0.087	TGCTT	0.146	0.236	0.089	TTAGA	0.047	0.095	0.048

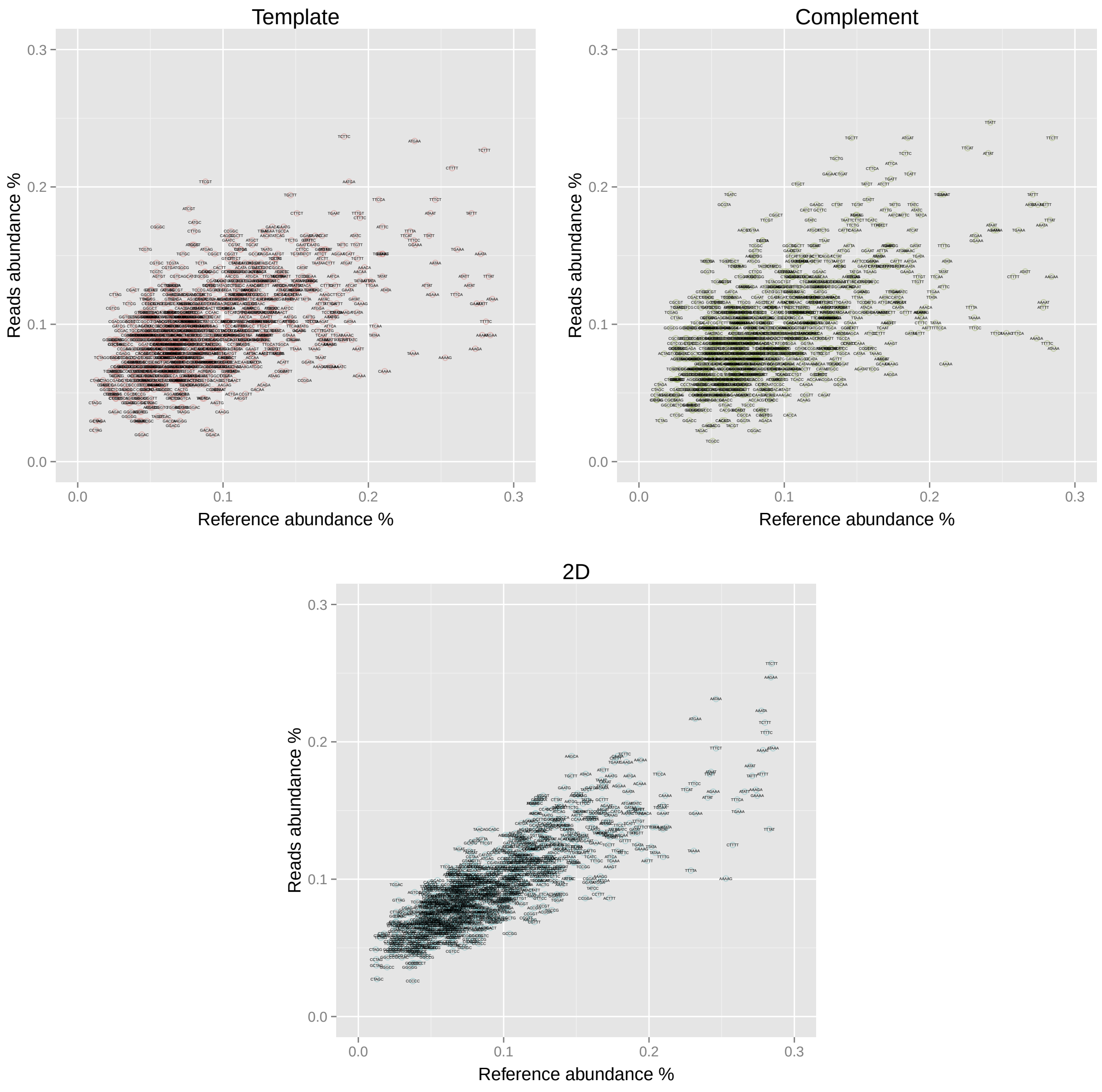

Bacteroides vulgatus GC content

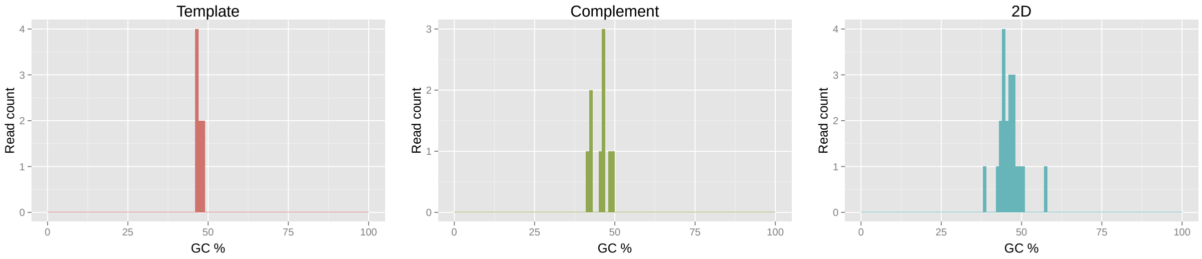

Clostridium beijerinckii error analysis

	Template	Complement	2D
Overall base identity (excluding indels)	64.04%	68.03%	71.39%
Aligned base identity (excluding indels)	76.59%	79.88%	82.95%
Identical bases per 100 aligned bases (including indels)	63.79%	65.36%	72.46%
Inserted bases per 100 aligned bases (including indels)	3.17%	2.20%	6.58%
Deleted bases per 100 aligned bases (including indels)	13.54%	15.98%	6.06%
Substitutions per 100 aligned bases (including indels)	19.49%	16.46%	14.90%
Mean insertion size	1.44	1.36	1.54
Mean deletion size	1.77	1.87	1.47

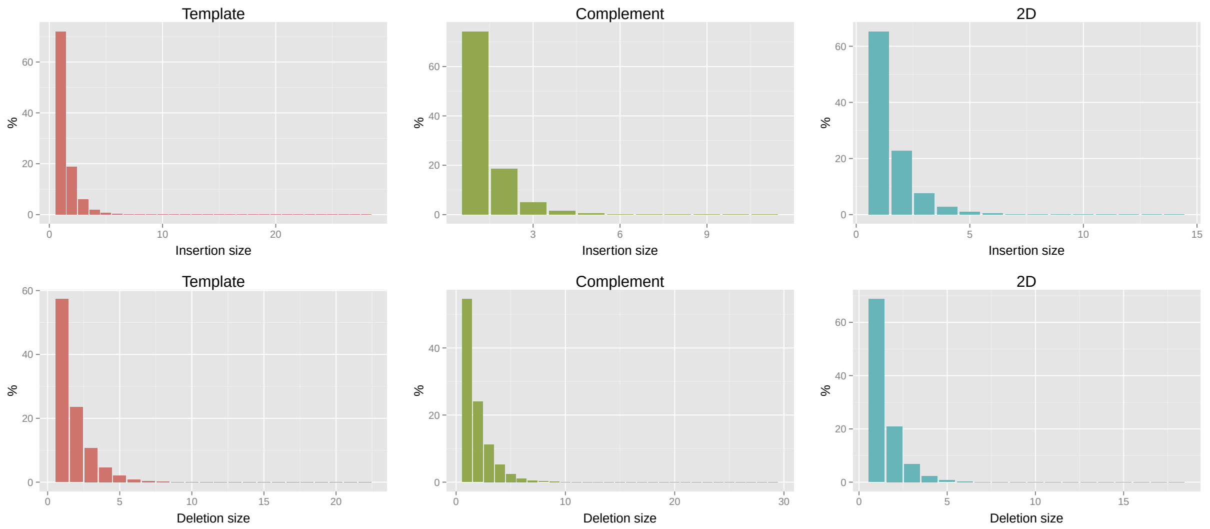

Clostridium beijerinckii read identity

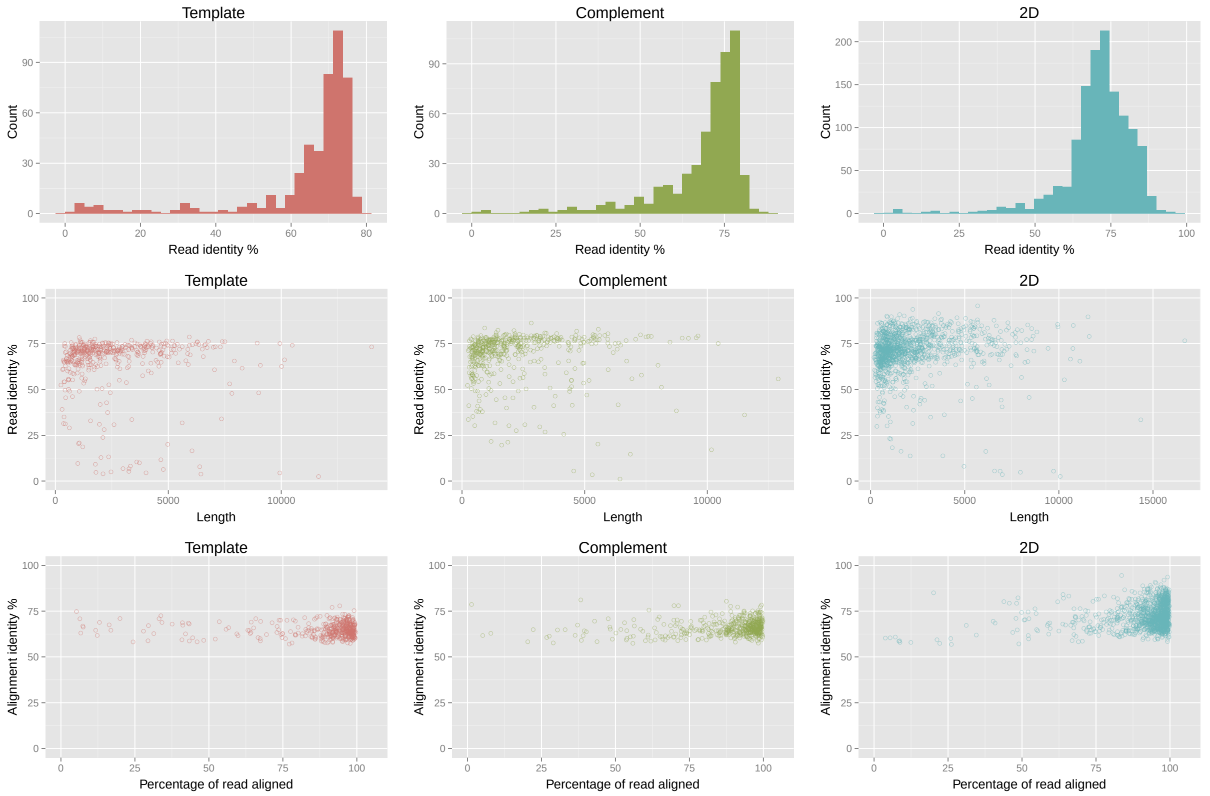

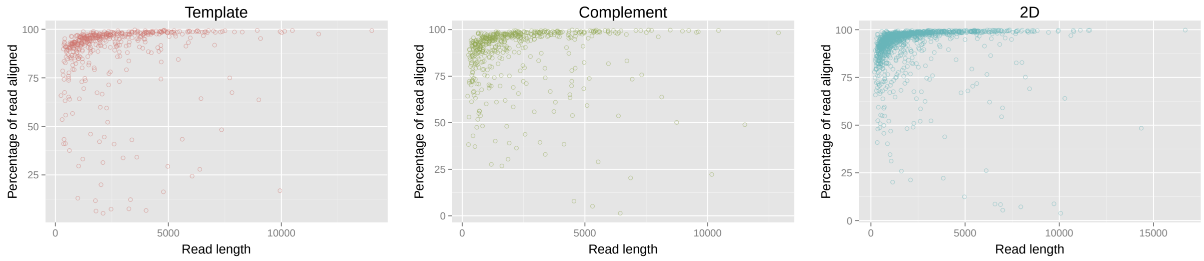

Clostridium beijerinckii perfect kmers

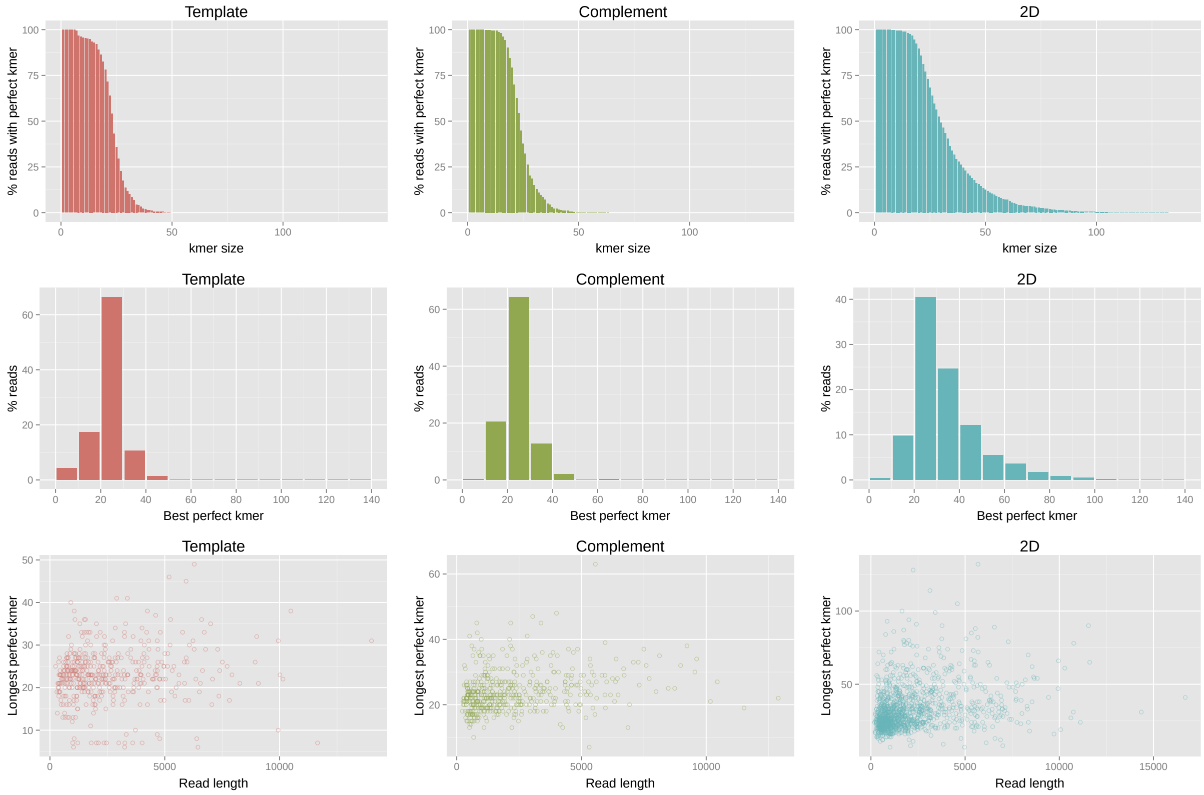

Clostridium beijerinckii coverage

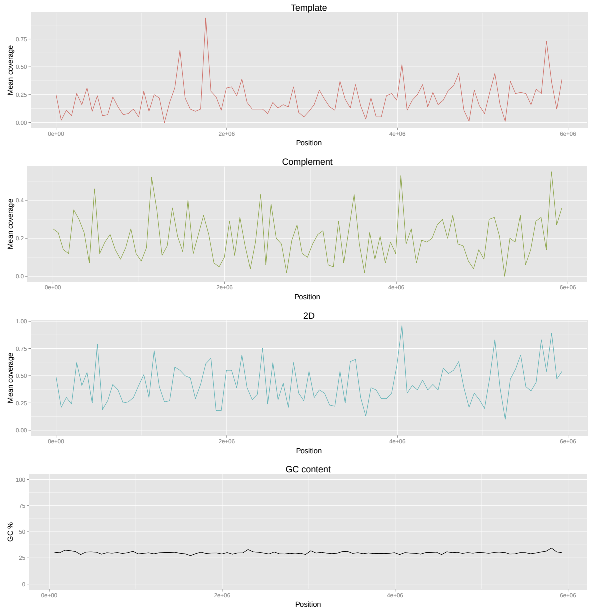

Clostridium beijerinckii 5-mer analysis

Under-represented 5-mers

	Template				Complement				2D			
Rank	kmer	Ref %	Read %	Diff %	kmer	Ref %	Read %	Diff %	kmer	Ref %	Read %	Diff %
1	AAAAA	0.647	0.113	-0.534	AAAAA	0.647	0.117	-0.530	AAAAA	0.647	0.131	-0.517
2	TTTTT	0.611	0.123	-0.488	TTTTT	0.611	0.092	-0.519	TTTTT	0.611	0.117	-0.494
3	TAAAA	0.586	0.112	-0.474	ATTTT	0.604	0.173	-0.431	TAAAA	0.586	0.209	-0.377
4	ATAAA	0.592	0.136	-0.456	AAAAT	0.622	0.202	-0.419	AAAAT	0.622	0.253	-0.369
5	AAAAT	0.622	0.196	-0.426	ATAAA	0.592	0.176	-0.416	TTTTA	0.566	0.218	-0.348
6	ATATT	0.593	0.168	-0.425	TAAAA	0.586	0.171	-0.415	ATTTT	0.604	0.269	-0.335
7	TATTT	0.593	0.213	-0.381	TTTTA	0.566	0.179	-0.386	ATATA	0.556	0.222	-0.335
8	AAATA	0.618	0.238	-0.380	AAATA	0.618	0.235	-0.383	AATAT	0.597	0.277	-0.320
9	AATAT	0.597	0.224	-0.373	TTAAA	0.531	0.178	-0.353	TTTAT	0.572	0.255	-0.317
10	TATAA	0.521	0.154	-0.368	AAATT	0.525	0.173	-0.352	ATAAA	0.592	0.277	-0.315

Over-represented 5-mers

	Template				Complement				2D			
Rank	kmer	Ref %	Read %	Diff %	kmer	Ref %	Read %	Diff %	kmer	Ref %	Read %	Diff %
1	CGGGC	0.004	0.153	0.149	TCGGC	0.011	0.146	0.136	TCGAC	0.009	0.078	0.069
2	ATGCG	0.027	0.143	0.117	CGGCT	0.015	0.144	0.129	CGACC	0.005	0.074	0.069
3	CGGCT	0.015	0.127	0.112	TCGTA	0.032	0.159	0.127	ATGCG	0.027	0.095	0.069
4	GGCTC	0.021	0.130	0.108	ATCGT	0.034	0.144	0.110	CGCAG	0.014	0.081	0.067
5	GAGGA	0.079	0.186	0.107	GCGTA	0.020	0.127	0.107	CGGGC	0.004	0.070	0.066
6	CGGAA	0.033	0.139	0.106	CGTAG	0.015	0.120	0.105	CATGC	0.044	0.108	0.065
7	TAGGC	0.035	0.139	0.104	CGTAA	0.036	0.139	0.103	CGATT	0.037	0.101	0.064
8	GGGAG	0.028	0.127	0.100	ATCGG	0.019	0.121	0.102	CGACT	0.015	0.079	0.064
9	TCGTG	0.014	0.114	0.099	TCGTG	0.014	0.115	0.101	TGCCG	0.015	0.079	0.064
10	GAACG	0.017	0.116	0.099	CTTCG	0.026	0.127	0.101	CACGA	0.015	0.078	0.063

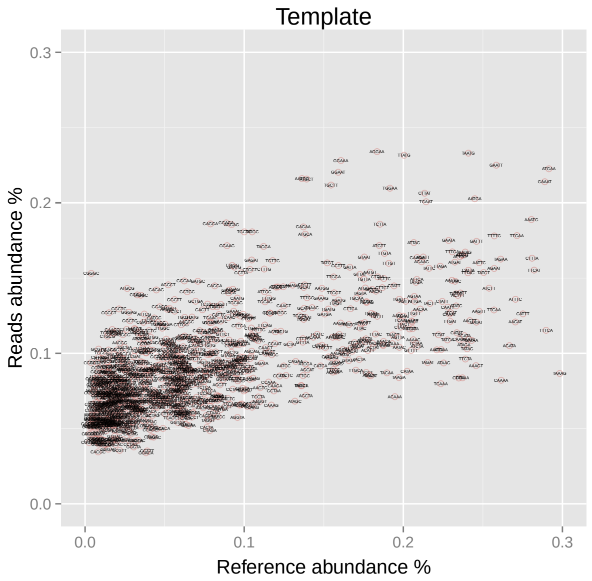

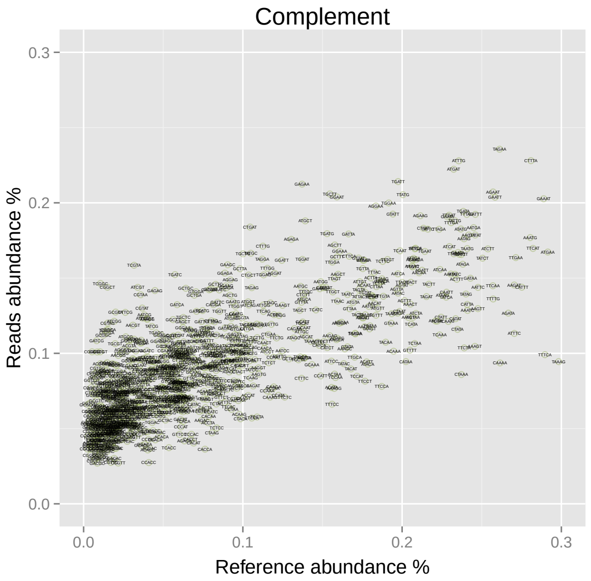

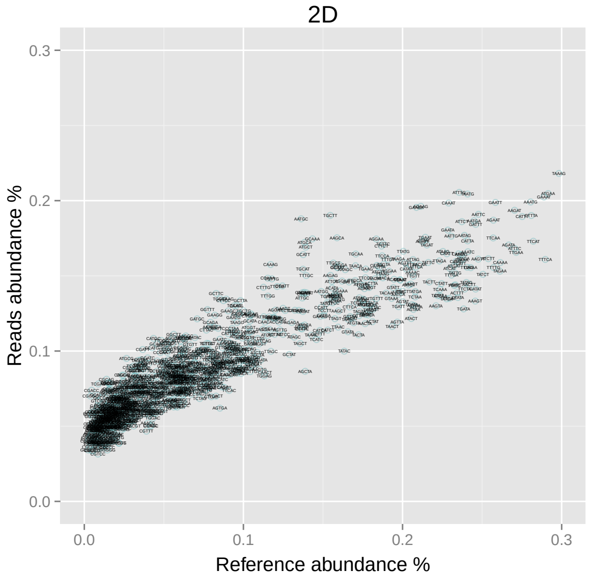

Clostridium beijerinckii GC content

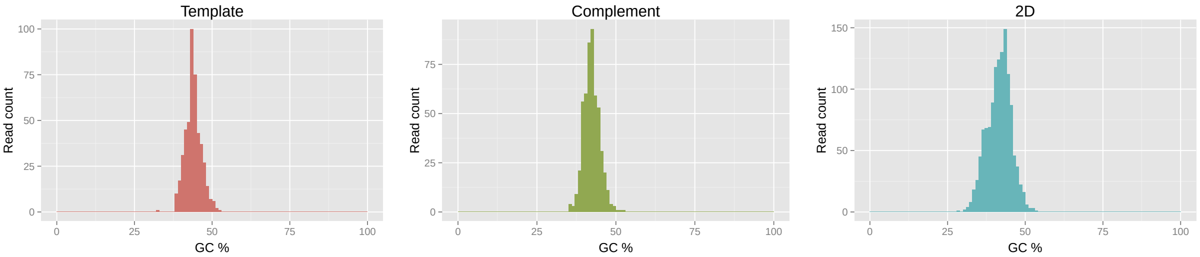

Control sequence error analysis

	Template	Complement	2D
Overall base identity (excluding indels)	33.70%	29.21%	43.16%
Aligned base identity (excluding indels)	76.35%	80.50%	74.72%
Identical bases per 100 aligned bases (including indels)	61.71%	65.60%	62.25%
Inserted bases per 100 aligned bases (including indels)	5.12%	3.15%	7.27%
Deleted bases per 100 aligned bases (including indels)	14.05%	15.37%	9.41%
Substitutions per 100 aligned bases (including indels)	19.12%	15.89%	21.07%
Mean insertion size	1.83	1.54	1.59
Mean deletion size	1.72	1.87	1.61

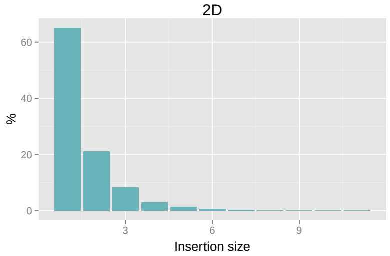

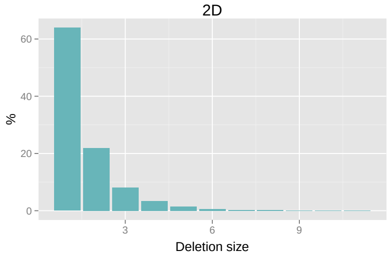

Control sequence read identity

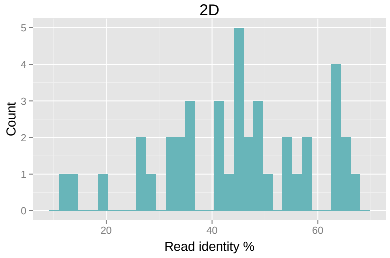

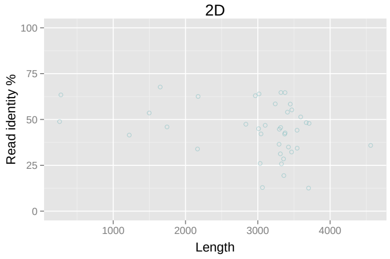

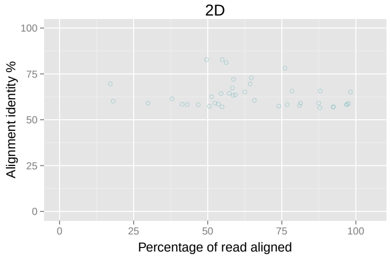

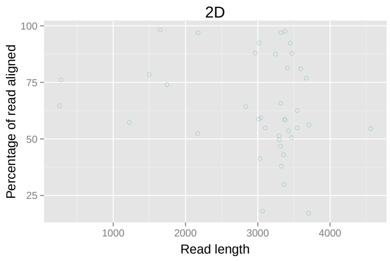

Control sequence perfect kmers

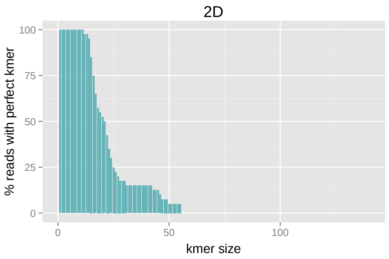

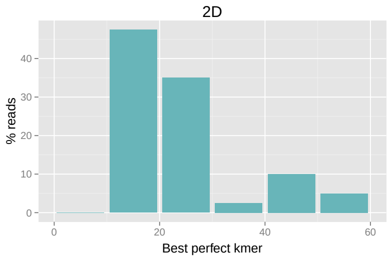

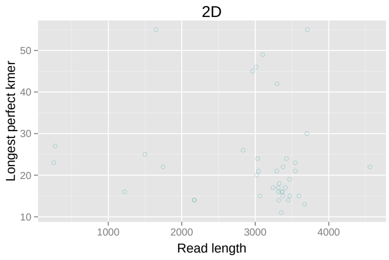

Control sequence coverage

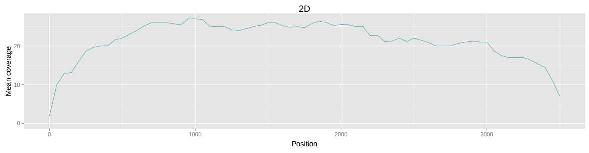

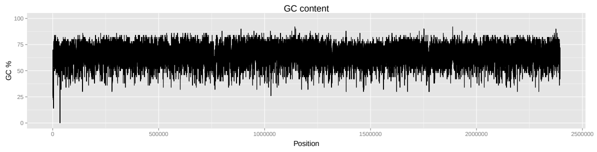

Control sequence 5-mer analysis

Under-represented 5-mers

	Template				Complement				2D			
Rank	kmer	Ref %	Read %	Diff %	kmer	Ref %	Read %	Diff %	kmer	Ref %	Read %	Diff %
1	CGCGC	0.577	0.106	-0.471	CGCGC	0.577	0.119	-0.458	CGCGC	0.577	0.081	-0.496
2	GCGCG	0.568	0.126	-0.441	GCGCG	0.568	0.119	-0.448	GCGCG	0.568	0.086	-0.482
3	CGACG	0.501	0.078	-0.423	CGACG	0.501	0.060	-0.441	CGACG	0.501	0.052	-0.448
4	CCTCG	0.463	0.055	-0.408	CGCCG	0.492	0.072	-0.420	CGGCG	0.518	0.090	-0.428
5	CGCCG	0.492	0.103	-0.389	CCTCG	0.463	0.066	-0.397	CGTCG	0.468	0.077	-0.391
6	CGAGG	0.462	0.101	-0.362	CGAGG	0.462	0.090	-0.372	CGCCG	0.492	0.113	-0.379
7	CGGCG	0.518	0.178	-0.340	CGTCG	0.468	0.125	-0.342	CCTCG	0.463	0.112	-0.351
8	CCGCG	0.386	0.066	-0.320	CGGCG	0.518	0.185	-0.333	CGAGG	0.462	0.131	-0.331
9	GCCGC	0.372	0.069	-0.303	CGCGG	0.399	0.078	-0.321	CGCGG	0.399	0.081	-0.318
10	GGCCG	0.355	0.060	-0.294	GGCGC	0.394	0.084	-0.310	CGGCC	0.361	0.068	-0.292

Over-represented 5-mers

	Template				Complement				2D			
Rank	kmer	Ref %	Read %	Diff %	kmer	Ref %	Read %	Diff %	kmer	Ref %	Read %	Diff %
1	ATAAT	0.012	0.187	0.175	ATGAT	0.085	0.275	0.189	TAACA	0.012	0.161	0.149
2	TTAAT	0.009	0.181	0.172	TGATT	0.027	0.209	0.182	AATAA	0.013	0.158	0.145
3	TGCTT	0.049	0.218	0.169	ATTTG	0.014	0.185	0.172	TTGTA	0.028	0.172	0.144
4	TGTAT	0.014	0.172	0.159	TTATT	0.011	0.179	0.168	TAATG	0.015	0.154	0.139
5	TAATG	0.015	0.172	0.157	CTTTA	0.011	0.173	0.162	ATAAT	0.012	0.150	0.139
6	TAATA	0.005	0.161	0.155	TGATA	0.020	0.179	0.159	ATTTG	0.014	0.151	0.138
7	ATTTT	0.017	0.172	0.155	TAGCT	0.017	0.173	0.157	ATTAA	0.009	0.144	0.135
8	TTTTG	0.024	0.178	0.154	GATTT	0.029	0.185	0.156	TGTTT	0.025	0.155	0.130
9	CTTAT	0.014	0.158	0.144	TACTA	0.012	0.167	0.156	CATTA	0.016	0.144	0.129
10	TAATT	0.006	0.146	0.141	TTCTG	0.058	0.209	0.151	AAACA	0.026	0.152	0.126

Control sequence GC content

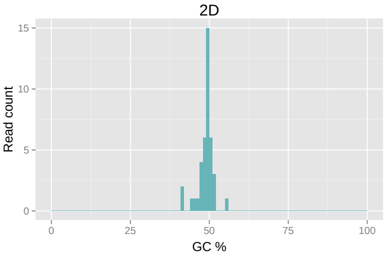

Deinococcus radiodurans 1 error analysis

	Template	Complement	2D
Overall base identity (excluding indels)	51.06%	58.51%	55.28%
Aligned base identity (excluding indels)	73.94%	79.36%	83.59%
Identical bases per 100 aligned bases (including indels)	61.32%	64.83%	72.97%
Inserted bases per 100 aligned bases (including indels)	4.00%	2.68%	7.02%
Deleted bases per 100 aligned bases (including indels)	13.07%	15.62%	5.68%
Substitutions per 100 aligned bases (including indels)	21.61%	16.87%	14.33%
Mean insertion size	1.44	1.41	1.60
Mean deletion size	1.67	1.85	1.43

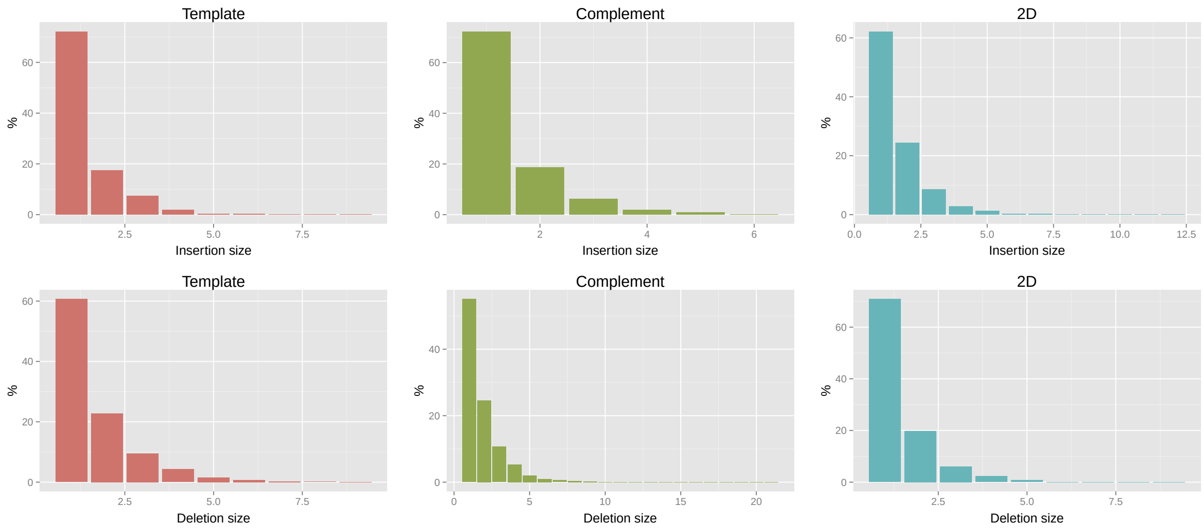

Deinococcus radiodurans 1 read identity

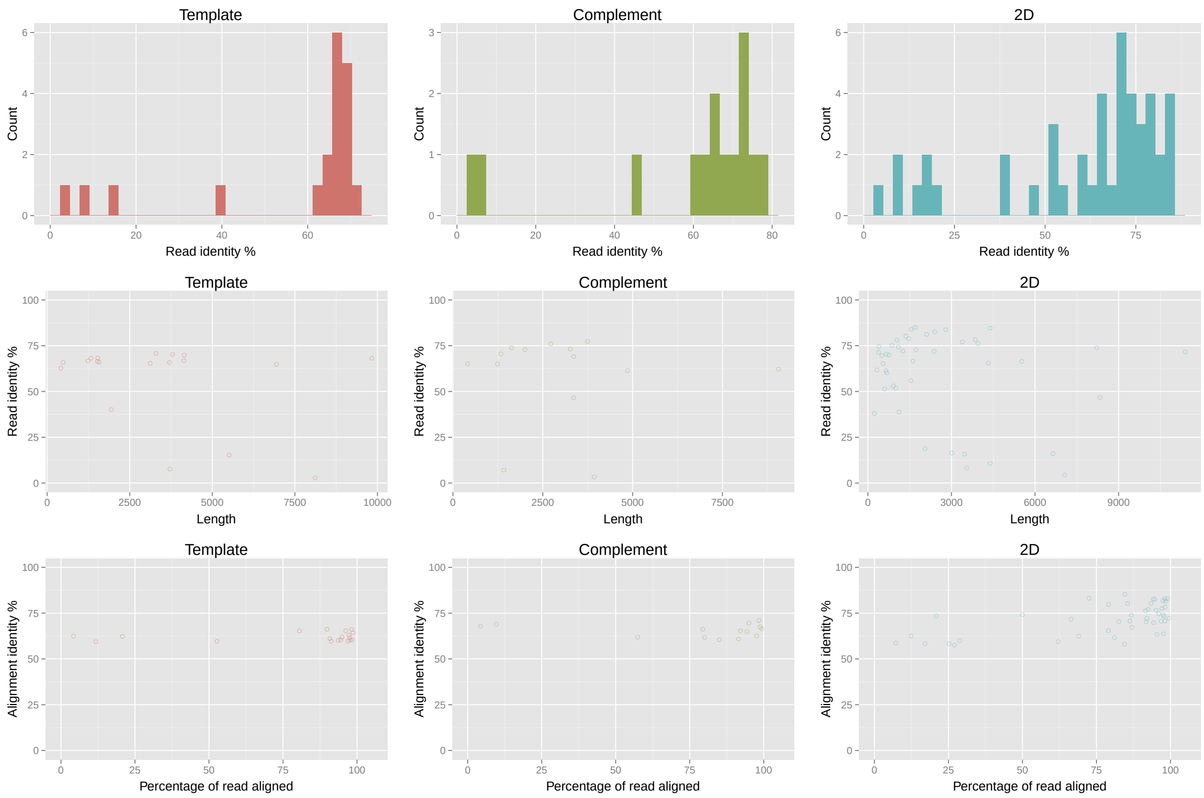

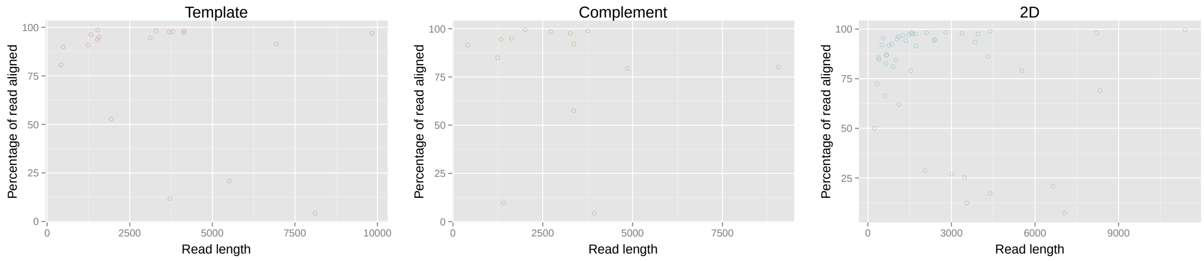

Deinococcus radiodurans 1 perfect kmers

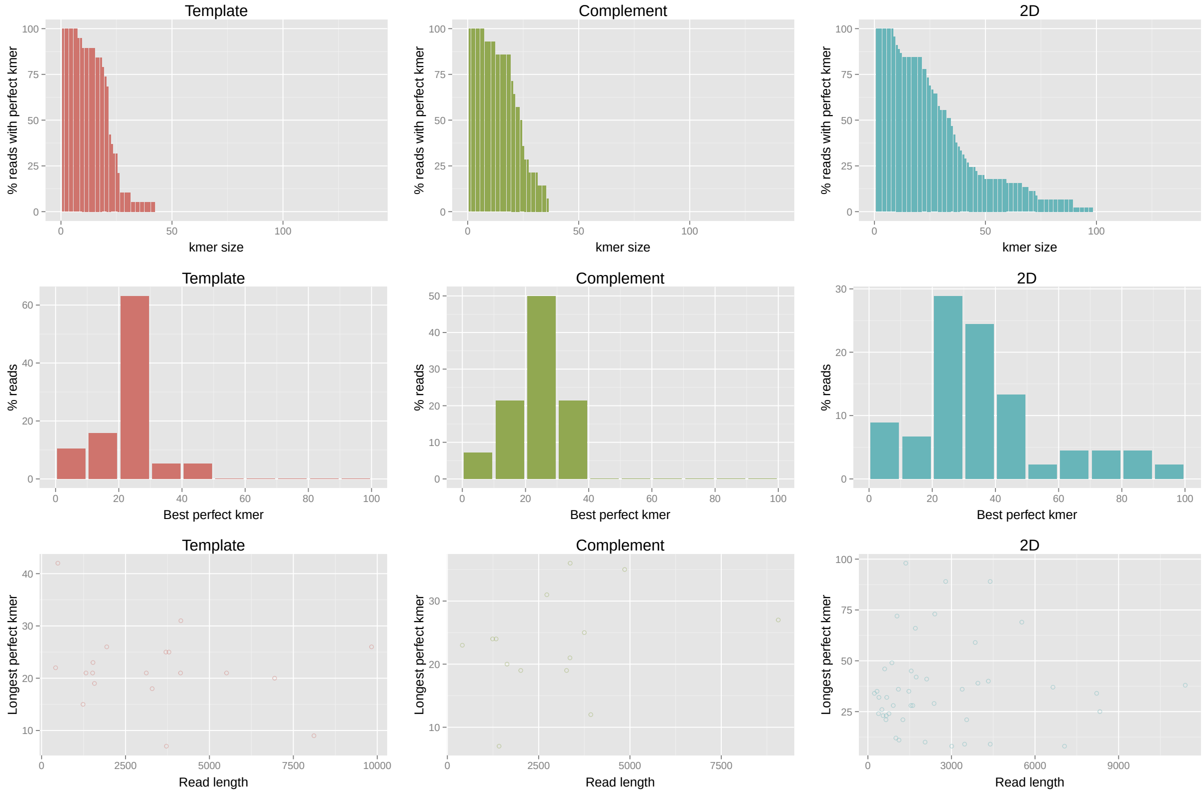

Deinococcus radiodurans 1 coverage

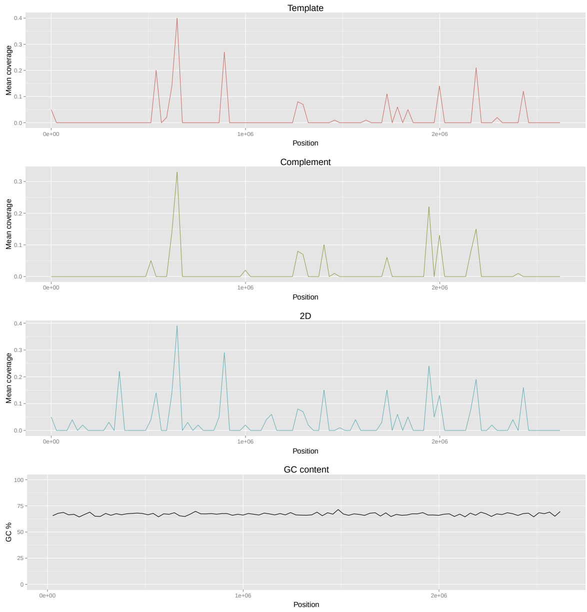

Deinococcus radiodurans 1 5-mer analysis

Under-represented 5-mers

	Template				Complement				2D			
Rank	kmer	Ref %	Read %	Diff %	kmer	Ref %	Read %	Diff %	kmer	Ref %	Read %	Diff %
1	CGCCG	0.734	0.136	-0.598	CGCCG	0.734	0.218	-0.516	CGCCG	0.734	0.335	-0.399
2	GCCGC	0.640	0.119	-0.520	GCCGC	0.640	0.201	-0.439	GCCGC	0.640	0.274	-0.366
3	CGGCG	0.743	0.294	-0.449	GCGCC	0.548	0.151	-0.397	CGGCG	0.743	0.440	-0.303
4	CGCGC	0.493	0.101	-0.392	GGCGC	0.556	0.163	-0.393	CCGCC	0.546	0.253	-0.293
5	GCGCG	0.493	0.118	-0.375	GGGCG	0.485	0.149	-0.336	GCGCC	0.548	0.271	-0.277
6	GGCGC	0.556	0.199	-0.357	CGGCG	0.743	0.416	-0.327	CCCGC	0.443	0.195	-0.248
7	GCGCC	0.548	0.202	-0.346	CGGGC	0.465	0.142	-0.324	GCGGC	0.642	0.396	-0.246
8	GCGGC	0.642	0.308	-0.334	GCGGG	0.441	0.123	-0.318	CGCCC	0.489	0.245	-0.243
9	CCGCC	0.546	0.220	-0.325	CGCGC	0.493	0.177	-0.316	CCGCG	0.402	0.162	-0.240
10	CGCTG	0.417	0.100	-0.318	CGCTG	0.417	0.121	-0.297	GGCGC	0.556	0.324	-0.233

Over-represented 5-mers

	Template				Complement				2D			
Rank	kmer	Ref %	Read %	Diff %	kmer	Ref %	Read %	Diff %	kmer	Ref %	Read %	Diff %
1	CACAC	0.056	0.222	0.166	CCTAC	0.070	0.265	0.195	CATAG	0.021	0.127	0.107
2	CTTAT	0.012	0.165	0.152	CACAC	0.056	0.234	0.178	ATAGC	0.016	0.118	0.102
3	ACACA	0.015	0.162	0.146	CTACC	0.069	0.215	0.146	CCATA	0.017	0.117	0.101
4	CCTAC	0.070	0.214	0.145	ACCTA	0.025	0.170	0.146	GCTAG	0.011	0.111	0.100
5	GCTTA	0.022	0.165	0.143	ACCAC	0.137	0.281	0.144	GGCTA	0.040	0.139	0.100
6	GGCTA	0.040	0.181	0.142	CGGCT	0.221	0.364	0.143	TAGCC	0.037	0.136	0.098
7	TGCTT	0.052	0.192	0.139	ACACG	0.066	0.196	0.131	TAGGG	0.026	0.120	0.094
8	TAGGC	0.042	0.180	0.137	ACACA	0.015	0.137	0.122	CTAGC	0.012	0.104	0.093
9	CTACA	0.042	0.178	0.136	ATCGT	0.053	0.175	0.122	GGATC	0.016	0.109	0.092
10	CCTTA	0.019	0.148	0.129	TACCA	0.031	0.147	0.116	TAGCG	0.034	0.124	0.090

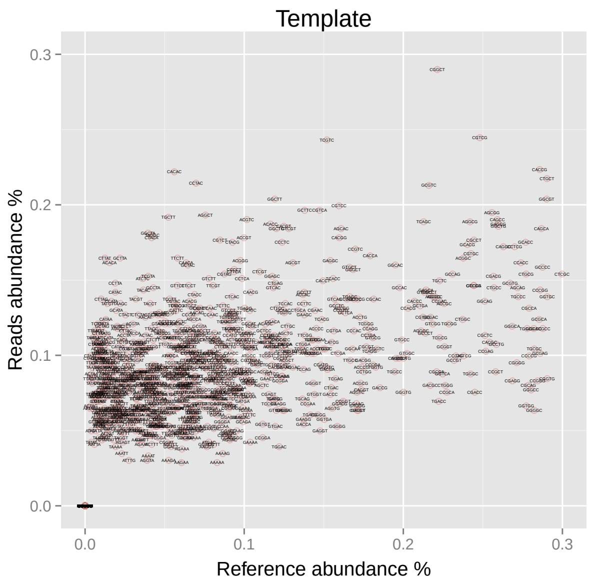

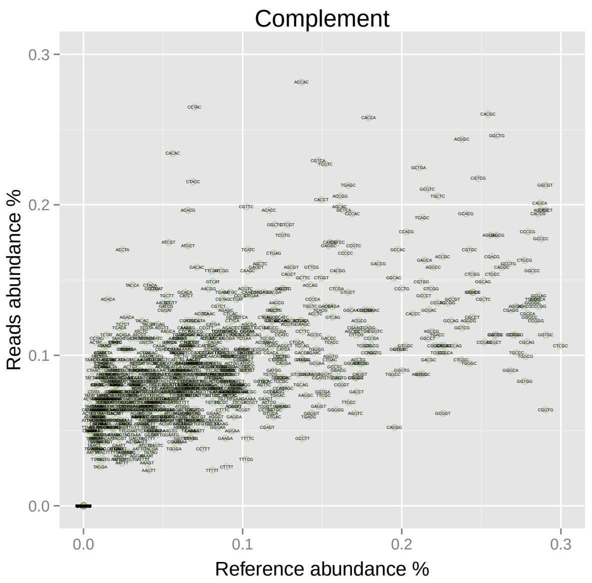

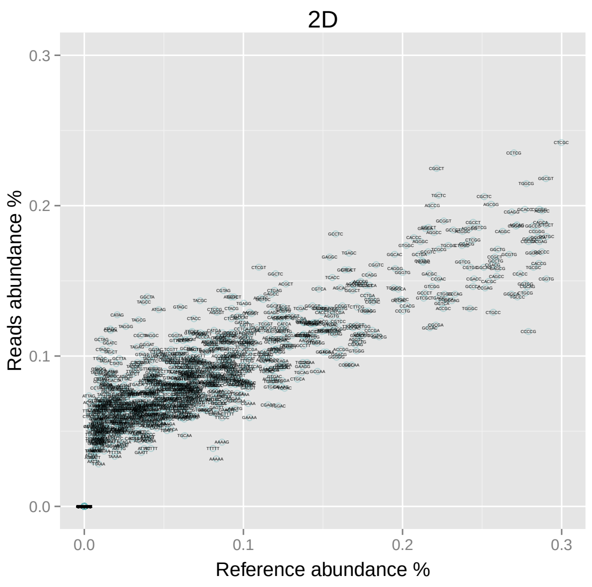

Deinococcus radiodurans 1 GC content

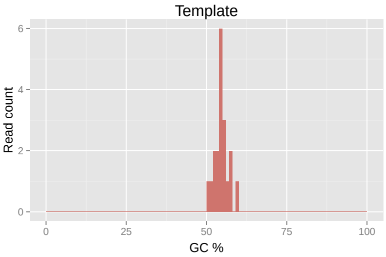

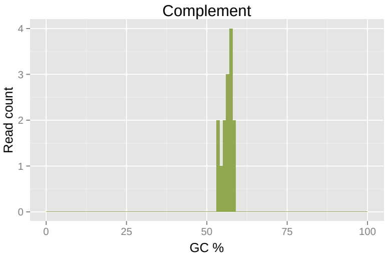

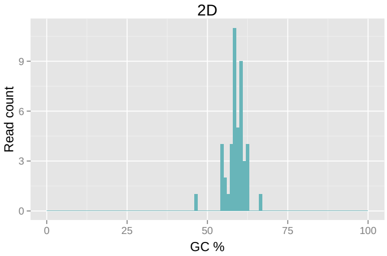

Deinococcus radiodurans 2 error analysis

	Template	Complement	2D
Overall base identity (excluding indels)	63.54%	48.95%	76.74%
Aligned base identity (excluding indels)	73.04%	78.46%	85.02%
Identical bases per 100 aligned bases (including indels)	61.16%	64.91%	74.65%
Inserted bases per 100 aligned bases (including indels)	5.47%	3.48%	5.53%
Deleted bases per 100 aligned bases (including indels)	10.79%	13.80%	6.67%
Substitutions per 100 aligned bases (including indels)	22.58%	17.82%	13.15%
Mean insertion size	1.60	1.49	1.56
Mean deletion size	1.60	1.83	1.55

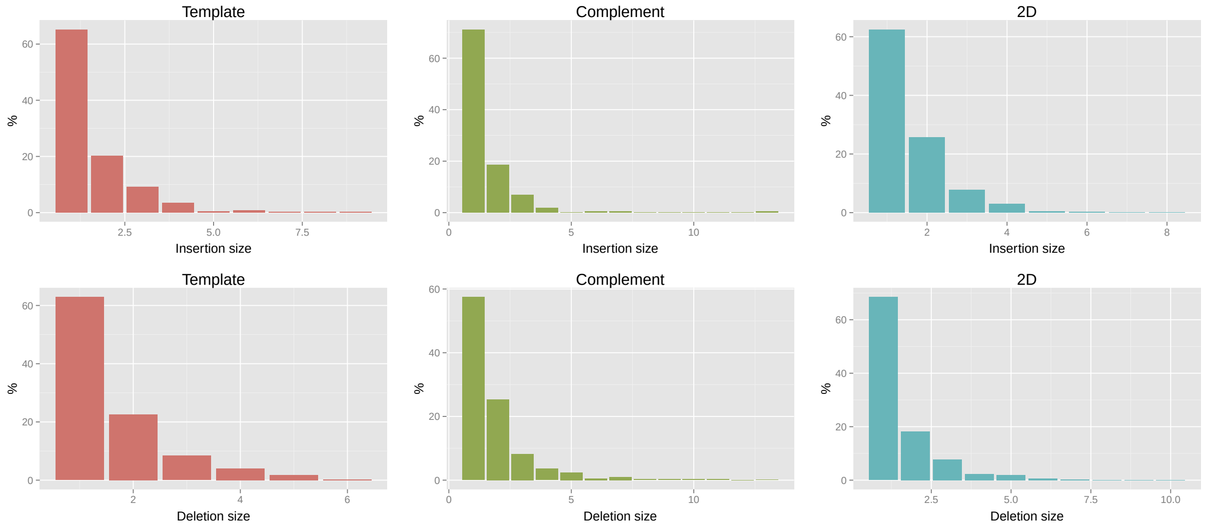

Deinococcus radiodurans 2 read identity

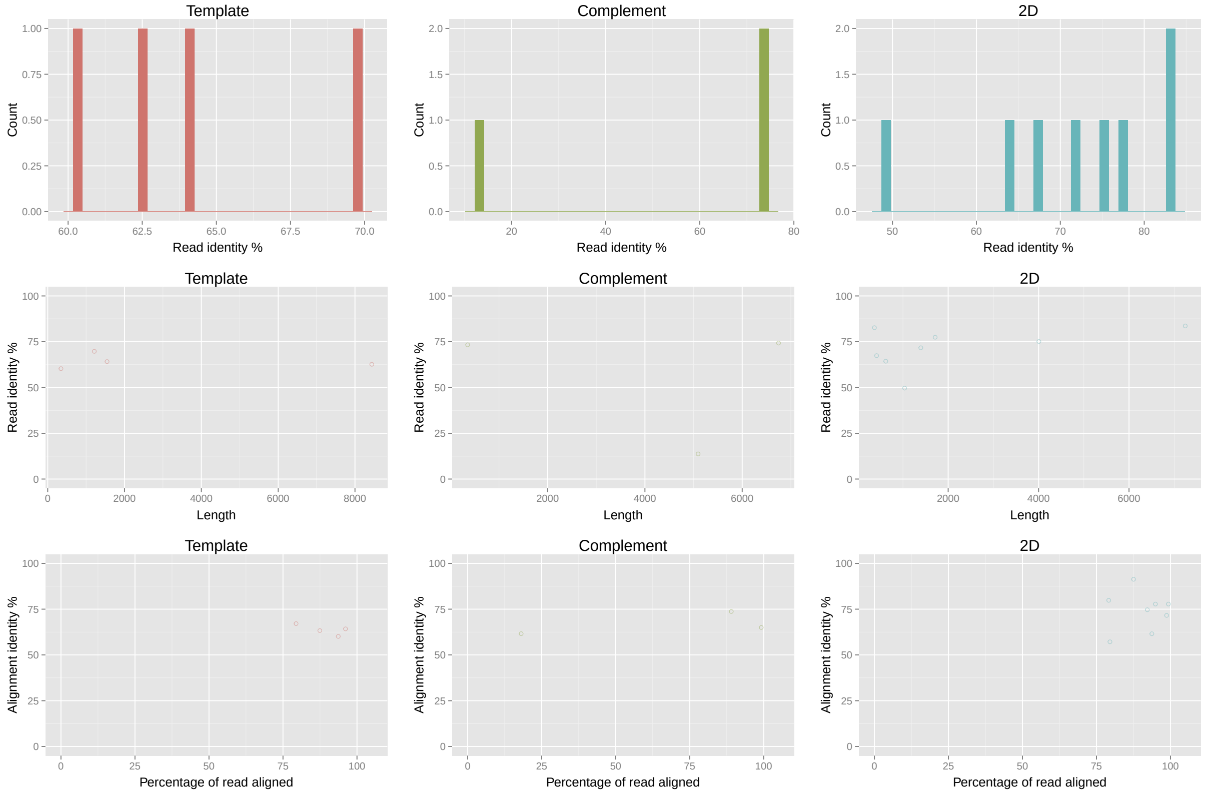

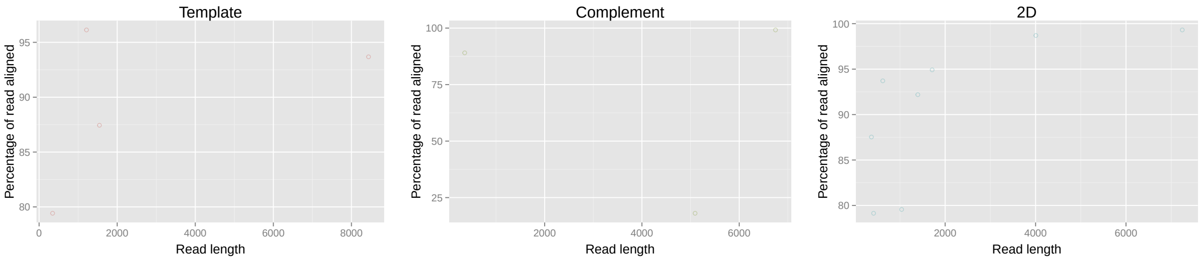

Deinococcus radiodurans 2 perfect kmers

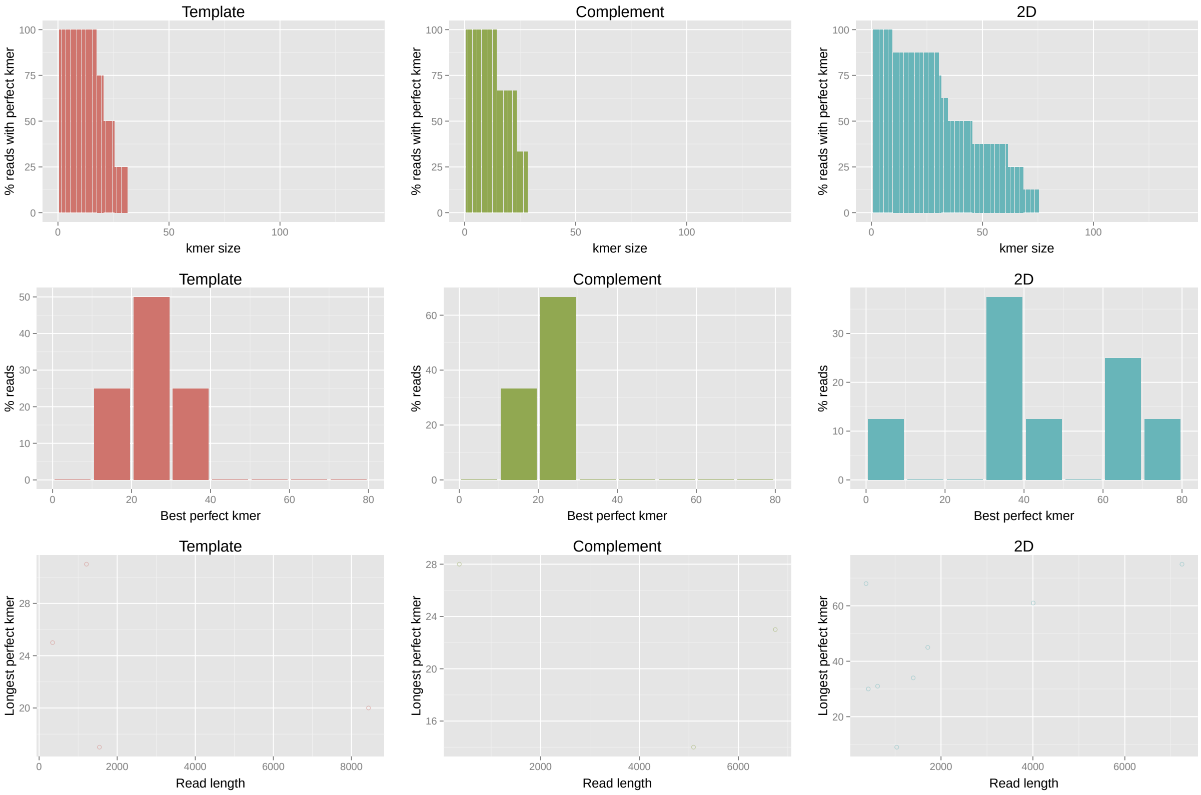

Deinococcus radiodurans 2 coverage

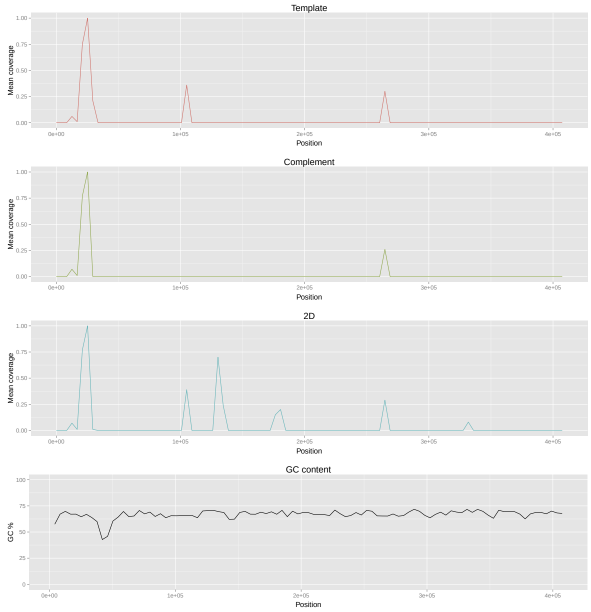

Deinococcus radiodurans 2 5-mer analysis

Under-represented 5-mers

	Template				Complement				2D			
Rank	kmer	Ref %	Read %	Diff %	kmer	Ref %	Read %	Diff %	kmer	Ref %	Read %	Diff %
1	CGCCG	0.740	0.226	-0.514	CGCCG	0.740	0.164	-0.575	CGCCG	0.740	0.346	-0.394
2	GCCGC	0.648	0.139	-0.509	GCCGC	0.648	0.181	-0.467	GCCGC	0.648	0.263	-0.385
3	CGGCG	0.724	0.234	-0.489	CGGCG	0.724	0.304	-0.420	CCGCC	0.530	0.161	-0.369
4	GCGGC	0.644	0.226	-0.418	GGCGC	0.556	0.140	-0.417	CGCCC	0.491	0.197	-0.294
5	GGGCG	0.452	0.069	-0.383	GCGCC	0.562	0.148	-0.414	GCTGC	0.427	0.167	-0.260
6	CGCGC	0.477	0.095	-0.381	CCGCC	0.530	0.131	-0.399	CCCGC	0.410	0.155	-0.255
7	GCAGC	0.432	0.069	-0.362	GCGGC	0.644	0.246	-0.398	GCGCC	0.562	0.316	-0.245
8	GCGCC	0.562	0.200	-0.362	CGCCC	0.491	0.123	-0.368	CAGCG	0.418	0.179	-0.239
9	GGCGG	0.523	0.165	-0.358	GGCGG	0.523	0.156	-0.367	CGGCG	0.724	0.489	-0.234
10	GGCGC	0.556	0.200	-0.357	CGGGC	0.449	0.115	-0.335	GGCGC	0.556	0.334	-0.222

Over-represented 5-mers

	Template				Complement				2D			
Rank	kmer	Ref %	Read %	Diff %	kmer	Ref %	Read %	Diff %	kmer	Ref %	Read %	Diff %
1	CTAGG	0.009	0.226	0.217	AACGA	0.064	0.205	0.141	CGGTA	0.048	0.191	0.143
2	CCTAA	0.007	0.182	0.175	ACAGA	0.024	0.164	0.141	TAGGG	0.022	0.155	0.133
3	ATCTC	0.034	0.208	0.174	GATAG	0.016	0.156	0.140	CTAGG	0.009	0.119	0.110
4	CCTTA	0.017	0.174	0.157	TAGCA	0.009	0.140	0.130	GTAGG	0.055	0.161	0.106
5	CCTAG	0.010	0.165	0.155	GCACA	0.072	0.197	0.125	TGCTA	0.011	0.113	0.103
6	TAACC	0.013	0.165	0.152	TGAGA	0.024	0.148	0.124	TCAGT	0.041	0.137	0.097
7	ACCTA	0.031	0.182	0.152	TACAG	0.034	0.156	0.122	GGCTA	0.041	0.137	0.097
8	TTAAC	0.006	0.156	0.150	AAGCC	0.109	0.230	0.121	GAGCC	0.120	0.215	0.095
9	TAGGC	0.034	0.182	0.149	GAGAC	0.046	0.164	0.118	CCATC	0.120	0.215	0.095
10	CTACC	0.075	0.217	0.142	CCAAT	0.024	0.140	0.115	GCTGT	0.109	0.203	0.094

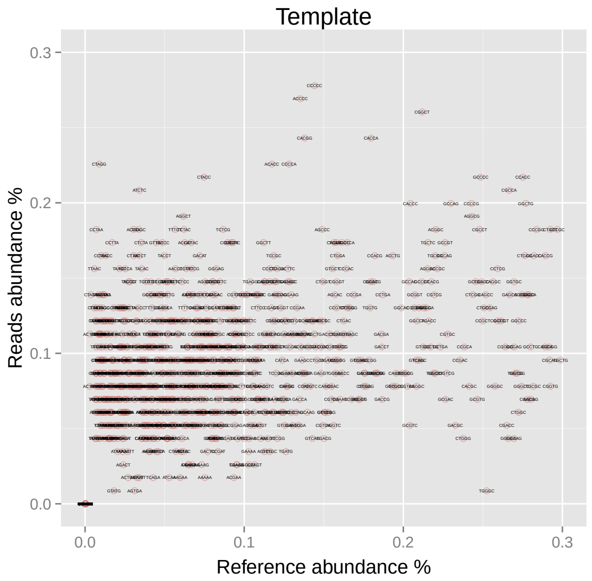

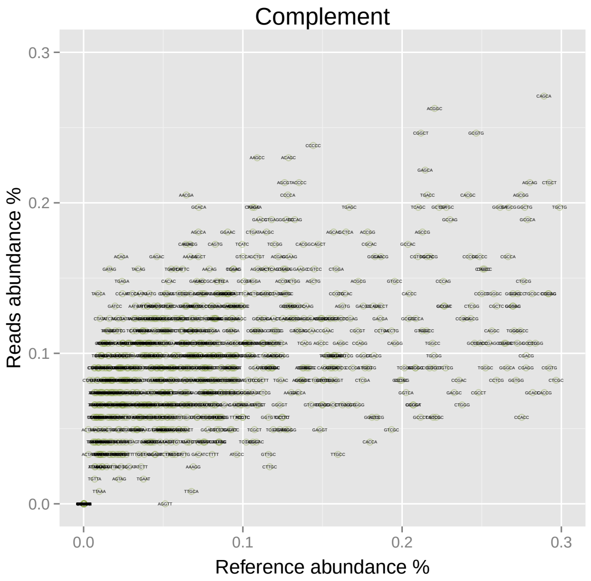

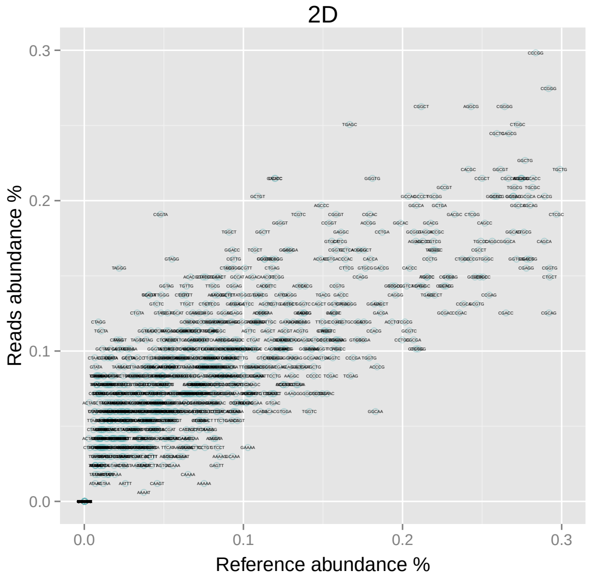

Deinococcus radiodurans 2 GC content

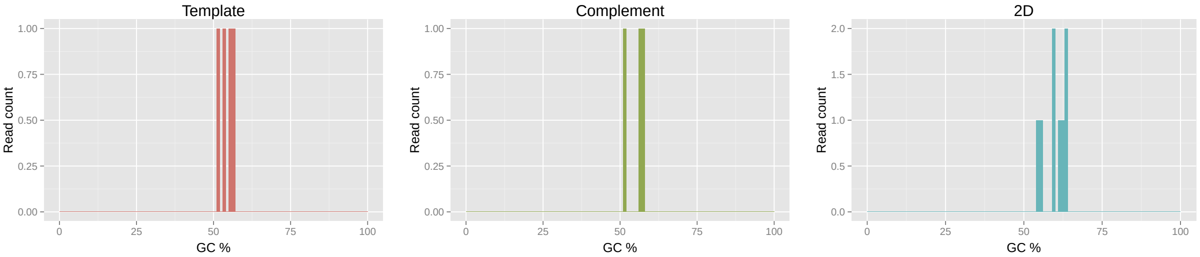

Enterococcus faecalis error analysis

	Template	Complement	2D
Overall base identity (excluding indels)	33.46%	58.66%	72.76%
Aligned base identity (excluding indels)	77.45%	77.57%	86.41%
Identical bases per 100 aligned bases (including indels)	64.13%	63.18%	76.23%
Inserted bases per 100 aligned bases (including indels)	3.28%	2.33%	5.83%
Deleted bases per 100 aligned bases (including indels)	13.92%	16.23%	5.95%
Substitutions per 100 aligned bases (including indels)	18.67%	18.26%	11.99%
Mean insertion size	1.46	1.30	1.51
Mean deletion size	1.80	1.90	1.45

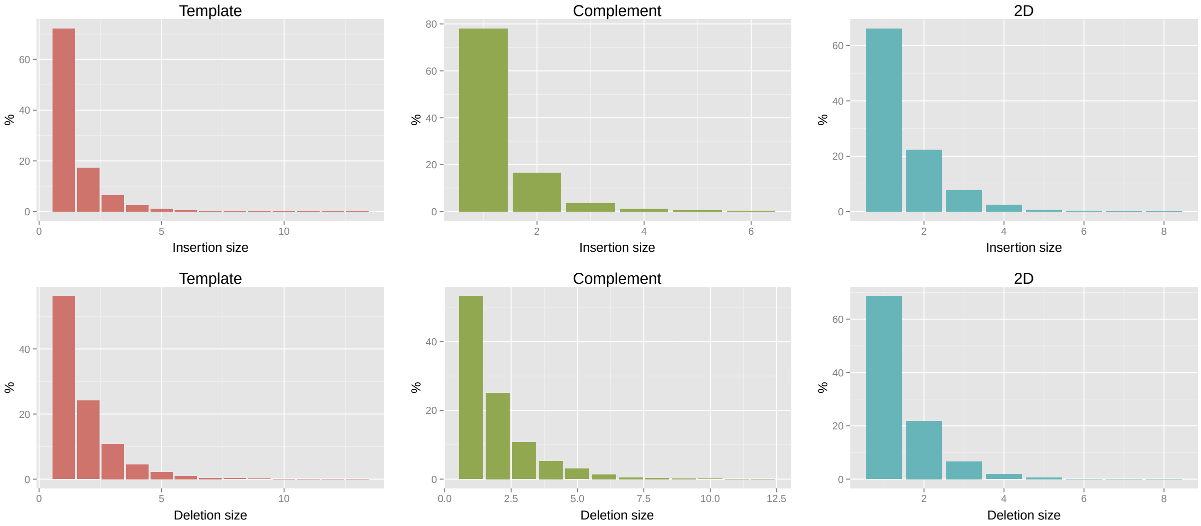

Enterococcus faecalis read identity

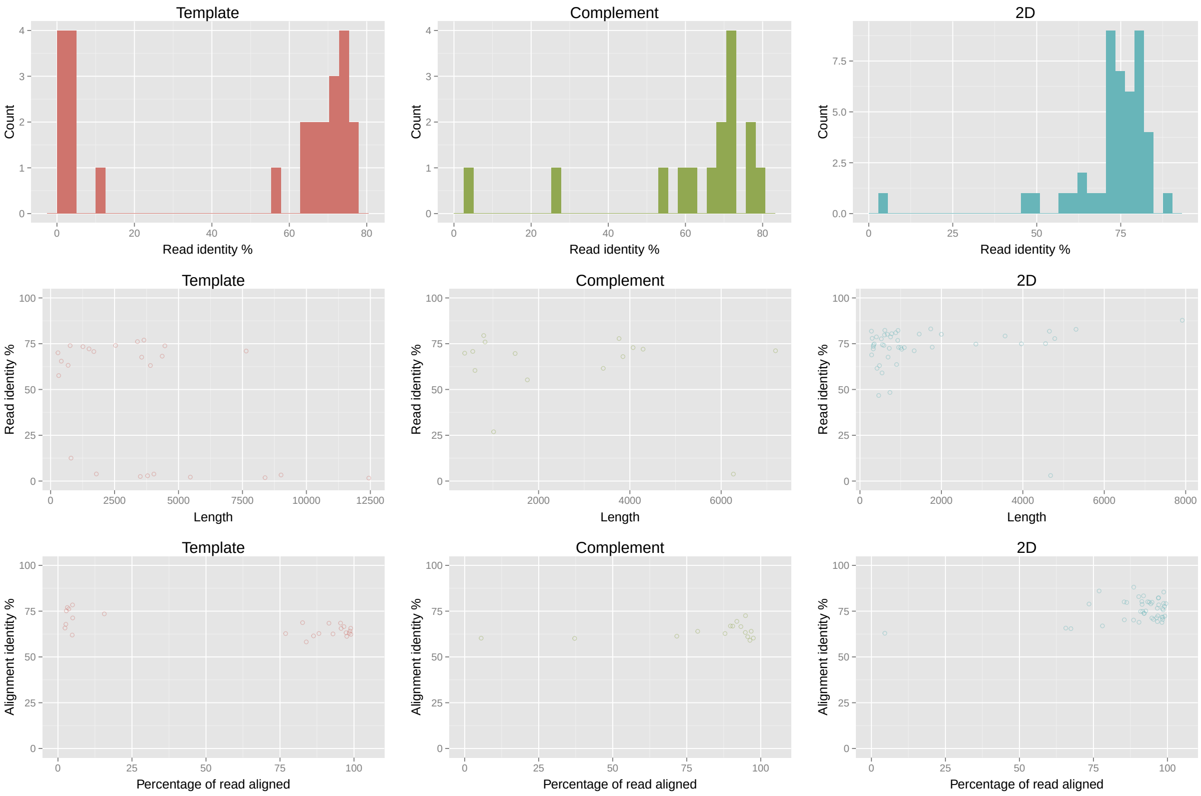

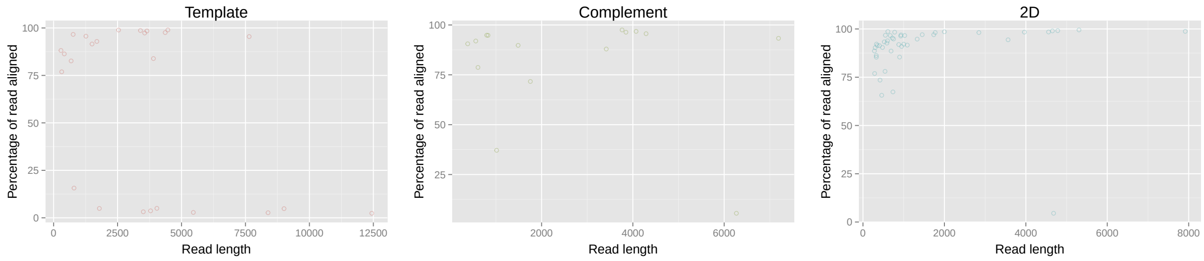

Enterococcus faecalis perfect kmers

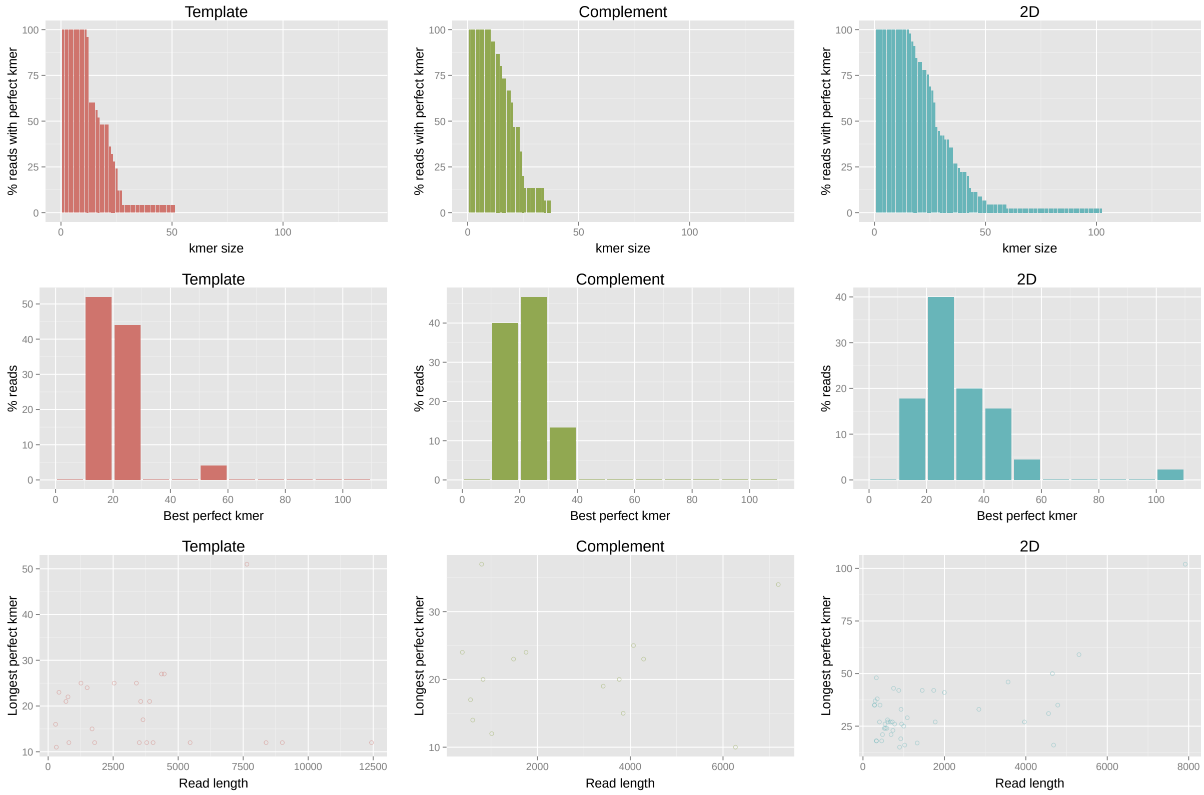

Enterococcus faecalis coverage

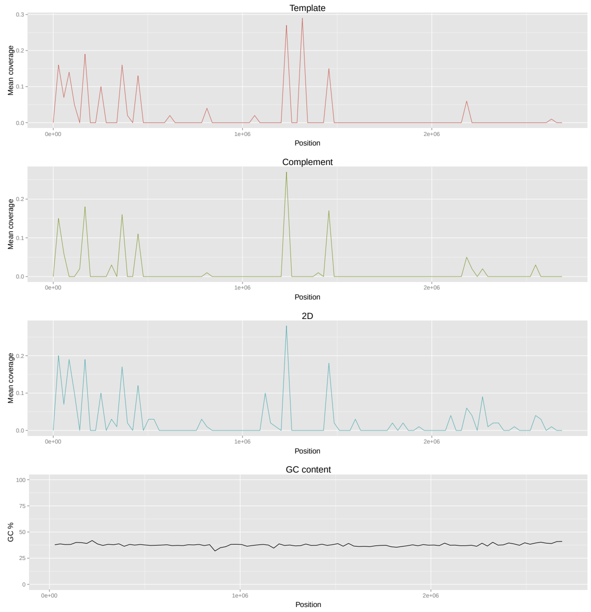

Enterococcus faecalis 5-mer analysis

Under-represented 5-mers

	Template				Complement				2D			
Rank	kmer	Ref %	Read %	Diff %	kmer	Ref %	Read %	Diff %	kmer	Ref %	Read %	Diff %
1	TTTTT	0.739	0.115	-0.624	AAAAA	0.721	0.102	-0.619	TTTTT	0.739	0.113	-0.626
2	AAAAA	0.721	0.099	-0.621	TTTTT	0.739	0.164	-0.575	AAAAA	0.721	0.196	-0.524
3	AAAAT	0.512	0.132	-0.381	AAAAT	0.512	0.137	-0.375	ATTTT	0.509	0.222	-0.287
4	TAAAA	0.427	0.066	-0.361	TAAAA	0.427	0.090	-0.337	TTTTA	0.427	0.150	-0.276
5	ATTTT	0.509	0.168	-0.342	ATTTT	0.509	0.209	-0.300	TAAAA	0.427	0.193	-0.233
6	TTTTA	0.427	0.116	-0.311	TTTTA	0.427	0.152	-0.275	CTTTT	0.361	0.148	-0.214
7	AAATT	0.399	0.097	-0.302	AAAAG	0.357	0.090	-0.267	AAAAT	0.512	0.311	-0.201
8	TTTTC	0.381	0.105	-0.276	AAATT	0.399	0.132	-0.267	AATTT	0.402	0.238	-0.164
9	AAAAG	0.357	0.082	-0.275	TTAAA	0.350	0.104	-0.245	AAAAG	0.357	0.198	-0.159
10	CAAAA	0.341	0.076	-0.266	CAAAA	0.341	0.102	-0.239	TTTTC	0.381	0.236	-0.145

Over-represented 5-mers

	Template				Complement				2D			
Rank	kmer	Ref %	Read %	Diff %	kmer	Ref %	Read %	Diff %	kmer	Ref %	Read %	Diff %
1	GTTGT	0.131	0.582	0.451	TCGTC	0.061	0.236	0.175	CATGC	0.053	0.113	0.060
2	TGTTG	0.181	0.604	0.423	GCGTG	0.036	0.174	0.138	ACCGG	0.017	0.076	0.059
3	TTGTT	0.318	0.546	0.228	GTCGT	0.062	0.192	0.130	ATGCA	0.090	0.149	0.059
4	CGGGC	0.021	0.206	0.185	ATGCT	0.091	0.216	0.126	GCATG	0.054	0.110	0.057
5	GCGTG	0.036	0.168	0.131	CGTCG	0.029	0.149	0.120	GGGCA	0.044	0.099	0.055
6	GAGGC	0.031	0.142	0.111	GAGAG	0.031	0.149	0.118	GAAGG	0.092	0.146	0.054
7	AGGCT	0.058	0.168	0.109	CGTGC	0.038	0.142	0.104	CGTGT	0.050	0.103	0.053
8	CGTCG	0.029	0.137	0.108	AGAGA	0.077	0.179	0.103	GTGTT	0.098	0.150	0.053
9	CGGCG	0.042	0.150	0.107	GTCTT	0.089	0.192	0.102	GTGCT	0.062	0.115	0.053
10	GTCGT	0.062	0.169	0.107	CGTCC	0.035	0.132	0.097	GATGC	0.082	0.135	0.053

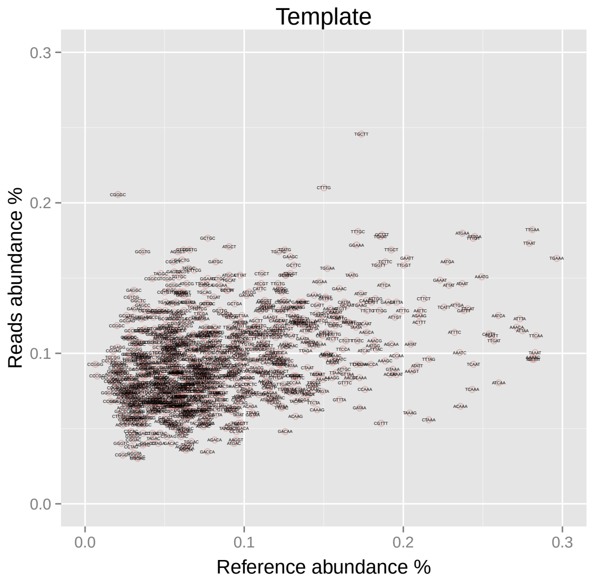

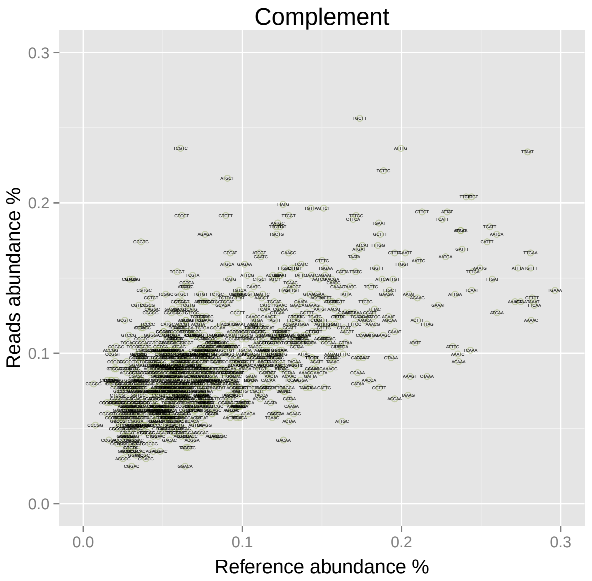

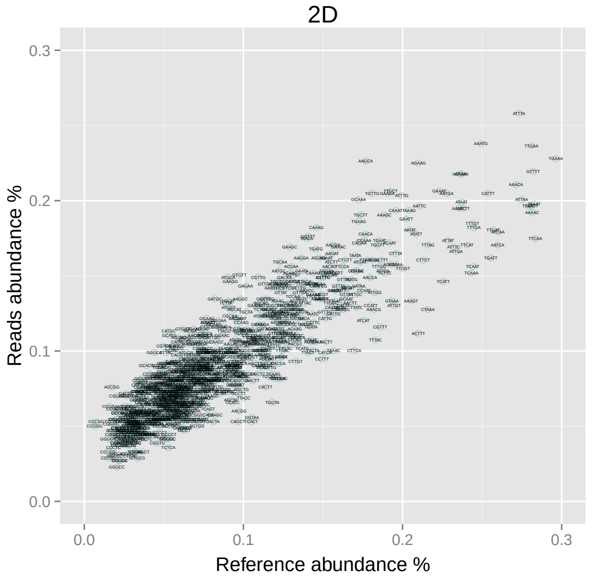

Enterococcus faecalis GC content

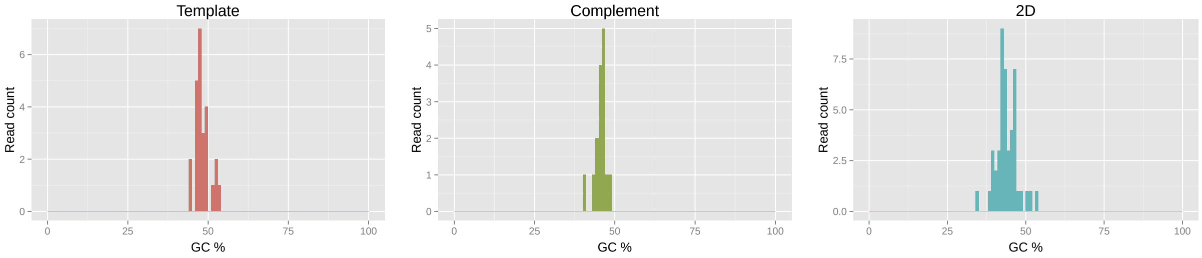

Escherichia coli error analysis

	Template	Complement	2D
Overall base identity (excluding indels)	72.32%	72.28%	79.72%
Aligned base identity (excluding indels)	78.30%	79.58%	88.70%
Identical bases per 100 aligned bases (including indels)	65.28%	64.59%	78.30%
Inserted bases per 100 aligned bases (including indels)	4.20%	2.73%	7.39%
Deleted bases per 100 aligned bases (including indels)	12.43%	16.11%	4.34%
Substitutions per 100 aligned bases (including indels)	18.10%	16.57%	9.98%
Mean insertion size	1.50	1.39	1.67
Mean deletion size	1.66	1.82	1.39

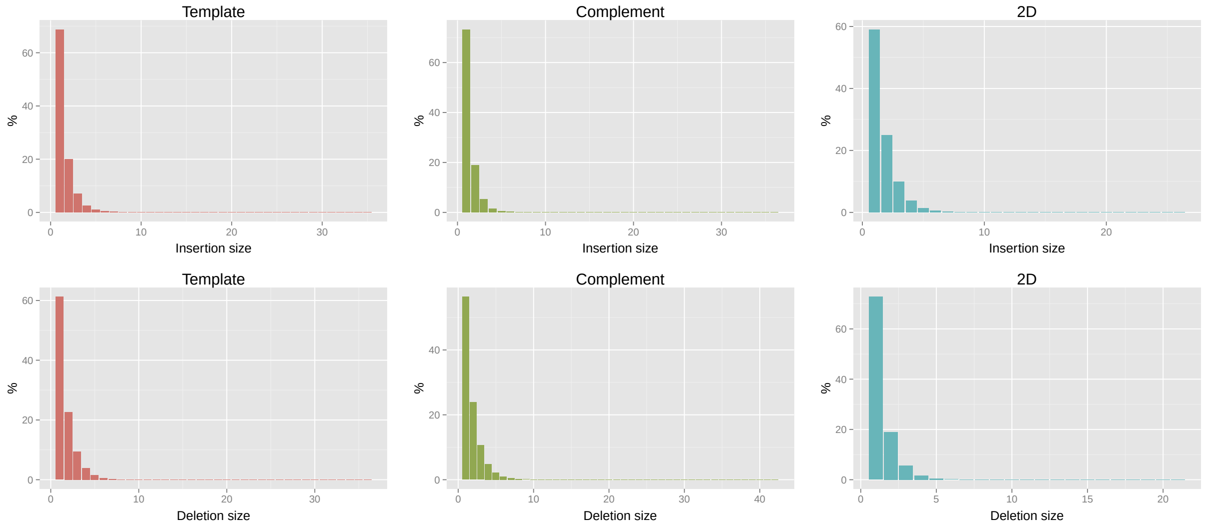

Escherichia coli read identity

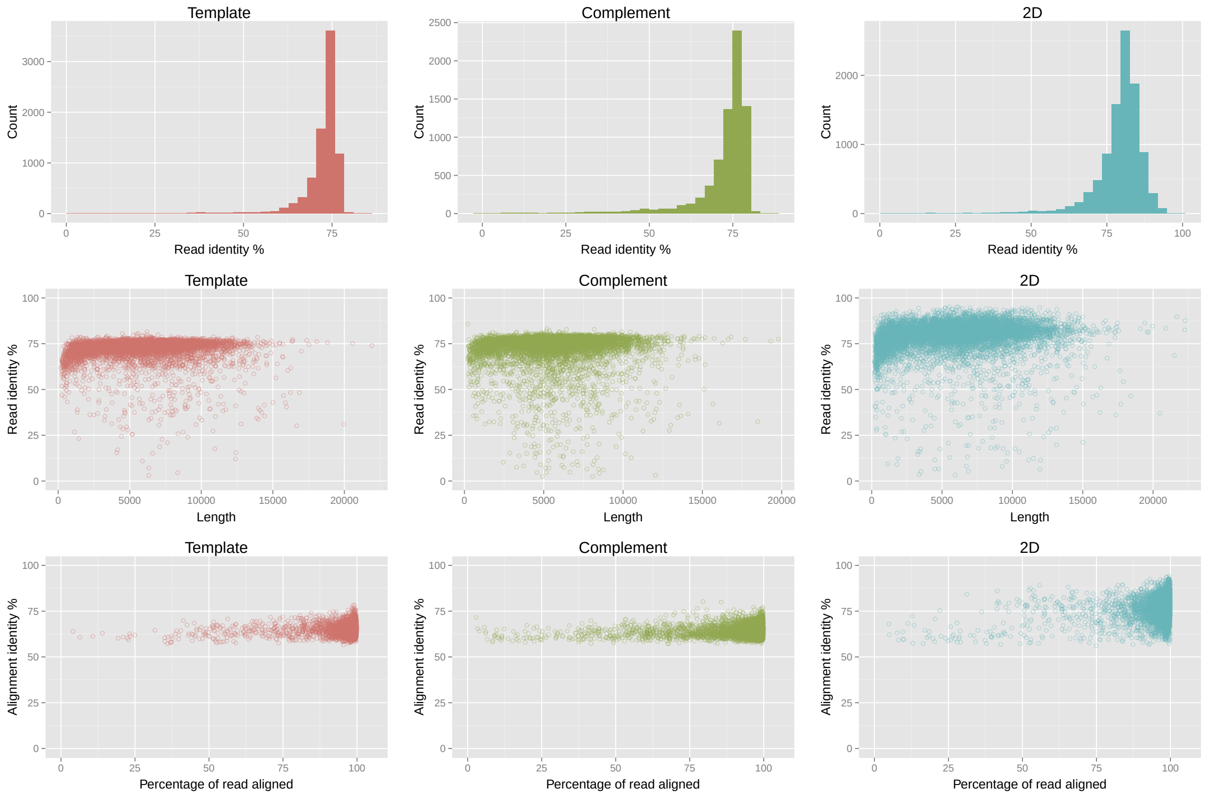

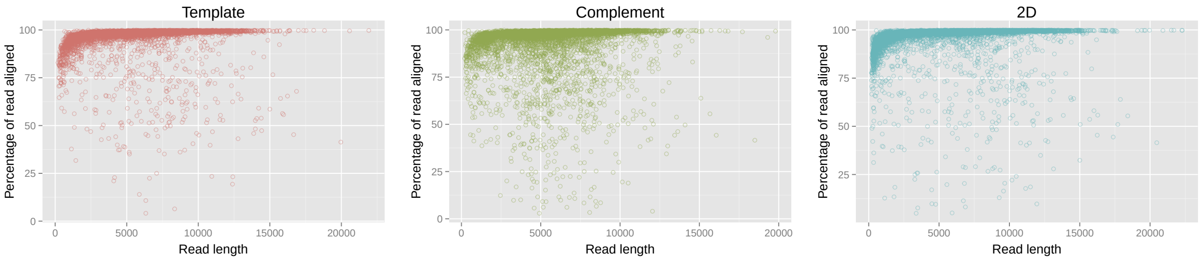

Escherichia coli perfect kmers

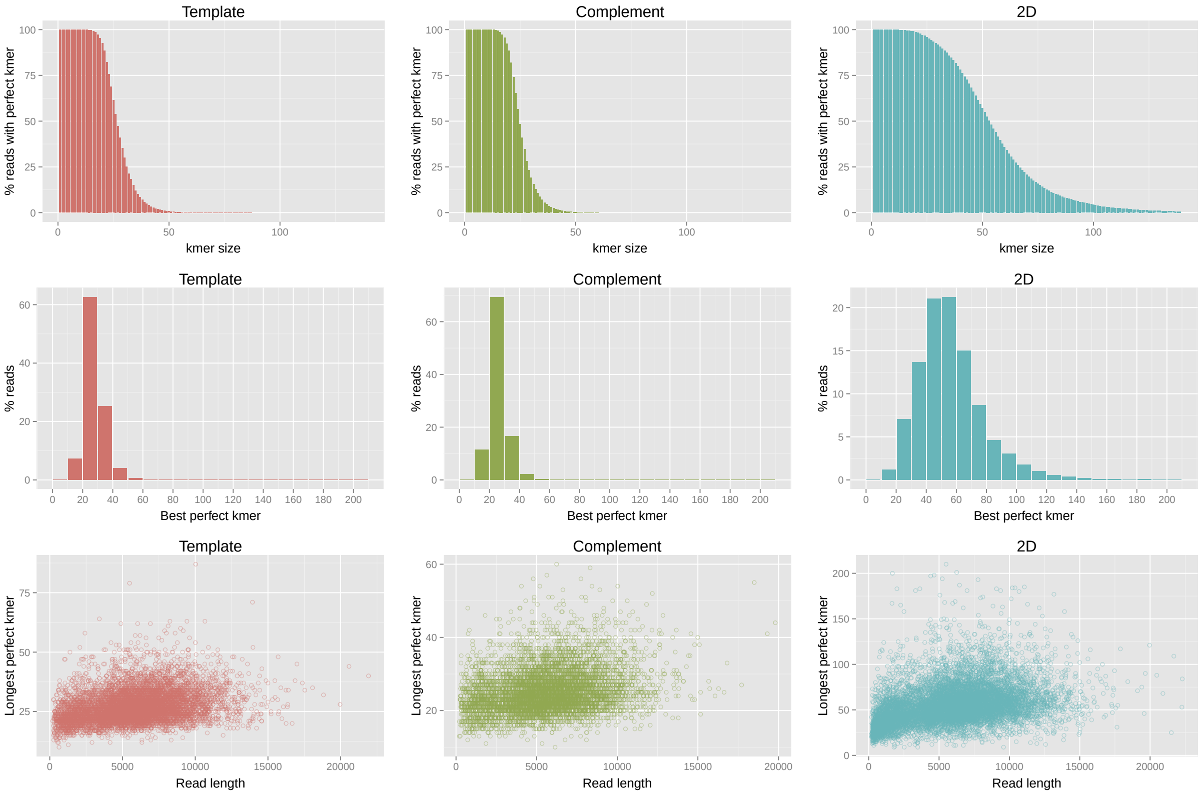

Escherichia coli coverage

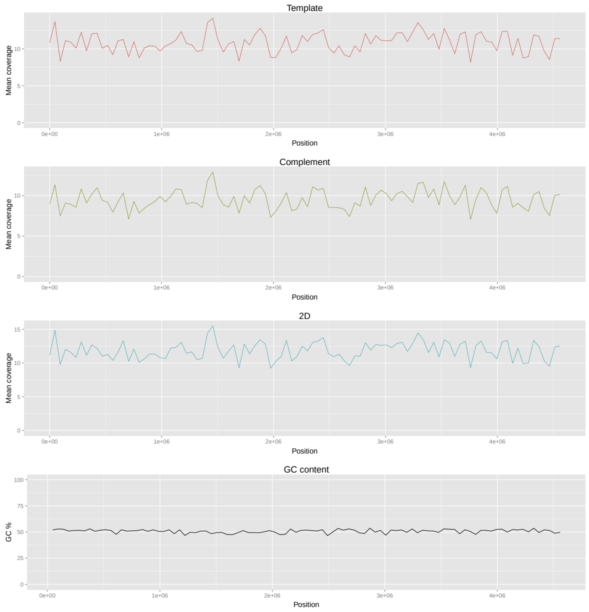

Escherichia coli 5-mer analysis

Under-represented 5-mers

	Template				Complement				2D			
Rank	kmer	Ref %	Read %	Diff %	kmer	Ref %	Read %	Diff %	kmer	Ref %	Read %	Diff %
1	AAAAA	0.247	0.076	-0.172	CGCCA	0.288	0.083	-0.205	TTTTT	0.251	0.084	-0.167
2	CGCTG	0.258	0.097	-0.161	AAAAA	0.247	0.063	-0.185	AAAAA	0.247	0.097	-0.151
3	TTTTT	0.251	0.093	-0.158	TTTTT	0.251	0.068	-0.183	CCAGC	0.288	0.161	-0.127
4	CGCCA	0.288	0.131	-0.157	CTGGC	0.279	0.122	-0.156	GCCAG	0.280	0.167	-0.113
5	GCCAG	0.280	0.131	-0.148	CGCTG	0.258	0.105	-0.154	GCTGG	0.280	0.178	-0.102
6	CTGGC	0.279	0.140	-0.139	GCCAG	0.280	0.128	-0.152	CGCCA	0.288	0.191	-0.097
7	GCTGG	0.280	0.143	-0.137	CCAGC	0.288	0.143	-0.146	CAGCG	0.262	0.172	-0.091
8	CCAGC	0.288	0.157	-0.131	TGGCG	0.276	0.134	-0.142	TGGCG	0.276	0.191	-0.085
9	TGGCG	0.276	0.148	-0.128	GCTGG	0.280	0.141	-0.139	CTGGC	0.279	0.194	-0.085
10	CGCCG	0.220	0.108	-0.112	CGCCG	0.220	0.086	-0.134	CGCTG	0.258	0.175	-0.084

Over-represented 5-mers

	Template				Complement				2D			
Rank	kmer	Ref %	Read %	Diff %	kmer	Ref %	Read %	Diff %	kmer	Ref %	Read %	Diff %
1	TAGGC	0.031	0.124	0.093	TCGTA	0.053	0.167	0.114	TAGAT	0.035	0.082	0.047
2	CGGGC	0.116	0.205	0.090	CGGCT	0.109	0.214	0.105	CTAGA	0.003	0.046	0.043
3	TCGTA	0.053	0.139	0.086	GTCGT	0.078	0.166	0.088	ACTAG	0.006	0.048	0.043
4	GAGGC	0.051	0.135	0.083	CGTAT	0.071	0.151	0.079	GCTAG	0.007	0.049	0.042
5	TGCTT	0.099	0.180	0.081	TGCTT	0.099	0.178	0.079	CTAGT	0.006	0.047	0.041
6	TAGTA	0.027	0.106	0.079	CTGAG	0.050	0.126	0.077	CGTAG	0.058	0.097	0.040
7	TCTTA	0.045	0.121	0.076	TCGTG	0.069	0.144	0.075	TCTAG	0.003	0.043	0.039
8	GGCTC	0.046	0.121	0.075	GGCTC	0.046	0.121	0.075	TTAGA	0.026	0.065	0.039
9	AGGCT	0.069	0.144	0.075	TAGTA	0.027	0.101	0.074	ACACG	0.050	0.089	0.038
10	CTTAG	0.022	0.097	0.075	CGTAG	0.058	0.131	0.074	ACGAG	0.040	0.077	0.038

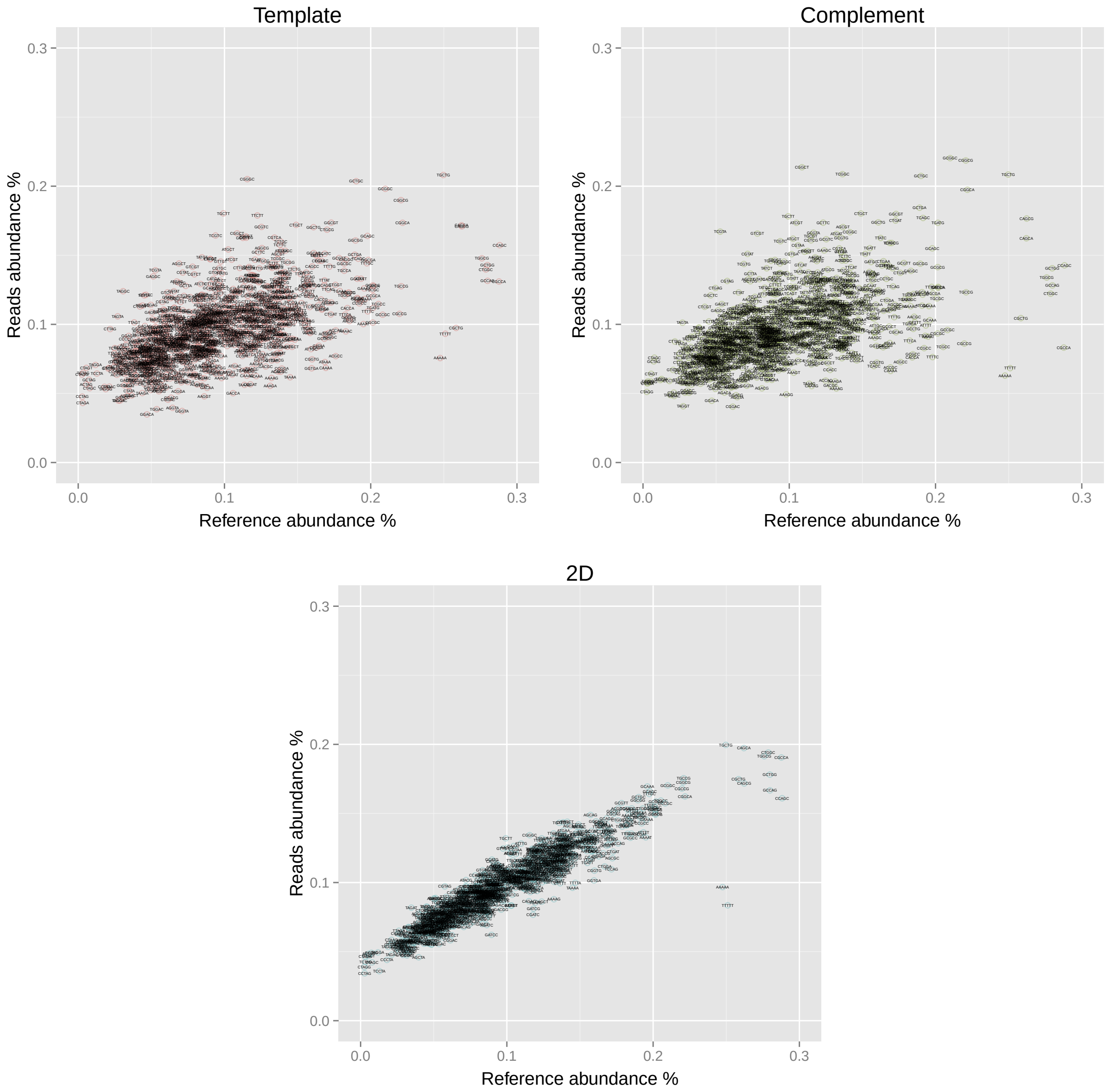

Escherichia coli GC content

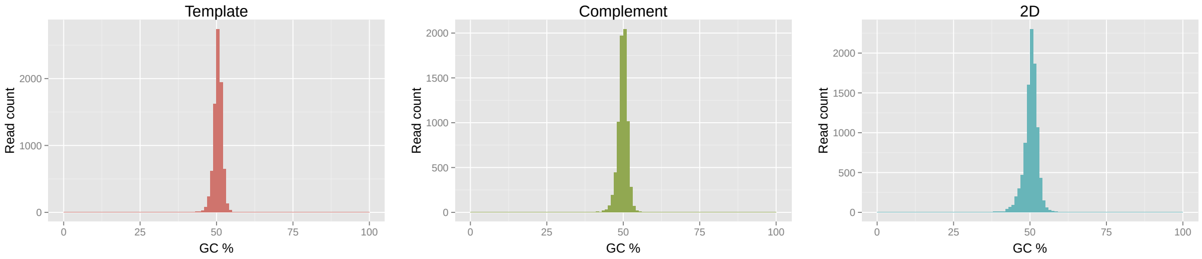

Helicobacter pylori error analysis

	Template	Complement	2D
Overall base identity (excluding indels)	70.29%	63.71%	77.32%
Aligned base identity (excluding indels)	76.56%	78.40%	86.31%
Identical bases per 100 aligned bases (including indels)	62.62%	62.90%	75.63%
Inserted bases per 100 aligned bases (including indels)	2.95%	2.47%	5.77%
Deleted bases per 100 aligned bases (including indels)	15.25%	17.29%	6.60%
Substitutions per 100 aligned bases (including indels)	19.18%	17.33%	11.99%
Mean insertion size	1.44	1.41	1.57
Mean deletion size	1.88	1.98	1.50

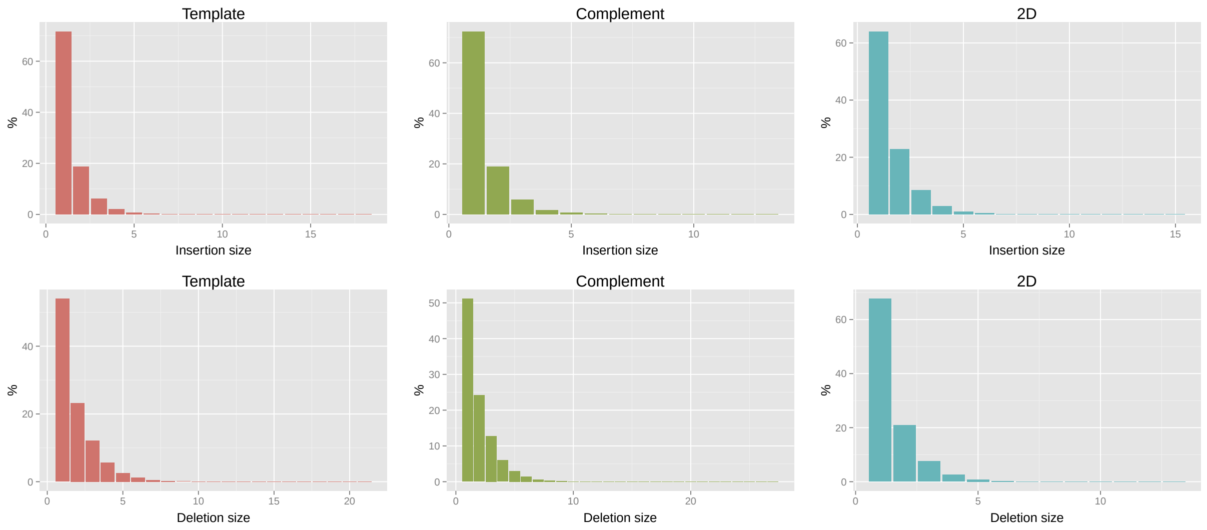

Helicobacter pylori read identity

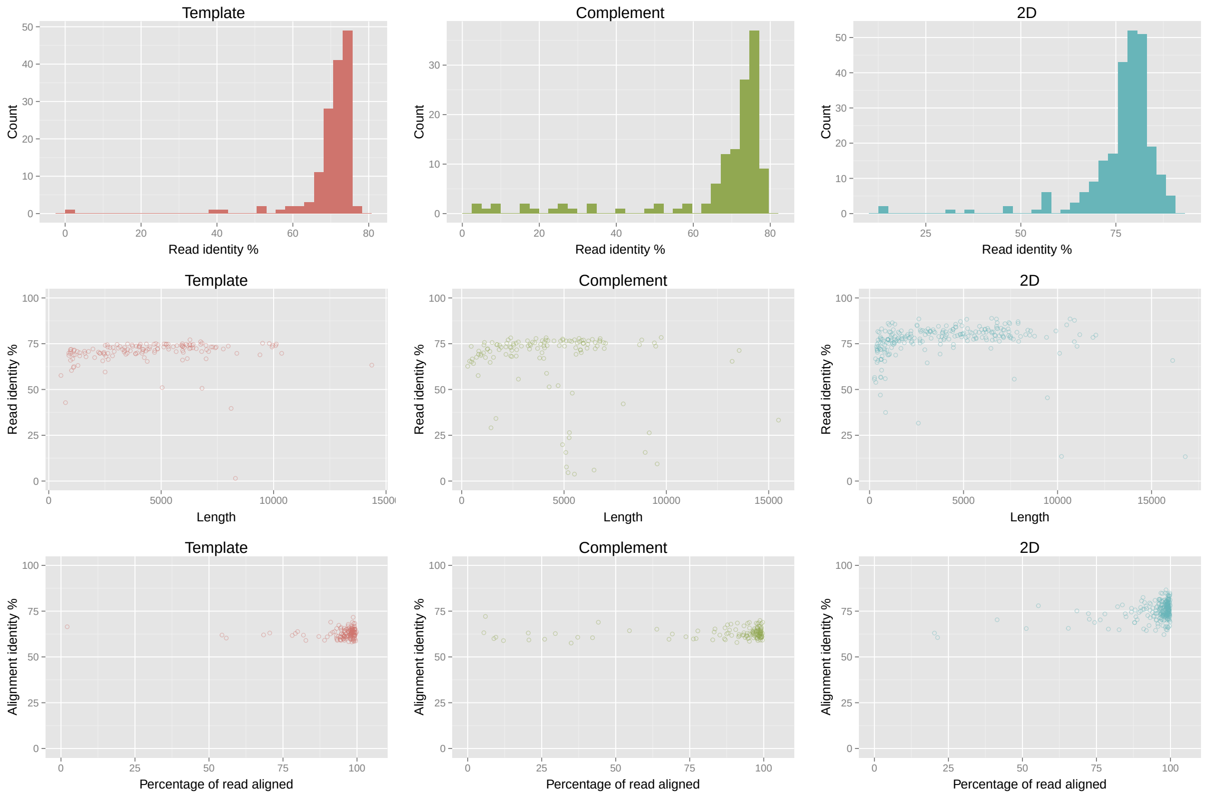

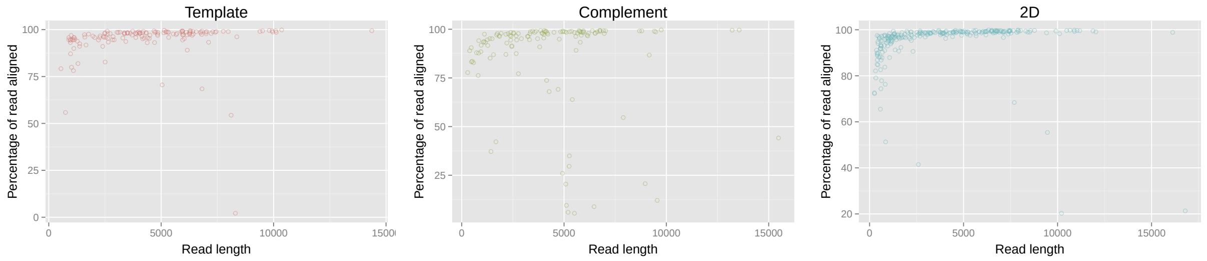

Helicobacter pylori perfect kmers

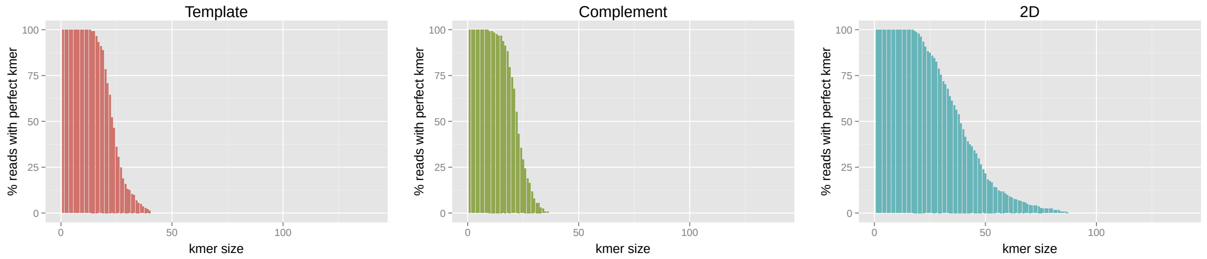

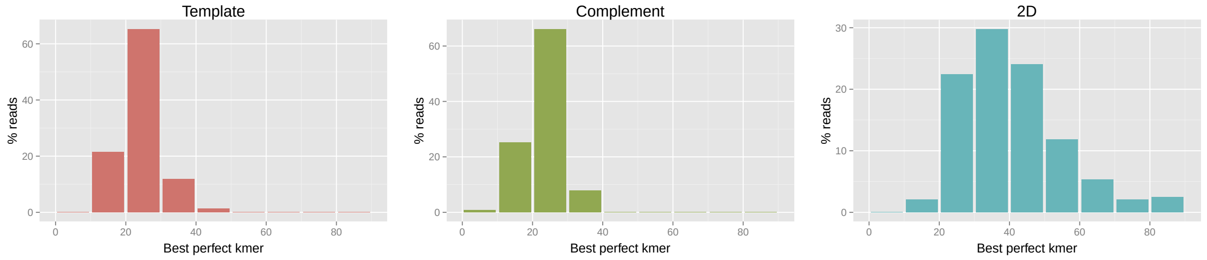

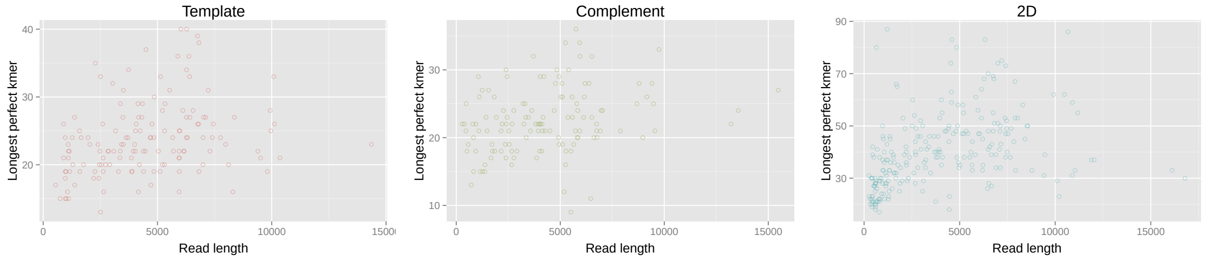

Helicobacter pylori coverage

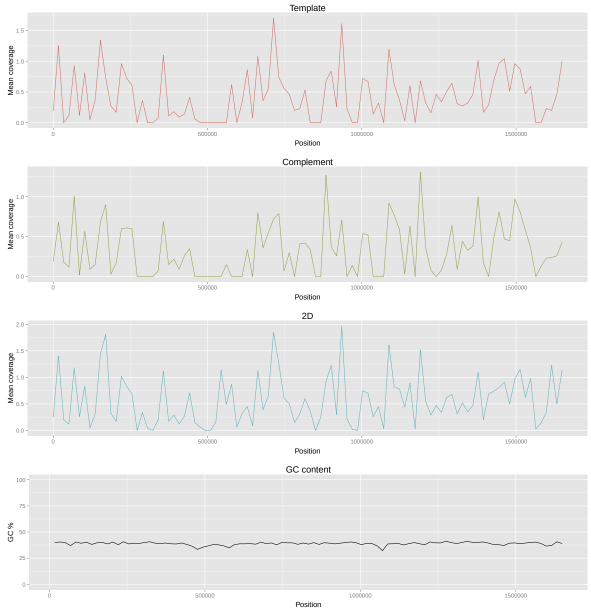

Helicobacter pylori 5-mer analysis

Under-represented 5-mers

Rank	Template kmer	Template Ref %	Template Read %	Template Diff %	Complement kmer	Complement Ref %	Complement Read %	Complement Diff %	2D kmer	2D Ref %	2D Read %	2D Diff %
1	AAAAA	0.944	0.144	-0.800	TTTTT	0.984	0.147	-0.837	TTTTT	0.984	0.198	-0.786
2	TTTTT	0.984	0.192	-0.792	AAAAA	0.944	0.140	-0.804	AAAAA	0.944	0.226	-0.718
3	TAAAA	0.712	0.137	-0.575	TAAAA	0.712	0.187	-0.525	TAAAA	0.712	0.327	-0.386
4	TTAAA	0.599	0.183	-0.416	TTTTA	0.711	0.266	-0.445	TTTTA	0.711	0.345	-0.366
5	TTTTA	0.711	0.300	-0.411	AAAAT	0.567	0.160	-0.407	AAAAT	0.567	0.298	-0.269
6	AAAAT	0.567	0.157	-0.410	TTAAA	0.599	0.198	-0.401	TTTAA	0.609	0.351	-0.258
7	TTTAA	0.609	0.239	-0.370	AAAAG	0.474	0.109	-0.366	ATTTT	0.577	0.326	-0.251
8	AAAAG	0.474	0.124	-0.351	ATTTT	0.577	0.234	-0.343	TTAAA	0.599	0.356	-0.243
9	CAAAA	0.438	0.118	-0.320	TTTAA	0.609	0.271	-0.338	AAAAG	0.474	0.233	-0.241
10	AAAAC	0.430	0.131	-0.299	CTTTT	0.505	0.185	-0.320	CTTTT	0.505	0.269	-0.236

Over-represented 5-mers

Rank	Template kmer	Template Ref %	Template Read %	Template Diff %	Complement kmer	Complement Ref %	Complement Read %	Complement Diff %	2D kmer	2D Ref %	2D Read %	2D Diff %
1	CGGGC	0.031	0.183	0.152	TCGTA	0.036	0.155	0.118	TCGAC	0.001	0.070	0.070
2	GCTGC	0.033	0.145	0.112	TCGGC	0.028	0.143	0.115	TTCGA	0.005	0.069	0.064
3	TCGTC	0.026	0.134	0.108	GCTGC	0.033	0.147	0.114	GCAGG	0.031	0.094	0.063
4	GAGGA	0.026	0.126	0.100	GTCGT	0.032	0.136	0.104	CGGTT	0.056	0.119	0.062
5	TTCGG	0.022	0.121	0.099	CGTAA	0.045	0.147	0.102	GGTTC	0.027	0.089	0.062
6	AGGAG	0.046	0.143	0.097	GCGGC	0.047	0.146	0.100	GAAGG	0.037	0.097	0.061
7	CTGCT	0.044	0.140	0.096	CGGCG	0.032	0.131	0.099	CGGGC	0.031	0.089	0.057
8	TCGGG	0.011	0.106	0.094	CGGCT	0.068	0.163	0.095	AGGAA	0.068	0.123	0.055
9	CGTCG	0.008	0.103	0.094	CGTAG	0.023	0.119	0.095	CCGAC	0.007	0.061	0.054
10	TCGTA	0.036	0.129	0.093	GAGGA	0.026	0.117	0.091	TGTAC	0.001	0.055	0.054

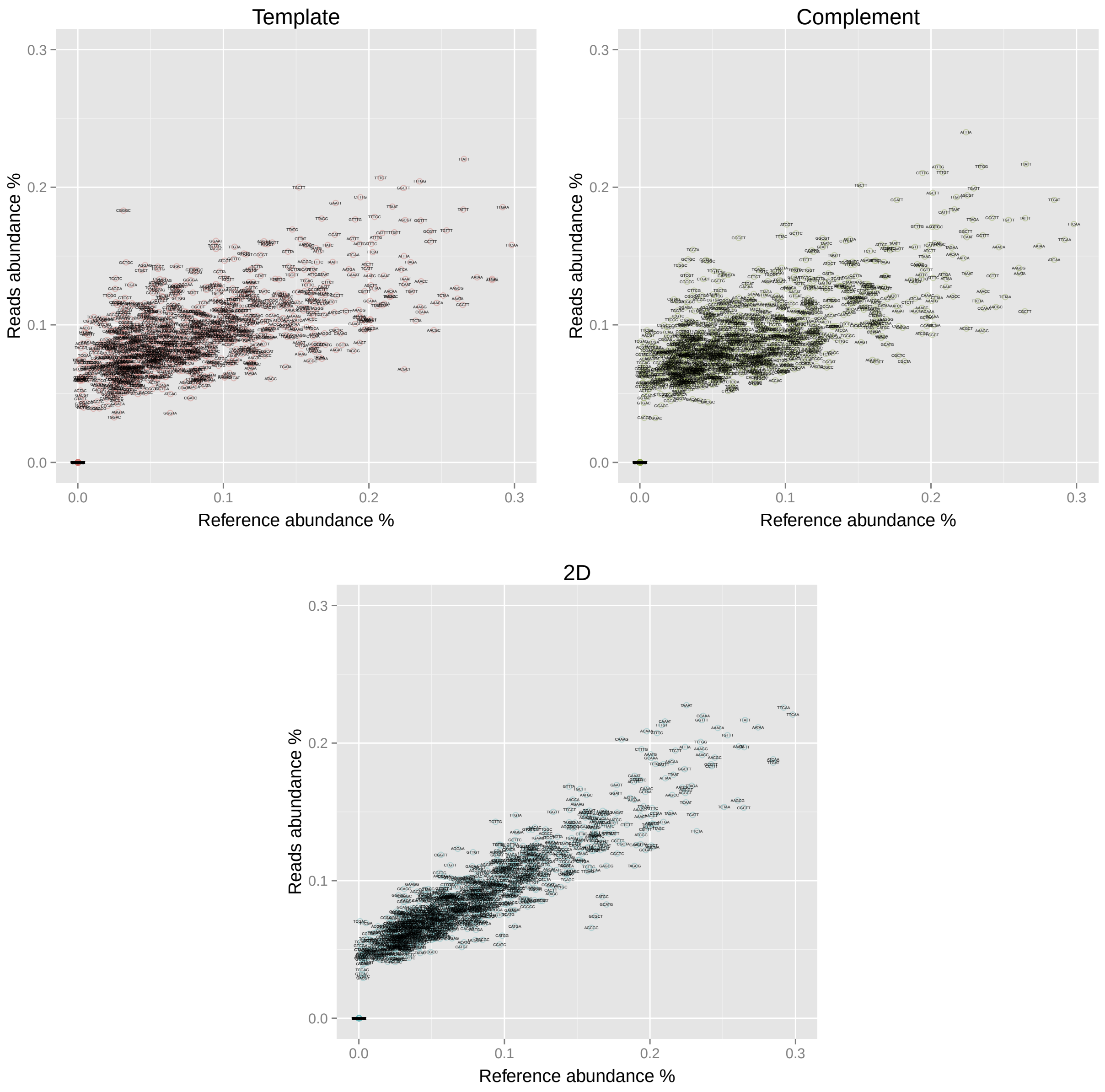

Helicobacter pylori GC content

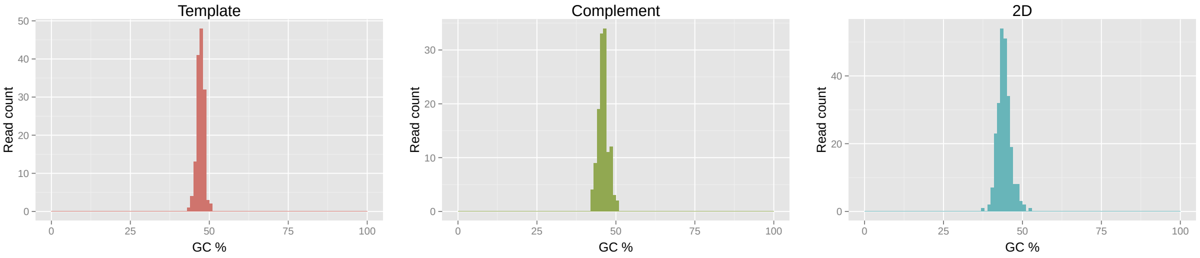

Lactobacillus gasseri error analysis

	Template	Complement	2D
Overall base identity (excluding indels)	68.52%	66.74%	76.29%
Aligned base identity (excluding indels)	76.19%	79.31%	85.29%
Identical bases per 100 aligned bases (including indels)	63.29%	64.89%	74.78%
Inserted bases per 100 aligned bases (including indels)	3.62%	2.40%	6.77%
Deleted bases per 100 aligned bases (including indels)	13.32%	15.78%	5.54%
Substitutions per 100 aligned bases (including indels)	19.77%	16.93%	12.90%
Mean insertion size	1.48	1.39	1.59
Mean deletion size	1.73	1.81	1.41

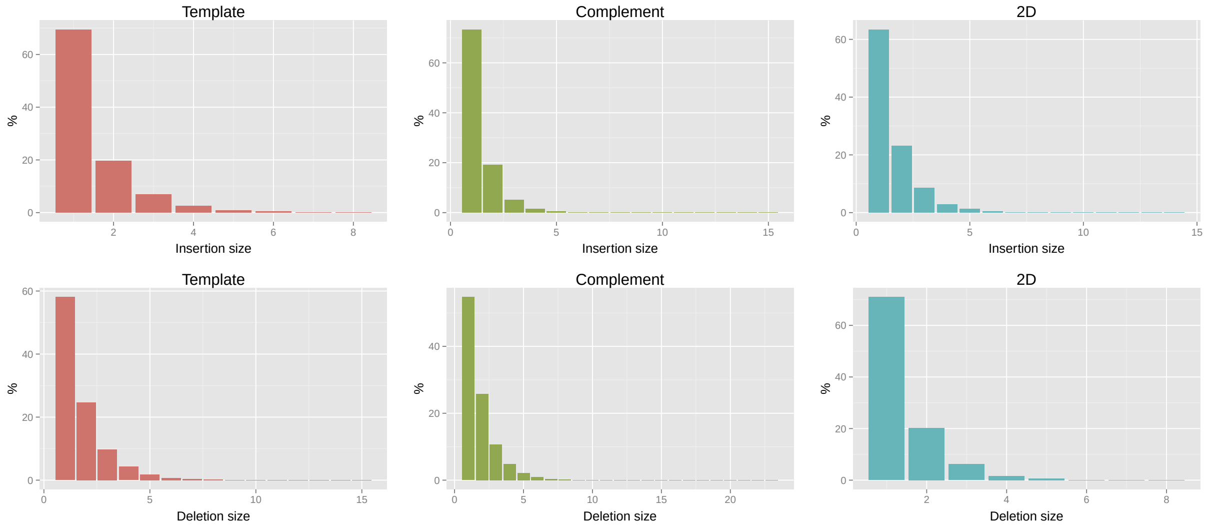

Lactobacillus gasseri read identity

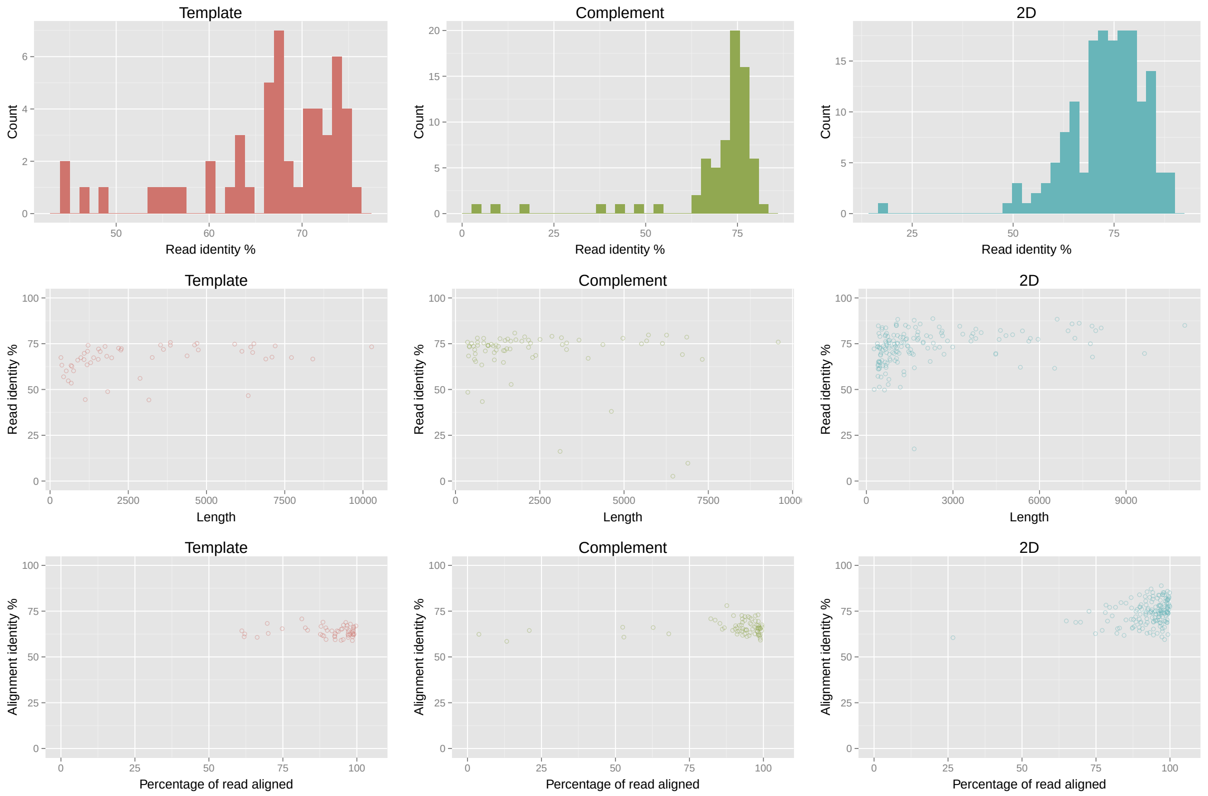

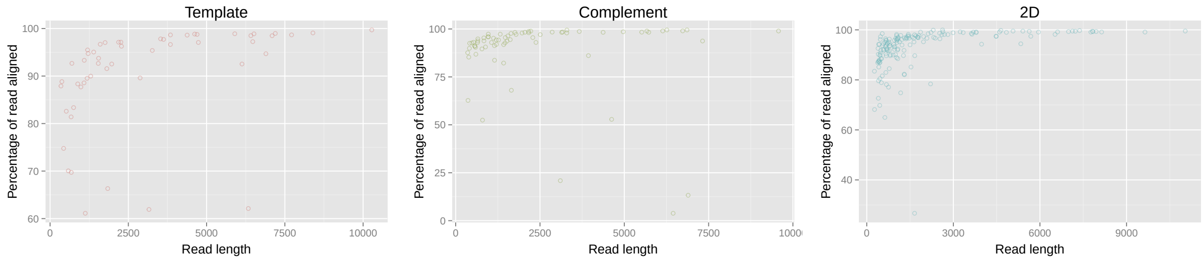

Lactobacillus gasseri perfect kmers

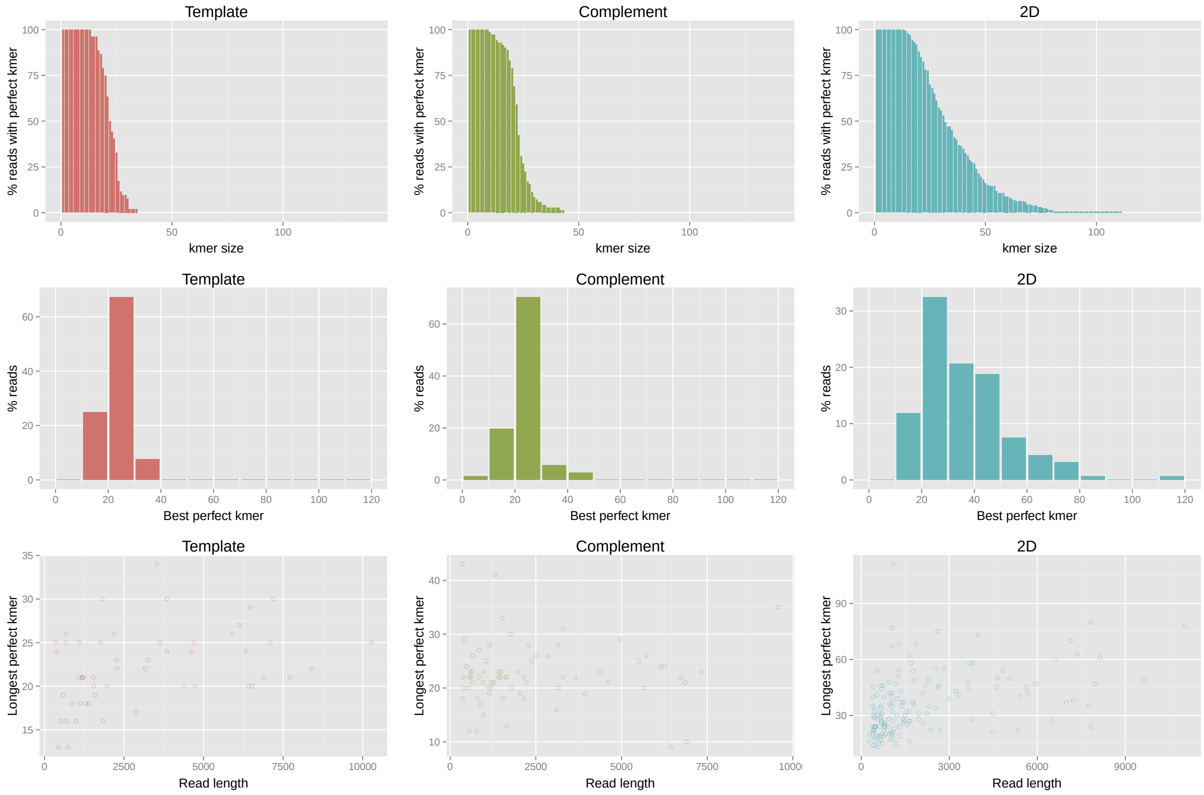

Lactobacillus gasseri coverage

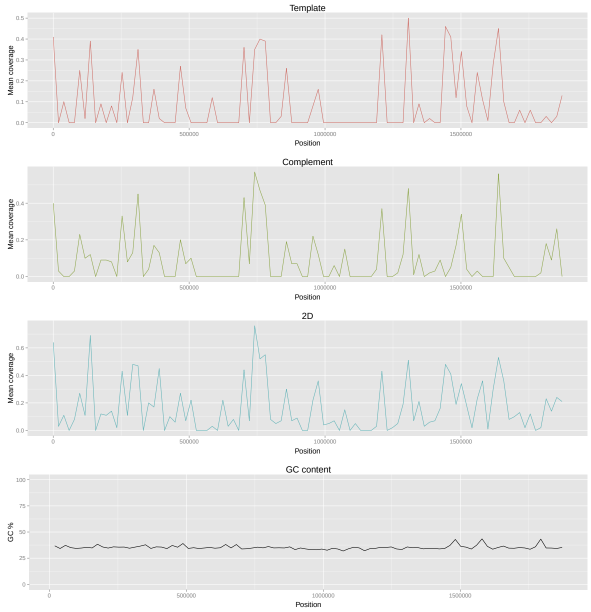

Lactobacillus gasseri 5-mer analysis

Under-represented 5-mers

	Template				Complement				2D			
Rank	kmer	Ref %	Read %	Diff %	kmer	Ref %	Read %	Diff %	kmer	Ref %	Read %	Diff %
1	AAAAA	0.552	0.085	-0.466	AAAAA	0.552	0.099	-0.453	TTTTT	0.535	0.109	-0.426
2	TTTTT	0.535	0.106	-0.429	TTTTT	0.535	0.094	-0.441	AAAAA	0.552	0.135	-0.417
3	TAAAA	0.438	0.076	-0.362	AAAAT	0.485	0.168	-0.317	AAAAT	0.485	0.219	-0.265
4	AAAAT	0.485	0.132	-0.353	TAAAA	0.438	0.146	-0.292	TAAAA	0.438	0.184	-0.254
5	AAATT	0.440	0.125	-0.315	ATTTT	0.465	0.174	-0.290	TTTTA	0.429	0.190	-0.239
6	TTAAA	0.425	0.116	-0.309	AAATT	0.440	0.158	-0.282	ATTTT	0.465	0.235	-0.229
7	AAAAG	0.365	0.091	-0.273	TTAAA	0.425	0.149	-0.276	AATTA	0.446	0.234	-0.212
8	AAGAA	0.386	0.119	-0.267	AAAAG	0.365	0.092	-0.273	AAAAG	0.365	0.156	-0.209
9	AATTA	0.446	0.180	-0.266	AAAGA	0.355	0.106	-0.250	TTAAA	0.425	0.220	-0.205
10	AAAGA	0.355	0.095	-0.261	TTTTA	0.429	0.183	-0.246	TTTAA	0.413	0.217	-0.196

Over-represented 5-mers

	Template				Complement				2D			
Rank	kmer	Ref %	Read %	Diff %	kmer	Ref %	Read %	Diff %	kmer	Ref %	Read %	Diff %
1	CGGGC	0.010	0.179	0.170	TCGTA	0.054	0.187	0.132	CGGGC	0.010	0.074	0.064
2	CTTCG	0.045	0.157	0.112	TCGGC	0.028	0.155	0.126	CATGC	0.053	0.111	0.058
3	ATGCG	0.042	0.152	0.111	CGGCT	0.036	0.161	0.125	GCATG	0.054	0.110	0.057
4	TCGTC	0.050	0.158	0.108	CGTAT	0.040	0.152	0.112	GGGCA	0.031	0.087	0.056
5	TGCGT	0.043	0.150	0.107	GCGGC	0.026	0.134	0.109	CGCAG	0.029	0.083	0.053
6	CGTGC	0.032	0.135	0.103	GCGTA	0.039	0.144	0.106	AATGC	0.131	0.184	0.052
7	GCGTG	0.029	0.128	0.099	CTTCG	0.045	0.150	0.105	CGGTT	0.043	0.095	0.052
8	GCTGC	0.090	0.189	0.099	CGTAG	0.031	0.133	0.101	GGCAG	0.046	0.096	0.050
9	GGCTG	0.051	0.149	0.099	GCGTG	0.029	0.129	0.100	CAGTC	0.047	0.098	0.050
10	CGGCT	0.036	0.131	0.096	TGCGT	0.043	0.141	0.098	CGACC	0.024	0.074	0.050

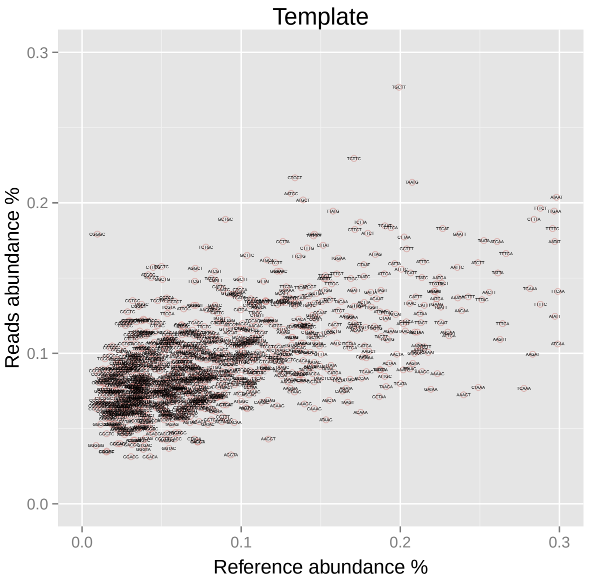

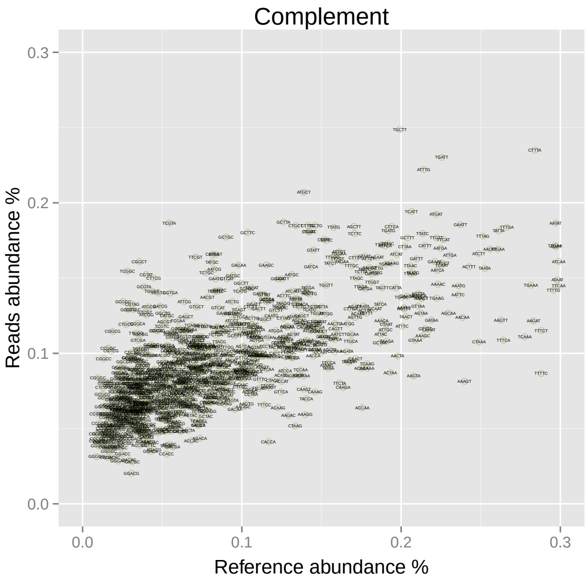

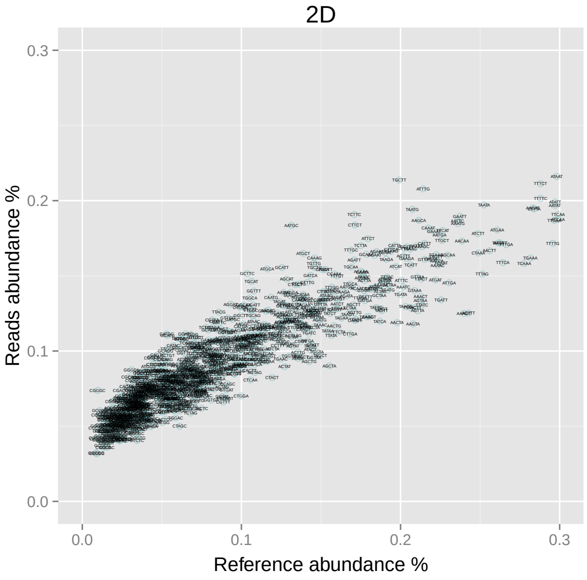

Lactobacillus gasseri GC content

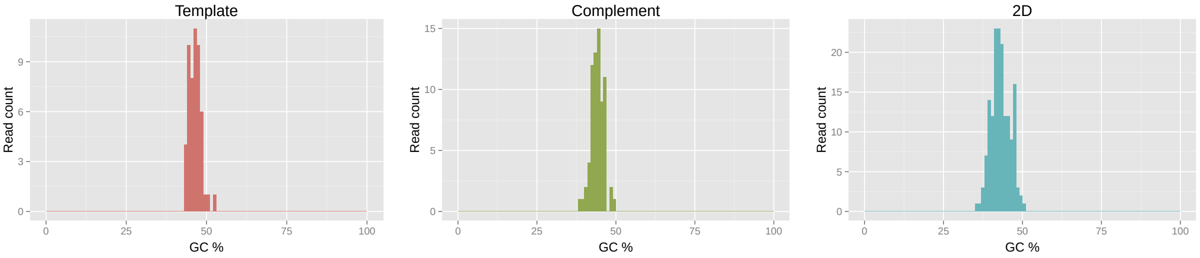

Listeria monocytogenes error analysis

	Template	Complement	2D
Overall base identity (excluding indels)	65.27%	68.65%	78.77%
Aligned base identity (excluding indels)	77.20%	79.51%	87.14%
Identical bases per 100 aligned bases (including indels)	64.18%	64.13%	77.33%
Inserted bases per 100 aligned bases (including indels)	3.34%	1.97%	5.59%
Deleted bases per 100 aligned bases (including indels)	13.52%	17.37%	5.66%
Substitutions per 100 aligned bases (including indels)	18.96%	16.53%	11.41%
Mean insertion size	1.43	1.32	1.52
Mean deletion size	1.72	1.87	1.43

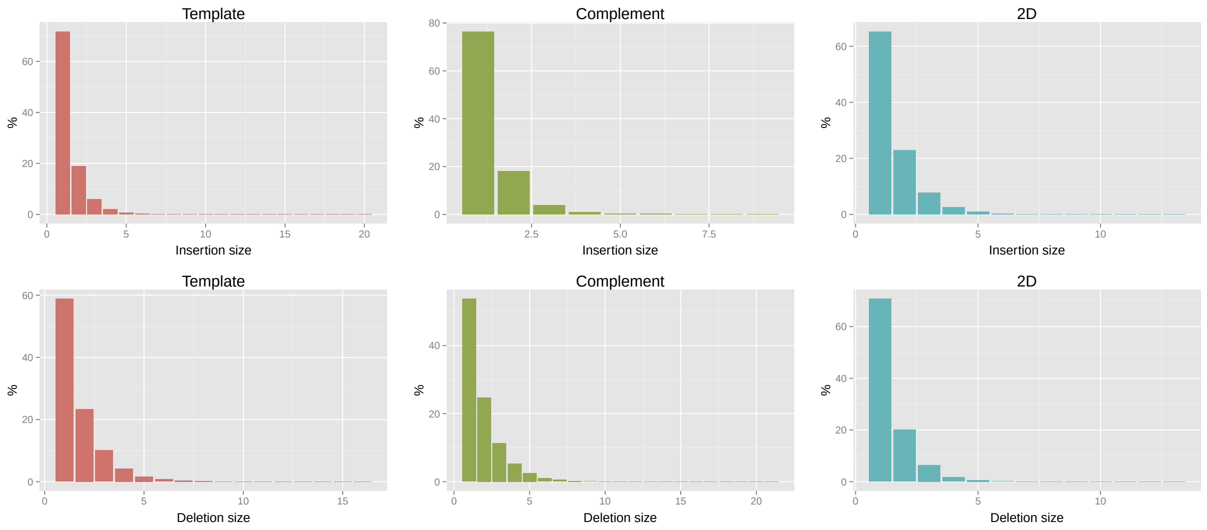

Listeria monocytogenes read identity

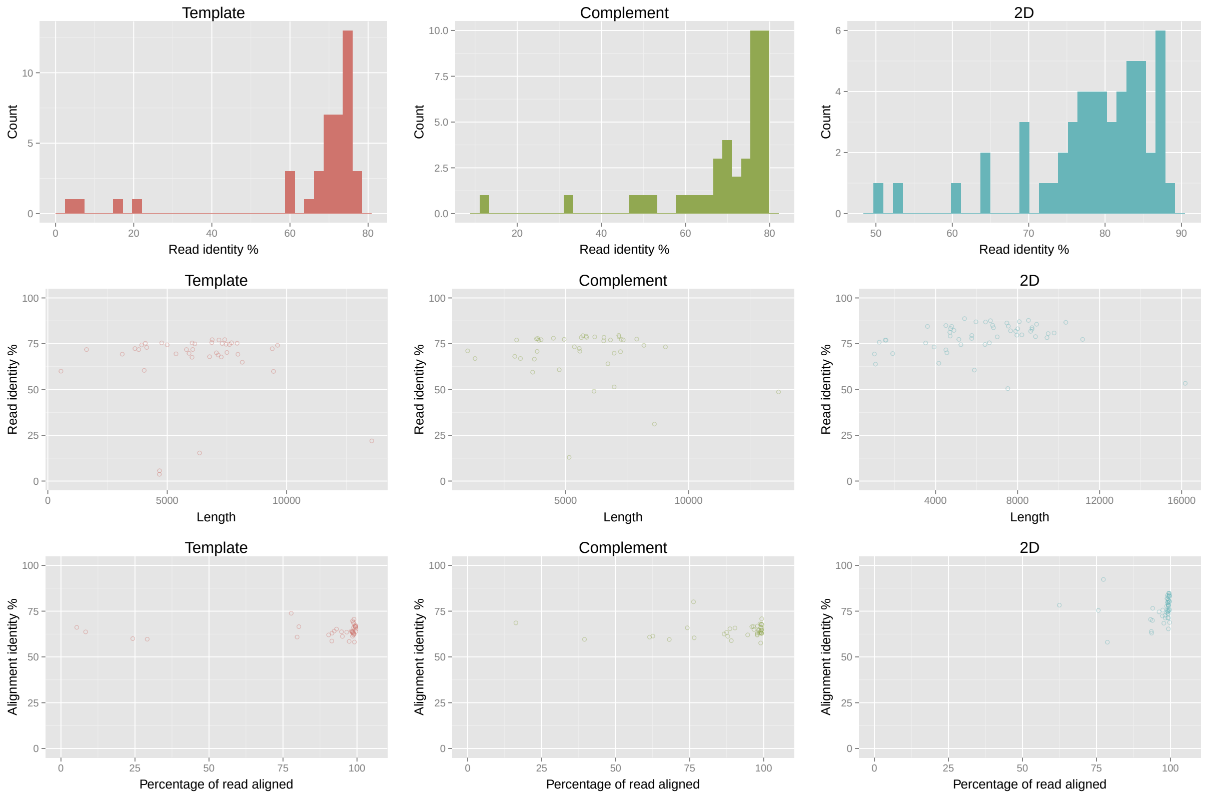

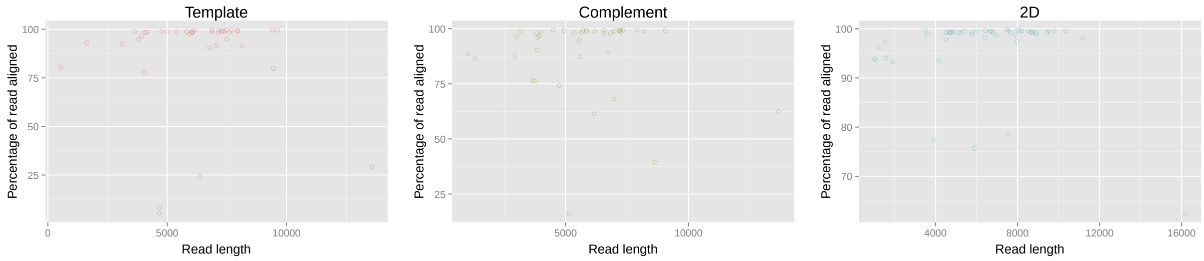

Listeria monocytogenes perfect kmers

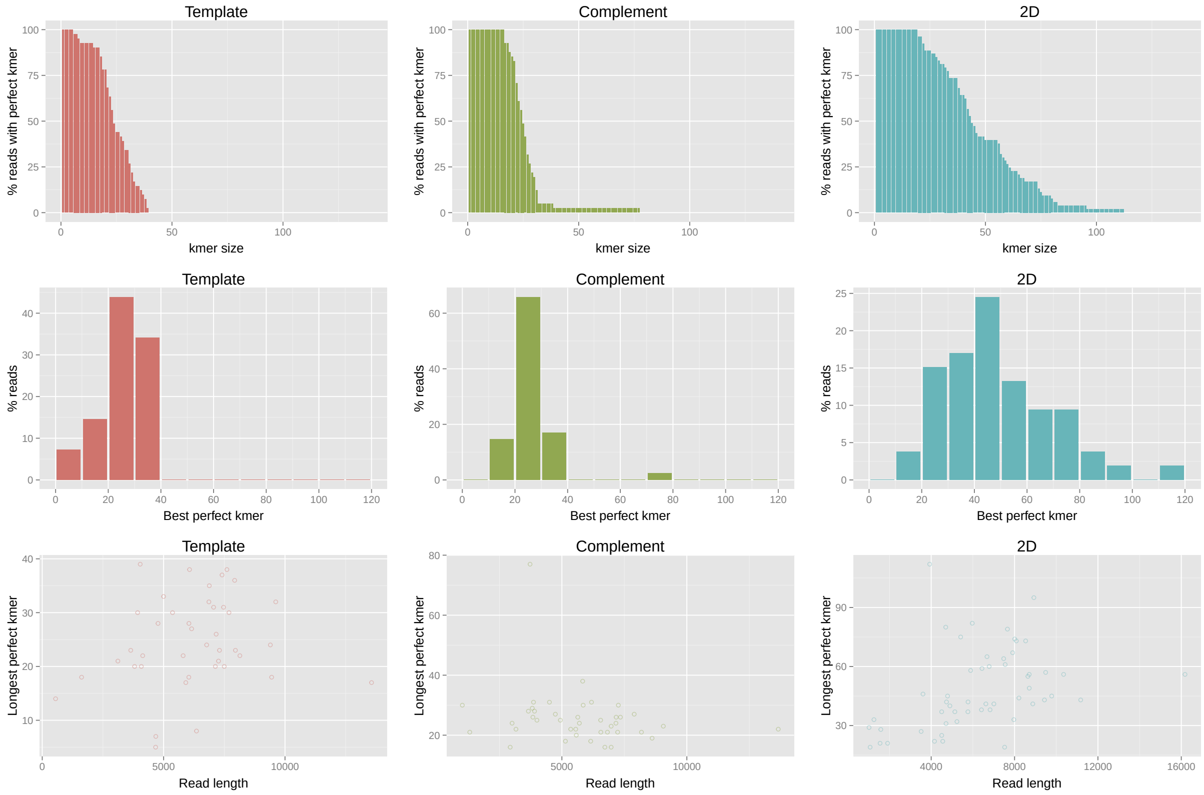

Listeria monocytogenes coverage

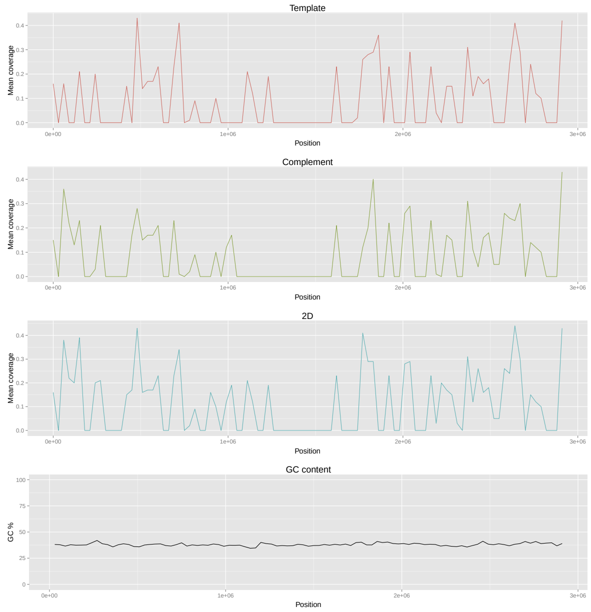

Listeria monocytogenes 5-mer analysis

Under-represented 5-mers

	Template				Complement				2D			
Rank	kmer	Ref %	Read %	Diff %	kmer	Ref %	Read %	Diff %	kmer	Ref %	Read %	Diff %
1	AAAAA	0.653	0.110	-0.543	TTTTT	0.639	0.088	-0.551	AAAAA	0.653	0.153	-0.499
2	TTTTT	0.639	0.134	-0.506	AAAAA	0.653	0.106	-0.547	TTTTT	0.639	0.143	-0.497
3	AAAAT	0.489	0.151	-0.338	ATTTT	0.491	0.154	-0.336	AAAAT	0.489	0.256	-0.233
4	TAAAA	0.413	0.082	-0.331	AAAAT	0.489	0.166	-0.323	TTTTA	0.407	0.184	-0.223
5	AAAAG	0.348	0.091	-0.257	TAAAA	0.413	0.123	-0.290	TAAAA	0.413	0.190	-0.223
6	ATTTT	0.491	0.239	-0.252	TTTTC	0.391	0.111	-0.279	ATTTT	0.491	0.274	-0.217
7	AAATT	0.366	0.120	-0.246	AAAAG	0.348	0.080	-0.268	AAAAG	0.348	0.158	-0.190
8	CAAAA	0.330	0.086	-0.244	TTTTA	0.407	0.151	-0.256	CTTTT	0.349	0.181	-0.168
9	ATAAA	0.327	0.085	-0.242	CAAAA	0.330	0.094	-0.236	GAAAA	0.388	0.227	-0.160
10	TTTTC	0.391	0.156	-0.234	AAATT	0.366	0.141	-0.226	AATTT	0.365	0.216	-0.149

Over-represented 5-mers

	Template				Complement				2D			
Rank	kmer	Ref %	Read %	Diff %	kmer	Ref %	Read %	Diff %	kmer	Ref %	Read %	Diff %
1	CGGGC	0.021	0.188	0.167	TCGTA	0.082	0.215	0.133	CGGGC	0.021	0.071	0.050
2	GAGAG	0.032	0.151	0.120	CGGCT	0.053	0.174	0.121	GCCCG	0.021	0.068	0.046
3	AGGCT	0.044	0.152	0.109	TCGGC	0.053	0.172	0.119	CCGAC	0.031	0.077	0.046
4	TCGTC	0.070	0.169	0.099	GTCGT	0.056	0.168	0.112	AGGCC	0.020	0.064	0.045
5	GTCGT	0.056	0.151	0.096	TGATC	0.056	0.164	0.108	GCAGG	0.042	0.087	0.044
6	GGCTC	0.035	0.130	0.095	GATCG	0.027	0.134	0.107	CACGA	0.062	0.106	0.044
7	GGGCT	0.035	0.129	0.094	TCGTG	0.060	0.156	0.096	ATGCT	0.111	0.155	0.044
8	CGGCT	0.053	0.147	0.094	CGTCG	0.035	0.128	0.093	GGATC	0.039	0.082	0.043
9	GCTGC	0.078	0.173	0.094	ATCGT	0.106	0.196	0.090	CGTAG	0.040	0.083	0.043
10	TAGGC	0.034	0.128	0.094	TCGTC	0.070	0.159	0.089	GCATG	0.056	0.099	0.043

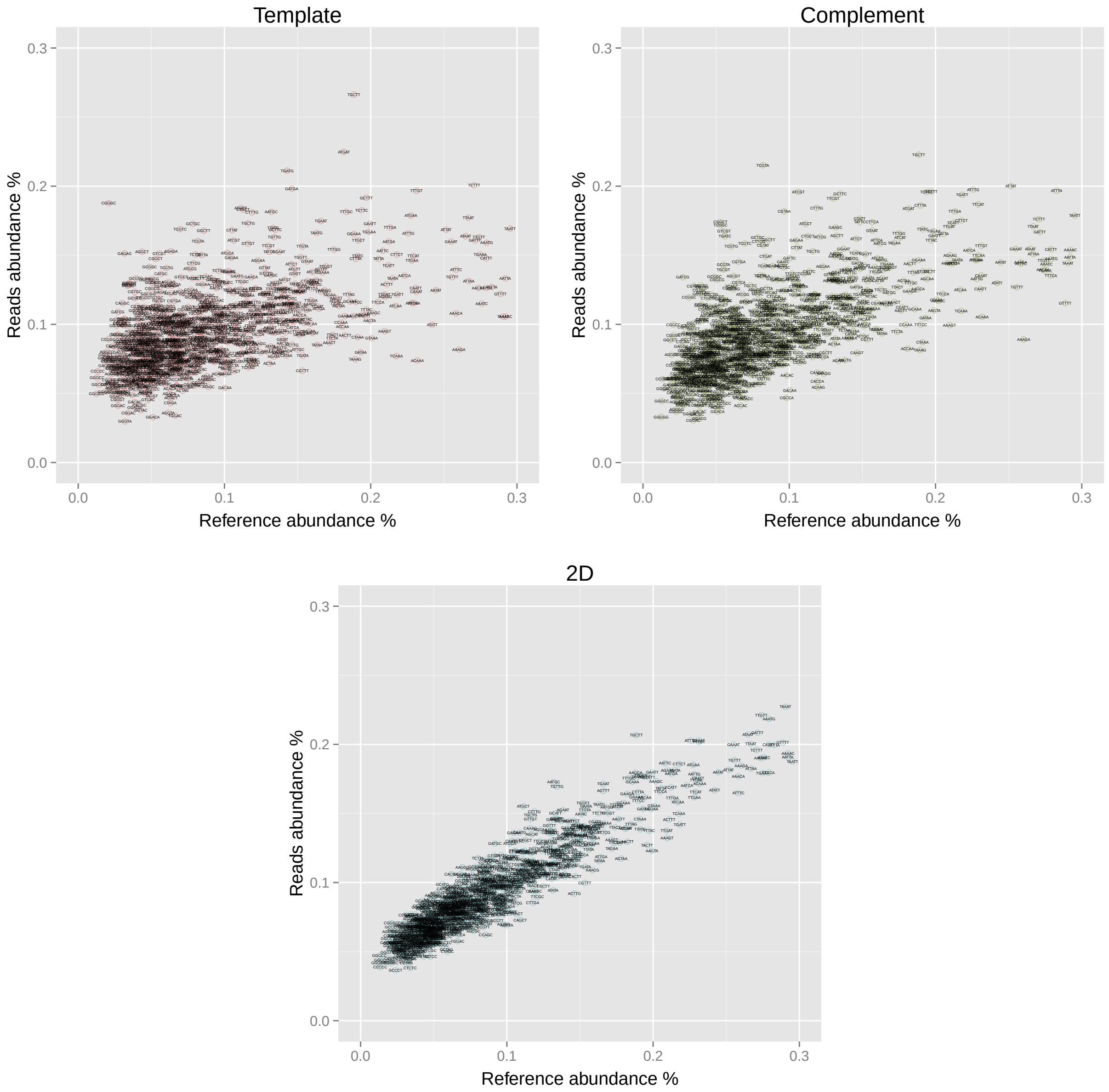

Listeria monocytogenes GC content

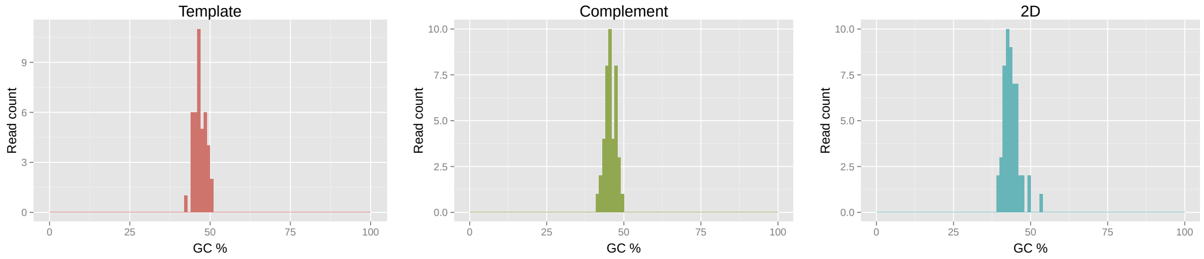

Neisseria meningitidis error analysis

	Template	Complement	2D
Overall base identity (excluding indels)	69.08%	72.88%	77.48%
Aligned base identity (excluding indels)	78.33%	80.66%	87.47%
Identical bases per 100 aligned bases (including indels)	65.59%	65.82%	77.19%
Inserted bases per 100 aligned bases (including indels)	3.74%	2.47%	6.82%
Deleted bases per 100 aligned bases (including indels)	12.52%	15.93%	4.93%
Substitutions per 100 aligned bases (including indels)	18.15%	15.78%	11.06%
Mean insertion size	1.47	1.38	1.61
Mean deletion size	1.68	1.84	1.42

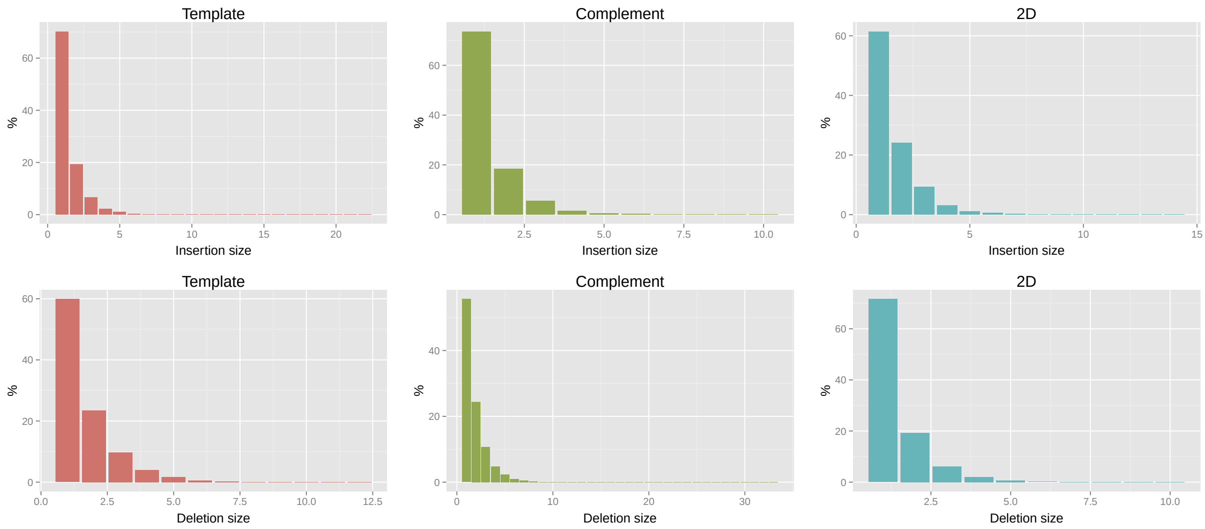

Neisseria meningitidis read identity

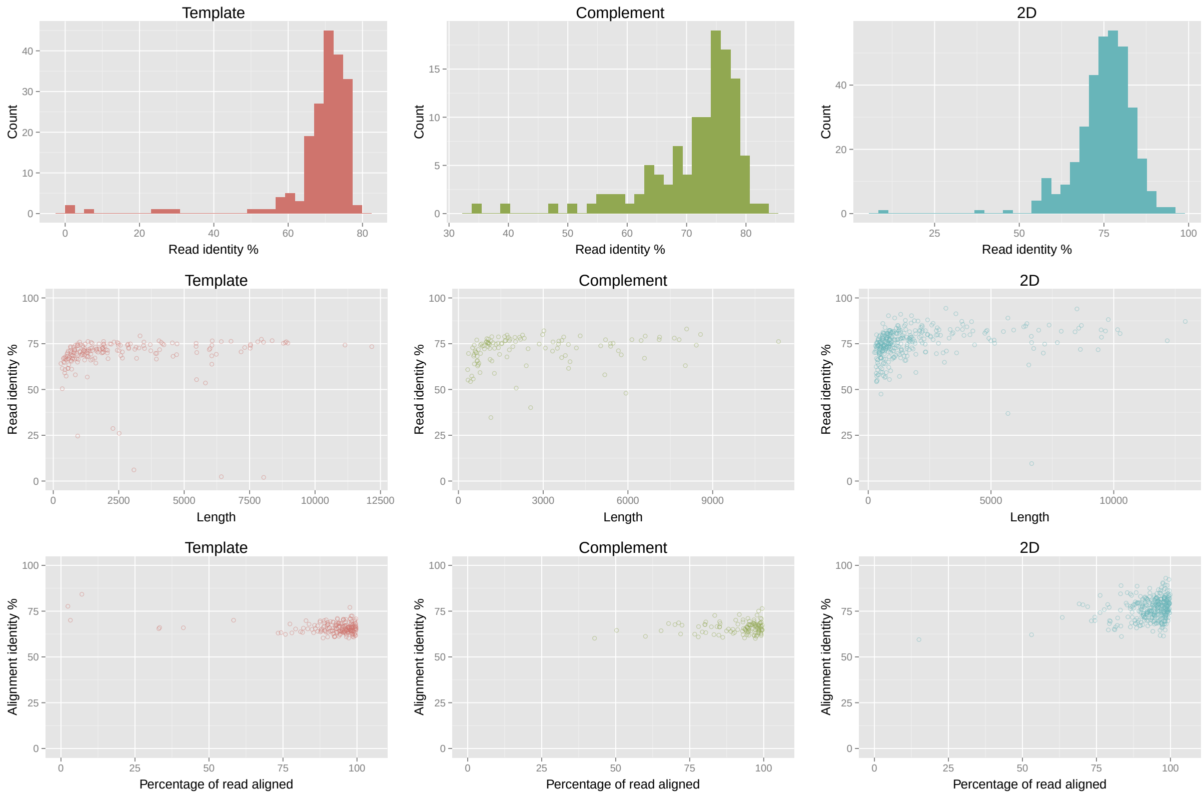

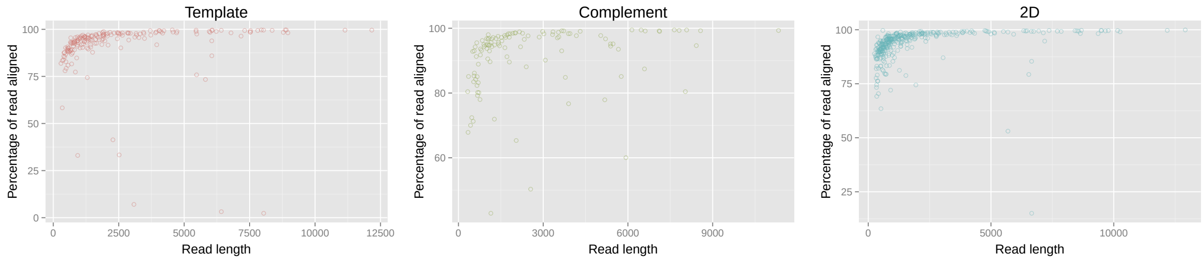

Neisseria meningitidis perfect kmers

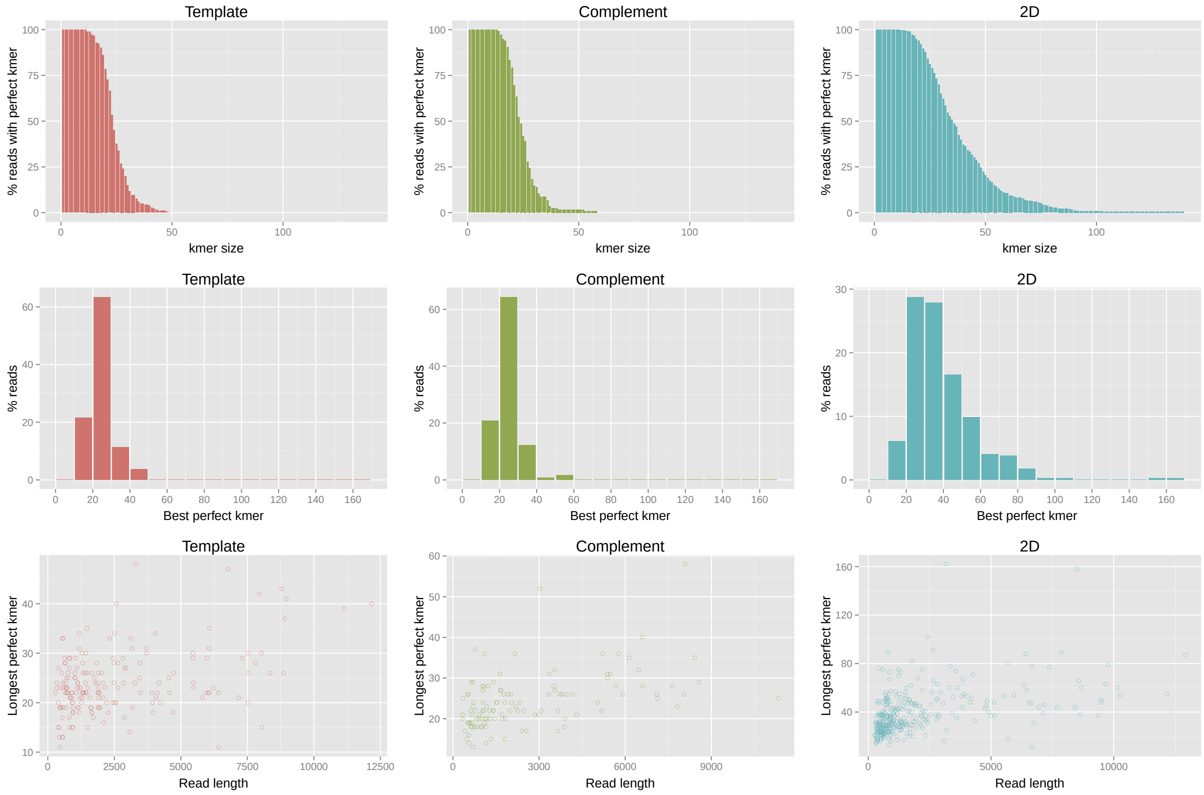

Neisseria meningitidis coverage

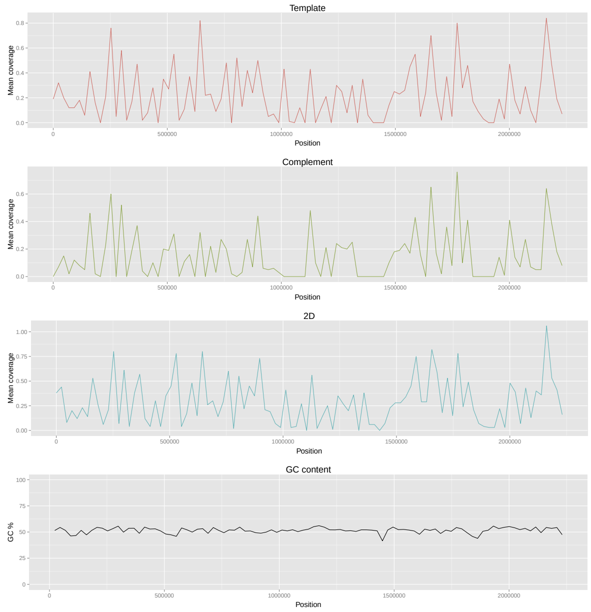

Neisseria meningitidis 5-mer analysis

Under-represented 5-mers

	Template				Complement				2D			
Rank	kmer	Ref %	Read %	Diff %	kmer	Ref %	Read %	Diff %	kmer	Ref %	Read %	Diff %
1	AAAAA	0.387	0.099	-0.288	CCGCC	0.393	0.104	-0.289	TTTTT	0.377	0.126	-0.250
2	TGCCG	0.436	0.163	-0.273	AAAAA	0.387	0.098	-0.289	AAAAA	0.387	0.138	-0.248
3	CGCCG	0.384	0.122	-0.262	TTTTT	0.377	0.095	-0.282	TGCCG	0.436	0.255	-0.181
4	CCGCC	0.393	0.146	-0.247	CGCCG	0.384	0.106	-0.278	CGGCA	0.420	0.244	-0.176
5	TTTTT	0.377	0.130	-0.247	TGCCG	0.436	0.171	-0.265	CCGCC	0.393	0.219	-0.175
6	GCCGC	0.364	0.127	-0.237	GCCGC	0.364	0.130	-0.234	GGCGG	0.403	0.241	-0.162
7	CAAAA	0.303	0.100	-0.203	GGCGG	0.403	0.196	-0.207	CGGCG	0.397	0.245	-0.152
8	CGGCA	0.420	0.231	-0.189	CAAAA	0.303	0.100	-0.203	CGCCG	0.384	0.235	-0.149
9	GGCGG	0.403	0.220	-0.183	CGGCA	0.420	0.227	-0.193	GCCGC	0.364	0.215	-0.149
10	AAAAC	0.295	0.121	-0.174	TTGCC	0.302	0.111	-0.191	ACGGC	0.288	0.162	-0.126

Over-represented 5-mers

	Template				Complement				2D			
Rank	kmer	Ref %	Read %	Diff %	kmer	Ref %	Read %	Diff %	kmer	Ref %	Read %	Diff %
1	TAGGC	0.044	0.138	0.093	TCGTA	0.055	0.149	0.094	TAGTT	0.025	0.083	0.058
2	CATGC	0.052	0.141	0.088	GATCG	0.034	0.127	0.093	TAGCA	0.020	0.078	0.058
3	TGCTT	0.112	0.199	0.086	TGATC	0.030	0.119	0.088	GTTAG	0.015	0.069	0.054
4	ACGTC	0.057	0.140	0.083	CGTGA	0.055	0.141	0.086	GCTAG	0.005	0.059	0.054
5	GTTGT	0.094	0.177	0.083	GTCGT	0.097	0.180	0.083	CTAAC	0.013	0.064	0.051
6	TAGTT	0.025	0.107	0.082	TGAGC	0.064	0.146	0.082	TTAGA	0.037	0.087	0.050
7	TCTTA	0.028	0.110	0.082	TCGTG	0.068	0.148	0.080	TTTAG	0.049	0.098	0.050
8	CACGT	0.042	0.123	0.081	GGCTC	0.042	0.120	0.078	TTAGC	0.026	0.075	0.049
9	GGGAG	0.032	0.112	0.081	AGAGC	0.034	0.111	0.077	CATGC	0.052	0.101	0.049
10	GCTTA	0.040	0.118	0.078	CACGT	0.042	0.117	0.075	TGCTA	0.023	0.071	0.048

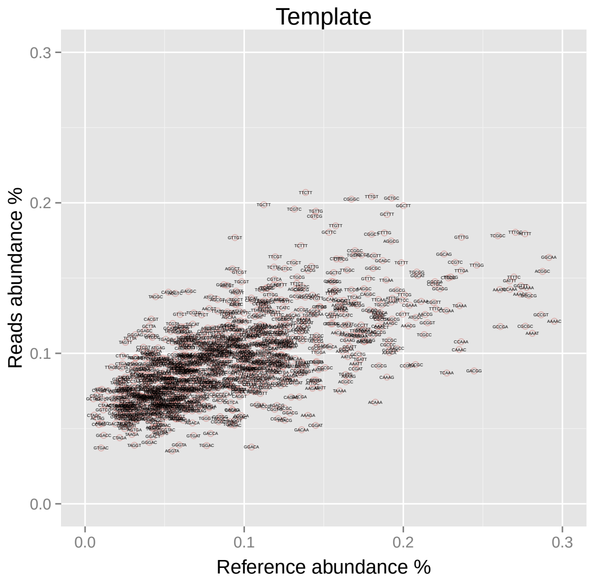

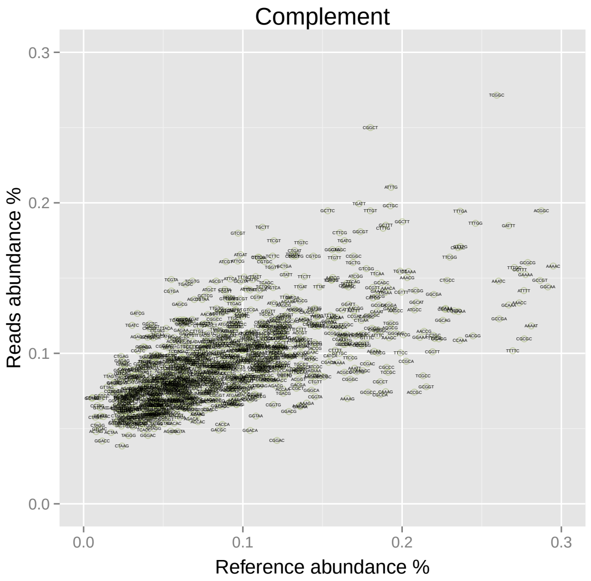

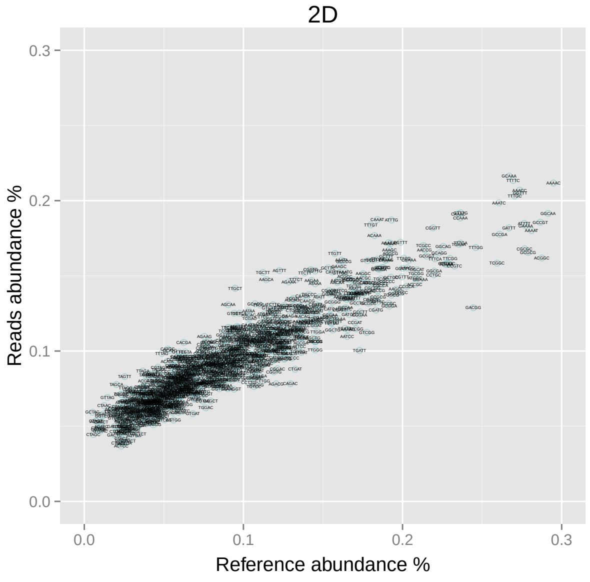

Neisseria meningitidis GC content

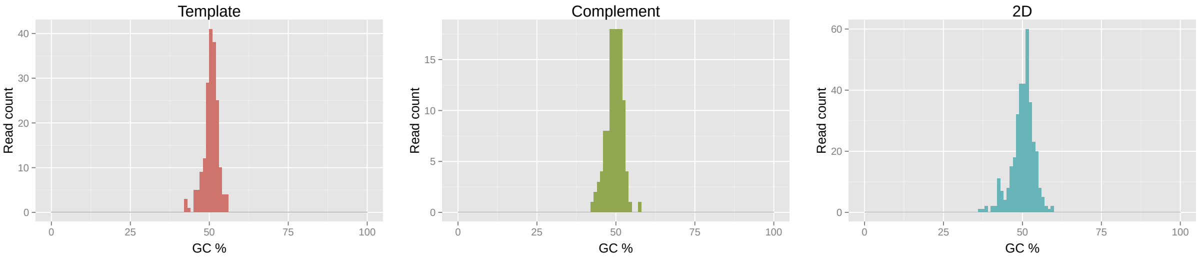

Propionibacterium acnes error analysis

	Template	Complement	2D
Overall base identity (excluding indels)	68.33%	70.91%	77.63%
Aligned base identity (excluding indels)	76.90%	78.71%	87.43%
Identical bases per 100 aligned bases (including indels)	64.26%	64.16%	76.86%
Inserted bases per 100 aligned bases (including indels)	4.36%	2.90%	7.97%
Deleted bases per 100 aligned bases (including indels)	12.08%	15.58%	4.11%
Substitutions per 100 aligned bases (including indels)	19.30%	17.36%	11.05%
Mean insertion size	1.48	1.39	1.69
Mean deletion size	1.62	1.79	1.37

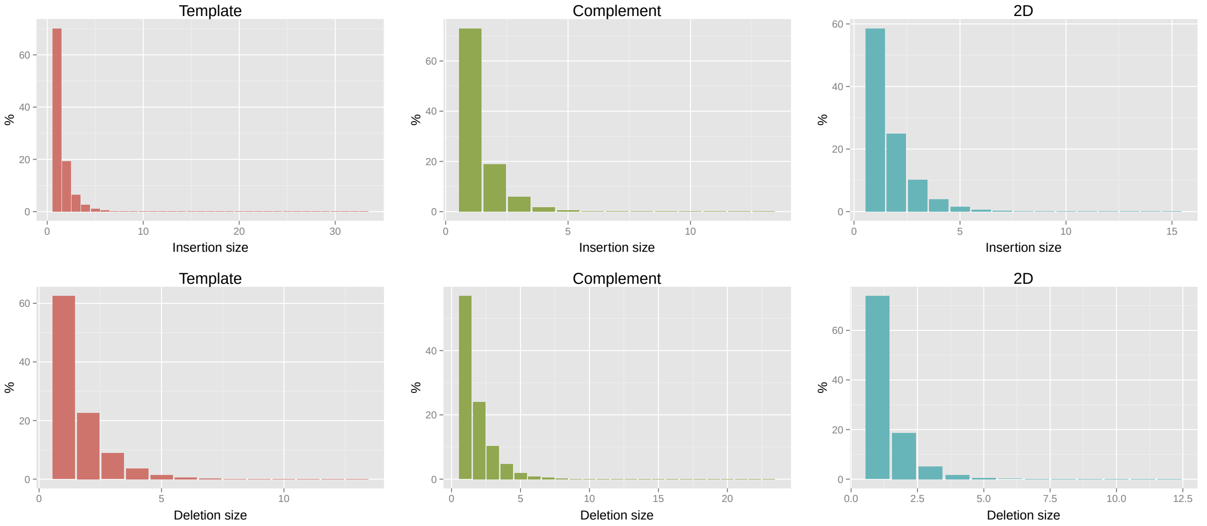

Propionibacterium acnes read identity

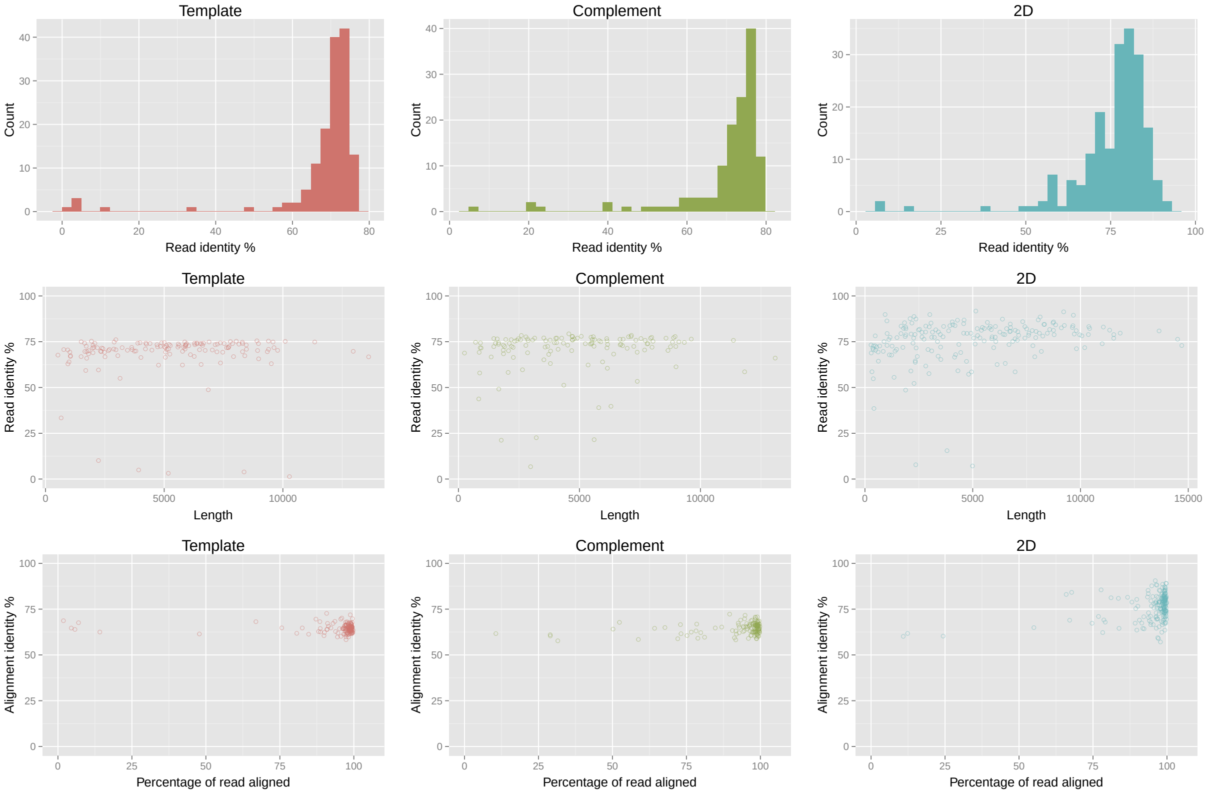

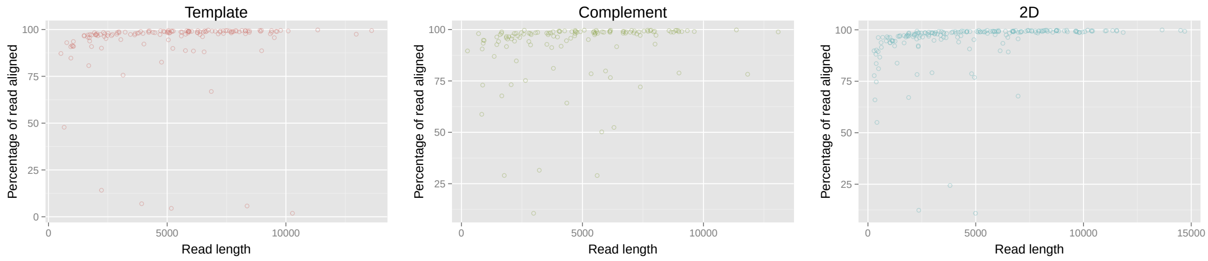

Propionibacterium acnes perfect kmers

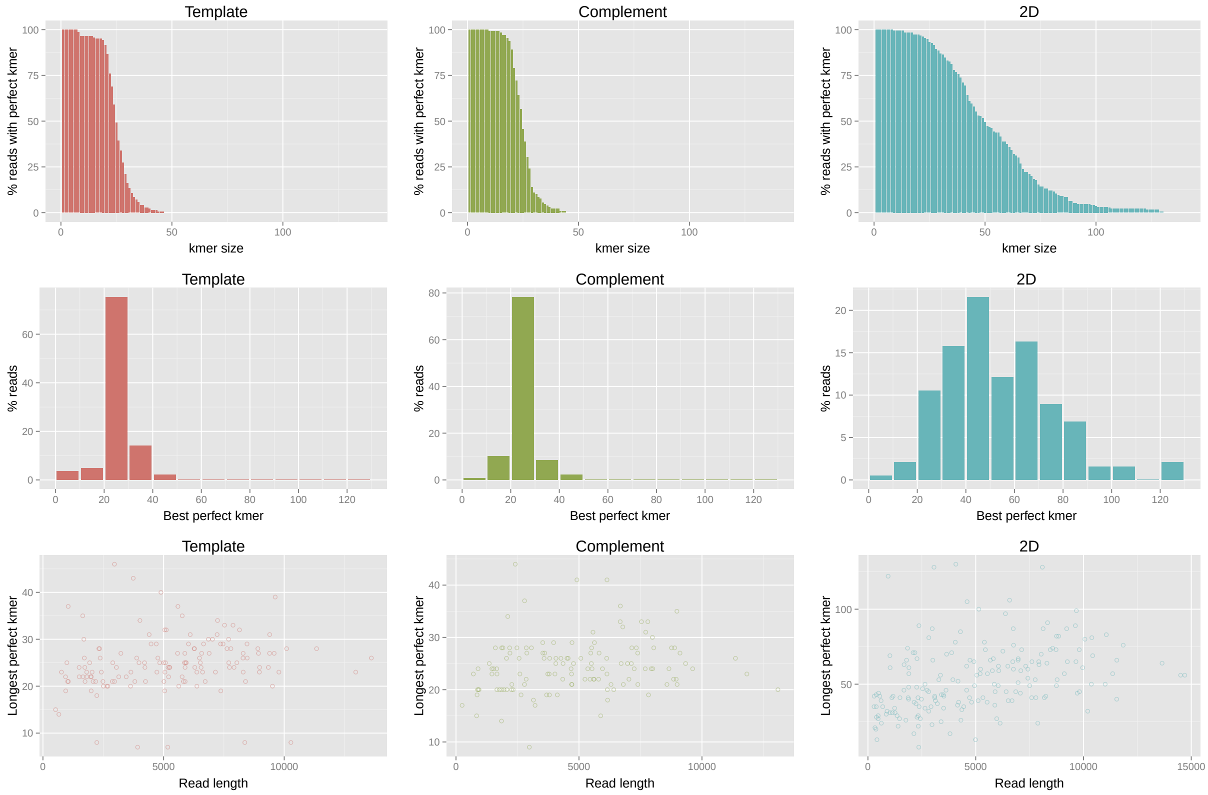

Propionibacterium acnes coverage

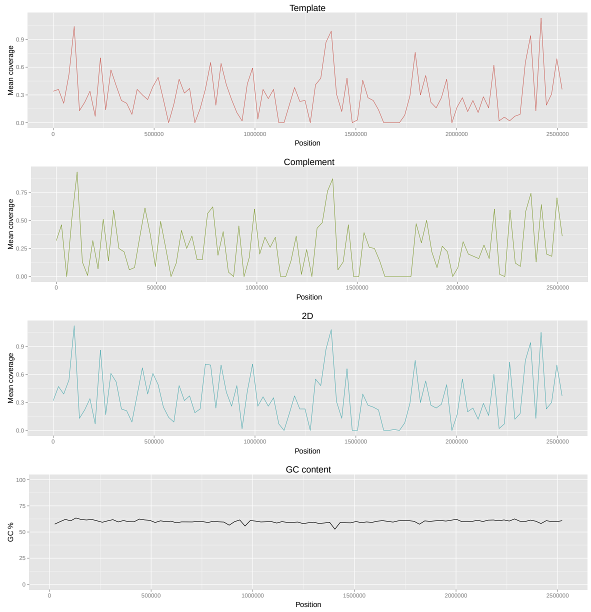

Propionibacterium acnes 5-mer analysis

Under-represented 5-mers

	Template				Complement				2D			
Rank	kmer	Ref %	Read %	Diff %	kmer	Ref %	Read %	Diff %	kmer	Ref %	Read %	Diff %
1	CGACG	0.383	0.147	-0.237	CGACG	0.383	0.123	-0.260	CGTCG	0.388	0.240	-0.148
2	GACGA	0.318	0.092	-0.226	GACGA	0.318	0.116	-0.202	CGACG	0.383	0.253	-0.130
3	CGGTG	0.276	0.086	-0.190	CGGTG	0.276	0.082	-0.194	CGGTG	0.276	0.151	-0.125
4	TGACG	0.278	0.091	-0.187	TGACG	0.278	0.102	-0.176	CACCG	0.271	0.160	-0.111
5	CGCCG	0.271	0.110	-0.162	CGCCG	0.271	0.103	-0.168	GACGA	0.318	0.208	-0.110
6	TCGAC	0.269	0.116	-0.153	CACCG	0.271	0.124	-0.147	TCGTC	0.327	0.228	-0.099
7	CGACC	0.233	0.082	-0.151	GGTGG	0.222	0.086	-0.137	CCAGC	0.219	0.124	-0.095
8	GGTGG	0.222	0.076	-0.146	TCGAC	0.269	0.135	-0.133	TGACG	0.278	0.184	-0.094
9	GGTGA	0.212	0.069	-0.143	CGAGG	0.246	0.115	-0.132	GGTGA	0.212	0.124	-0.088
10	GACGG	0.224	0.091	-0.133	GACGC	0.207	0.077	-0.130	TCGGC	0.250	0.164	-0.086

Over-represented 5-mers

	Template				Complement				2D			
Rank	kmer	Ref %	Read %	Diff %	kmer	Ref %	Read %	Diff %	kmer	Ref %	Read %	Diff %
1	TAGGC	0.044	0.136	0.092	TCGTA	0.060	0.174	0.114	TAGAG	0.030	0.090	0.060
2	TGCTT	0.064	0.155	0.091	CGTAA	0.041	0.135	0.094	TAACG	0.047	0.103	0.056
3	TGTGT	0.046	0.136	0.091	CGGCT	0.152	0.239	0.087	TAGAT	0.026	0.080	0.053
4	TATCT	0.035	0.124	0.089	CGTAT	0.041	0.128	0.087	ATCTA	0.025	0.077	0.052
5	TCTAA	0.015	0.101	0.086	AACGT	0.070	0.155	0.086	TAGTA	0.014	0.065	0.051
6	ATCTA	0.025	0.110	0.086	ATCTA	0.025	0.109	0.085	ATACG	0.043	0.094	0.051
7	ATAAT	0.023	0.108	0.085	CTAAT	0.016	0.099	0.083	TTAGA	0.014	0.065	0.051
8	TAGTA	0.014	0.098	0.084	GCGTA	0.059	0.141	0.082	TATAG	0.012	0.062	0.051
9	TAATA	0.014	0.097	0.083	TAATA	0.014	0.094	0.080	GTTAG	0.027	0.077	0.050
10	GCTTA	0.035	0.117	0.082	TAATC	0.028	0.107	0.079	GACTA	0.040	0.089	0.048

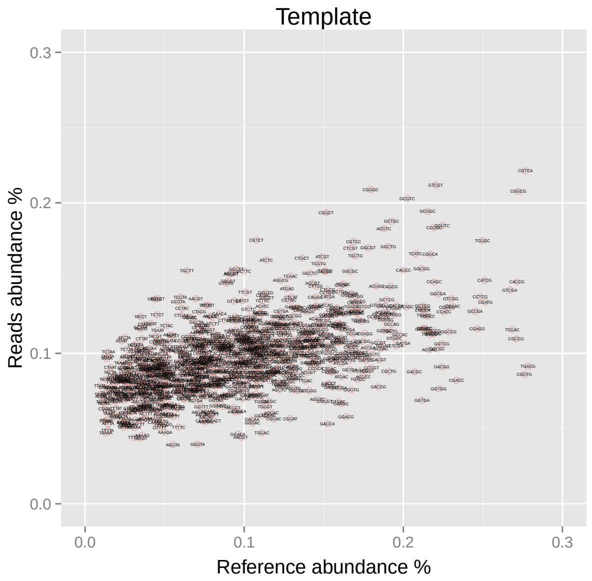

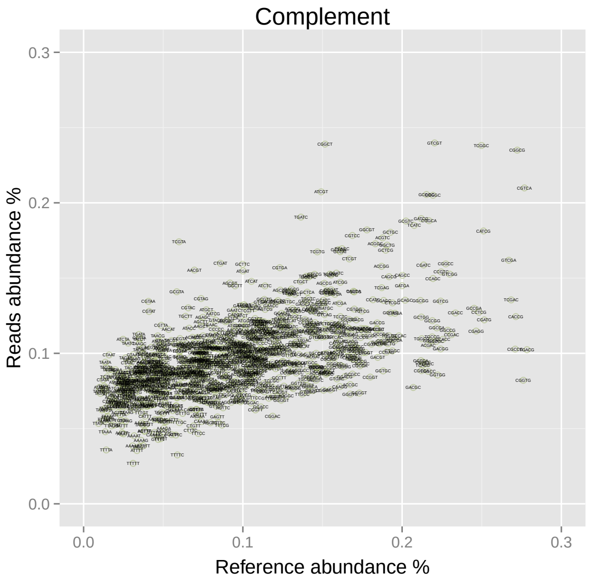

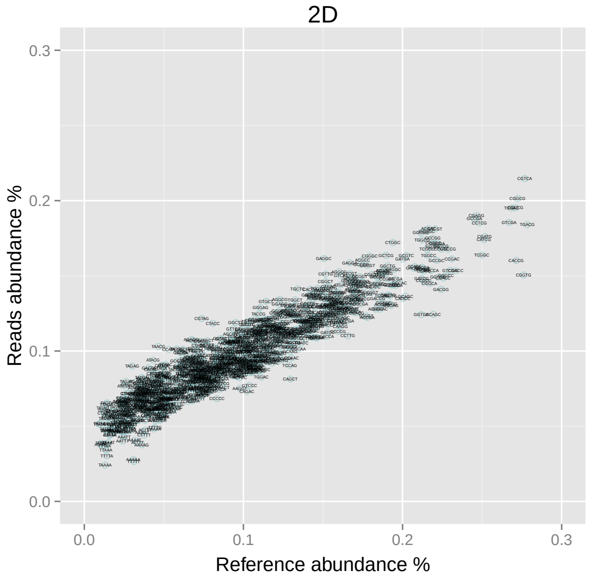

Propionibacterium acnes GC content

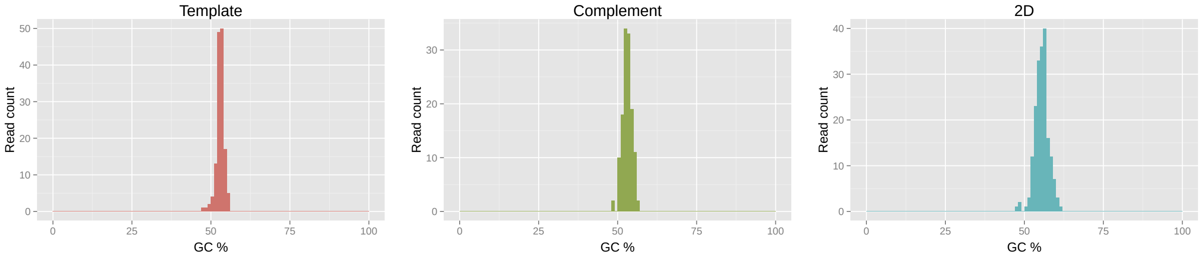

Pseudomonas aeruginosa error analysis

	Template	Complement	2D
Overall base identity (excluding indels)	65.37%	68.53%	72.12%
Aligned base identity (excluding indels)	73.92%	77.99%	83.95%
Identical bases per 100 aligned bases (including indels)	61.57%	63.34%	72.90%
Inserted bases per 100 aligned bases (including indels)	4.50%	2.76%	8.24%
Deleted bases per 100 aligned bases (including indels)	12.20%	16.02%	4.91%
Substitutions per 100 aligned bases (including indels)	21.73%	17.88%	13.94%
Mean insertion size	1.46	1.37	1.67
Mean deletion size	1.63	1.81	1.41

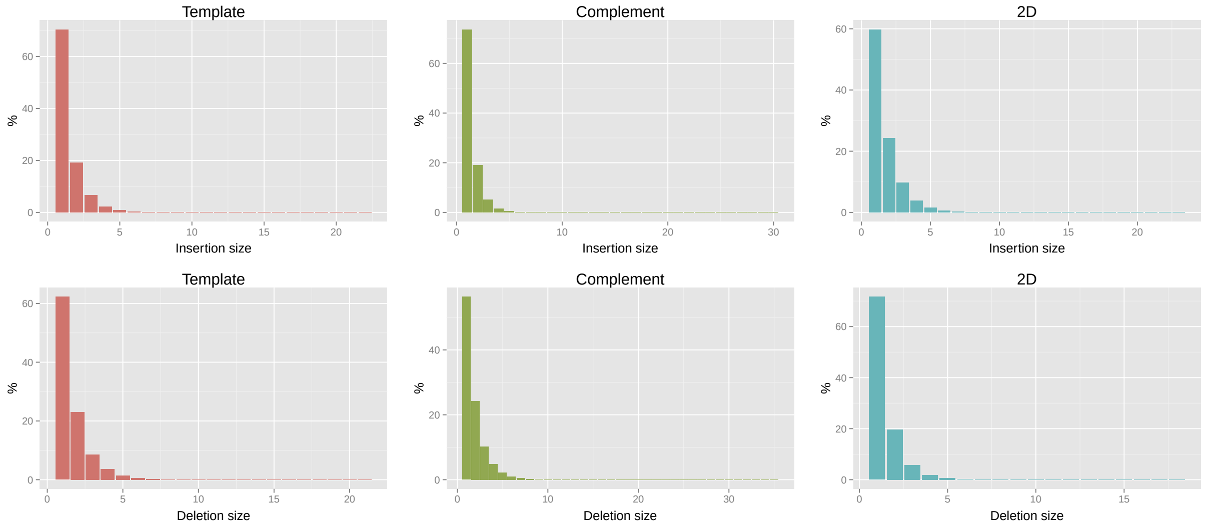

Pseudomonas aeruginosa read identity

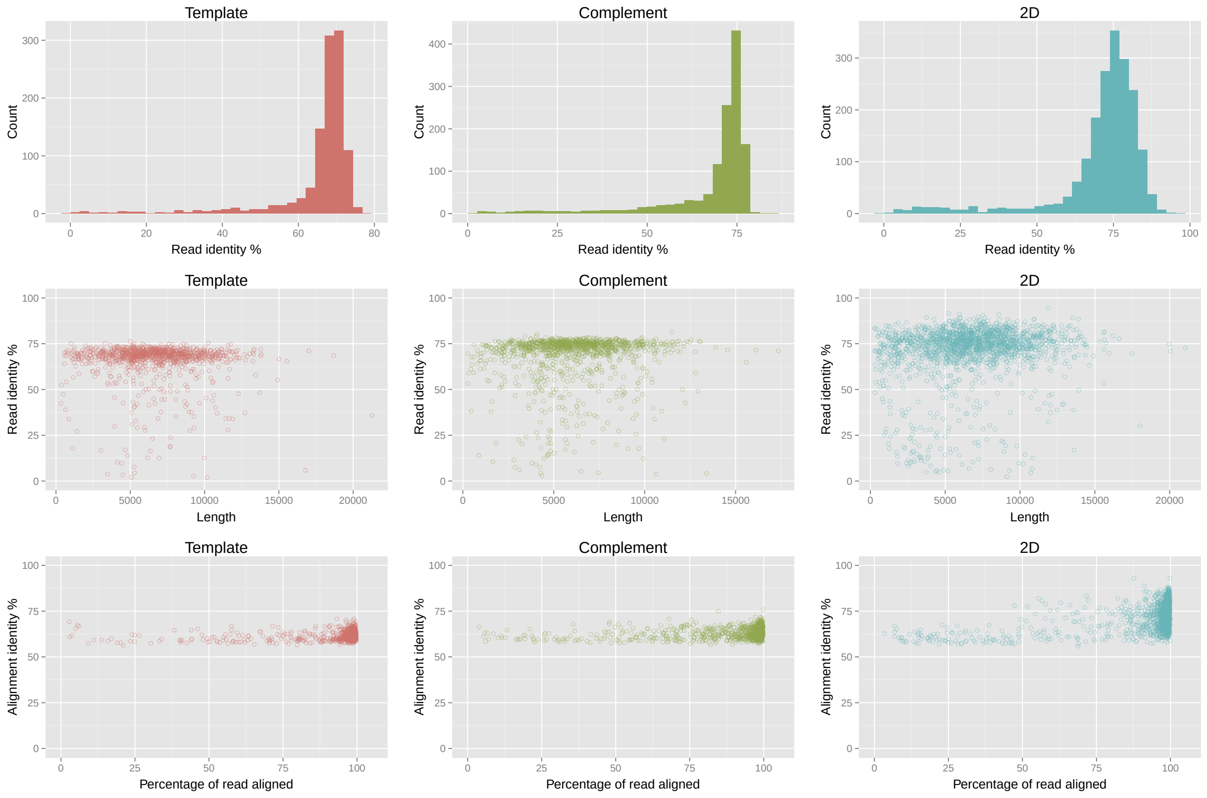

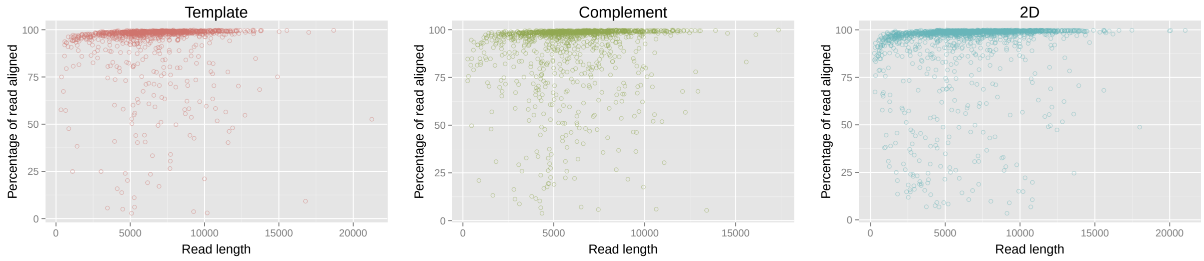

Pseudomonas aeruginosa perfect kmers

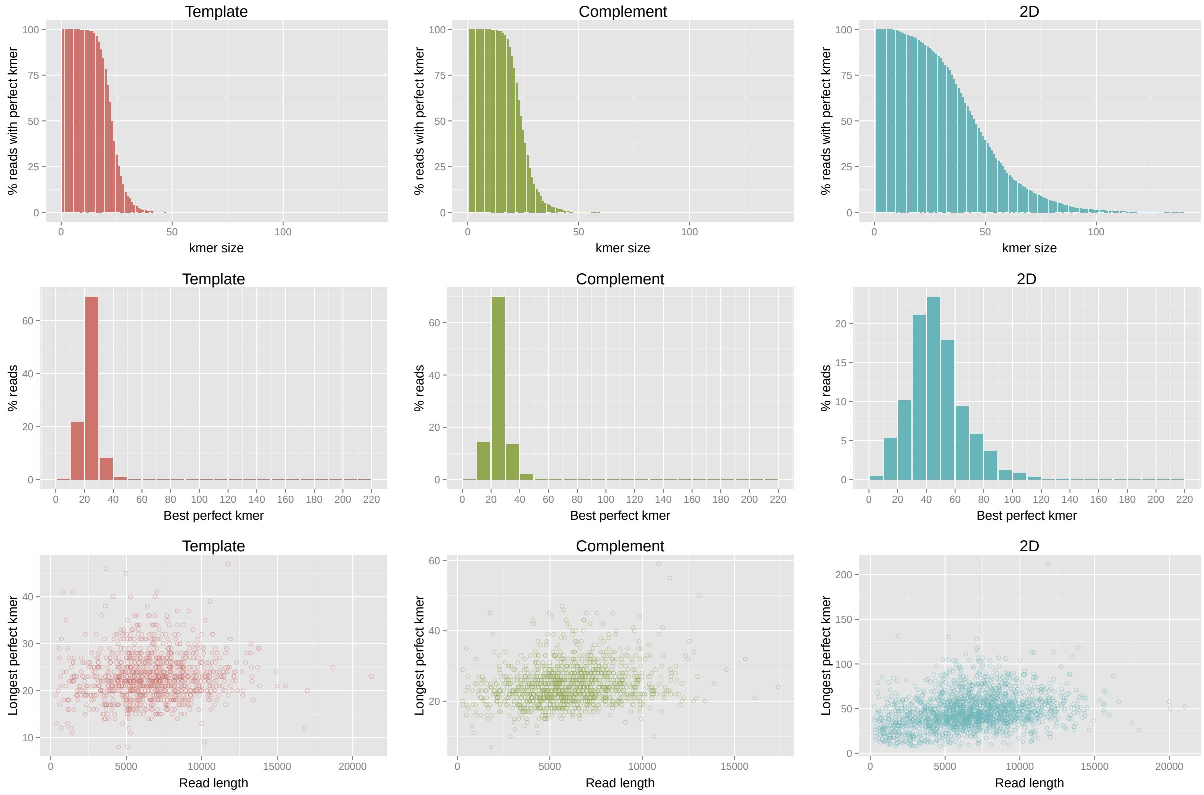

Pseudomonas aeruginosa coverage

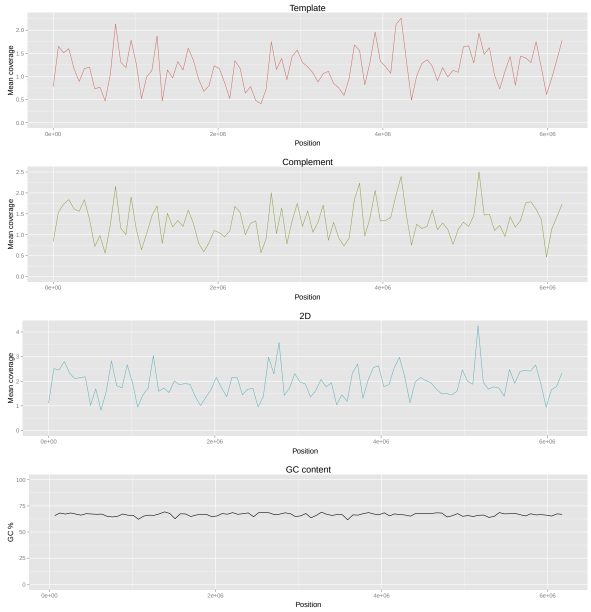

Pseudomonas aeruginosa 5-mer analysis

Under-represented 5-mers

	Template				Complement				2D			
Rank	kmer	Ref %	Read %	Diff %	kmer	Ref %	Read %	Diff %	kmer	Ref %	Read %	Diff %
1	CGCCG	0.796	0.145	-0.651	CGCCG	0.796	0.150	-0.646	CGCCG	0.796	0.371	-0.425
2	GCCGC	0.586	0.127	-0.459	GCGCC	0.615	0.145	-0.470	CGGCG	0.780	0.401	-0.379
3	CGGCG	0.780	0.330	-0.451	GCCGC	0.586	0.143	-0.443	GCCGC	0.586	0.271	-0.315
4	GCCGG	0.559	0.153	-0.406	CGGCG	0.780	0.380	-0.400	GCGCC	0.615	0.306	-0.309
5	GCGCC	0.615	0.217	-0.399	GGCGC	0.589	0.209	-0.381	CCAGC	0.457	0.179	-0.277
6	CGCGC	0.509	0.114	-0.395	CCGCC	0.497	0.124	-0.372	GGCGC	0.589	0.328	-0.262
7	GGCGC	0.589	0.228	-0.361	GCCGG	0.559	0.190	-0.369	CCGCC	0.497	0.248	-0.249
8	GCGCG	0.495	0.143	-0.352	TCGCC	0.463	0.114	-0.349	CGCGC	0.509	0.260	-0.248
9	GCCGA	0.475	0.124	-0.351	CGCGC	0.509	0.164	-0.345	TCGGC	0.484	0.236	-0.248
10	GGCCG	0.458	0.123	-0.335	GCCGA	0.475	0.154	-0.321	CCGGC	0.572	0.328	-0.244

Over-represented 5-mers

	Template				Complement				2D			
Rank	kmer	Ref %	Read %	Diff %	kmer	Ref %	Read %	Diff %	kmer	Ref %	Read %	Diff %
1	TATCT	0.016	0.153	0.137	TAAGC	0.010	0.130	0.121	GCTAG	0.011	0.122	0.111
2	TAGGC	0.052	0.183	0.131	CTAGC	0.011	0.126	0.115	CTAGC	0.011	0.111	0.099
3	GGCTA	0.055	0.178	0.123	CGGCT	0.229	0.337	0.108	CCATA	0.022	0.118	0.096
4	CATAC	0.019	0.139	0.121	CACAC	0.027	0.133	0.107	CATAG	0.032	0.121	0.089
5	TAATA	0.005	0.123	0.118	TGATA	0.013	0.119	0.105	CTAGG	0.009	0.097	0.089
6	CCTAA	0.004	0.121	0.118	CGTAA	0.012	0.116	0.104	GGCTA	0.055	0.143	0.088
7	TAAGC	0.010	0.126	0.116	GCTAA	0.007	0.108	0.101	TAGCC	0.059	0.145	0.086
8	TACAC	0.023	0.138	0.115	CTAAT	0.004	0.104	0.100	TAGGG	0.023	0.107	0.084
9	GCTTA	0.010	0.125	0.115	GCTAG	0.011	0.111	0.100	TAGAG	0.029	0.112	0.083
10	CTCTA	0.029	0.144	0.114	ATGAT	0.056	0.155	0.099	TAGCA	0.014	0.097	0.083

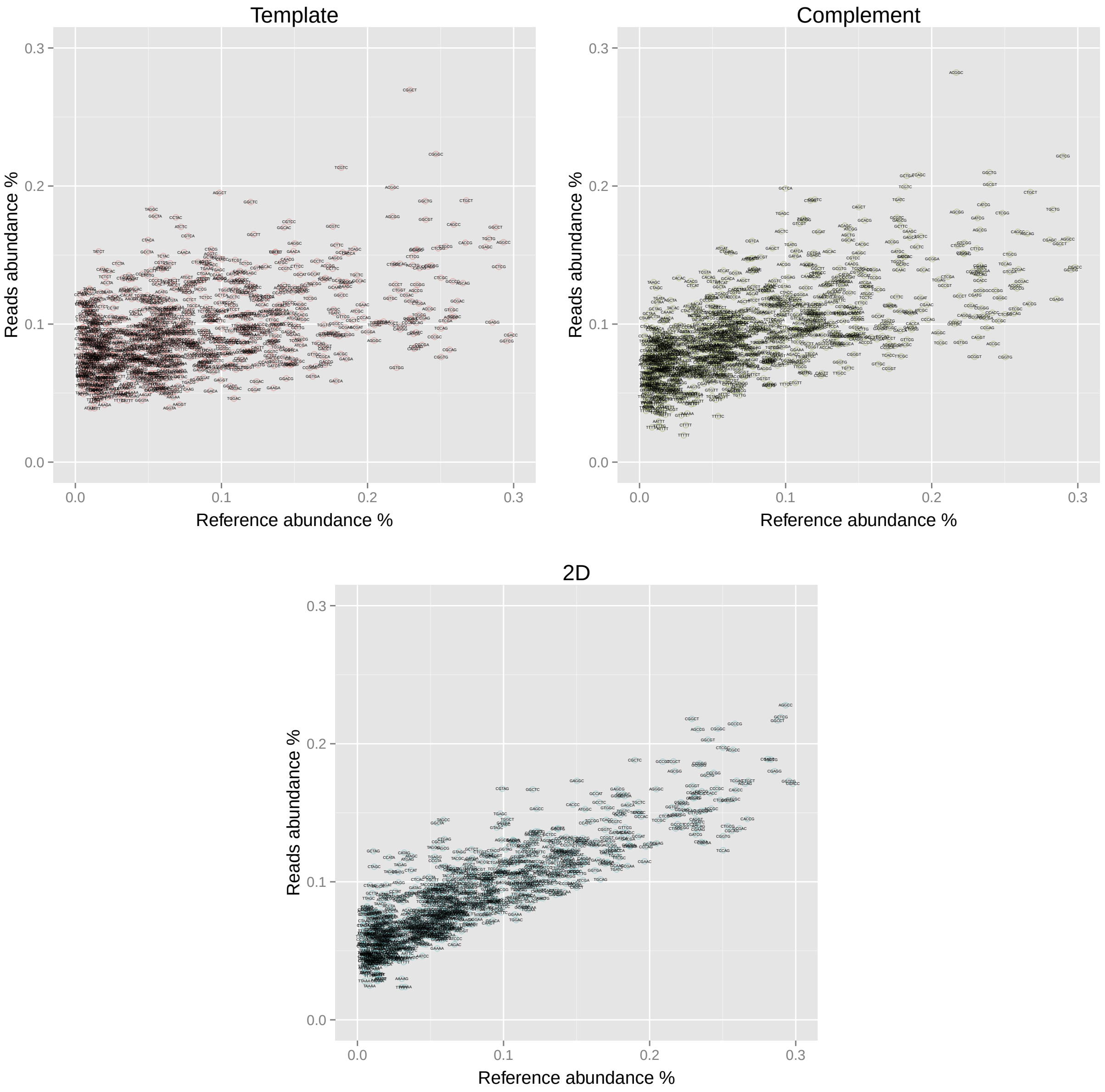

Pseudomonas aeruginosa GC content

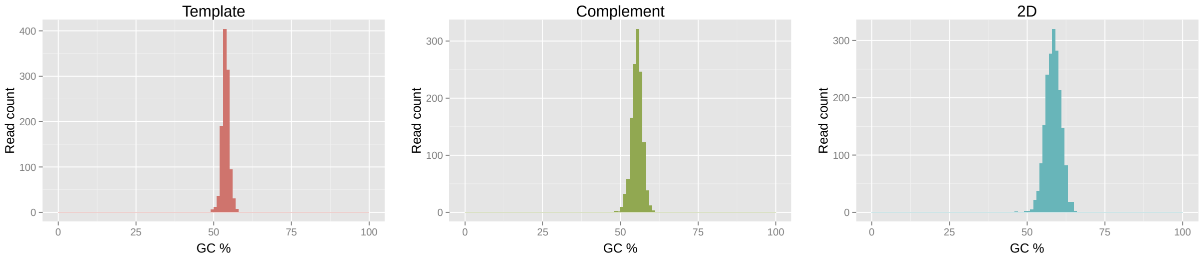

Rhodobacter sphaeroides 1 error analysis

	Template	Complement	2D
Overall base identity (excluding indels)	62.28%	66.12%	67.69%
Aligned base identity (excluding indels)	73.40%	77.25%	81.33%
Identical bases per 100 aligned bases (including indels)	61.31%	63.04%	70.08%
Inserted bases per 100 aligned bases (including indels)	4.55%	2.83%	8.40%
Deleted bases per 100 aligned bases (including indels)	11.93%	15.56%	5.44%
Substitutions per 100 aligned bases (including indels)	22.22%	18.56%	16.09%
Mean insertion size	1.47	1.37	1.66
Mean deletion size	1.63	1.81	1.43

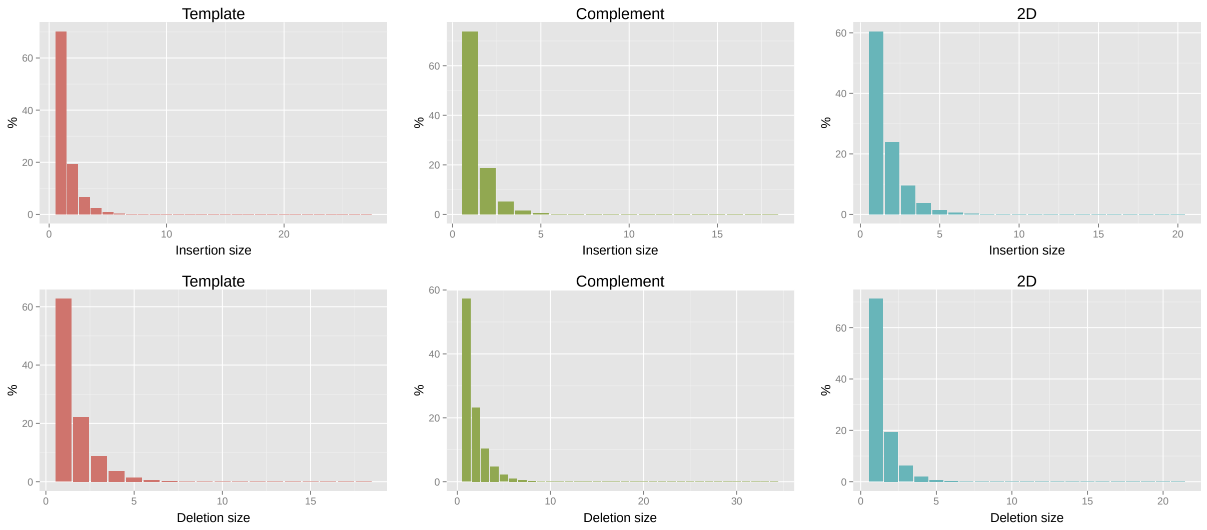

Rhodobacter sphaeroides 1 read identity

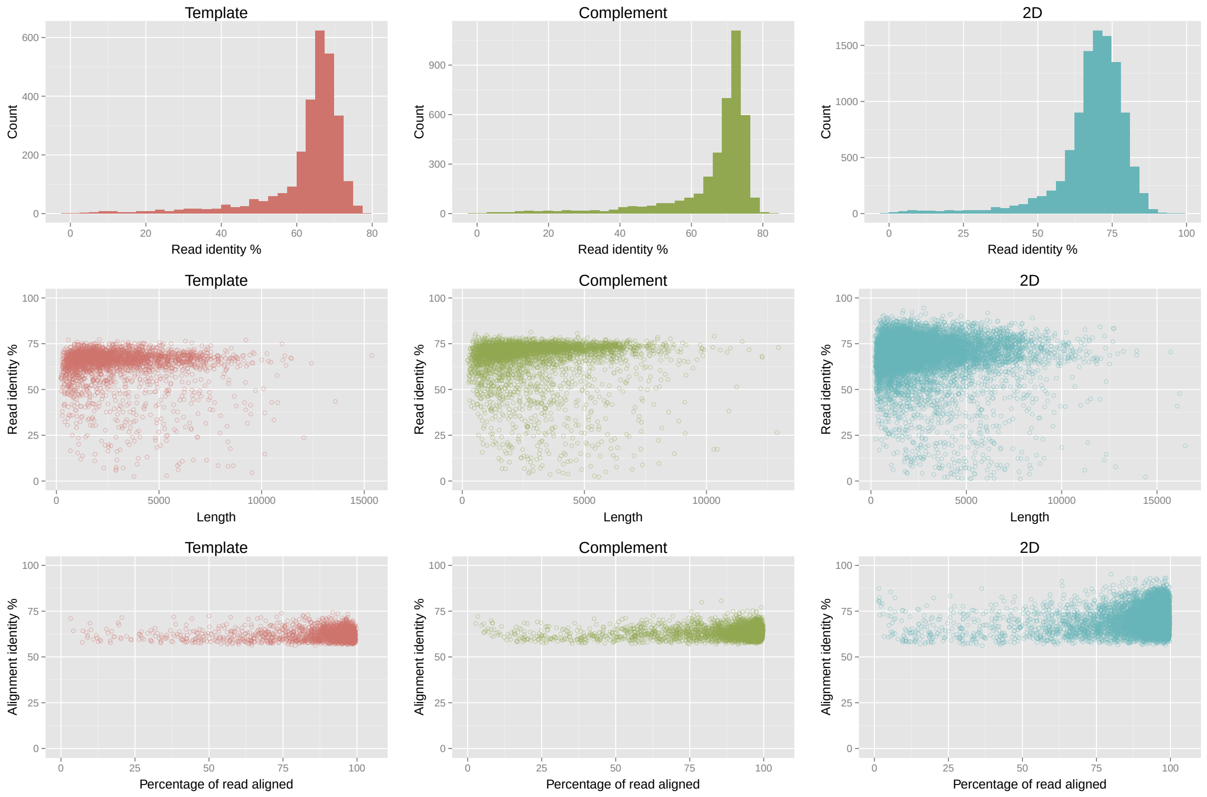

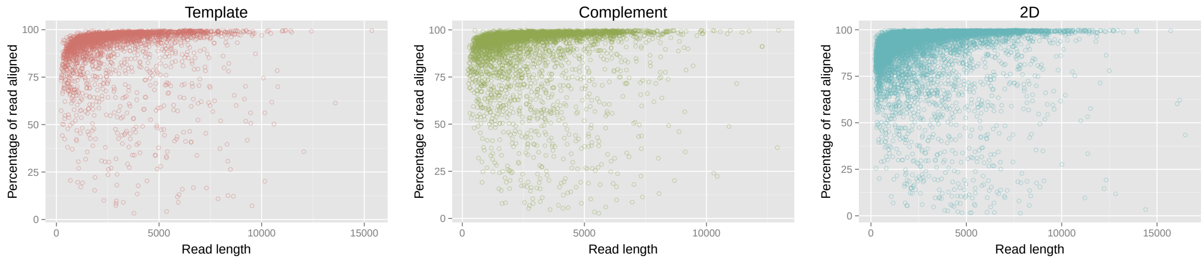

Rhodobacter sphaeroides 1 perfect kmers

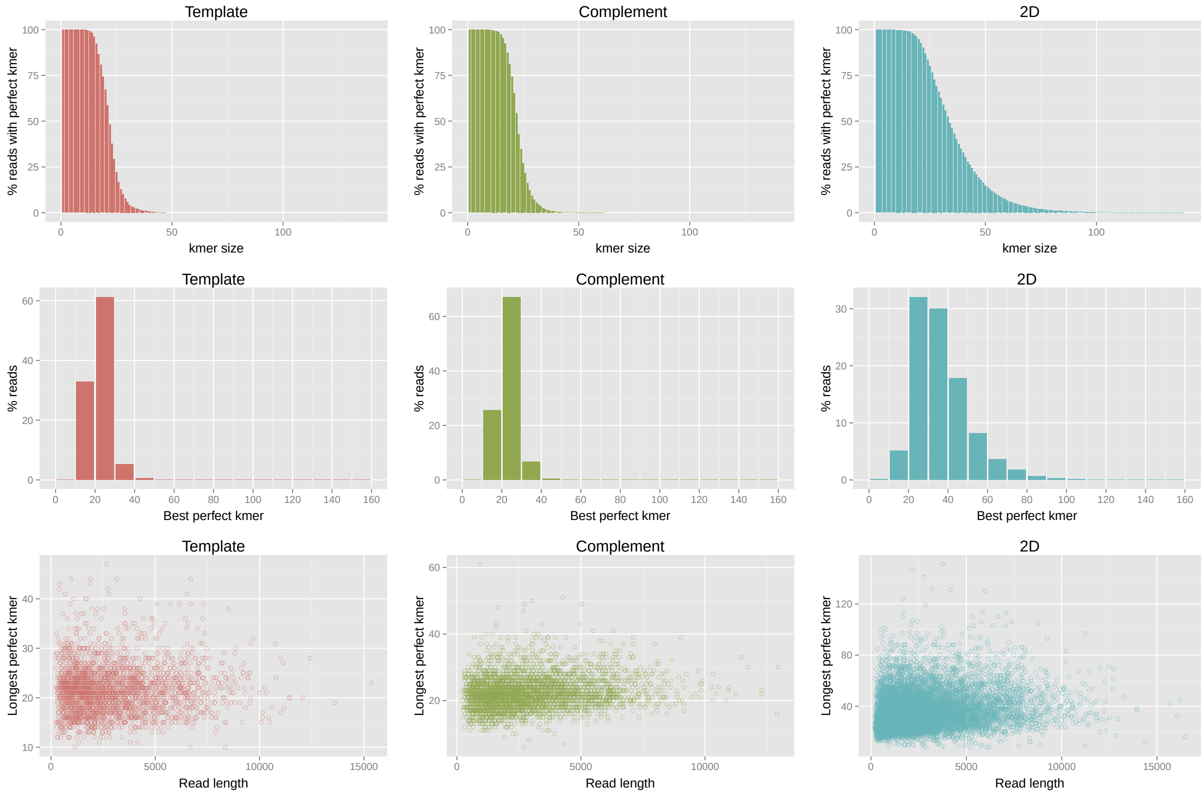

Rhodobacter sphaeroides 1 coverage

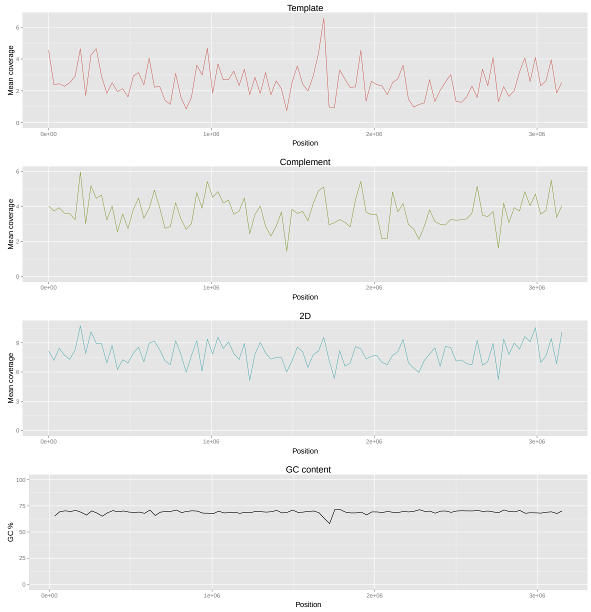

Rhodobacter sphaeroides 1 5-mer analysis

Under-represented 5-mers

	Template				Complement				2D			
Rank	kmer	Ref %	Read %	Diff %	kmer	Ref %	Read %	Diff %	kmer	Ref %	Read %	Diff %
1	GCCGC	0.760	0.136	-0.623	GCCGC	0.760	0.166	-0.593	GCCGC	0.760	0.280	-0.480
2	CGCGC	0.726	0.124	-0.602	CGCCG	0.699	0.150	-0.549	CGCGC	0.726	0.279	-0.447
3	CGCCG	0.699	0.135	-0.565	CGCGC	0.726	0.206	-0.520	GCGCG	0.716	0.291	-0.425
4	GCGCG	0.716	0.156	-0.560	GCGCC	0.641	0.145	-0.496	CGGCC	0.641	0.250	-0.391
5	GGCCG	0.638	0.128	-0.511	GCGCG	0.716	0.234	-0.482	CCGCG	0.599	0.213	-0.386
6	CCGCG	0.599	0.101	-0.498	CCGCC	0.609	0.130	-0.479	CGCCG	0.699	0.319	-0.381
7	CGGCC	0.641	0.189	-0.452	CCGCG	0.599	0.133	-0.466	CCGCC	0.609	0.241	-0.369
8	CGCGG	0.592	0.144	-0.449	GGCCG	0.638	0.191	-0.448	GCGGC	0.730	0.366	-0.365
9	GCGCC	0.641	0.207	-0.434	CGGGC	0.553	0.122	-0.432	GCGCC	0.641	0.277	-0.364
10	CCGCC	0.609	0.176	-0.433	GGCGC	0.623	0.207	-0.416	GGCCG	0.638	0.280	-0.359

Over-represented 5-mers

	Template				Complement				2D			
Rank	kmer	Ref %	Read %	Diff %	kmer	Ref %	Read %	Diff %	kmer	Ref %	Read %	Diff %
1	CCTAC	0.037	0.187	0.150	CACAC	0.020	0.156	0.136	GCTAG	0.007	0.153	0.146
2	TACAC	0.010	0.156	0.146	CTAGC	0.006	0.133	0.127	CTAGC	0.006	0.144	0.138
3	GGCTA	0.035	0.171	0.136	CTAAT	0.002	0.117	0.115	CGTAG	0.055	0.186	0.131
4	TATCT	0.025	0.156	0.131	GCTAG	0.007	0.118	0.111	TAGCC	0.038	0.168	0.130
5	CTACA	0.030	0.160	0.130	TAAGC	0.008	0.119	0.111	GGCTA	0.035	0.162	0.127
6	CACAC	0.020	0.143	0.124	ACACG	0.046	0.157	0.110	TAGGG	0.020	0.138	0.118
7	ACACA	0.007	0.130	0.124	CGTAC	0.006	0.115	0.109	CTAGG	0.006	0.122	0.116
8	CTACT	0.014	0.138	0.124	CCTAC	0.037	0.143	0.106	TAGCG	0.027	0.142	0.115
9	CTAAT	0.002	0.123	0.121	CCTAG	0.005	0.111	0.106	CGCTA	0.025	0.134	0.109
10	TCTCT	0.032	0.153	0.120	TCGTA	0.035	0.140	0.105	TAGAG	0.032	0.136	0.104

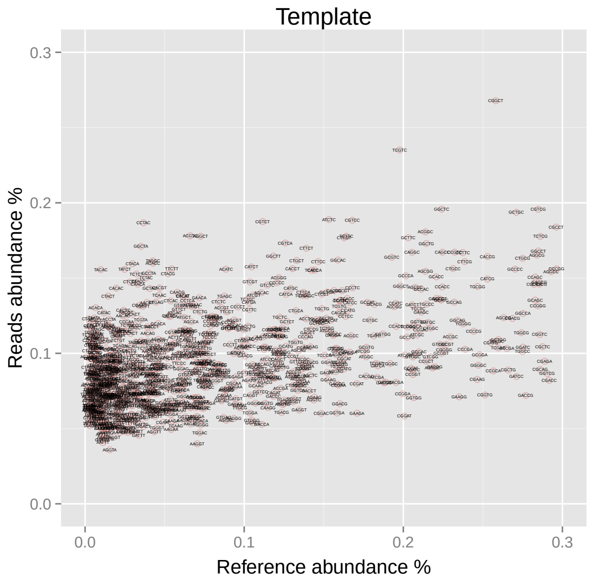

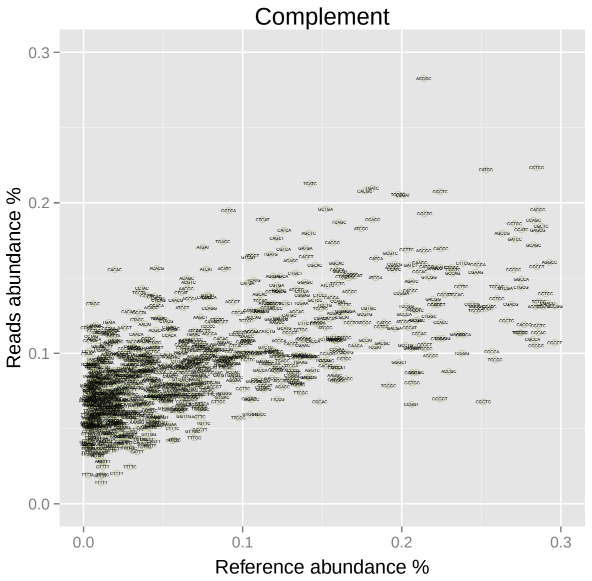

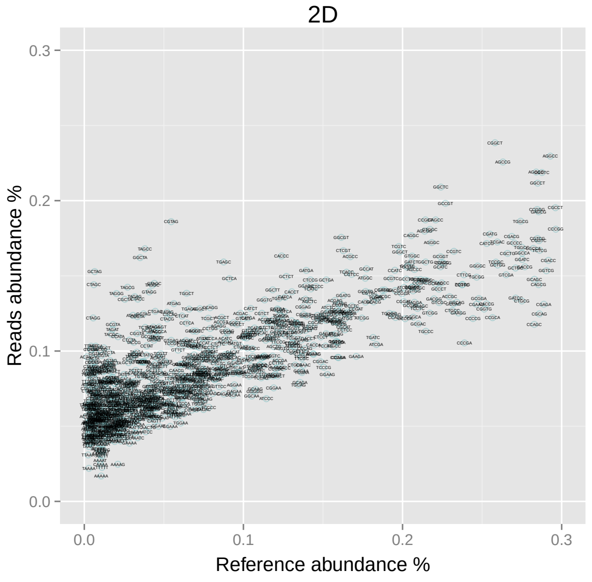

Rhodobacter sphaeroides 1 GC content

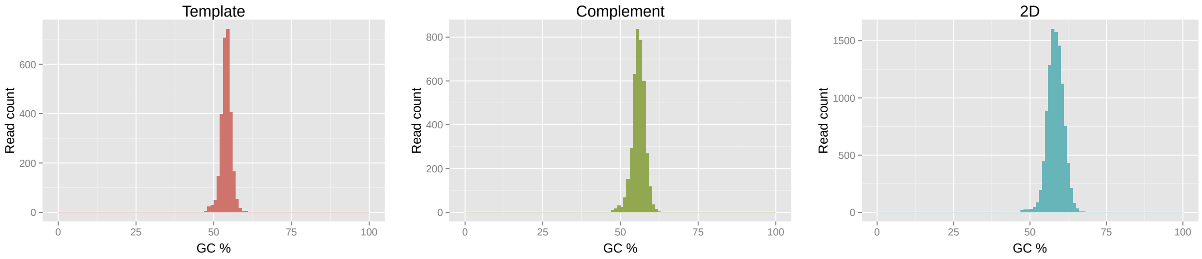

Rhodobacter sphaeroides 2 error analysis

	Template	Complement	2D
Overall base identity (excluding indels)	59.98%	64.50%	65.56%
Aligned base identity (excluding indels)	73.82%	77.23%	81.27%
Identical bases per 100 aligned bases (including indels)	61.71%	63.00%	69.97%
Inserted bases per 100 aligned bases (including indels)	4.59%	2.81%	8.51%
Deleted bases per 100 aligned bases (including indels)	11.82%	15.61%	5.39%
Substitutions per 100 aligned bases (including indels)	21.88%	18.57%	16.13%
Mean insertion size	1.47	1.37	1.67
Mean deletion size	1.62	1.81	1.43

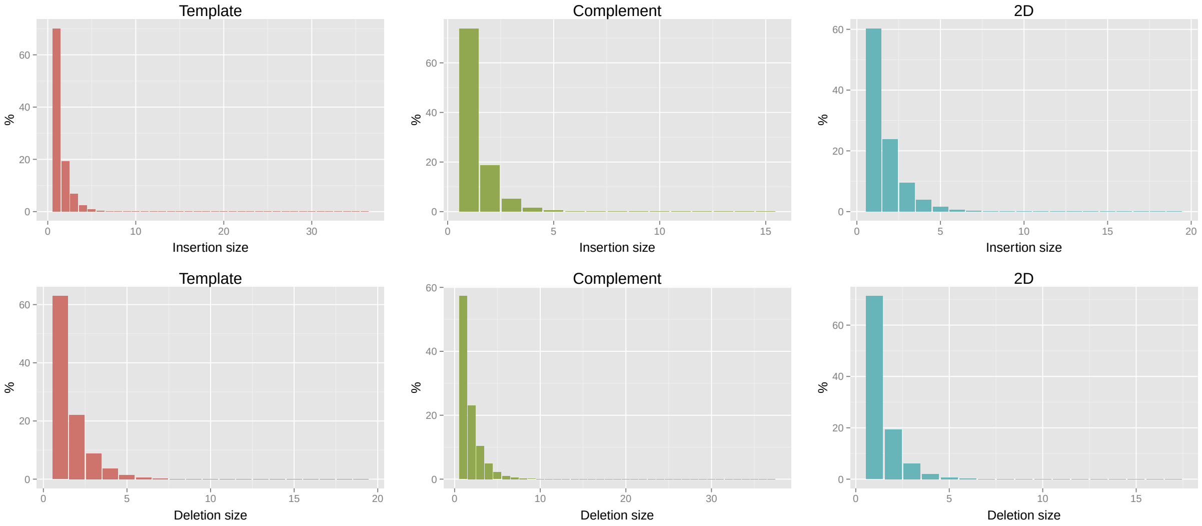

Rhodobacter sphaeroides 2 read identity

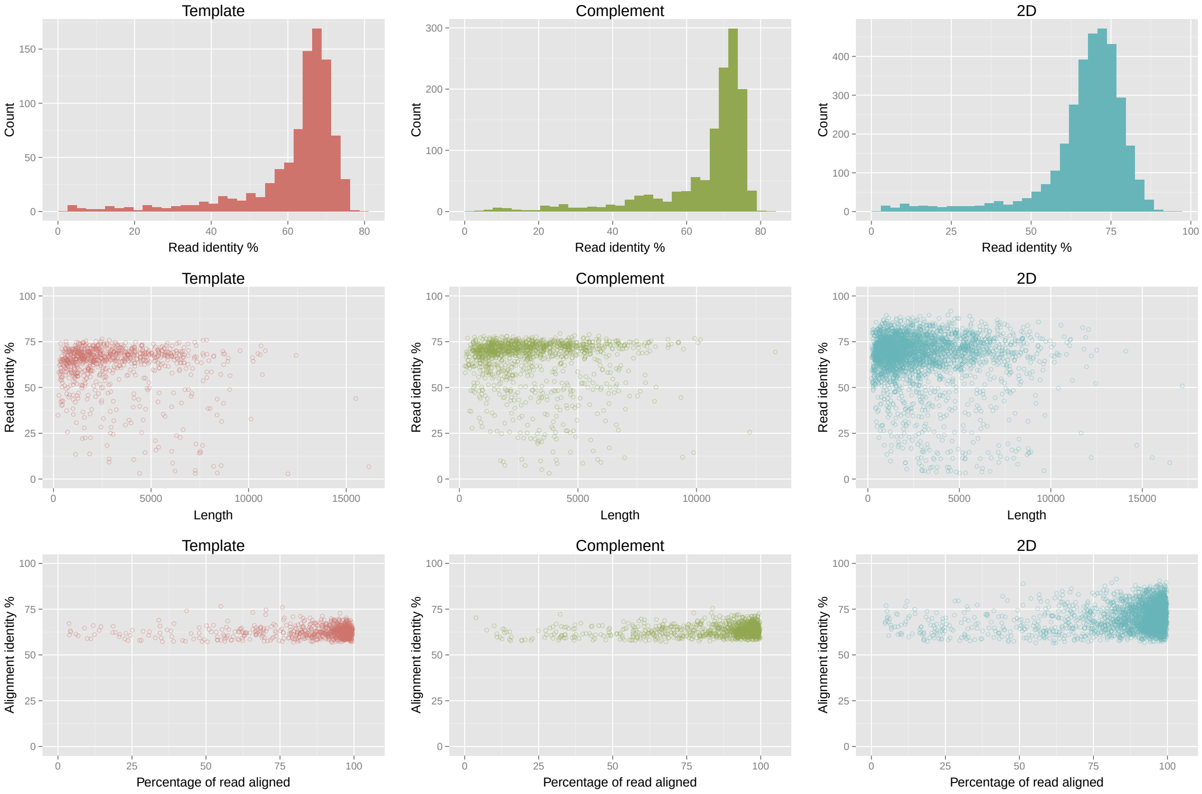

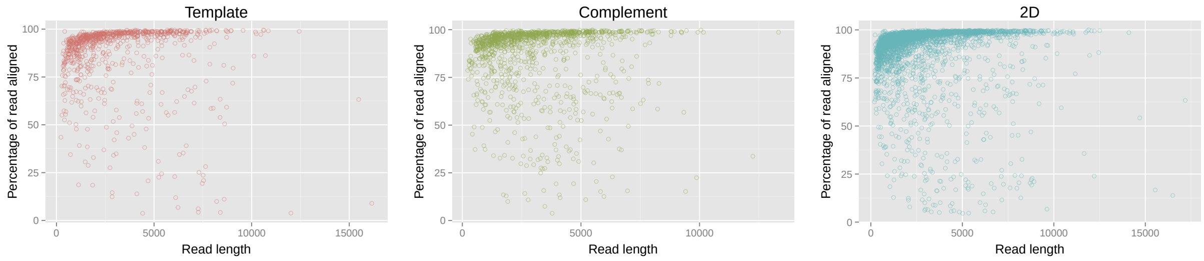

Rhodobacter sphaeroides 2 perfect kmers

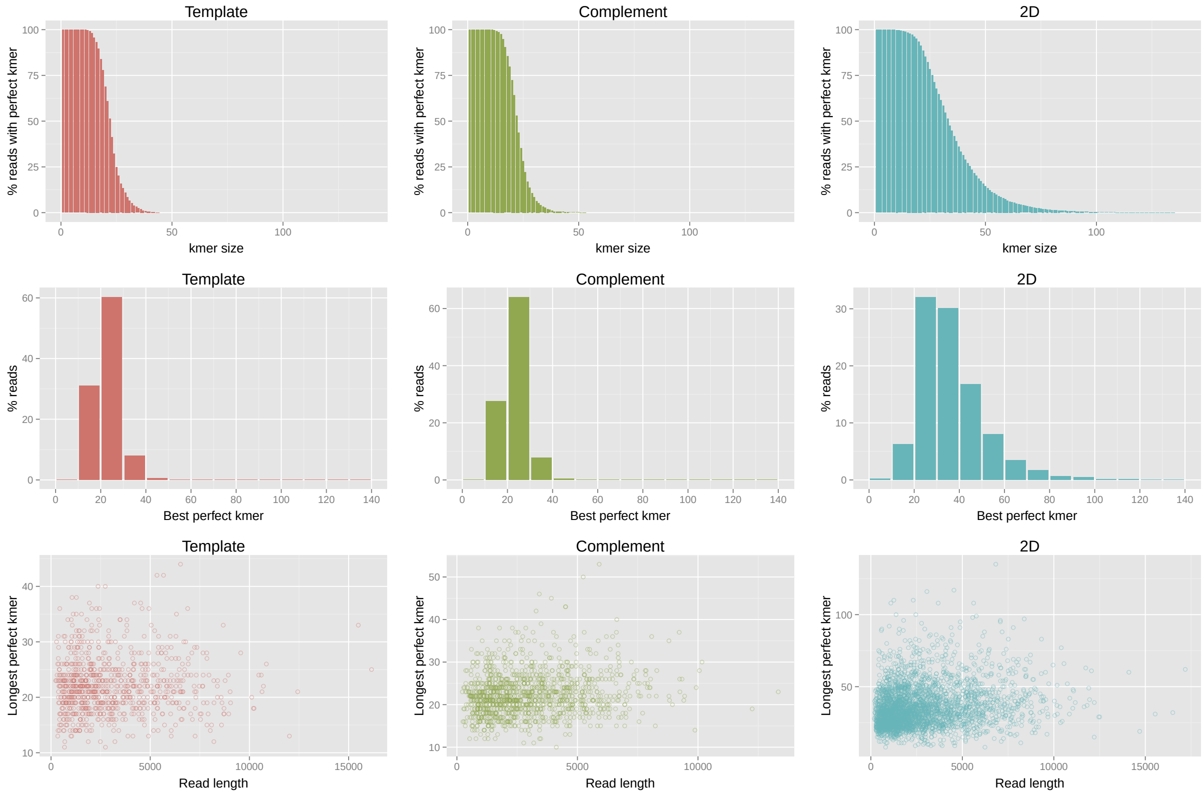

Rhodobacter sphaeroides 2 coverage

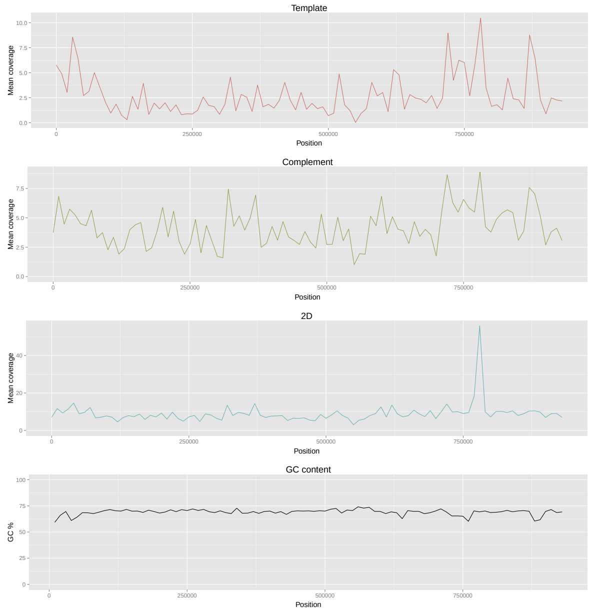

Rhodobacter sphaeroides 2 5-mer analysis

Under-represented 5-mers

	Template				Complement				2D			
Rank	kmer	Ref %	Read %	Diff %	kmer	Ref %	Read %	Diff %	kmer	Ref %	Read %	Diff %
1	GCCGC	0.762	0.137	-0.624	GCCGC	0.762	0.163	-0.599	GCCGC	0.762	0.279	-0.482
2	CGCGC	0.717	0.123	-0.594	CGCCG	0.703	0.146	-0.557	CGCGC	0.717	0.273	-0.444
3	CGCCG	0.703	0.133	-0.570	CGCGC	0.717	0.200	-0.517	GCGCG	0.717	0.282	-0.435
4	GCGCG	0.717	0.155	-0.562	GCGCC	0.630	0.139	-0.491	CGGCC	0.639	0.248	-0.391
5	GGCCG	0.655	0.130	-0.525	GCGCG	0.717	0.227	-0.489	CGCCG	0.703	0.316	-0.387
6	CCGCG	0.596	0.103	-0.492	CCGCC	0.599	0.130	-0.469	GCGGC	0.746	0.361	-0.385
7	CGGCC	0.639	0.184	-0.454	GGCCG	0.655	0.187	-0.468	CCGCG	0.596	0.212	-0.384
8	CGCGG	0.596	0.146	-0.449	CCGCG	0.596	0.128	-0.468	GGCCG	0.655	0.277	-0.378
9	GCGGC	0.746	0.308	-0.438	CGGGC	0.539	0.117	-0.422	CCGCC	0.599	0.240	-0.359
10	GCGCC	0.630	0.204	-0.426	GGCGC	0.621	0.202	-0.419	GCGCC	0.630	0.271	-0.359

Over-represented 5-mers

	Template				Complement				2D			
Rank	kmer	Ref %	Read %	Diff %	kmer	Ref %	Read %	Diff %	kmer	Ref %	Read %	Diff %
1	CCTAC	0.039	0.176	0.137	CTAGC	0.008	0.135	0.128	GCTAG	0.008	0.154	0.146
2	TACAC	0.012	0.146	0.135	CACAC	0.022	0.149	0.127	CTAGC	0.008	0.144	0.136
3	TATCT	0.026	0.158	0.132	GCTAG	0.008	0.124	0.116	TAGCC	0.037	0.166	0.129
4	GGCTA	0.034	0.165	0.131	TCGTA	0.034	0.147	0.113	CGTAG	0.058	0.184	0.126
5	TCTCT	0.031	0.151	0.120	CTAAT	0.003	0.115	0.112	GGCTA	0.034	0.158	0.124
6	TAGGC	0.042	0.160	0.119	TAAGC	0.010	0.122	0.111	CTAGG	0.006	0.122	0.116
7	CACAC	0.022	0.141	0.119	CGTAA	0.015	0.122	0.107	TAGGG	0.020	0.135	0.116
8	TAATA	0.006	0.124	0.118	CGTAC	0.010	0.117	0.107	TAGCG	0.030	0.140	0.110
9	CTCTA	0.027	0.145	0.118	TGATA	0.018	0.124	0.106	CGCTA	0.031	0.138	0.107
10	CTACT	0.014	0.131	0.117	GCTCA	0.092	0.198	0.105	TAGAG	0.031	0.133	0.102

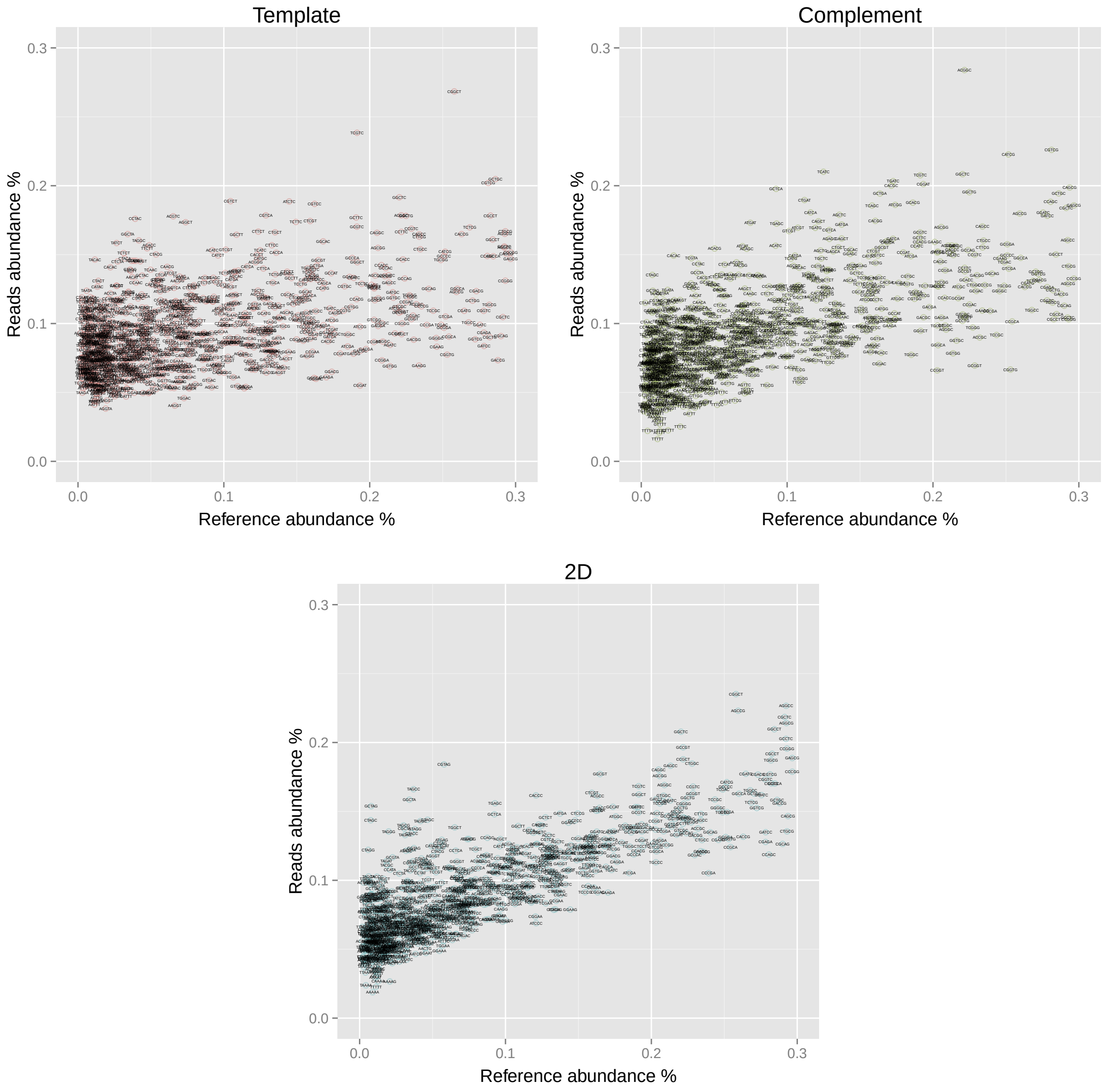

Rhodobacter sphaeroides 2 GC content

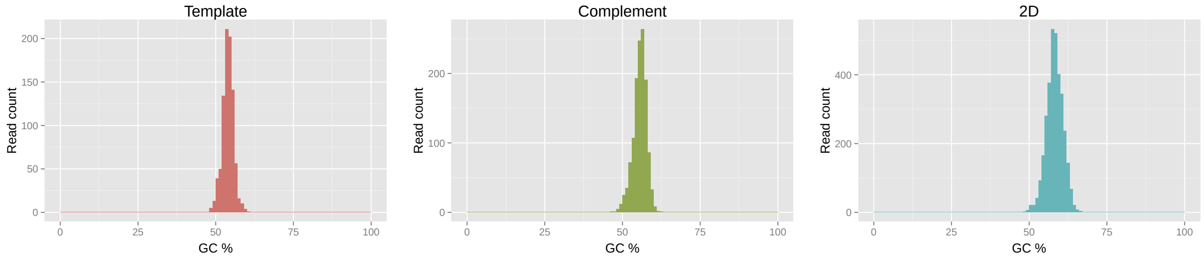

Staphylococcus aureus error analysis

	Template	Complement	2D
Overall base identity (excluding indels)	65.80%	66.77%	70.00%
Aligned base identity (excluding indels)	76.22%	79.16%	82.58%
Identical bases per 100 aligned bases (including indels)	63.59%	64.60%	72.03%
Inserted bases per 100 aligned bases (including indels)	3.42%	2.30%	6.70%
Deleted bases per 100 aligned bases (including indels)	13.16%	16.10%	6.07%
Substitutions per 100 aligned bases (including indels)	19.83%	17.01%	15.20%
Mean insertion size	1.45	1.35	1.54
Mean deletion size	1.72	1.83	1.44

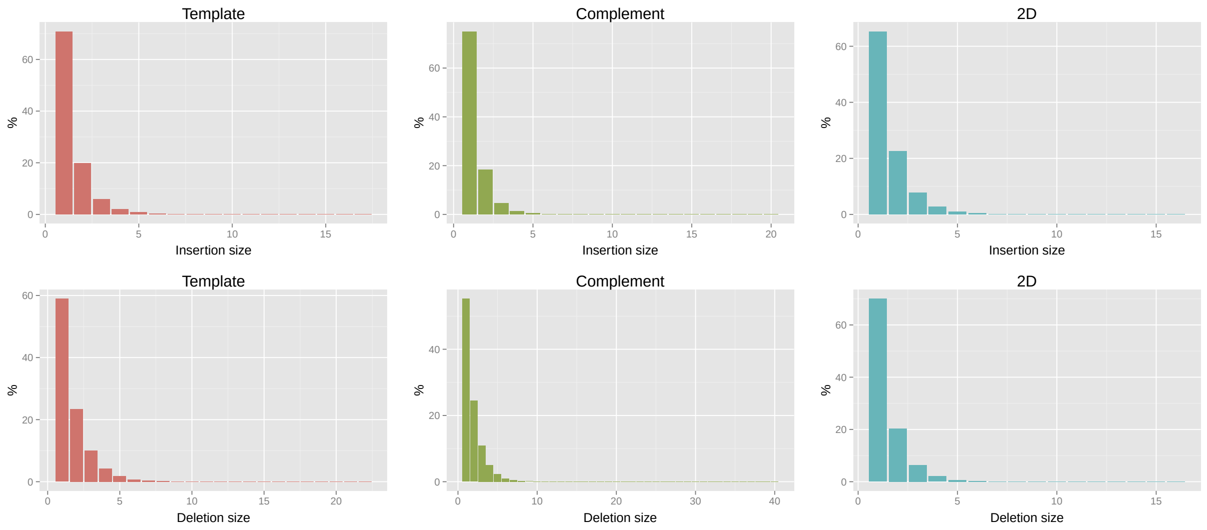

Staphylococcus aureus read identity

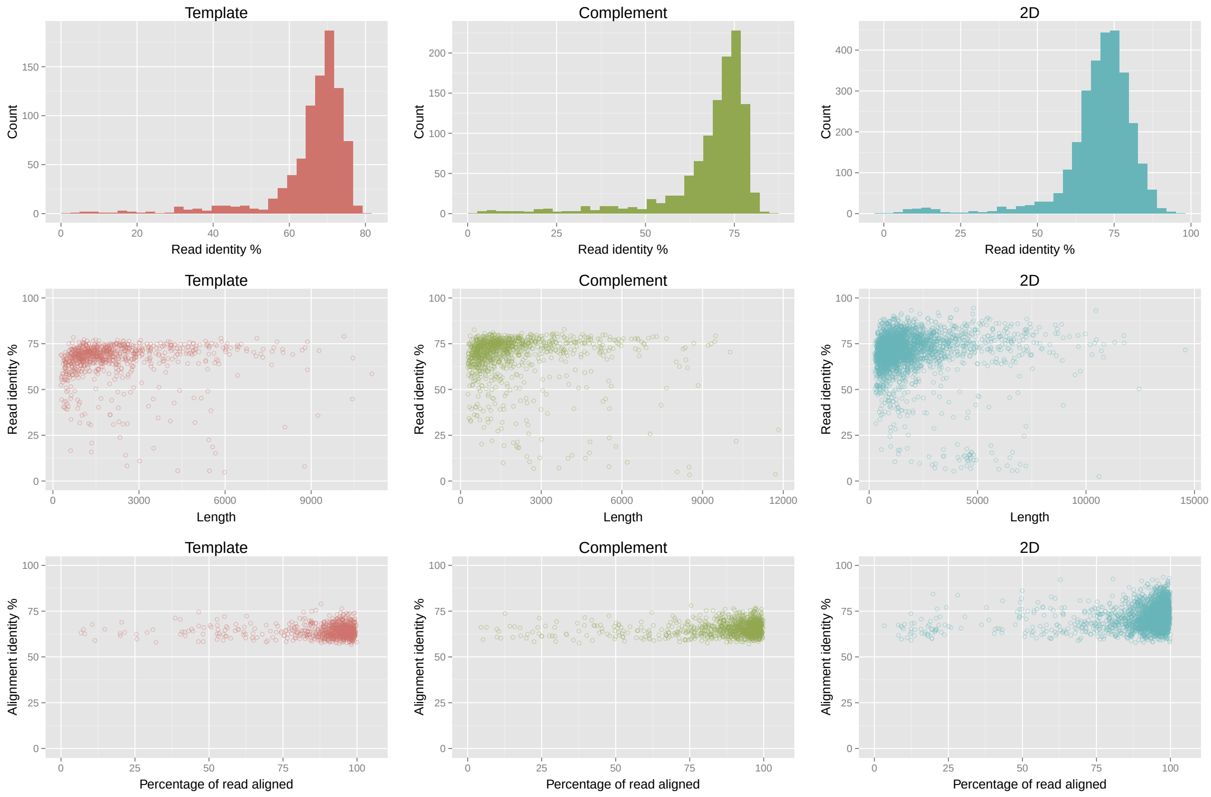

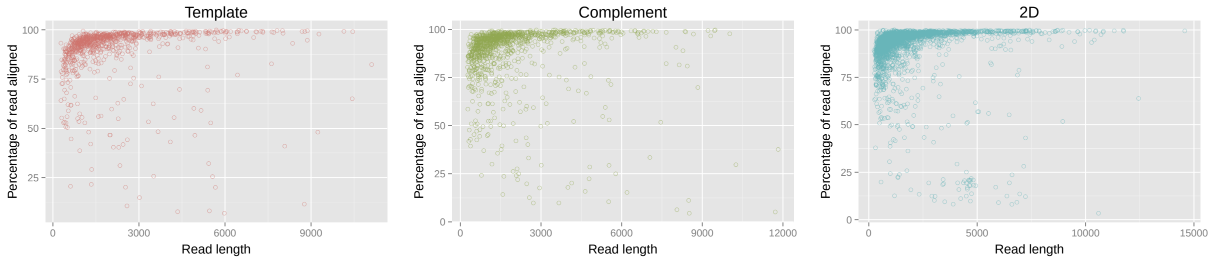

Staphylococcus aureus perfect kmers

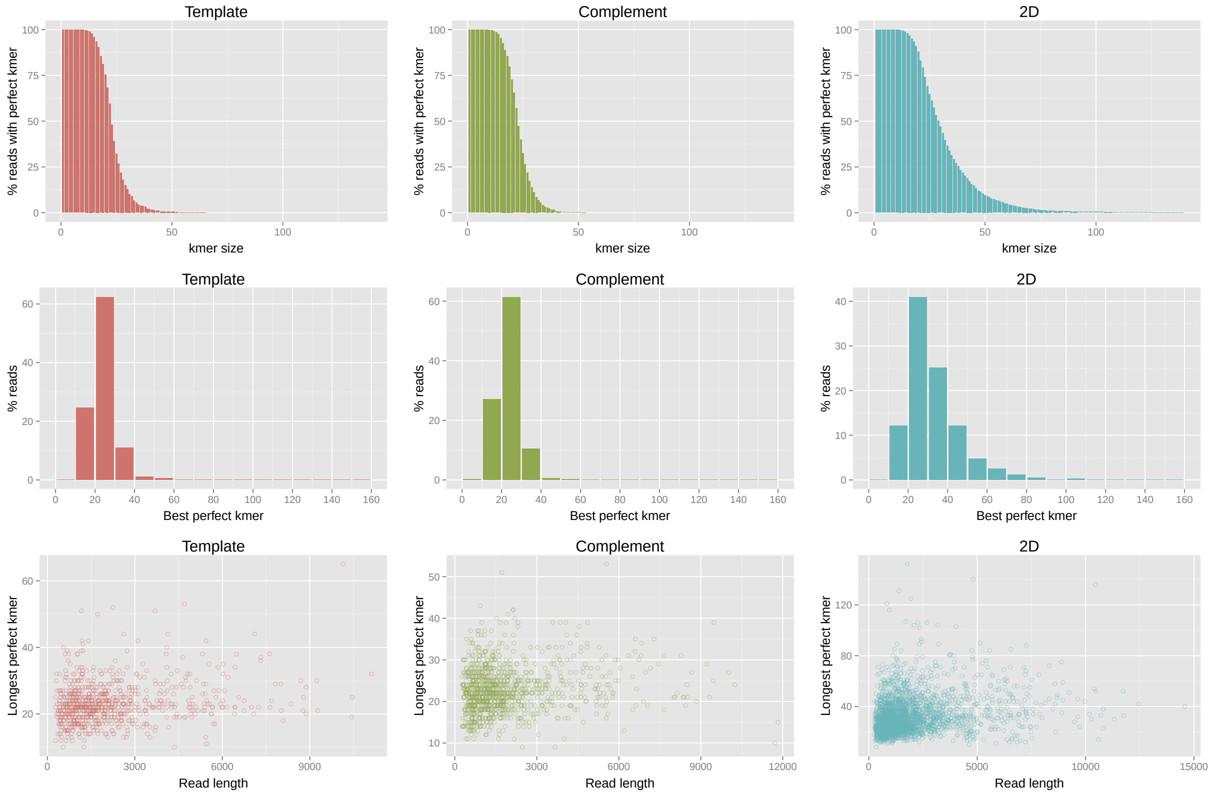

Staphylococcus aureus coverage

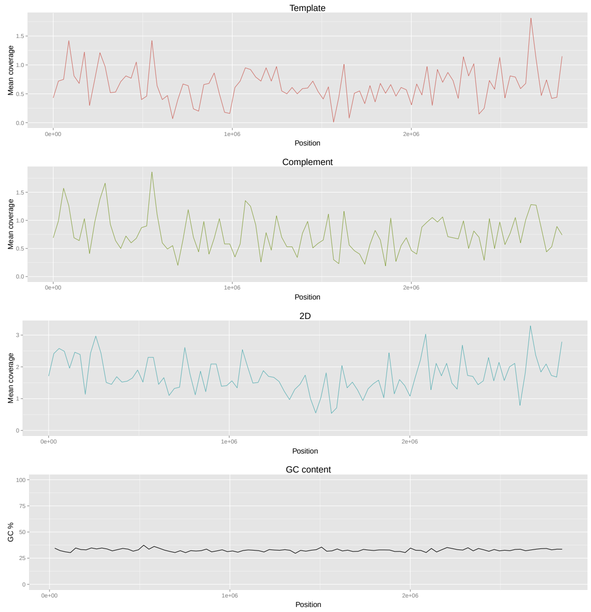

Staphylococcus aureus 5-mer analysis

Under-represented 5-mers

	Template				Complement				2D			
Rank	kmer	Ref %	Read %	Diff %	kmer	Ref %	Read %	Diff %	kmer	Ref %	Read %	Diff %
1	TTTTT	0.502	0.099	-0.402	TTTTT	0.502	0.080	-0.422	TTTTT	0.502	0.089	-0.412
2	AAAAA	0.504	0.105	-0.399	AAAAA	0.504	0.100	-0.404	AAAAA	0.504	0.118	-0.386
3	TAAAA	0.492	0.100	-0.393	ATTTT	0.529	0.150	-0.379	AAAAT	0.533	0.210	-0.323
4	AAAAT	0.533	0.157	-0.376	AAAAT	0.533	0.166	-0.367	ATTTT	0.529	0.209	-0.320
5	TTAAA	0.517	0.158	-0.359	TAAAA	0.492	0.147	-0.346	TTTTA	0.490	0.175	-0.315
6	TTTAA	0.521	0.168	-0.353	TTAAA	0.517	0.175	-0.342	TAAAA	0.492	0.181	-0.311
7	ATAAA	0.440	0.102	-0.338	TTTTA	0.490	0.165	-0.325	TTTAA	0.521	0.216	-0.305
8	ATTTT	0.529	0.196	-0.333	ATAAA	0.440	0.120	-0.320	TTAAA	0.517	0.242	-0.275
9	TTTTA	0.490	0.178	-0.312	AAATT	0.443	0.144	-0.300	TTTAT	0.448	0.189	-0.258
10	AAATT	0.443	0.133	-0.310	TTTAA	0.521	0.229	-0.293	AATTT	0.439	0.184	-0.255

Over-represented 5-mers

	Template				Complement				2D			
Rank	kmer	Ref %	Read %	Diff %	kmer	Ref %	Read %	Diff %	kmer	Ref %	Read %	Diff %
1	CGGGC	0.006	0.183	0.178	CGGCT	0.021	0.169	0.149	CGGGC	0.006	0.083	0.077
2	CGGCT	0.021	0.142	0.121	TCGGC	0.020	0.165	0.145	CATGC	0.059	0.133	0.073
3	AGGCT	0.033	0.151	0.118	GATCG	0.026	0.140	0.113	CGCAG	0.022	0.090	0.068
4	GGCTG	0.024	0.138	0.114	GCGGC	0.014	0.125	0.111	GCATG	0.055	0.122	0.066
5	GGCTC	0.019	0.132	0.113	GCTGC	0.057	0.162	0.105	GGATC	0.034	0.097	0.064
6	ATGCG	0.060	0.169	0.109	CGGCG	0.011	0.115	0.103	CGACC	0.021	0.084	0.063
7	GCTGC	0.057	0.165	0.108	TCGTG	0.045	0.148	0.103	GGGCA	0.022	0.085	0.063
8	TAGGC	0.032	0.135	0.103	GCGTG	0.025	0.127	0.102	CAGGC	0.026	0.088	0.062
9	TCGGC	0.020	0.121	0.101	CGTAG	0.027	0.128	0.102	TGCCG	0.030	0.092	0.062
10	GGCTT	0.054	0.153	0.099	GCGTA	0.044	0.145	0.101	GGCAG	0.025	0.087	0.062

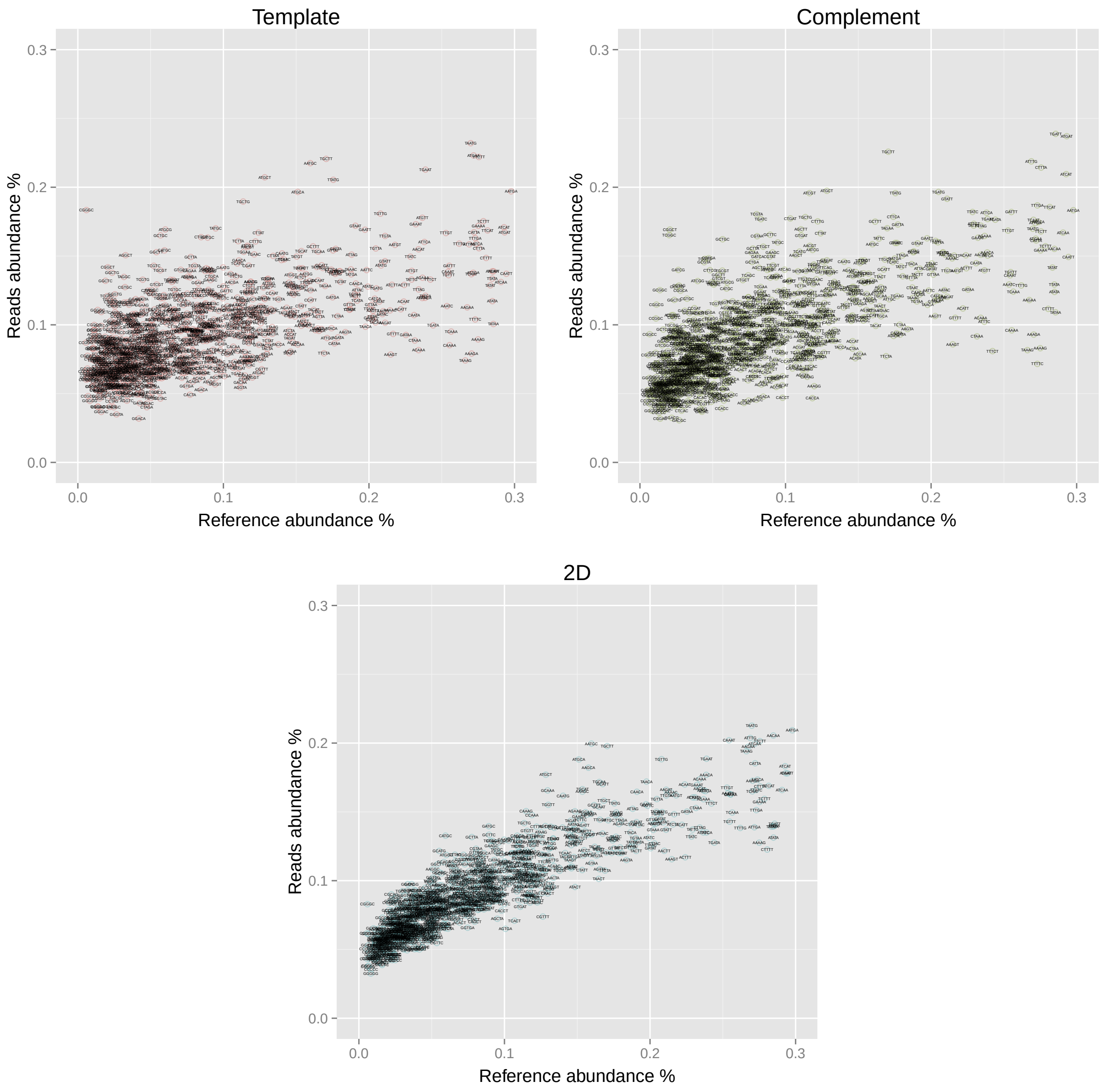

Staphylococcus aureus GC content

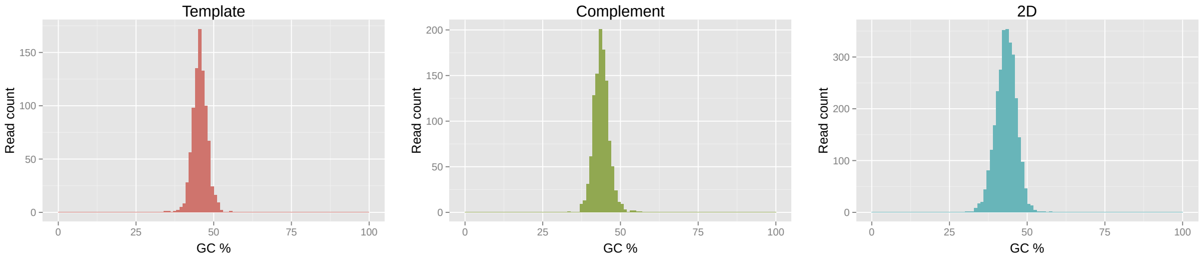

Staphylococcus epidermidis error analysis

	Template	Complement	2D
Overall base identity (excluding indels)	65.00%	68.44%	73.43%
Aligned base identity (excluding indels)	75.47%	78.24%	83.57%
Identical bases per 100 aligned bases (including indels)	62.73%	63.65%	73.13%
Inserted bases per 100 aligned bases (including indels)	3.53%	2.32%	6.46%
Deleted bases per 100 aligned bases (including indels)	13.36%	16.33%	6.03%
Substitutions per 100 aligned bases (including indels)	20.39%	17.70%	14.38%
Mean insertion size	1.45	1.35	1.54
Mean deletion size	1.73	1.85	1.45

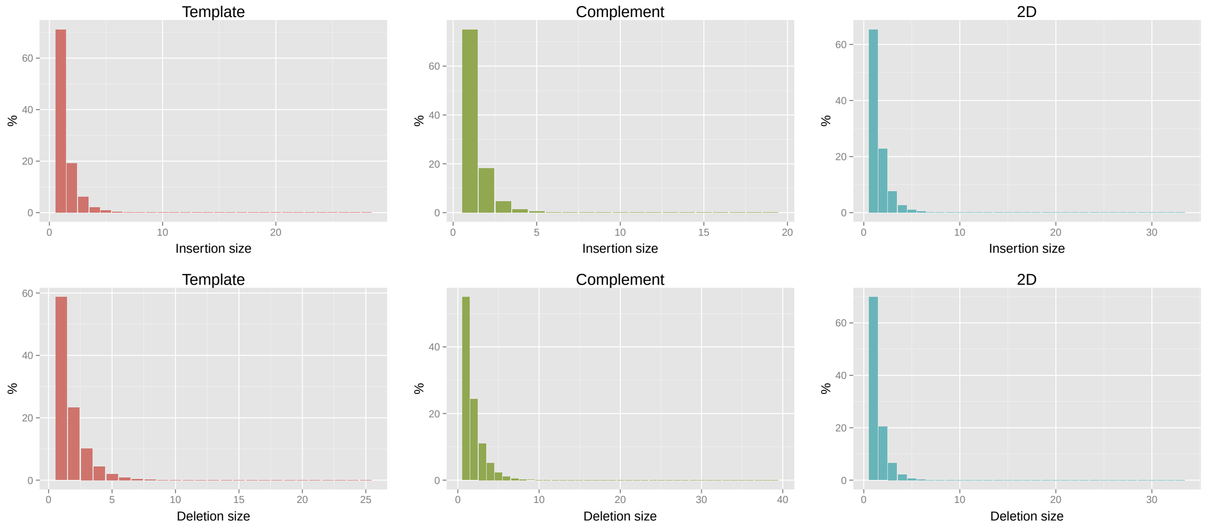

Staphylococcus epidermidis read identity

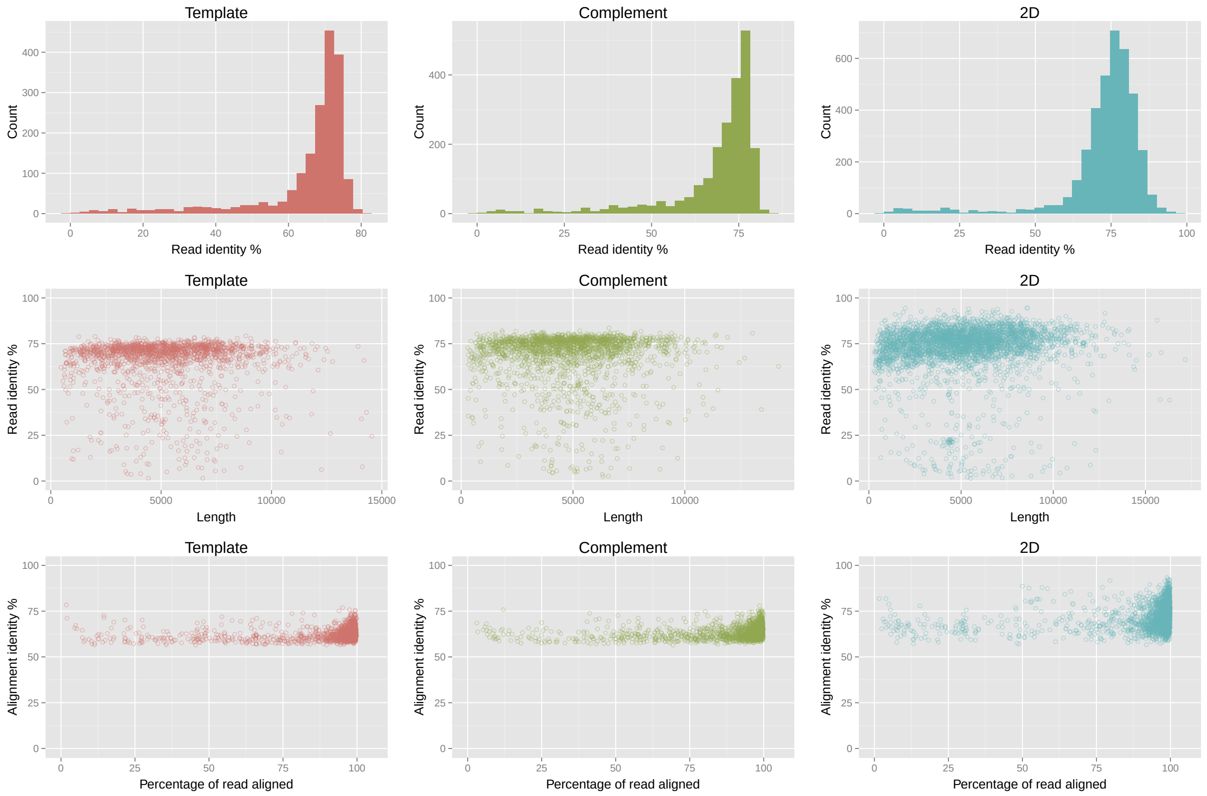

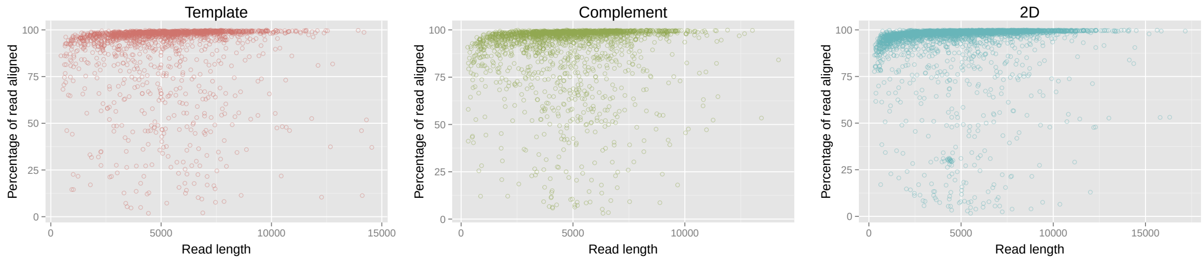

Staphylococcus epidermidis perfect kmers

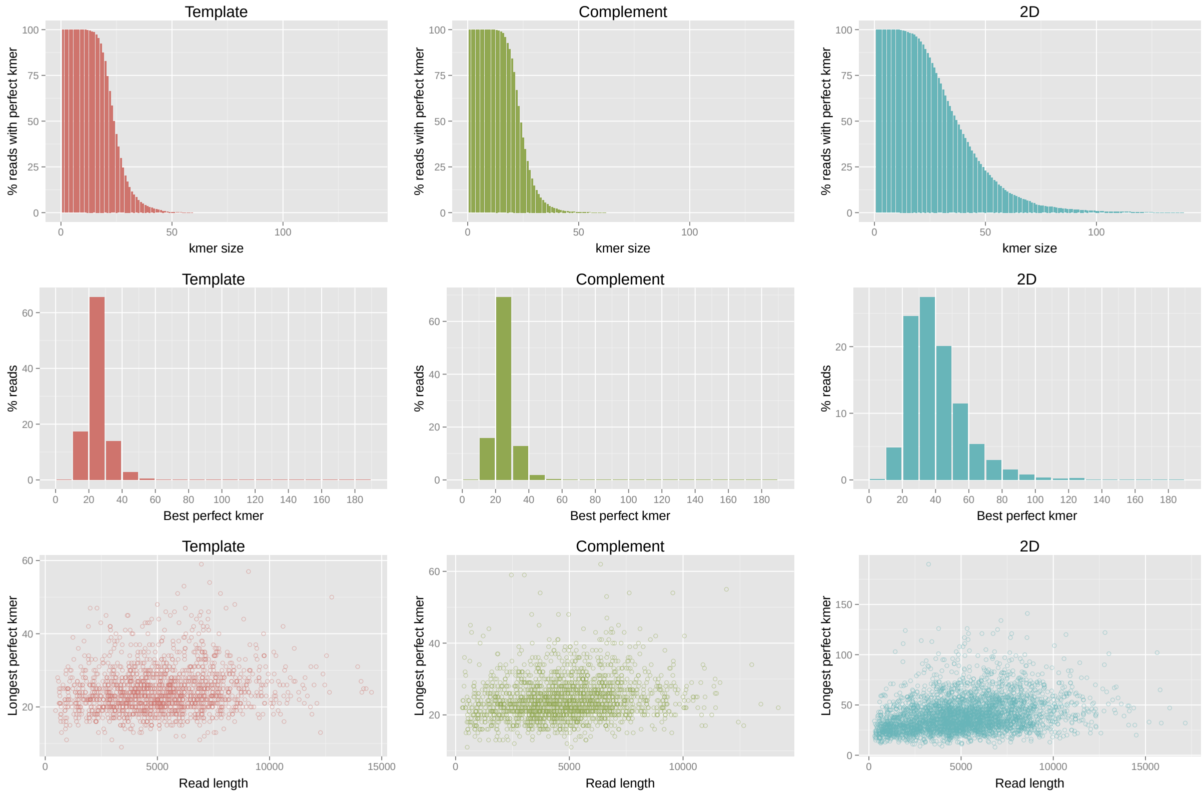

Staphylococcus epidermidis coverage

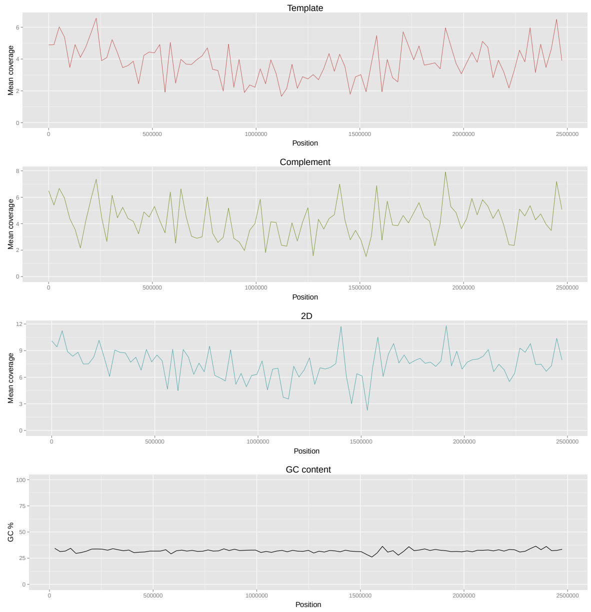

Staphylococcus epidermidis 5-mer analysis

Under-represented 5-mers

	Template				Complement				2D			
Rank	kmer	Ref %	Read %	Diff %	kmer	Ref %	Read %	Diff %	kmer	Ref %	Read %	Diff %
1	TTTTT	0.575	0.112	-0.463	TTTTT	0.575	0.086	-0.489	TTTTT	0.575	0.105	-0.470
2	AAAAA	0.522	0.096	-0.426	AAAAA	0.522	0.098	-0.424	AAAAA	0.522	0.126	-0.395
3	TAAAA	0.510	0.092	-0.418	ATTTT	0.555	0.169	-0.387	TTTTA	0.530	0.203	-0.327
4	TTAAA	0.544	0.155	-0.389	TTAAA	0.544	0.171	-0.373	ATTTT	0.555	0.234	-0.321
5	AAAAT	0.518	0.143	-0.375	TAAAA	0.510	0.142	-0.368	TAAAA	0.510	0.205	-0.305
6	TTTAA	0.547	0.176	-0.372	AAAAT	0.518	0.152	-0.365	TTTAA	0.547	0.249	-0.298
7	ATAAA	0.458	0.095	-0.362	TTTTA	0.530	0.176	-0.354	AAAAT	0.518	0.229	-0.289
8	TAAAT	0.470	0.130	-0.340	ATAAA	0.458	0.122	-0.336	TTAAA	0.544	0.268	-0.276
9	ATTTT	0.555	0.218	-0.338	TTTAA	0.547	0.228	-0.319	AATTT	0.469	0.208	-0.261
10	TTTTA	0.530	0.195	-0.336	AAATT	0.453	0.138	-0.315	TTTAT	0.467	0.215	-0.252

Over-represented 5-mers

	Template				Complement				2D			
Rank	kmer	Ref %	Read %	Diff %	kmer	Ref %	Read %	Diff %	kmer	Ref %	Read %	Diff %
1	CGGGC	0.006	0.185	0.179	CGGCT	0.018	0.175	0.157	CGGGC	0.006	0.072	0.066
2	CGGCT	0.018	0.146	0.128	TCGGC	0.018	0.167	0.148	CATGC	0.050	0.116	0.066
3	AGGCT	0.034	0.159	0.125	GCTGC	0.045	0.159	0.114	AAGGC	0.040	0.101	0.061
4	GGCTG	0.021	0.143	0.122	GCGGC	0.011	0.124	0.113	GGCTT	0.048	0.109	0.060
5	GGCTC	0.020	0.137	0.116	TCGTA	0.075	0.186	0.111	CGCAG	0.017	0.077	0.059
6	GCTGC	0.045	0.158	0.113	CTTCG	0.043	0.152	0.109	GGATC	0.033	0.091	0.059
7	TAGGC	0.029	0.139	0.110	CGGCG	0.009	0.116	0.108	CGACC	0.020	0.079	0.059
8	ATGCG	0.046	0.154	0.109	GATCG	0.024	0.131	0.106	GGCAG	0.019	0.077	0.057
9	TCGGC	0.018	0.125	0.106	TCGTG	0.043	0.148	0.105	GCAGG	0.032	0.089	0.057
10	GGCTT	0.048	0.153	0.105	GCGTG	0.023	0.127	0.104	GCATG	0.049	0.106	0.057

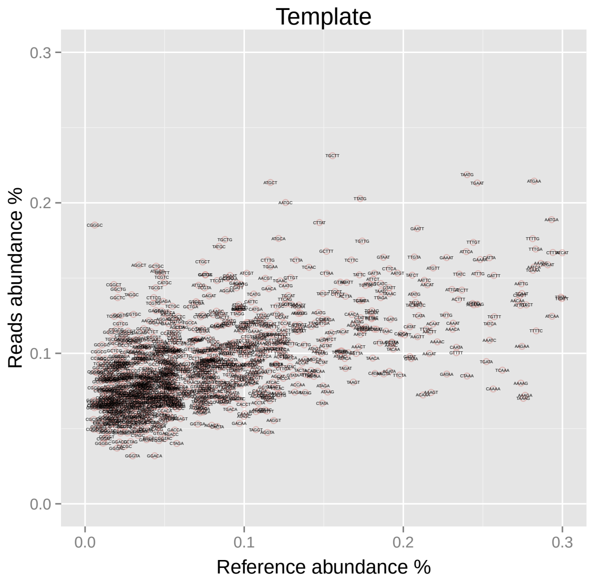

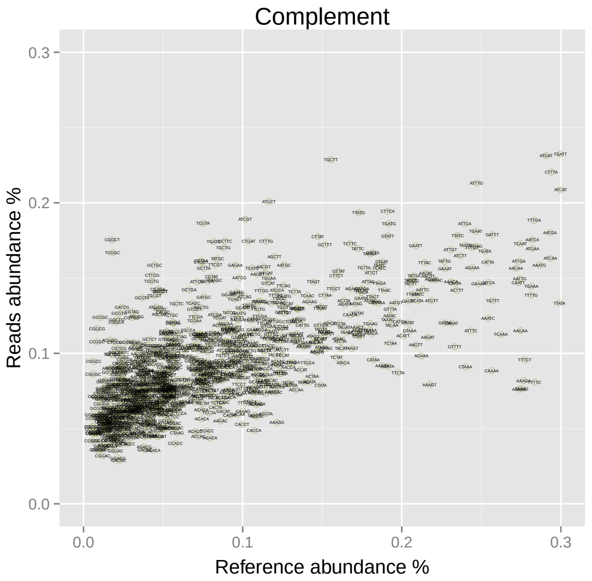

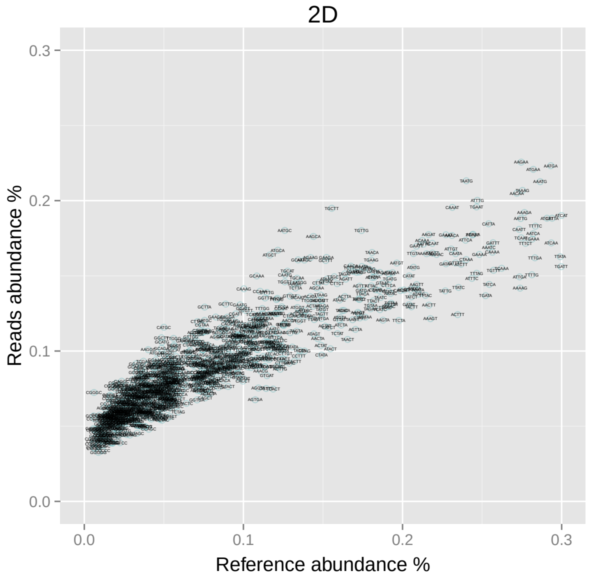

Staphylococcus epidermidis GC content

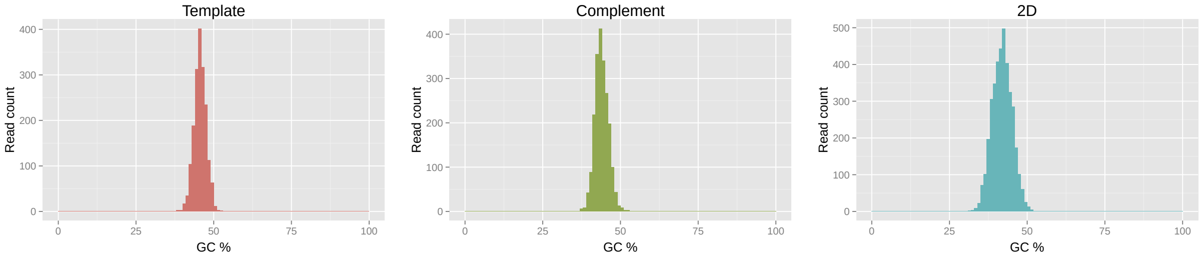

Streptococcus agalactiae error analysis

	Template	Complement	2D
Overall base identity (excluding indels)	67.70%	69.94%	79.32%
Aligned base identity (excluding indels)	76.60%	78.53%	86.24%
Identical bases per 100 aligned bases (including indels)	63.14%	63.56%	76.03%
Inserted bases per 100 aligned bases (including indels)	3.53%	2.40%	5.88%
Deleted bases per 100 aligned bases (including indels)	14.04%	16.67%	5.97%
Substitutions per 100 aligned bases (including indels)	19.29%	17.38%	12.13%
Mean insertion size	1.49	1.37	1.54
Mean deletion size	1.77	1.87	1.45

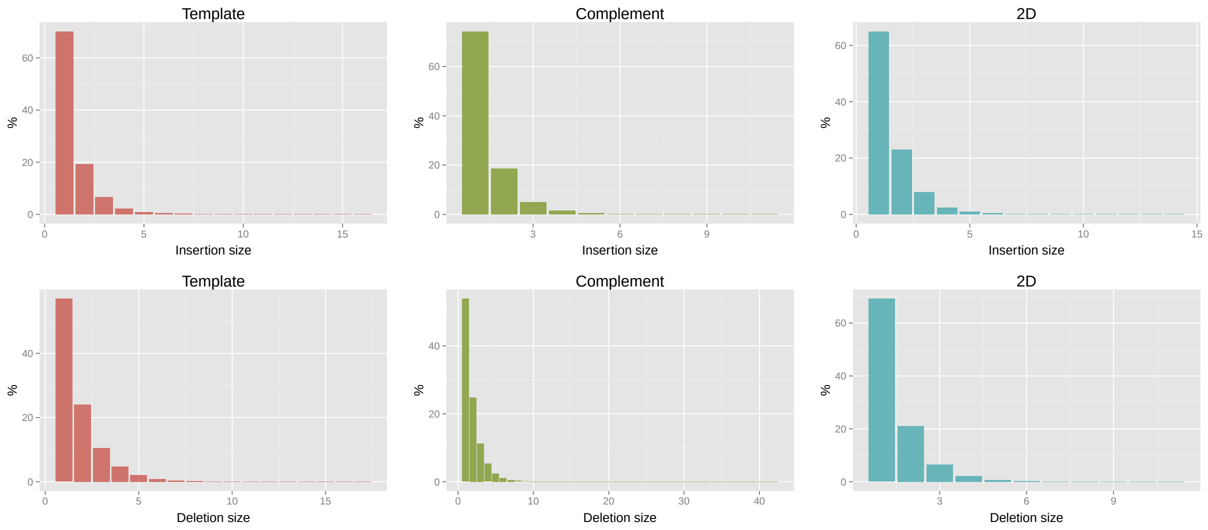

Streptococcus agalactiae read identity

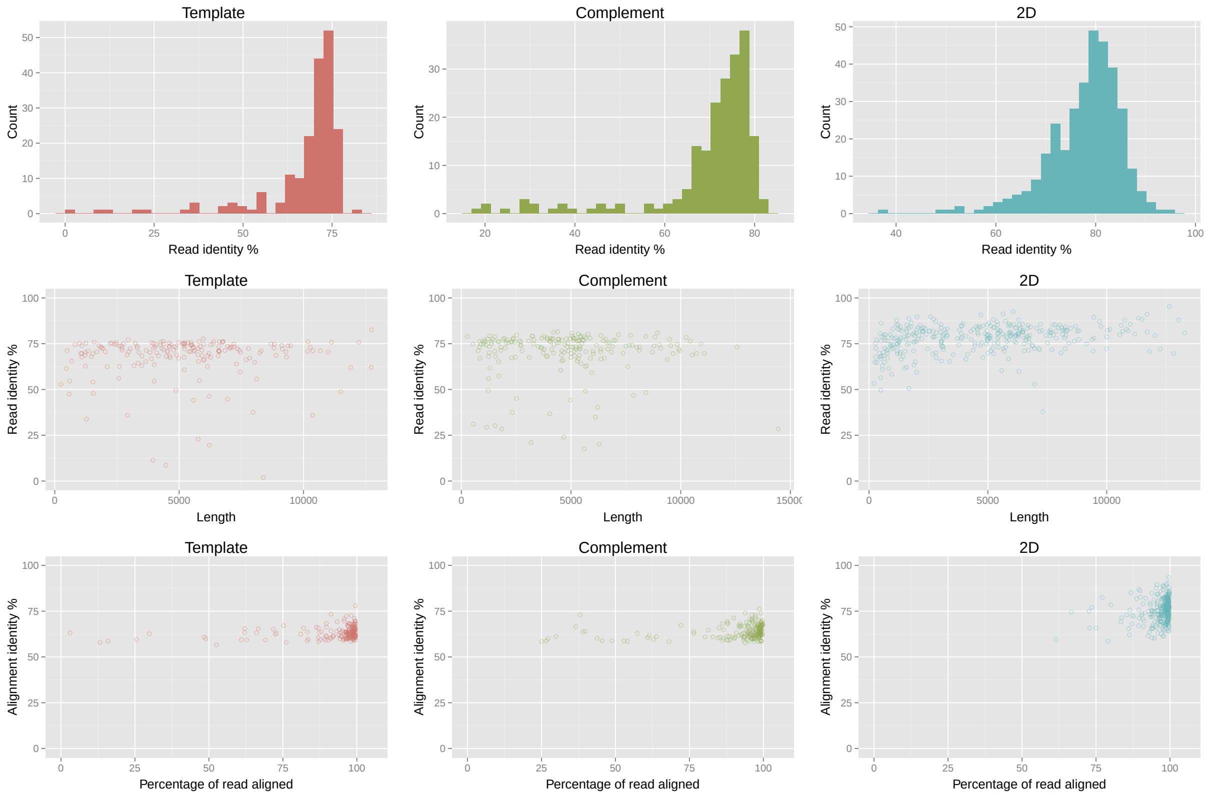

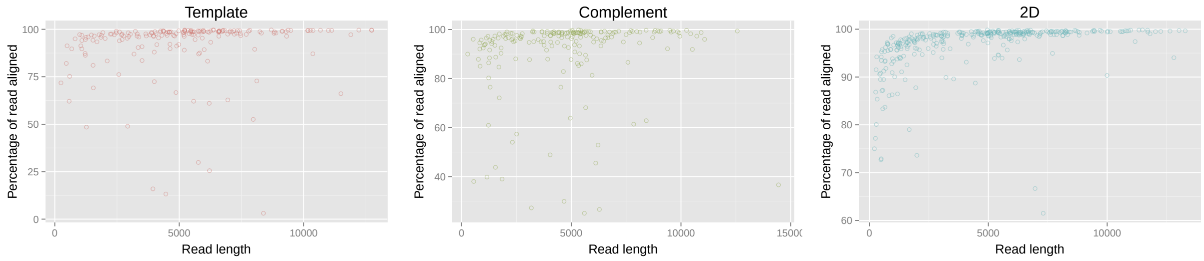

Streptococcus agalactiae perfect kmers

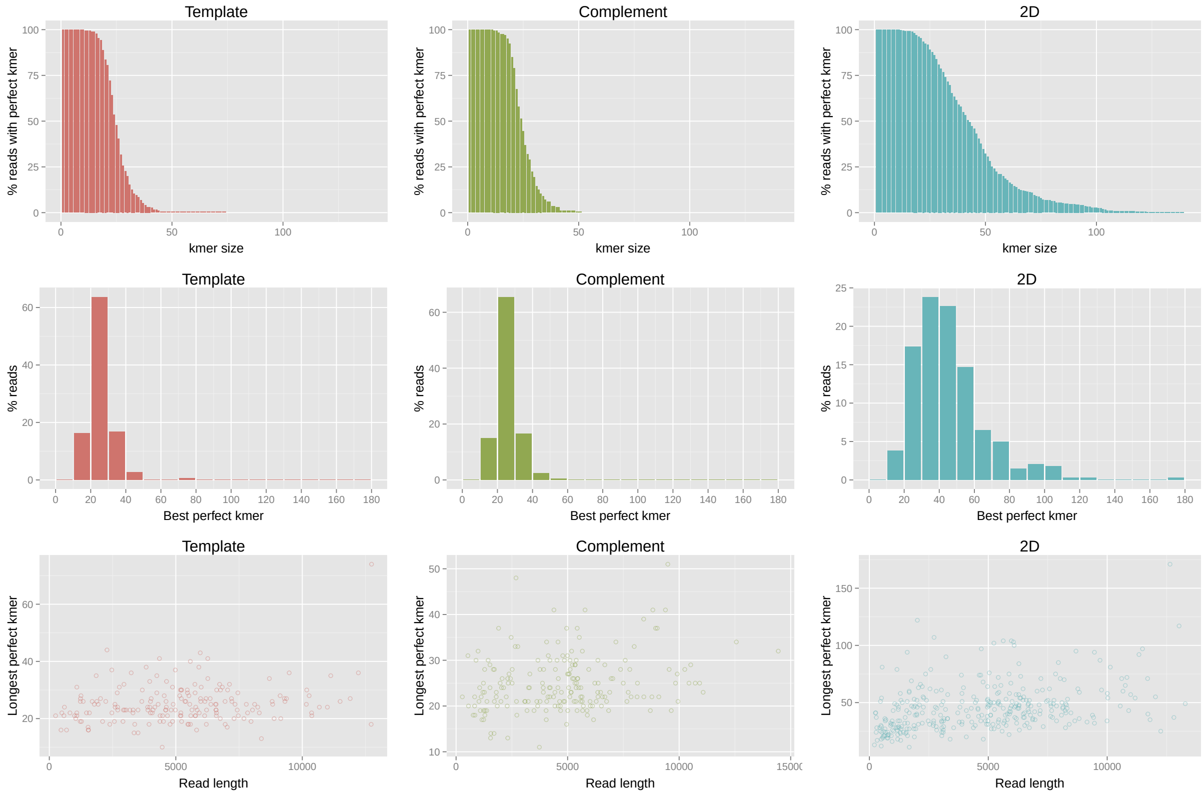

Streptococcus agalactiae coverage

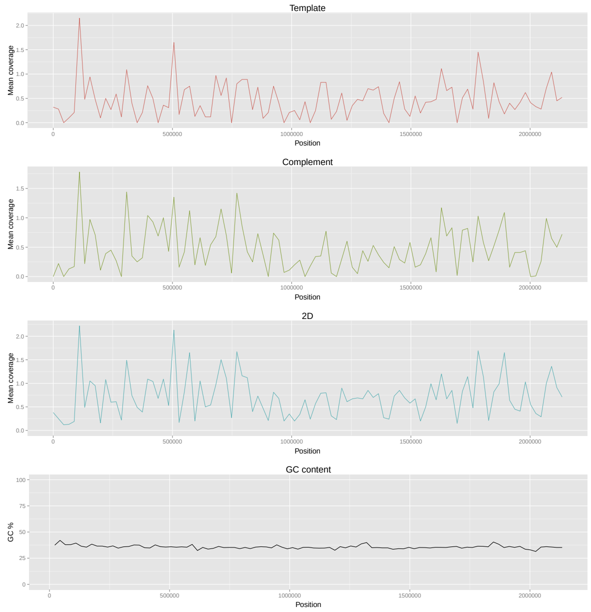

Streptococcus agalactiae 5-mer analysis

Under-represented 5-mers

	Template				Complement				2D			
Rank	kmer	Ref %	Read %	Diff %	kmer	Ref %	Read %	Diff %	kmer	Ref %	Read %	Diff %
1	AAAAA	0.580	0.104	-0.477	AAAAA	0.580	0.093	-0.487	TTTTT	0.574	0.127	-0.447
2	TTTTT	0.574	0.140	-0.434	TTTTT	0.574	0.114	-0.460	AAAAA	0.580	0.150	-0.431
3	TAAAA	0.451	0.083	-0.368	AAAAT	0.493	0.139	-0.354	TAAAA	0.451	0.201	-0.251
4	AAAAT	0.493	0.135	-0.358	TAAAA	0.451	0.120	-0.331	AAAAT	0.493	0.248	-0.245
5	ATAAA	0.362	0.087	-0.274	ATTTT	0.475	0.185	-0.290	TTTTA	0.429	0.206	-0.223
6	CAAAA	0.324	0.082	-0.243	ATAAA	0.362	0.103	-0.259	ATTTT	0.475	0.258	-0.217
7	ATTTT	0.475	0.239	-0.236	TTTTA	0.429	0.181	-0.248	AAAAG	0.317	0.169	-0.148
8	TTTTA	0.429	0.194	-0.235	CAAAA	0.324	0.086	-0.238	AATTT	0.353	0.209	-0.144
9	AAATT	0.361	0.130	-0.231	AAATT	0.361	0.125	-0.236	AAATA	0.369	0.227	-0.143
10	AAAAG	0.317	0.088	-0.229	AAAAG	0.317	0.082	-0.235	ATAAA	0.362	0.220	-0.142

Over-represented 5-mers

	Template				Complement				2D			
Rank	kmer	Ref %	Read %	Diff %	kmer	Ref %	Read %	Diff %	kmer	Ref %	Read %	Diff %
1	CGGGC	0.009	0.171	0.162	TCGGC	0.022	0.164	0.142	TCGAC	0.029	0.082	0.053
2	CGGCT	0.029	0.143	0.115	CGGCT	0.029	0.171	0.142	ATGCA	0.076	0.127	0.051
3	TCGTC	0.059	0.161	0.102	CTTCG	0.045	0.153	0.108	GGATC	0.037	0.087	0.050
4	TCGGC	0.022	0.123	0.101	TCGTG	0.053	0.161	0.108	CGCAG	0.023	0.073	0.050
5	ATGCG	0.033	0.132	0.099	CGGCG	0.012	0.118	0.106	CATGC	0.045	0.095	0.050
6	GGCTC	0.035	0.131	0.096	GTCGT	0.053	0.156	0.104	CGGGC	0.009	0.058	0.049
7	CTTCG	0.045	0.141	0.096	TCGTA	0.072	0.176	0.104	GCATG	0.042	0.091	0.049
8	CGTCG	0.024	0.120	0.095	GCGGC	0.016	0.118	0.102	ATGCG	0.033	0.081	0.048
9	TGCGT	0.035	0.131	0.095	GCGTA	0.034	0.134	0.100	CGACC	0.027	0.073	0.046
10	GCTGC	0.067	0.161	0.095	CGTAG	0.032	0.130	0.098	AACCC	0.044	0.090	0.046

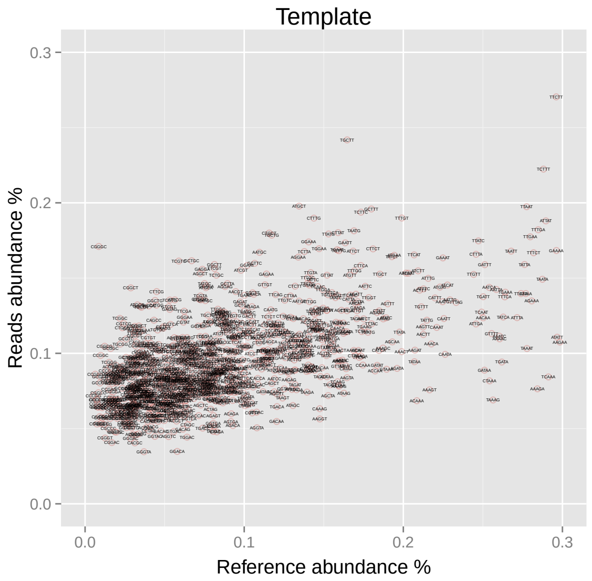

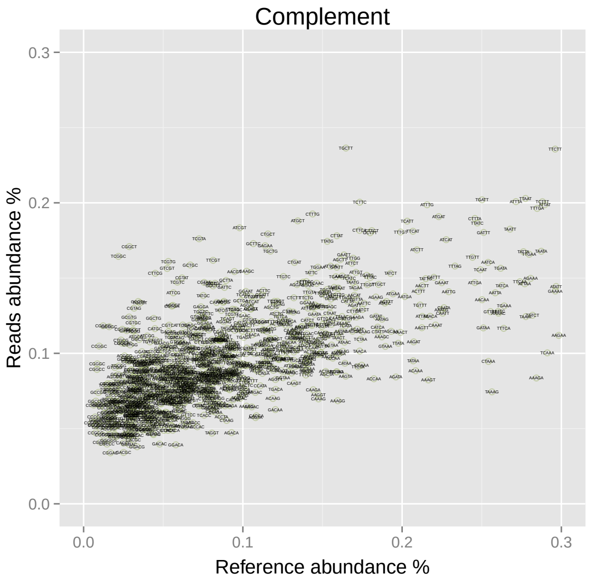

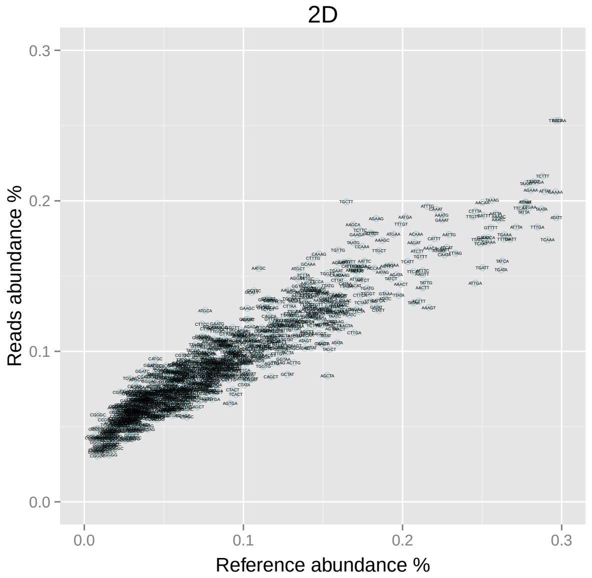

Streptococcus agalactiae GC content

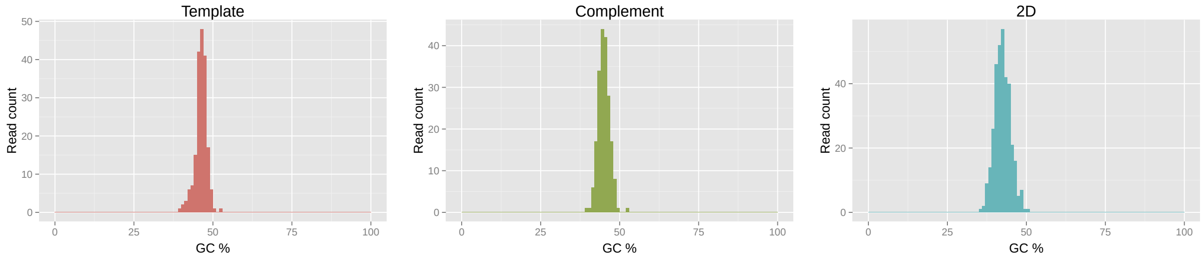

Streptococcus mutans error analysis

	Template	Complement	2D
Overall base identity (excluding indels)	69.59%	71.10%	78.39%
Aligned base identity (excluding indels)	77.17%	79.56%	86.75%
Identical bases per 100 aligned bases (including indels)	63.90%	64.53%	76.49%
Inserted bases per 100 aligned bases (including indels)	3.36%	2.40%	5.87%
Deleted bases per 100 aligned bases (including indels)	13.84%	16.49%	5.95%
Substitutions per 100 aligned bases (including indels)	18.90%	16.58%	11.69%
Mean insertion size	1.45	1.37	1.55
Mean deletion size	1.75	1.87	1.45

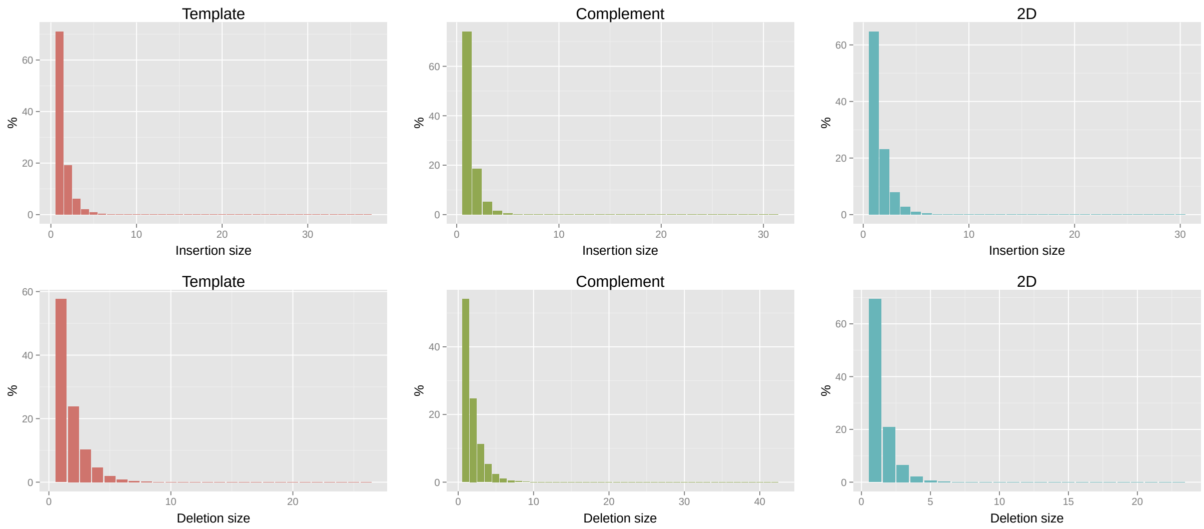

Streptococcus mutans read identity

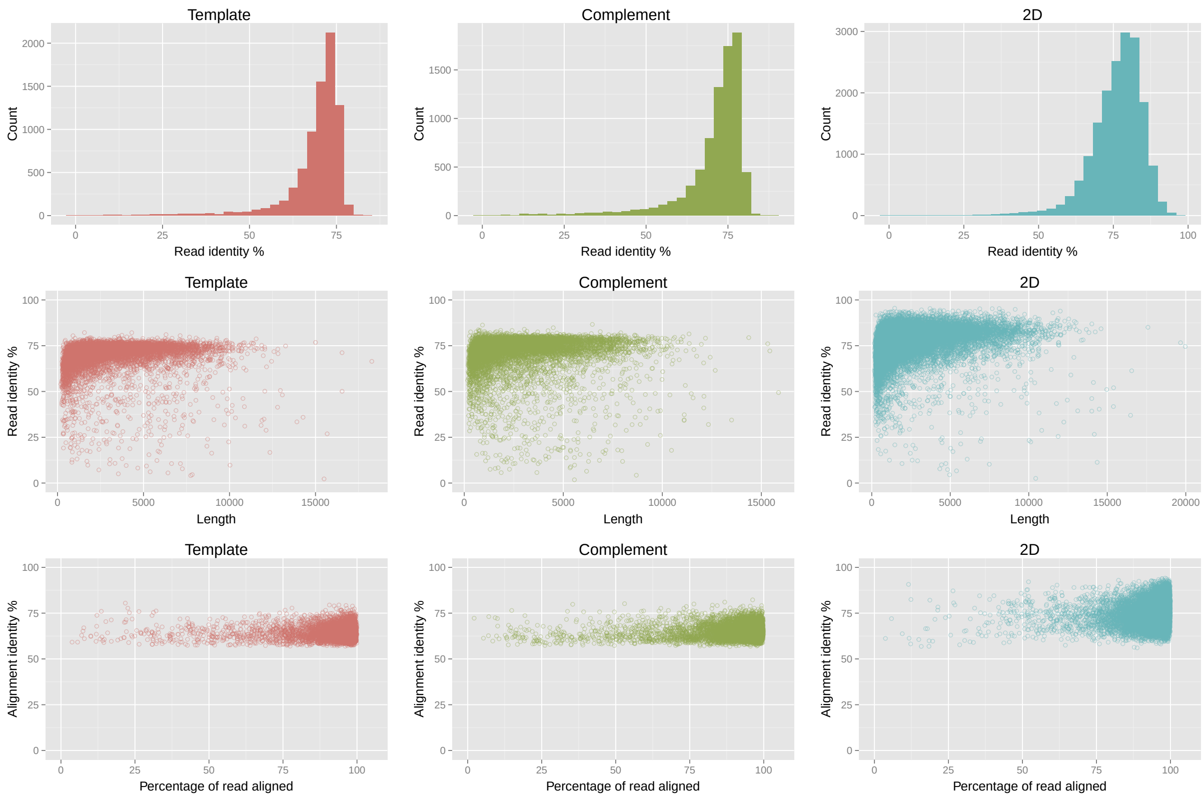

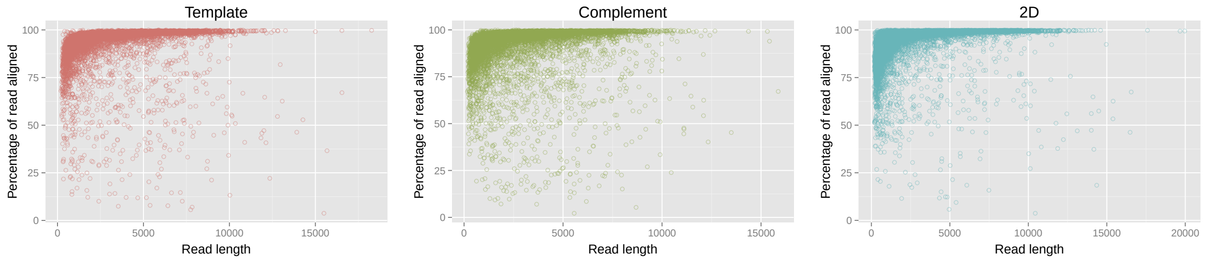

Streptococcus mutans perfect kmers

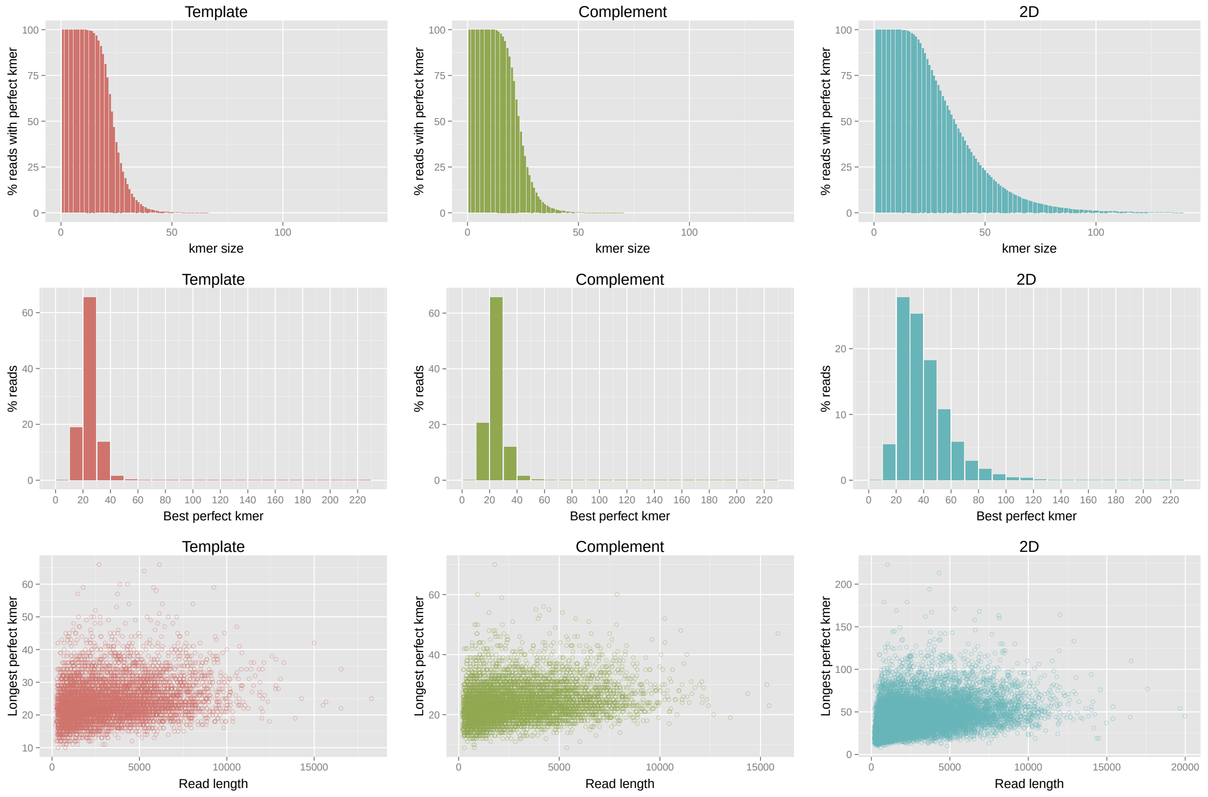

Streptococcus mutans coverage

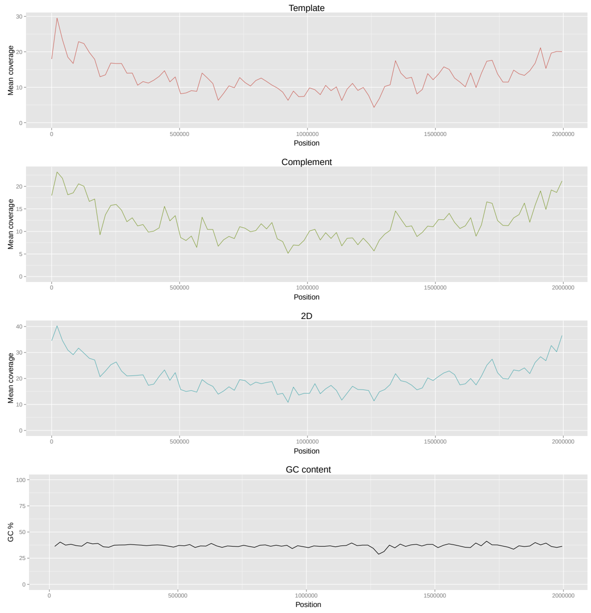

Streptococcus mutans 5-mer analysis

Under-represented 5-mers

	Template				Complement				2D			
Rank	kmer	Ref %	Read %	Diff %	kmer	Ref %	Read %	Diff %	kmer	Ref %	Read %	Diff %
1	AAAAA	0.605	0.105	-0.500	TTTTT	0.632	0.111	-0.521	TTTTT	0.632	0.133	-0.499
2	TTTTT	0.632	0.148	-0.484	AAAAA	0.605	0.107	-0.498	AAAAA	0.605	0.152	-0.452
3	AAAAT	0.498	0.142	-0.355	AAAAT	0.498	0.154	-0.344	AAAAT	0.498	0.245	-0.253
4	TAAAA	0.435	0.085	-0.350	ATTTT	0.508	0.183	-0.324	ATTTT	0.508	0.262	-0.246
5	AAAAG	0.371	0.092	-0.278	TAAAA	0.435	0.126	-0.310	TAAAA	0.435	0.194	-0.241
6	CAAAA	0.360	0.087	-0.273	AAAAG	0.371	0.091	-0.280	TTTTA	0.436	0.203	-0.232
7	ATTTT	0.508	0.246	-0.261	CAAAA	0.360	0.096	-0.264	AAAAG	0.371	0.176	-0.195
8	AAATT	0.389	0.131	-0.257	TTTTA	0.436	0.181	-0.255	CTTTT	0.383	0.198	-0.185
9	ATAAA	0.340	0.084	-0.256	AAATT	0.389	0.143	-0.245	AATTT	0.394	0.225	-0.169
10	AAAGA	0.316	0.079	-0.237	TTTTC	0.357	0.113	-0.244	CAAAA	0.360	0.202	-0.158

Over-represented 5-mers

	Template				Complement				2D			
Rank	kmer	Ref %	Read %	Diff %	kmer	Ref %	Read %	Diff %	kmer	Ref %	Read %	Diff %
1	CGGGC	0.016	0.165	0.149	TCGGC	0.029	0.162	0.133	TCGAC	0.021	0.079	0.057
2	TCGTC	0.053	0.159	0.106	CGGCT	0.044	0.169	0.125	CGCAG	0.034	0.083	0.049
3	CGTCG	0.020	0.118	0.098	TCGTA	0.054	0.175	0.121	CGGGC	0.016	0.065	0.049
4	CGGCT	0.044	0.143	0.098	CTTCG	0.041	0.153	0.112	CGAAG	0.037	0.086	0.048
5	CTTCG	0.041	0.139	0.098	GTCGT	0.047	0.151	0.104	GTCGA	0.021	0.068	0.048
6	TCGGC	0.029	0.123	0.094	TCGTG	0.048	0.148	0.100	TCGAT	0.036	0.083	0.047
7	TAGGC	0.042	0.131	0.089	GCGTA	0.031	0.128	0.097	CGTAG	0.018	0.063	0.046
8	GTCGT	0.047	0.134	0.087	CGTAG	0.018	0.114	0.097	CCTGG	0.014	0.060	0.046
9	TGCGT	0.039	0.125	0.086	CGTAT	0.050	0.146	0.096	CATGC	0.054	0.099	0.045
10	CTGCG	0.035	0.120	0.086	CGGCG	0.021	0.116	0.095	ATGCA	0.069	0.114	0.045

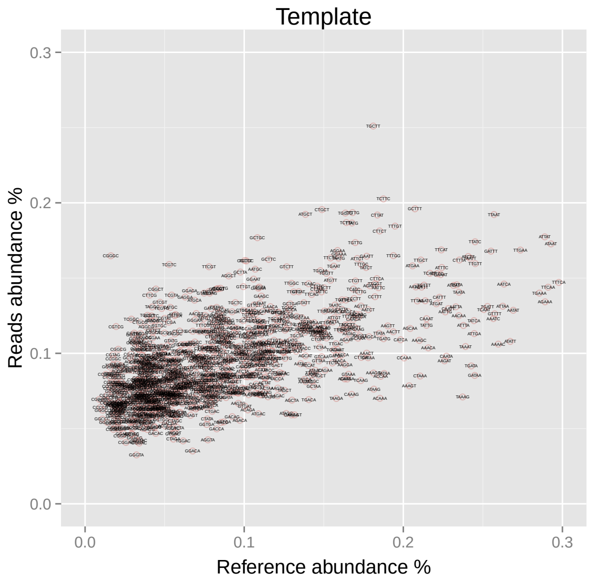

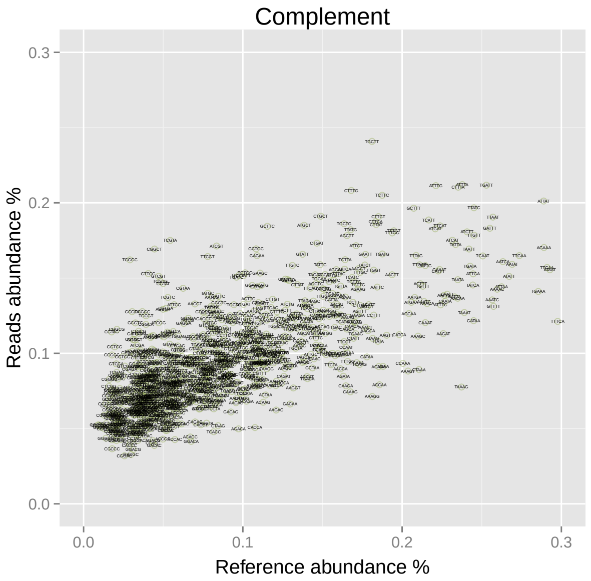

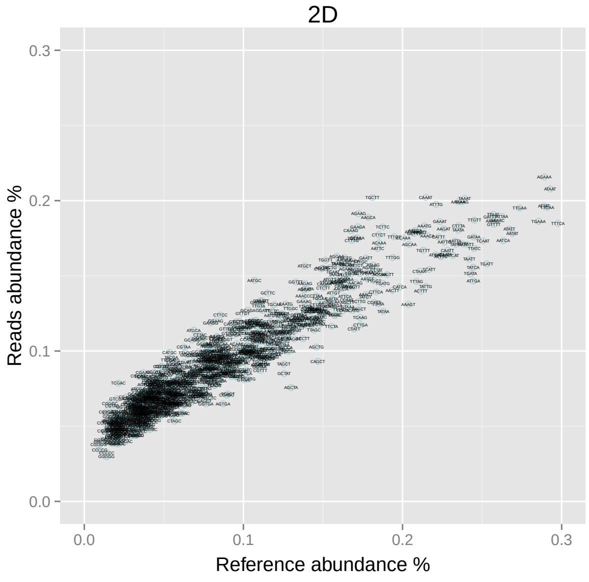

Streptococcus mutans GC content

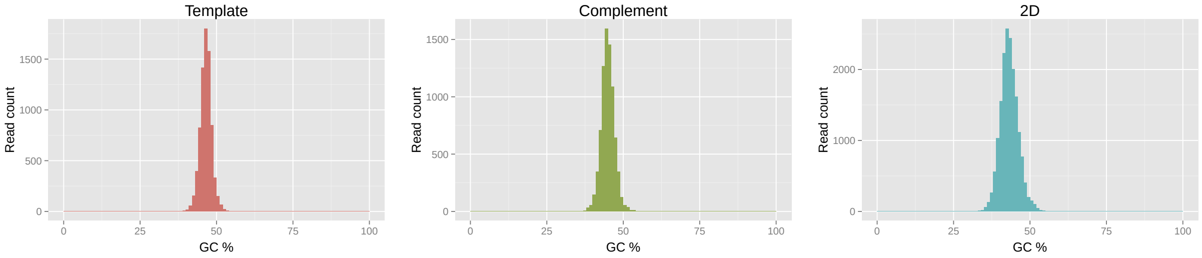

Streptococcus pneumoniae error analysis

	Template	Complement	2D
Overall base identity (excluding indels)	38.13%	32.94%	72.69%
Aligned base identity (excluding indels)	74.54%	76.74%	86.34%
Identical bases per 100 aligned bases (including indels)	62.75%	63.54%	75.95%
Inserted bases per 100 aligned bases (including indels)	4.48%	3.53%	6.42%
Deleted bases per 100 aligned bases (including indels)	11.34%	13.67%	5.62%
Substitutions per 100 aligned bases (including indels)	21.43%	19.26%	12.01%
Mean insertion size	1.45	1.37	1.55
Mean deletion size	1.62	1.75	1.42

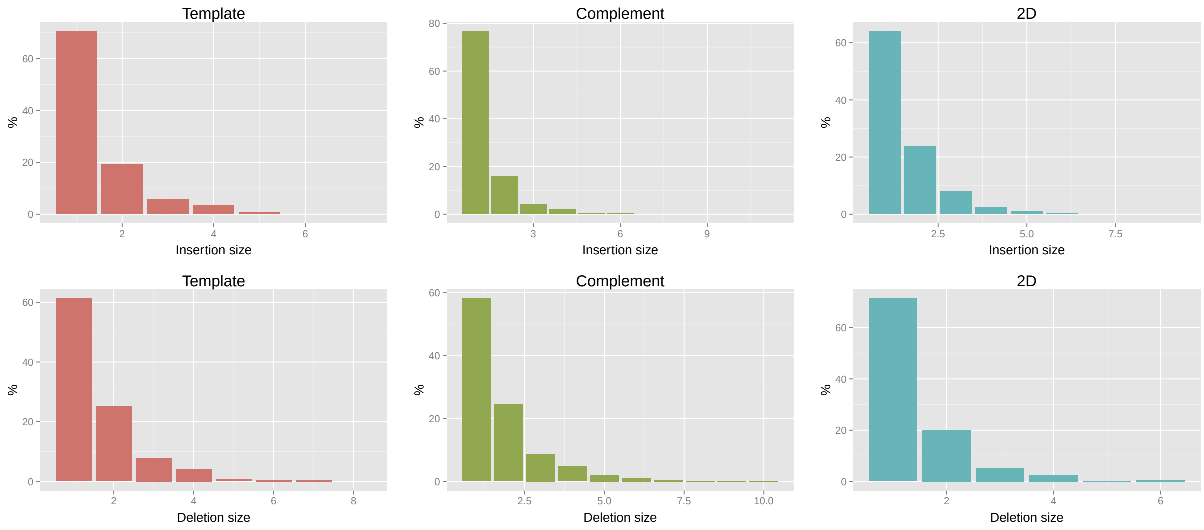

Streptococcus pneumoniae read identity

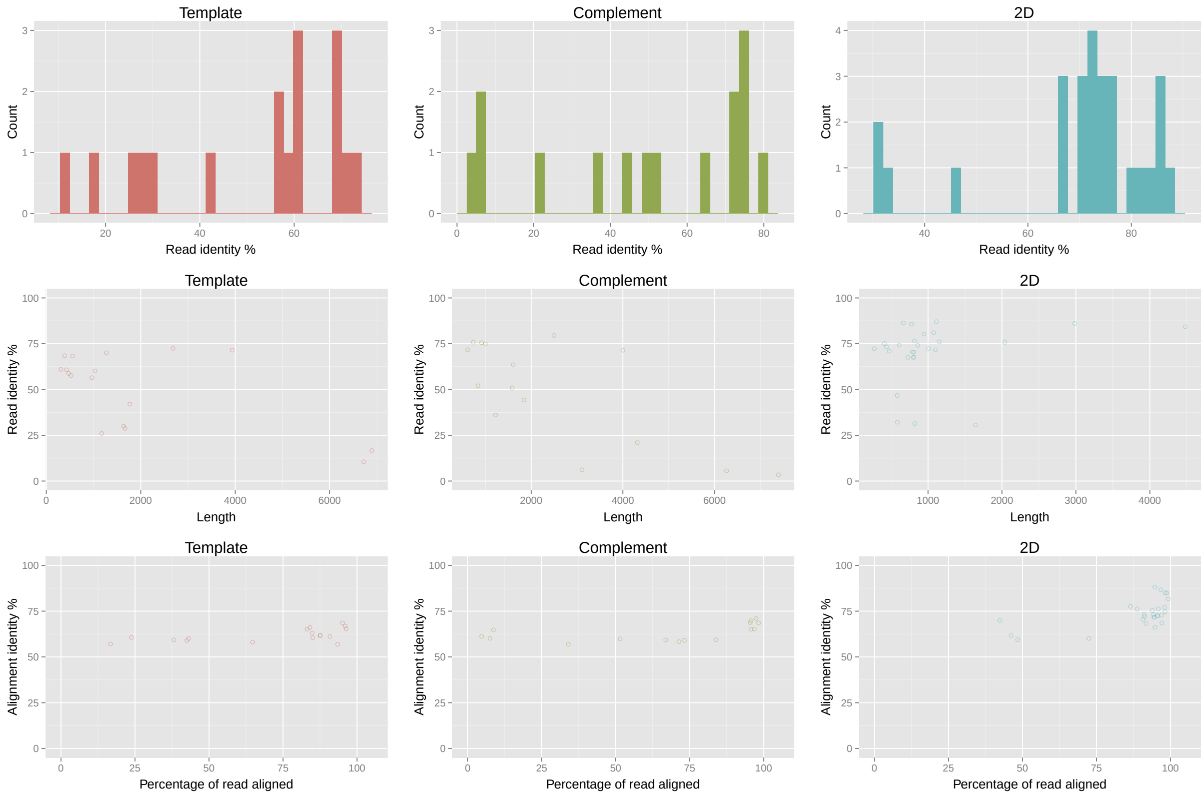

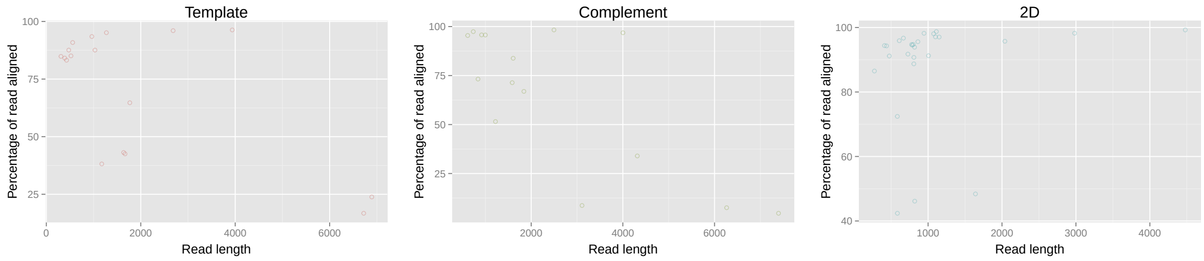

Streptococcus pneumoniae perfect kmers

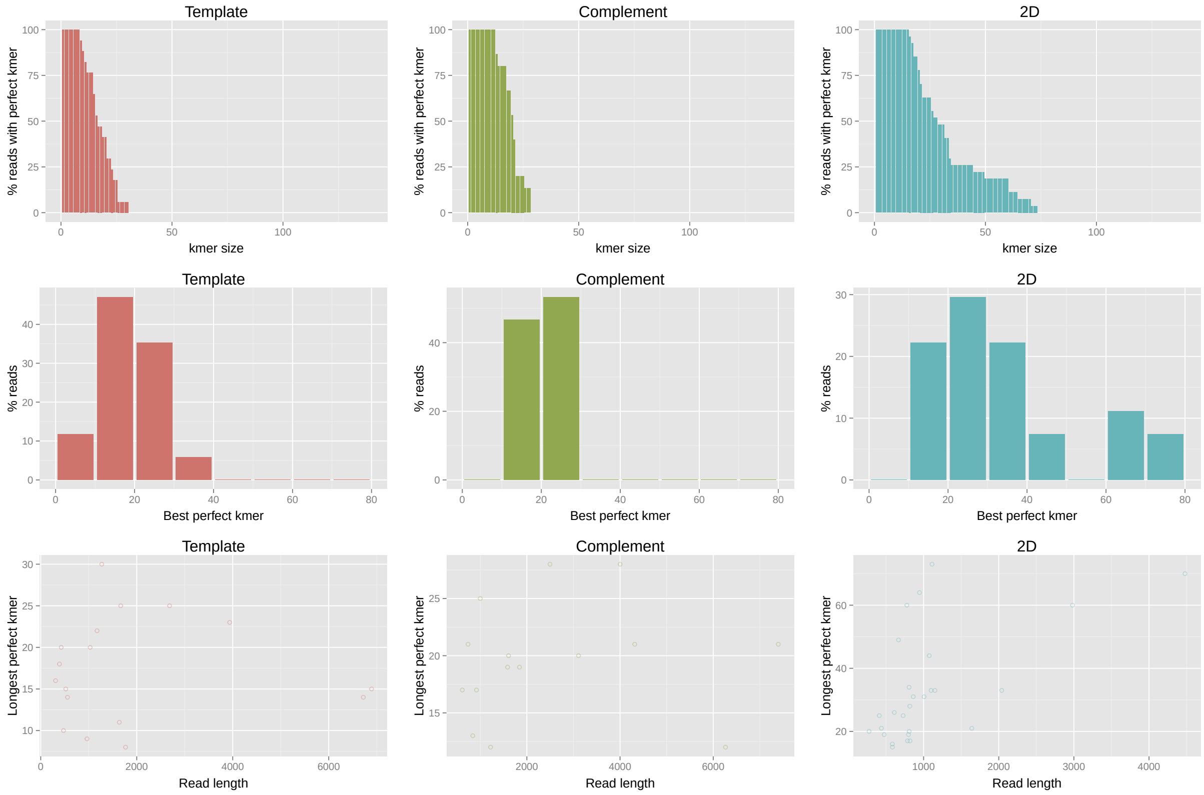

Streptococcus pneumoniae coverage

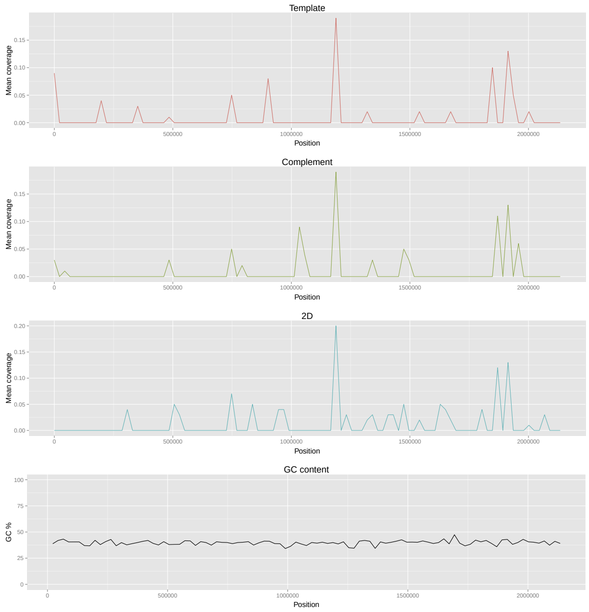

Streptococcus pneumoniae 5-mer analysis

Under-represented 5-mers

	Template				Complement				2D			
Rank	kmer	Ref %	Read %	Diff %	kmer	Ref %	Read %	Diff %	kmer	Ref %	Read %	Diff %
1	AAAAA	0.478	0.124	-0.354	AAAAA	0.478	0.103	-0.375	AAAAA	0.478	0.098	-0.380
2	TTTTT	0.456	0.136	-0.320	TTTTT	0.456	0.098	-0.358	TTTTT	0.456	0.091	-0.365
3	AAAAT	0.408	0.130	-0.278	AAAAT	0.408	0.169	-0.239	AAAAT	0.408	0.161	-0.247
4	TAAAA	0.295	0.053	-0.243	TTTTC	0.316	0.092	-0.224	GAAAA	0.343	0.168	-0.175
5	TCAAA	0.296	0.068	-0.228	AAGAA	0.331	0.111	-0.220	AAAAG	0.288	0.119	-0.169
6	AAAGA	0.300	0.080	-0.219	ATTTT	0.390	0.174	-0.216	TAAAA	0.295	0.129	-0.166
7	ATTTT	0.390	0.186	-0.204	TCAAA	0.296	0.082	-0.214	ATTTT	0.390	0.230	-0.159
8	CAAAA	0.285	0.083	-0.202	CAAAA	0.285	0.074	-0.211	TTTTG	0.282	0.147	-0.136
9	AAAAG	0.288	0.087	-0.201	AAAGA	0.300	0.092	-0.207	TCAAA	0.296	0.161	-0.135
10	ATAAA	0.253	0.059	-0.195	TAAAA	0.295	0.092	-0.203	AAAGA	0.300	0.168	-0.132

Over-represented 5-mers

	Template				Complement				2D			
Rank	kmer	Ref %	Read %	Diff %	kmer	Ref %	Read %	Diff %	kmer	Ref %	Read %	Diff %
1	CGGGC	0.014	0.182	0.169	CGGGC	0.014	0.180	0.166	GTCGA	0.041	0.119	0.078
2	GCTGC	0.070	0.195	0.125	CGGCT	0.039	0.195	0.156	CACCC	0.035	0.108	0.074
3	ATGCG	0.045	0.158	0.113	CTGCG	0.033	0.187	0.155	TAACG	0.048	0.119	0.071
4	GCGTG	0.034	0.145	0.111	TGCGT	0.039	0.193	0.154	ACGAC	0.059	0.126	0.067
5	CTGCG	0.033	0.142	0.110	GCGTC	0.033	0.182	0.149	CGTTG	0.060	0.126	0.066
6	GCGTC	0.033	0.142	0.109	GCGGC	0.022	0.161	0.139	ACGAT	0.090	0.154	0.064
7	TCGGC	0.027	0.136	0.109	TCGTC	0.070	0.209	0.138	GTGCT	0.066	0.129	0.063
8	CCGGC	0.009	0.117	0.108	CGGCG	0.015	0.145	0.131	AACGC	0.049	0.112	0.063
9	TGCGT	0.039	0.145	0.106	CGTCG	0.032	0.161	0.130	ACCCC	0.025	0.087	0.062
10	GGGGA	0.043	0.148	0.106	CGTGC	0.035	0.164	0.129	TCGAC	0.040	0.101	0.062

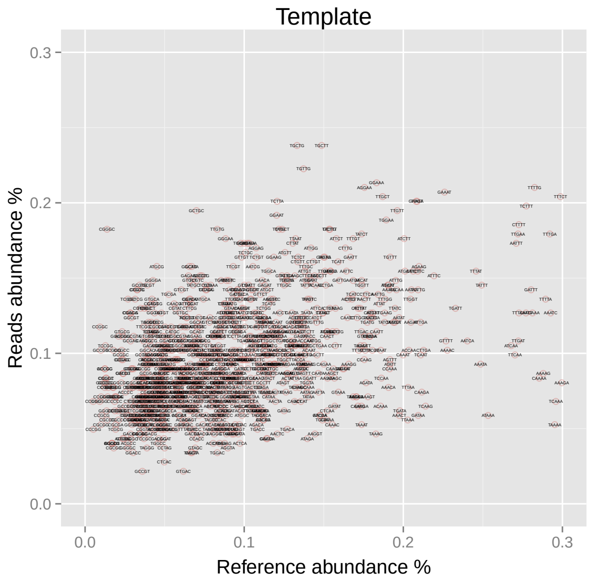

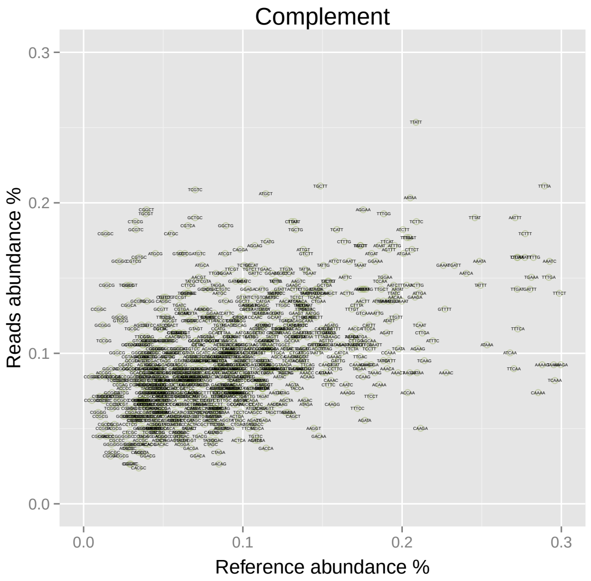

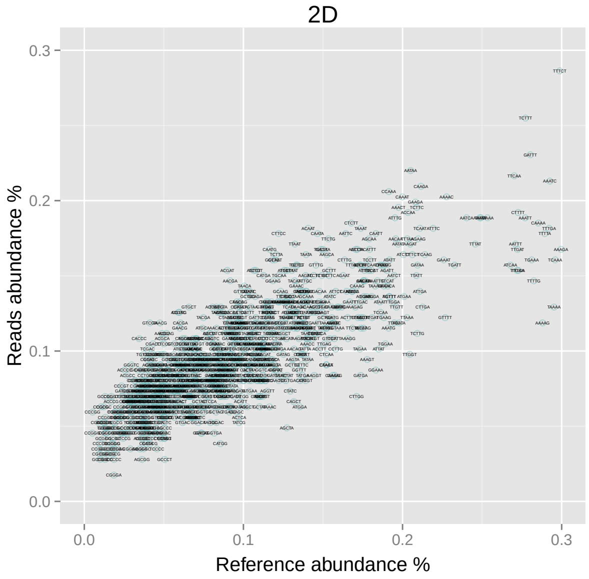

Streptococcus pneumoniae GC content

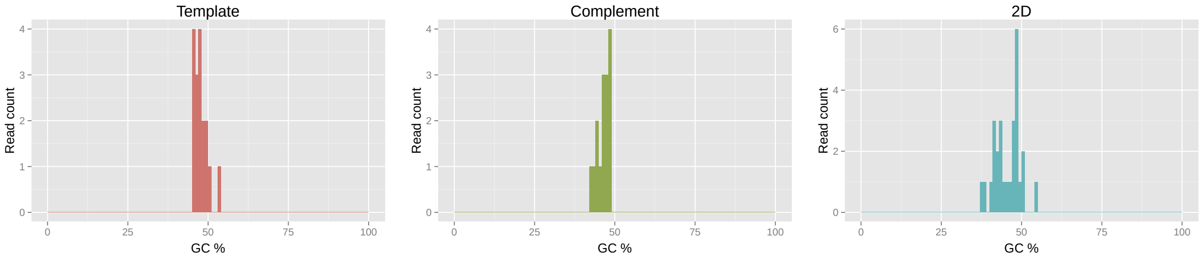

All reference 21mer analysis

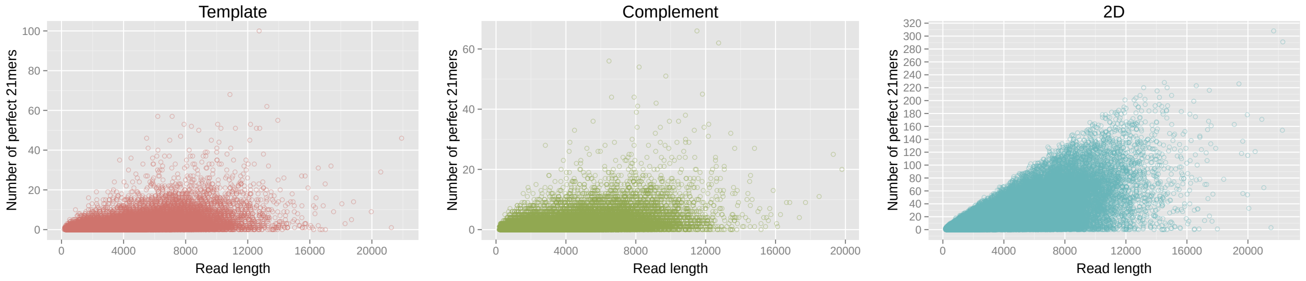

All reference substitutions

		Template substituted %				Complement substituted %				2D substituted %			
		a	c	g	t	a	c	g	t	a	c	g	t
Reference	A	0.00	9.74	10.39	4.84	0.00	9.75	9.52	4.96	0.00	9.39	9.23	4.29
	C	8.00	0.00	8.47	8.67	8.88	0.00	8.02	8.88	8.79	0.00	9.55	8.80
	G	8.57	8.35	0.00	7.92	8.73	7.94	0.00	8.85	8.72	9.45	0.00	8.76
	T	4.87	10.39	9.80	0.00	4.98	9.63	9.85	0.00	4.31	9.27	9.45	0.00

Kmer motifs before errors

3-mer error motif analysis

	Template			Complement			2D			
Rank	Insertion	Deletion	Substitution	Insertion	Deletion	Substitution	Insertion	Deletion	Substitution	
1	AAA (3.12%)	AAA (3.94%)	AAA (4.03%)	AAA (2.89%)	AAA (3.62%)	AAA (3.73%)	AAA (3.26%)	AAA (5.94%)	AAA (4.38%)	Most common
2	TTC (3.09%)	TTC (3.50%)	TTC (3.61%)	TTT (2.68%)	TTT (3.09%)	GAA (3.15%)	TTT (2.98%)	TTT (5.07%)	TTT (3.33%)	
3	TTT (2.92%)	TTT (3.15%)	GAA (3.19%)	GAA (2.42%)	GAA (2.92%)	GCA (2.91%)	TTC (2.73%)	GAA (2.92%)	GAA (3.29%)	
4	GCA (2.51%)	GAA (2.78%)	GCA (3.09%)	TTC (2.41%)	TTC (2.54%)	TTT (2.75%)	GAA (2.54%)	TTC (2.62%)	GCA (2.98%)	
5	GAA (2.39%)	TGC (2.53%)	TTT (2.95%)	GCA (2.26%)	TGC (2.36%)	TTC (2.71%)	GCA (2.45%)	GCA (2.31%)	TTC (2.79%)	
6	TCA (2.23%)	GCA (2.52%)	AAT (2.50%)	TGC (2.24%)	GCA (2.33%)	TCA (2.51%)	GCC (2.37%)	GCC (2.30%)	AAT (2.54%)	
7	TGC (2.23%)	TCA (2.33%)	TCA (2.39%)	GGC (2.18%)	GCC (2.24%)	TGC (2.27%)	TCA (2.14%)	CAA (2.17%)	GCC (2.38%)	
8	AAT (2.23%)	GCC (2.27%)	TGC (2.24%)	TCA (2.15%)	ATC (2.24%)	ATC (2.21%)	GCG (2.13%)	ATT (2.15%)	TCA (2.26%)	
9	GCC (2.17%)	AAT (2.15%)	CAA (2.21%)	ATC (2.04%)	GGC (2.23%)	GGC (2.16%)	CGC (2.07%)	TGC (2.04%)	GTT (2.18%)	
10	TCT (2.10%)	ATT (2.14%)	GCC (2.17%)	ATT (2.00%)	ATT (2.13%)	AAT (2.14%)	TGC (2.07%)	TCA (2.01%)	CGC (2.05%)	
	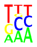	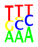	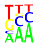	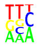	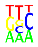	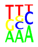	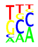	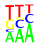	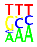	
-10	GTG (1.02%)	ACT (0.92%)	GAC (0.91%)	ACT (1.01%)	AGG (0.95%)	TAC (0.91%)	CGA (1.06%)	GAG (1.01%)	GAG (0.94%)	Least common
-9	GTA (1.00%)	CCC (0.92%)	AGG (0.89%)	CAC (1.01%)	ACT (0.93%)	CCC (0.89%)	CCC (1.05%)	GGG (0.94%)	CGA (0.84%)	
-8	GAG (0.95%)	AGT (0.92%)	CCC (0.88%)	GAG (0.97%)	AGT (0.91%)	AGT (0.88%)	TAC (1.01%)	AGT (0.87%)	GGG (0.81%)	
-7	AGG (0.93%)	GTA (0.89%)	CGA (0.85%)	TAC (0.95%)	CCC (0.90%)	GGT (0.86%)	GTA (0.98%)	AGA (0.86%)	TGA (0.81%)	
-6	AGT (0.92%)	GAG (0.87%)	GGA (0.84%)	AGT (0.91%)	GGT (0.90%)	GAG (0.80%)	AGT (0.94%)	CGA (0.85%)	GTA (0.80%)	
-5	CCC (0.90%)	CGA (0.85%)	GAG (0.80%)	AGG (0.88%)	GAG (0.88%)	AGG (0.79%)	ACT (0.93%)	GTA (0.77%)	ACT (0.78%)	
-4	GGA (0.84%)	GGG (0.78%)	GGT (0.78%)	CCC (0.85%)	GTA (0.85%)	ACT (0.72%)	GGG (0.91%)	ACT (0.74%)	AGA (0.76%)	
-3	GGG (0.74%)	GGA (0.72%)	GGG (0.63%)	CTA (0.77%)	GGG (0.77%)	CTA (0.71%)	CTA (0.79%)	CTA (0.74%)	TAG (0.65%)	
-2	CTA (0.63%)	TAG (0.67%)	CTA (0.53%)	GGG (0.74%)	CTA (0.74%)	GGG (0.66%)	GGA (0.73%)	TAG (0.73%)	CTA (0.65%)	
-1	TAG (0.56%)	CTA (0.60%)	TAG (0.51%)	TAG (0.62%)	TAG (0.64%)	TAG (0.50%)	TAG (0.69%)	GGA (0.62%)	GGA (0.53%)	
	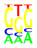	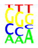	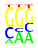	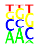	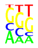	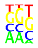	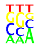	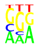	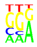	

Kmer space for 3-mers: 64 Random chance for any given 3-mer: 1.56%

4-mer error motif analysis

Rank	Template			Complement			2D			
	Insertion	Deletion	Substitution	Insertion	Deletion	Substitution	Insertion	Deletion	Substitution	
1	AAAA (1.08%)	AAAA (1.45%)	AAAA (1.43%)	CGGC (0.90%)	AAAA (1.14%)	AAAA (1.12%)	AAAA (1.02%)	AAAA (2.37%)	AAAA (1.53%)	Most common
2	TTTT (1.07%)	TTTT (1.21%)	GAAA (1.12%)	AAAA (0.89%)	TTTT (1.03%)	AGAA (0.98%)	TTTT (0.95%)	TTTT (2.20%)	TTTT (1.20%)	
3	GAAA (0.92%)	GAAA (1.06%)	TTTT (1.07%)	TTTT (0.89%)	ATTT (0.96%)	ATCA (0.96%)	ATTT (0.89%)	TAAA (1.48%)	GAAA (1.01%)	
4	TTCT (0.88%)	TTTC (0.96%)	TTTC (1.04%)	ATCA (0.84%)	CAAA (0.96%)	CAAA (0.91%)	GAAA (0.86%)	CAAA (1.41%)	TAAA (1.00%)	
5	TTTC (0.87%)	CAAA (0.90%)	CTTC (0.98%)	ATTT (0.82%)	TAAA (0.92%)	AGCA (0.87%)	CGCC (0.86%)	ATTT (1.30%)	AGAA (0.99%)	
6	CTTC (0.83%)	CTTC (0.89%)	TTCA (0.89%)	CTTC (0.77%)	AGAA (0.89%)	TTTT (0.86%)	AACA (0.81%)	CTTT (1.22%)	TGAA (0.92%)	
7	AACA (0.82%)	TTCA (0.88%)	GGAA (0.87%)	CAAA (0.77%)	CGGC (0.87%)	TAAA (0.86%)	CTTC (0.81%)	GAAA (1.12%)	CAAA (0.92%)	
8	ATTT (0.81%)	TTCC (0.84%)	AGAA (0.86%)	TTCT (0.75%)	GAAA (0.81%)	CGGC (0.86%)	CAAA (0.76%)	AGAA (0.88%)	CGCC (0.87%)	
9	TTCA (0.78%)	ATTC (0.83%)	AGCA (0.83%)	GAAA (0.74%)	TGAA (0.80%)	TGAA (0.85%)	TAAA (0.75%)	TGAA (0.87%)	ATTT (0.86%)	
10	ATCA (0.76%)	ATTT (0.80%)	AACA (0.82%)	CTGC (0.72%)	CTTT (0.78%)	CTTC (0.83%)	GGCG (0.73%)	CGCC (0.80%)	CTTC (0.83%)	
-10	GGGT (0.14%)	GTGA (0.15%)	TAGT (0.13%)	TAGT (0.16%)	ACTC (0.16%)	GTAC (0.14%)	GGGT (0.17%)	CGTA (0.15%)	GTAG (0.14%)	Least common
-9	AGGG (0.14%)	GAGT (0.15%)	GTAG (0.13%)	GAGT (0.16%)	GGGA (0.16%)	CCCT (0.14%)	CACT (0.16%)	GTAC (0.15%)	GTGA (0.12%)	
-8	TAGT (0.14%)	GGAC (0.14%)	CGGA (0.10%)	CCCT (0.16%)	GAGT (0.15%)	AGGG (0.13%)	TAGT (0.16%)	TGGA (0.15%)	CACT (0.12%)	
-7	CGGA (0.13%)	GGGA (0.14%)	TAGG (0.10%)	GTAC (0.15%)	GTAG (0.15%)	GTAG (0.13%)	GTAG (0.16%)	TAGG (0.14%)	TAGG (0.12%)	
-6	GTAG (0.13%)	TAGG (0.13%)	AGGG (0.10%)	CACT (0.15%)	GTAC (0.14%)	GGAC (0.13%)	TAGG (0.15%)	CACT (0.13%)	TGGA (0.11%)	
-5	TAGA (0.12%)	GGGT (0.12%)	TAGA (0.10%)	GTAG (0.14%)	GGAC (0.14%)	GGGT (0.11%)	TAGA (0.15%)	TAGA (0.12%)	CCTA (0.10%)	
-4	TAGG (0.12%)	CGGA (0.11%)	GGAC (0.10%)	GGAC (0.13%)	GGGT (0.12%)	CACT (0.11%)	CGGA (0.14%)	CCTA (0.12%)	TAGA (0.09%)	
-3	GGAC (0.11%)	TAGA (0.10%)	GGGT (0.10%)	TAGG (0.10%)	CCTA (0.12%)	CCTA (0.10%)	GGGA (0.14%)	CGGA (0.12%)	GGGA (0.09%)	
-2	CCTA (0.08%)	CCTA (0.09%)	CCTA (0.08%)	CCTA (0.10%)	TAGG (0.10%)	TAGG (0.08%)	CCTA (0.12%)	GGGA (0.10%)	CGGA (0.09%)	
-1	CTAG (0.04%)	CTAG (0.05%)	CTAG (0.03%)	CTAG (0.05%)	CTAG (0.06%)	CTAG (0.05%)	CTAG (0.07%)	CTAG (0.07%)	CTAG (0.06%)	

Kmer space for 4-mers: 256 Random chance for any given 4-mer: 0.39%

5-mer error motif analysis

Rank	Template			Complement			2D			
	Insertion	Deletion	Substitution	Insertion	Deletion	Substitution	Insertion	Deletion	Substitution	
1	ATTTT (0.40%)	ATTTT (0.44%)	TCTTC (0.42%)	ATTTT (0.35%)	ATTTT (0.39%)	AAGAA (0.41%)	ATTTT (0.35%)	ATTTT (0.80%)	AAGAA (0.44%)	Most common
2	CAAAA (0.34%)	GAAAA (0.44%)	GAAAA (0.40%)	GCGGC (0.31%)	AAGAA (0.38%)	CAGCA (0.38%)	TCTTC (0.31%)	TAAAA (0.72%)	TAAAA (0.43%)	
3	TCTTC (0.32%)	CAAAA (0.41%)	AAGAA (0.39%)	TCTTC (0.30%)	ATAAA (0.38%)	ATAAA (0.36%)	GAAAA (0.30%)	CTTTT (0.66%)	GAAAA (0.42%)	
4	GAAAA (0.31%)	TCTTC (0.36%)	CAAAA (0.39%)	ATAAA (0.29%)	GAAAA (0.35%)	TCTTC (0.34%)	AAGAA (0.30%)	GAAAA (0.66%)	ATTTT (0.42%)	
5	CAGCA (0.30%)	TAAAA (0.36%)	CAGCA (0.38%)	AAGAA (0.29%)	TCTTT (0.33%)	CATCA (0.30%)	TAAAA (0.29%)	CAAAA (0.66%)	CAAAA (0.38%)	
6	AGAAA (0.29%)	CTTTT (0.35%)	AGAAA (0.36%)	CAGCA (0.28%)	TAAAA (0.32%)	AATAA (0.30%)	CAAAA (0.29%)	ATAAA (0.54%)	ATAAA (0.36%)	
7	AAGAA (0.28%)	AAGAA (0.35%)	ATTTT (0.35%)	GTTTT (0.27%)	ACAAA (0.31%)	GCGGC (0.30%)	AGAAA (0.27%)	TCTTT (0.53%)	TTAAA (0.34%)	
8	TTTCT (0.28%)	AGAAA (0.35%)	TAAAA (0.32%)	CAAAA (0.27%)	TCTTC (0.31%)	TAAAA (0.30%)	ATAAA (0.27%)	AAAAA (0.50%)	AGAAA (0.34%)	
9	CTTTT (0.26%)	ATAAA (0.31%)	AAAAA (0.30%)	TAAAA (0.26%)	TATTT (0.30%)	ACAAA (0.30%)	AATTT (0.27%)	TTAAA (0.49%)	TCTTC (0.33%)	
10	TCTTT (0.26%)	AAAAA (0.31%)	TGAAA (0.30%)	CATCA (0.26%)	GCGGC (0.29%)	ATTTT (0.29%)	TGAAA (0.26%)	TTTTT (0.47%)	CAGCA (0.32%)	
-10	GGACC (0.01%)	ACCTA (0.02%)	TAGGT (0.01%)	CTAGA (0.02%)	ACTAG (0.02%)	ACCTA (0.01%)	GGGAC (0.02%)	GGACC (0.02%)	GCTAG (0.02%)	Least common
-9	ACTAG (0.01%)	GGACC (0.01%)	TAGGG (0.01%)	TCTAG (0.02%)	GCTAG (0.02%)	TAGGG (0.01%)	TCGGA (0.02%)	TCCGA (0.02%)	TCTAG (0.02%)	
-8	CTAGT (0.01%)	GCTAG (0.01%)	CCCTA (0.01%)	ACCTA (0.02%)	ACCTA (0.02%)	CCCTA (0.01%)	TCTAG (0.02%)	TCTAG (0.02%)	TAGGA (0.02%)	
-7	CCTAG (0.01%)	CTAGC (0.01%)	CTAGC (0.01%)	GCTAG (0.02%)	CCCTA (0.02%)	GCTAG (0.01%)	GCTAG (0.02%)	CCCTA (0.02%)	CCCTA (0.02%)	
-6	GCTAG (0.01%)	TCTAG (0.01%)	TCTAG (0.01%)	GGGAC (0.02%)	CTAGA (0.02%)	ACTAG (0.01%)	CCCTA (0.02%)	GCTAG (0.02%)	CTAGT (0.01%)	
-5	CCCTA (0.01%)	CCCTA (0.01%)	GCTAG (0.01%)	CCCTA (0.01%)	TCTAG (0.02%)	TCTAG (0.01%)	CTAGC (0.02%)	CTAGT (0.02%)	ACGGA (0.01%)	
-4	TCTAG (0.01%)	CTAGT (0.01%)	CTAGT (0.01%)	CTAGT (0.01%)	TAGGT (0.01%)	TAGGT (0.01%)	CTAGA (0.02%)	TCGGA (0.02%)	TCGGA (0.01%)	
-3	CTAGA (0.01%)	CTAGG (0.01%)	CTAGA (0.01%)	ACTAG (0.01%)	CTAGT (0.01%)	CTAGT (0.01%)	CTAGT (0.01%)	CTAGG (0.01%)	CCTAG (0.01%)	
-2	CTAGC (0.01%)	CTAGA (0.01%)	CTAGG (0.01%)	CCTAG (0.01%)	CTAGG (0.01%)	CTAGG (0.01%)	CTAGG (0.01%)	CCTAG (0.01%)	CTAGG (0.01%)	
-1	CTAGG (0.01%)	CCTAG (0.01%)	CCTAG (0.00%)	CTAGG (0.01%)	CCTAG (0.01%)	CCTAG (0.01%)	CCTAG (0.01%)	CTAGA (0.01%)	CTAGA (0.01%)	

Kmer space for 5-mers: 1024 Random chance for any given 5-mer: 0.10%
